# Supplementary material for: Synthesis, Antifungal Activity, Cytotoxicity and QSAR Study of Camphor Derivatives
Source: J Fungi (Basel). 2022 Jul 22;8(8):762. doi: 10.3390/jof8080762 (PMC9332567; doi:10.3390/jof8080762)
Supplement: Supplementary file 1 [file jof-08-00762-s001.zip › jof-1794191-supplementary.pdf]

Supplementary Materials

Synthesis, Antifungal activity, Cytotoxicity and QSAR study of camphor derivatives

Xinying Duan[1a], Li Zhang[1a], Hongyan Si[1], Jie Song[2], Peng Wang[1], Shangxing Chen[1], Hai Luo[1], Xiaoping Rao[3], Zongde Wang[1*], Shengliang Liao[1*]

[1]College of Forestry, Jiangxi Agricultural University, East China Woody Fragrance and Flavor Engineering Research Center of National Forestry and Grassland Administration; Camphor Engineering Research Center of National Forestry and Grassland Administration/ Jiangxi Province, Nanchang 330045, China

[2]Department of Natural Sciences, University of Michigan-Flint, 303E Kearsley, Flint, MI 48502, United States

[3]College of Chemical Engineering, Huaqiao University; Xiamen 361021, China

[a]These authors contributed equally to this work.

[*]Corresponding Authors:

Zongde Wang, Email: zongdewang@163.com

Shengliang Liao, Email: liaosl@jxau.edu.cn.

Content

Structural identification analysis data of compounds 3a, 4a-4s and 5a-5o.........4

Spectrum of the title compounds...22

Figure S141. Scanning electron micrographs of mycelial morphology of *T. versicolor*: [a]blank control, ×3000; [b]treated with compound 3a at 0.43 mg/L (EC_{50}), ×3000; [c]treated with compound 4a at 6.80 mg/L (EC_{50}), ×3000; [d]treated with compound 5k at 4.86 mg/L (EC_{50}), ×3000 92

Figure S142. Photograph of antifungal experiments of tricyclazole against six fungi 93

Figure S143. Photograph of antifungal experiments of carbendazim against six fungi 94

Table S1. EC_{50} values and toxicity regression equation of camphor derivatives against *Phytophthora nicotianae* (mg/L) 95

Table S2. EC_{50} values and toxicity regression equation of camphor derivatives against *Fusarium verticillioides* (mg/L)..98

Table S3. EC_{50} values and toxicity regression equation of camphor derivatives against *Colletotrichum gloeosporioides* (mg/L) 102

Table S4. EC_{50} values and toxicity regression equation of camphor derivatives against *Sphaeropsis sapinea* (mg/L)..106

Table S5. EC_{50} values and toxicity regression equation of camphor derivatives against *Fusarium oxysporum* (mg/L) 110

Table S6. EC_{50} values and toxicity regression equation of camphor derivatives against *Trametes versicolor* (mg/L) 114
Table S7. EC_{50} values and toxicity regression equation of carbendazim against six fungi (mg/L) 120

Structural identification analysis data of compounds 3a, 4a-4s and 5a-5o.

2-(1,7,7-trimethylbicyclo[2.2.1]heptan-2-ylidene)hydrazine-1-carbothioamide (3a): A white acicular crystal, yield: 93.24%; m.p. 196 – 200°C; FT-IR *v* (cm^{-1}): 3423, 3250, 3163 (N-H), 1739, 1664 (C=N), 1304 (C=S); ^1H NMR (400 MHz, DMSO-d_6) δ 9.82 (s, 1H,), 8.04 (s, 1H), 7.41 (s, 1H), 3.35 (s, 1H), 2.02 (d, *J*=18.0 Hz, 1H), 1.92 (t, *J*=4.3 Hz, 1H), 1.78 (ddd, *J*=11.9, 8.0, 3.8 Hz, 1H), 1.72–1.66 (m, 1H), 1.30 – 1.25 (m, 1H), 1.20–1.15 (m, 1H), 0.95 (s, 3H), 0.88 (s, 3H), 0.69 (s, 3H). ^{13}C NMR (101 MHz, DMSO-d_6) δ 178.57, 166.64, 52.87, 47.99, 43.80, 35.00, 32.82, 27.20, 19.69, 18.95, 11.61. HRMS: *m/z*: $C_{11}H_{19}N_3S$, calculated, 225.1300, found, 225.1378 $[M+H]^+$.

(E)-N-2-(1,7,7-trimethylbicyclo[2.2.1]heptan-2-ylidene)hydrazine-1-carbothioamide (4a): A white acicular crystal, yield: 87.22%; m.p. 182-185 °C; FT-IR *v* (cm^{-1}): 3241, 3157 (N-H), 3064 (Ar-H), 1633 (C=N), 1579 (Benzene ring), 1247 (C=S); ^1H NMR (400 MHz, $CDCl_3$) δ 11.57 (s, 1H), 8.30 (s, 1H), 7.42 (d, *J* = 7.2 Hz, 2H), 7.35 – 7.28 (m, 3H), 4.47 (d, *J* = 12.8 Hz, 2H), 2.55 (s, 1H), 2.18 (s, 1H), 1.98 (s, 1H), 1.80 (s, 1H), 1.69 (s, 1H), 1.32 (s, 1H), 1.19 (s, 1H), 0.92 (s, 3H), 0.88 (s, 3H), 0.70 (s, 3H). ^{13}C NMR (101 MHz, $CDCl_3$) δ 175.64 (s), 171.05 (s), 132.55 (s), 129.51 (s), 129.10 (s), 128.94 (s), 128.52 (s), 128.27 (s), 53.69 (s), 48.30 (s), 44.08 (s), 36.57 (s), 35.67 (s), 32.41 (s), 26.85 (s), 19.54 (s), 18.55 (s), 10.85 (s). HRMS: *m/z*: $C_{18}H_{25}N_3S$, calculated, 315.1769, found, 315.1856 $[M+H]^+$.

(E)-N-(2-fluorophenyl)-2-(1,7,7-trimethylbicyclo[2.2.1]heptan-2-ylidene)hydrazine-1-carbothioamide (4b): A white acicular crystal, yield:

86.32%; m.p. 182-185 °C; FT-IR *v* (cm^{-1}):3247, 3162 (N-H), 3070 (Ar-H), 1636 (C=N), 1580, 1491, 1454 (Benzene ring), 1230 (C=S), 722 (*o*-disubstituted benzene ring); ^{1}H NMR (400 MHz, $CDCl_3$) *δ* 11.47 (s, 1H), 8.43 (s, 1H), 7.36 (s, 1H), 7.20 (s, 1H), 6.97 (d, *J* = 8.1 Hz, 2H), 4.43 (s, 2H), 2.44 (s, 1H), 2.05 (s, 1H), 1.86 (s, 1H), 1.68 (s, 1H), 1.57 (s, 1H), 1.20 (s, 1H), 1.08 (s, 1H), 0.79 (s, 3H), 0.75 (s, 3H), 0.58 (s, 3H). ^{13}C NMR (101 MHz, $CDCl_3$) *δ* 175.93 (s), 171.35 (s), 132.15 (s), 131.02 (d, *J* = 7.8 Hz), 125.11 (s), 116.37 (s), 116.16 (s), 54.02 (s), 48.64 (s), 44.40 (s), 36.91 (s), 32.73 (s), 29.68 (s), 27.19 (s), 19.88 (s), 18.90 (s), 11.20 (s). HRMS: *m*/*z*: $C_{18}H_{24}FN_3S$, calculated, 333.1675, found, 333.1737 $[M+H]^+$.

(E)-N-(3-fluorophenyl)-2-(1,7,7-trimethylbicyclo[2.2.1]heptan-2-ylidene)hydrazine-1-carbothioamide (4c): A white acicular crystal, yield: 85.74%; m.p. 175-180 °C; FT-IR *v* (cm^{-1}): 3262, 3186 (N-H), 3080 (Ar-H), 1629 (C=N), 1587, 1451 (Benzene ring), 1267 (C=S), 947, 790, 716 (*m*-disubstituted benzene ring); ^{1}H NMR (400 MHz, $CDCl_3$) *δ* 11.80 (s, 1H), 8.72 (s, 1H), 7.52 (d, *J* = 17.7 Hz, 2H), 7.42 (s, 1H), 7.24 (s, 1H), 4.78 (d, *J* = 2.8 Hz, 2H), 2.81 (s, 1H), 2.43 (s, 1H), 2.24 (s, 1H), 2.07 (s, 1H), 1.95 (s, 1H), 1.58 (s, 1H), 1.45 (s, 1H), 1.19 (s, 3H), 1.14 (s, 3H), 0.96 (s, 3H). ^{13}C NMR (101 MHz, $CDCl_3$) *δ* 175.78 (s), 170.71 (s), 130.67 (s), 125.27 (s), 116.47 (d, *J* = 22.1 Hz), 115.64 (s), 115.43 (s), 53.69 (s), 48.30 (s), 44.03 (s), 36.53 (s), 35.06 (s), 32.36 (s), 26.81 (s), 19.50 (s), 18.52 (s), 10.83 (s). HRMS: *m*/*z*: $C_{18}H_{24}FN_3S$, calculated, 333.1675, found, 333.1729 $[M+H]^+$.

(E)-N-(4-fluorophenyl)-2-(1,7,7-trimethylbicyclo[2.2.1]heptan-2-ylidene)hydrazine-1-carbothioamide (4d): A white acicular crystal, yield:

84.91%; m.p. 179-184 °C; FT-IR *v* (cm^{-1}): 3256, 3182 (N-H), 3077 (Ar-H), 2961, 2868 (C-H), 1630 (C=N), 1585, 1509, 1449 (Benzene ring), 1227 (C=S), 841 (*p*-disubstituted benzene ring); ^{1}H NMR (400 MHz, $CDCl_3$) δ 11.80 (s, 1H), 8.72 (s, 1H), 7.54 (s, 1H), 7.50 (s, 1H), 7.42 (s, 1H), 7.24 (s, 1H), 4.78 (s, *J* = 2.8 Hz, 2H), 2.81 (s, 1H), 2.43 (s, 1H), 2.24 (s, 1H), 2.07 (s, 1H), 1.95 (s, 1H), 1.58 (s, 1H), 1.45 (s, 1H), 1.19 (s, 3H), 1.14 (s, 3H), 0.96 (s, 3H). ^{13}C NMR (101 MHz, $CDCl_3$) δ 175.59 (s), 170.76 (s), 131.35 (d, *J* = 8.1 Hz), 128.39 (s), 116.11 (s), 115.89 (s), 115.61 (s), 53.64 (s), 48.26 (s), 44.01 (s), 36.48 (s), 34.87 (s), 32.34 (s), 26.80 (s), 19.48 (s), 18.49 (s), 10.81 (s). HRMS: *m*/*z*: $C_{18}H_{24}FN_3S$, calculated, 333.1675, found, 333.1731 $[M+H]^+$.

(E)-N-(2-(trifluoromethyl)benzyl)-2-(1,7,7-trimethylbicyclo[2.2.1]heptan-2-ylidene)hydrazine-1-carbothioamide (4e): A white acicular crystal, yield: 81.34%; m.p. 161-165 °C; FT-IR *v* (cm-1): 3220, 3162 (N-H), 3066 (Ar-H), , 1639 (C=N), 1582, 1426 (Benzene ring), 1313, 1151 (C-F), 1116 (C=S), 729 (o-disubstituted benzene ring); ^{1}H NMR (400 MHz, $CDCl_3$) δ 11.85 (s, 1H), 8.79 (s, 1H), 7.90 (s, 2H), 7.80 (s, 1H), 7.68 (s, 1H), 4.90 (s, 2H), 2.82 (s, 1H), 2.44 (s, 1H), 2.24 (s, 1H), 2.06 (s, 1H), 1.95 (s, 1H), 1.58 (s, 1H), 1.46 (s, 1H), 1.16 (s, 3H), 1.13 (s, 3H), 0.96 (s, 3H). ^{13}C NMR (101 MHz, $CDCl_3$) δ 176.32 (s), 171.16 (s), 133.30 (s), 133.03 (s), 131.51 (s), 129.24 (s), 127.06 (s), 125.69 (s), 122.97 (s), 54.12 (s), 48.72 (s), 44.47 (s), 36.96 (s), 32.87 (d, *J* = 17.5 Hz), 27.22 (s), 19.92 (s), 18.93 (s), 11.20 (s). HRMS: *m*/*z*: $C_{19}H_{24}F_3N_3S$, calculated, 383.1643, found, 383.1721 $[M+H]^+$.

(E)-N-(4-(trifluoromethyl)benzyl)-2-(1,7,7-trimethylbicyclo[2.2.1]heptan-2-ylidene)hydrazine-1-carbothioamide (4f): A white acicular crystal, yield: 80.72%; m.p. 167-171 °C; FT-IR *v* (cm-1): 3253, 3191 (N-H), 3082 (Ar-H), 1632 (C=N), 1587, 1458, 1421 (Benzene ring), 1326, 1166 (C-F), 1128 (C=S), 895 (p-disubstituted benzene ring); ^{1}H NMR (400 MHz, $CDCl_3$) δ 11.78 (s, 1H), 8.87 (s, 1H), 7.84 (s, 4H), 4.86 (s, 2H), 2.80 (s, 1H), 2.42 (s, 1H), 2.24 (s, 1H), 2.07 (s, 1H), 1.96 (s, 1H), 1.58 (s, 1H), 1.45 (s, 1H), 1.19 (s, 3H), 1.14 (s, 3H), 0.96 (s, 3H). ^{13}C NMR (101 MHz, $CDCl_3$) δ 176.38 (s), 171.02 (s), 137.44 (s), 131.21(s), 130.28 (d, *J* = 15.0 Hz), 126.24 (d, *J* = 27.1 Hz), 125.58(s), 54.14 (s), 48.73 (s), 44.45 (s), 36.94 (s), 35.38 (s), 32.76 (s), 27.23 (s), 19.89 (s), 18.89 (s), 11.20 (s). HRMS: *m*/*z*: $C_{19}H_{24}F_3N_3S$, calculated, 383.1643, found, 383.1718 $[M+H]^+$.

(E)-N-(2-bromobenzyl)-2-(1,7,7-trimethylbicyclo[2.2.1]heptan-2-ylidene)hydrazine-1-carbothioamide (4g): A white acicular crystal, yield: 84.22%; m.p. 180-184 °C; FT-IR *v* (cm-1): 3241, 3170 (N-H), 3064 (Ar-H), 1639 (C=N), 1583, 1474, 1424 (Benzene ring), 1107 (C=S), 721 (o-disubstituted benzene ring); ^{1}H NMR (400 MHz, $CDCl_3$) δ 8.86 (s, 1H), 7.50 (d, *J* = 6.7 Hz, 2H), 7.22 (d, *J* = 16.2 Hz, 2H), 7.12 (s, 1H), 4.54 (s, 2H), 2.56 (s, 1H), 2.14 (s, 1H), 1.94 (s, 1H), 1.76 (s, 1H), 1.65 (s, 1H), 1.28 (s, 1H), 1.15 (s, 1H), 0.87 (s, 3H), 0.83 (s, 3H), 0.65 (s, 3H). ^{13}C NMR (101 MHz, $CDCl_3$) δ 175.83 (s), 170.99 (s), 133.44 (s), 132.46 (s), 131.92 (s), 130.33 (s), 128.19 (s), 125.06 (s), 53.17 (s), 48.33 (s), 44.15 (s), 36.57 (s), 36.22 (s), 32.40 (s), 26.85 (s), 19.56 (s), 18.54 (s), 10.85 (s). HRMS: *m*/*z*: $C_{18}H_{24}BrN_3S$, calculated, 393.0874, found, 393.0939 $[M+H]^+$.

(E)-N-(3-bromobenzyl)-2-(1,7,7-trimethylbicyclo[2.2.1]heptan-2-ylidene)hydrazine-1-carbothioamide (4h): A taupe acicular crystal, yield: 81.27%; m.p. 171-174 °C; FT-IR *v* (cm-1): 3227, 3172 (N-H), 3065 (Ar-H), 1638 (C=N), 1586, 1474, 1426 (Benzene ring), 1111(C=S), 891, 772, 725 (m-disubstituted benzene ring); ^{1}H NMR (400 MHz, $CDCl_3$) δ 11.48 (s, 1H), 8.36 (s, 1H), 7.53 (s, 1H), 7.37 (s, 1H), 7.33 (s, 1H), 7.15 (s, 1H), 4.42 (s, 2H), 2.50 (s, 1H), 2.13 (s, 1H), 1.95 (s, 1H), 1.77 (s, 1H), 1.66 (s, 1H), 1.28 (s, 1H), 1.15 (s, 1H), 0.88 (s, 3H), 0.84 (s, 3H), 0.66 (s, 3H). ^{13}C NMR (101 MHz, $CDCl_3$) δ 176.02 (s), 170.69 (s), 134.91 (s), 132.42 (s), 131.72 (s), 130.60 (s), 128.24 (s), 122.98 (s), 53.76 (s), 48.36 (s), 44.07 (s), 36.56 (s), 34.95 (s), 32.40 (s), 26.85 (s), 19.56 (s), 18.54 (s), 10.86 (s). HRMS: *m*/*z*: $C_{18}H_{24}BrN_3S$, calculated, 393.0874, found, 393.0942 $[M+H]^+$.

(E)-N-(2-chlorobenzyl)-2-(1,7,7-trimethylbicyclo[2.2.1]heptan-2-ylidene)hydrazine-1-carbothioamide (4i): A white acicular crystal, yield: 83.16%; m.p. 173-175 °C; FT-IR *v* (cm-1): 3240, 3169 (N-H), 3062 (Ar-H), 1638 (C=N), 1583, 1474, 1424 (Benzene ring), 1106(C=S), 721 (o-disubstituted benzene ring); ^{1}H NMR (400 MHz, $CDCl_3$) δ 11.52 (s, 1H), 8.41 (s, 1H), 7.48 (s, 1H), 7.30 (s, 1H), 7.19 (d, *J* = 4.4 Hz, 2H), 4.52 (s, 2H), 2.49 (s, 1H), 2.12 (s, 1H), 1.92 (s, 1H), 1.73 (s, 1H), 1.63 (s, 1H), 1.26 (s, 1H), 1.14 (s, 1H), 0.85 (s, 3H), 0.81 (s, 3H), 0.64 (s, 3H). ^{13}C NMR (101 MHz, $CDCl_3$) δ 175.69 (s), 171.05 (s), 134.77 (s), 131.91 (s), 130.71 (s), 130.11 (d, *J* = 7.1 Hz), 127.53 (s), 53.69 (s), 48.31 (s), 44.06 (s), 36.53 (s), 33.58 (s), 32.38 (s), 26.84 (s), 19.54 (s), 18.53 (s), 10.84 (s). HRMS: *m*/*z*: $C_{18}H_{24}ClN_3S$, calculated, 349.1379, found, 349.1454 $[M+H]^+$.

(E)-N-(3-chlorobenzyl)-2-(1,7,7-trimethylbicyclo[2.2.1]heptan-2-ylidene)hydrazine-1-carbothioamide (4j): A white acicular crystal, yield: 85.43%; m.p. 172-176 °C; FT-IR *v* (cm-1): 3225, 3174 (N-H), 3066 (Ar-H), 1638 (C=N), 1587, 1474, 1430 (Benzene ring), 1110 (C=S), 880, 785, 725 (m-disubstituted benzene ring); ^{1}H NMR (400 MHz, $CDCl_3$) δ 11.51 (s, 1H), 8.31 (s, 1H), 7.38 (s, 1H), 7.29 (s, 1H), 7.22 (d, *J* = 5.8 Hz, 2H), 7.19 (s, 1H), 4.42 (s, 2H), 2.51 (s, 1H), 2.13 (s, 1H), 1.95 (s, 1H), 1.77 (s, 1H), 1.67 (s, 1H), 1.29 (s, 1H), 1.16 (s, 1H), 0.89 (s, 3H), 0.84 (s, 3H), 0.66 (s, 3H). ^{13}C NMR (101 MHz, $CDCl_3$) δ 176.00 (s), 170.71 (s), 134.89 (s), 134.68 (s), 130.34 (s), 129.52 (s), 128.79 (s), 127.74 (s), 53.76 (s), 48.35 (s), 44.09 (s), 36.54 (s), 34.96 (s), 32.41 (s), 26.85 (s), 19.54 (s), 18.53 (s), 10.84 (s). HRMS: *m*/*z*: $C_{18}H_{24}ClN_3S$, calculated, 349.1379, found, 349.1445 $[M+H]^+$.

(E)-N-(4-chlorobenzyl)-2-(1,7,7-trimethylbicyclo[2.2.1]heptan-2-ylidene)hydrazine-1-carbothioamide (4k): A yellow acicular crystal, yield: 82.25%; m.p. 151-154 °C; FT-IR *v* (cm-1): 3226, 3170 (N-H), 3067 (Ar-H), 1637 (C=N), 1585, 1490, 1421 (Benzene ring), 1103 (C=S), 896 (p-disubstituted benzene ring); ^{1}H NMR (400 MHz, $CDCl_3$) δ 11.45 (s, 1H), 8.42 (s, 1H), 7.29 (d, *J* = 8.3 Hz, 2H), 7.20 (d, *J* = 6.8 Hz, 2H), 4.40 (d, *J* = 2.2 Hz, 2H), 2.46 (s, 1H), 2.08 (s, 1H), 1.90 (s, 1H), 1.73 (s, 1H), 1.62 (s, 1H), 1.24 (s, 1H), 1.11 (s, 1H), 0.84 (s, 3H), 0.80 (s, 3H), 0.62 (s, 3H). ^{13}C NMR (101 MHz, $CDCl_3$) δ 175.76 (s), 170.71 (s), 134.63 (s), 131.19 (s), 130.90 (s), 129.23 (s), 128.97 (s), 128.64 (s), 53.70 (s), 48.30

(s), 44.04 (s), 36.52 (s), 34.89 (s), 32.37 (s), 26.84 (s), 19.52 (s), 18.53 (s), 10.83 (s). HRMS: *m/z*: $C_{18}H_{24}ClN_3S$, calculated, 349.1379, found, 349.1456 $[M+H]^+$.

(E)-N-(2,5-difluorobenzyl)-2-(1,7,7-trimethylbicyclo[2.2.1]heptan-2-ylidene)hydrazine-1-carbothioamide (4l): A white acicular crystal, yield: 83.15%; m.p. 154-158 °C; FT-IR *v* (cm-1): 3263, 3169 (N-H), 3082 (Ar-H), 1639 (C=N), 1583, 1496, 1450, 1419 (Benzene ring), 1139 (C=S), 833 (1,2,5-trisubstituted benzene ring); ^{1}H NMR (400 MHz, $CDCl_3$) δ 11.84 (s, 1H), 8.96 (s, 1H), 7.51 (s, 1H), 7.26 (d, *J* = 4.0 Hz, 2H), 4.81 (s, 2H), 2.81 (s, 1H), 2.43 (s, 1H), 2.25 (s, 1H), 2.06 (s, 1H), 1.96 (s, 1H), 1.58 (s, 1H), 1.46 (s, 1H), 1.21 (d, *J* = 22.2 Hz, 3H), 1.14 (s, 3H), 0.97 (d, *J* = 9.0 Hz, 3H). ^{13}C NMR (101 MHz, $CDCl_3$) δ 176.23 (s), 171.10 (s), 118.56 (s), 118.31 (s), 117.42 (dd, *J* = 24.0, 8.2 Hz), 54.09 (s), 48.68 (s), 44.43 (s), 36.95 (s), 32.74 (s), 29.44 (s), 27.21 (s), 19.87 (s), 18.91 (s), 11.20 (s). HRMS: *m/z*: $C_{18}H_{23}F_2N_3S$, calculated, 351.1581, found,351.1646 $[M+H]^+$.

(E)-N-(3,5-difluorobenzyl)-2-(1,7,7-trimethylbicyclo[2.2.1]heptan-2-ylidene)hydrazine-1-carbothioamide (4m): A white acicular crystal, yield: 82.95%; m.p. 159-164 °C; FT-IR *v* (cm-1): 3259, 3186 (N-H), 3082 (Ar-H), 1630 (C=N), 1594, 1463 (Benzene ring), 1120 (C=S), 849 (1,3,5-trisubstituted benzene ring); ^{1}H NMR (400 MHz, $CDCl_3$) δ 11.75 (s, 1H), 8.72 (s, 1H), 7.27 (d, *J* = 6.3 Hz, 2H), 6.99 (s, 1H), 4.77 (d, *J* = 13.5 Hz, 2H), 2.79 (s, 1H), 2.41 (s, 1H), 2.25 (s, 1H), 2.07 (s, 1H), 1.97 (s, 1H), 1.58 (s, 1H), 1.46 (s, 1H), 1.19 (s, 3H), 1.14 (s, 3H), 0.96 (s, 3H). ^{13}C NMR (101 MHz, $CDCl_3$) δ 170.83 (s), 136.99 (s), 112.93 (dd, *J* = 18.8, 7.2 Hz), 104.50 (s), 54.16 (s), 48.74 (s), 44.44 (s), 36.90 (s), 35.04 (s), 32.75 (s), 27.20

(s), 19.89 (s), 18.90 (s), 11.20 (s). HRMS: *m/z*: $C_{18}H_{23}F_2N_3S$, calculated, 351.1581, found, 351.1649 $[M+H]^+$.

(E)-N-(2,4-difluorobenzyl)-2-(1,7,7-trimethylbicyclo[2.2.1]heptan-2-ylidene)hydrazine-1-carbothioamide (4n): A white acicular crystal, yield: 82.78%; m.p. 153-157 °C; FT-IR *v* (cm^{-1}): 3244, 3170 (N-H), 3067 (Ar-H), 2958, 2929 (C-H), 1639 (C=N), 1584, 1505, 1422 (Benzene ring), 1135 (C=S), 858 (1,2,4-trisubstituted benzene ring); ^1H NMR (400 MHz, $CDCl_3$) δ 11.77 (s, 1H), 8.87 (s, 1H), 7.77 (s, 1H), 7.09 (d, *J* = 12.2 Hz, 2H), 4.79 (s, 2H), 2.80 (s, 1H), 2.42 (s, 1H), 2.25 (s, 1H), 2.07 (s, 1H), 1.96 (s, 1H), 1.59 (s, 1H), 1.46 (s, 1H), 1.18 (s, 3H), 1.14 (s, 3H), 0.96 (s, 3H). ^{13}C NMR (101 MHz, $CDCl_3$) δ 176.20 (s), 171.22 (s), 133.02 (s), 112.51 (s), 112.30 (s), 105.13 (s), 104.76 (d, *J* = 25.3 Hz), 54.10 (s), 48.70 (s), 44.44 (s), 36.90 (s), 32.75 (s), 29.29 (s), 27.22 (s), 19.90 (s), 18.91 (s), 11.20 (s). HRMS: *m/z*: $C_{18}H_{23}F_2N_3S$, calculated, 351.1581, found, 351.1647 [M+H]+.

(E)-N-(4-methylbenzyl)-2-(1,7,7-trimethylbicyclo[2.2.1]heptan-2-ylidene)hydrazine-1-carbothioamide (4o): A white acicular crystal, yield: 83.22%; m.p. 160-164 °C; FT-IR *v* (cm^{-1}): 3253, 3191 (N-H), 3082 (Ar-H), 1632 (C=N), 1587, 1458, 1421 (Benzene ring), 1326, 1166 (C-F), 1128 (C=S), 895 (*p*-disubstituted benzene ring); ^1H NMR (400 MHz, $CDCl_3$) δ 11.85 (s, 1H), 8.39 (s, 1H), 7.56 (s, 1H), 7.54 (s, 1H), 7.40 (s, 1H), 7.38 (s, 1H), 4.68 (s, 2H), 2.81 (s, 1H), 2.57 (s, 3H), 2.44 (s, 1H), 2.25 (s, 1H), 2.06 (s, 1H), 1.95 (s, 1H), 1.58 (s, 1H), 1.46 (s, 1H), 1.17 (s, 3H), 1.14 (s, 3H), 0.96 (s, 3H). ^{13}C NMR (101 MHz, $CDCl_3$) δ 175.95 (s), 171.46 (s), 138.81 (s), 130.18 (s), 130.02 (s), 129.93 (s), 129.72 (s), 128.52 (s),

54.03 (s), 48.65 (s), 44.43 (s), 36.90 (s), 35.77 (s), 32.76 (s), 27.19 (s), 21.56 (s), 19.89 (s), 18.90 (s), 11.20 (s). HRMS: *m/z*: $C_{19}H_{27}N_3S$, calculated, 329.1926, found, 329.2007 $[M+H]^+$.

(E)-N-(2-nitrobenzyl)-2-(1,7,7-trimethylbicyclo[2.2.1]heptan-2-ylidene)hydrazine-1-carbothioamide (4p): A yellow acicular crystal, yield: 79.55%; m.p. 116-121 °C; FT-IR *v* (cm-1): 3477, 3449, 3359, 3329 (N-H), 3073 (Ar-H), 1654 (C=N), 1590, 1523, 1446 (Benzene ring), 1197 (C=S), 1343 (C-NO2), 753 (o-disubstituted benzene ring); ^1H NMR (400 MHz, $CDCl_3$) δ 8.15 (s, 1H), 7.93 (s, 1H), 7.70 (s, 1H), 7.56 (s, 1H), 5.33 (s, 2H), 4.77 (s, 2H), 2.74 (s, 1H), 2.31 (s, 1H), 2.09 (s, 1H), 2.03 (s, 1H), 1.90 (s, 1H), 1.59 (s, 1H), 1.42 (s, 1H), 1.18 (s, 3H), 1.11 (s, 3H), 0.96 (s, 3H). ^{13}C NMR (101 MHz, $CDCl_3$) δ 176.82(s), 156.27(s), 148.07(s), 134.42(s), 132.94 (s), 132.58 (s), 127.90 (s), 124.70 (s), 52.25(s), 47.59 (s), 43.72 (s), 35.75 (s), 32.64 (s), 30.41 (s), 27.15 (s), 19.42 (s), 18.60 (s), 11.20 (s). HRMS: *m/z*: $C_{18}H_{24}N_4S$, calculated, 360.1620, found, 360.1688 $[M+H]^+$.

(E)-N-(3-nitrobenzyl)-2-(1,7,7-trimethylbicyclo[2.2.1]heptan-2-ylidene)hydrazine-1-carbothioamide (4q): A taupe acicular crystal, yield: 78.15%; m.p. 114-118 °C; FT-IR *v* (cm-1): 3236 (N-H), 3067 (Ar-H), 1634 (C=N), 1591, 1449 (Benzene ring), 1128 (C=S), 1326 (C-NO2), 895, 848, 717 (m-disubstituted benzene ring); ^1H NMR (400 MHz, $CDCl_3$) δ 12.10 (s, 1H), 9.61 (s, 1H), 7.84 (s, 4H), 4.85 (s, 2H), 2.83 (s, 1H), 2.40 (s, 1H), 2.22 (s, 1H), 2.07 (s, 1H), 1.97 (s, 1H), 1.58 (s, 1H), 1.44 (s, 1H), 1.19 (s, 3H), 1.15 (s, 3H), 0.96 (s, 3H). ^{13}C NMR (101 MHz, $CDCl_3$) δ 175.94 (s), 170.91 (s), 137.72 (s), 130.29 (d, J = 16.0 Hz),

126.34 (d, *J* = 2.7 Hz), 126.08 (s), 54.07 (s), 48.71 (s), 44.43 (s), 36.59 (s), 35.12 (s), 32.78 (s), 27.25 (s), 19.88 (s), 18.89 (s), 11.20 (s). HRMS: *m*/*z*: $C_{18}H_{24}N_4S$, calculated, 360.1620, found, 360.1688 $[M+H]^+$.

(E)-N-(2-fluoro-5-(trifluoromethyl)benzyl)-2-(1,7,7-trimethylbicyclo[2.2.1]heptan-2-ylidene)hydrazine-1-carbothioamide (4r): A buff acicular crystal, yield: 81.42%; m.p. 155-159 °C; FT-IR *v* (cm-1): 3246, 3174 (N-H), 3066 (Ar-H), 1642(C=N), 1588, 1504, 1417 (Benzene ring), 1332, 1167 (C-F), 1128 (C=S), 839 (1,2,5-trisubstituted benzene ring); ^{1}H NMR (400 MHz, $CDCl_3$) δ 11.82 (s, 1H), 9.58 (s, 1H), 7.93 (s, 1H), 7.63 (s, 1H), 7.51 (s, 1H), 4.91 (s, 2H), 2.80 (s, 1H), 2.42 (s, 1H), 2.25 (s, 1H), 2.07 (s, 1H), 1.96 (s, 1H), 1.58 (s, 1H), 1.46 (s, 1H), 1.16 (s, 3H), 1.14 (s, 3H), 0.96 (s, 3H). ^{13}C NMR (101 MHz, $CDCl_3$) δ 176.52 (s), 171.00 (s), 129.72 (s), 128.52 (s), 125.09 (s), 121.80 (d, *J* = 15.9 Hz), 117.11 (d, *J* = 22.5 Hz), 54.15 (s), 48.74 (s), 44.45 (s), 36.92 (s), 32.76 (s), 29.57 (s), 27.21 (s), 19.89 (s), 18.90 (s), 11.20 (s). HRMS: *m*/*z*: $C_{19}H_{23}F_4N_4S$, calculated, 401.1549, found, 401.1629 $[M+H]^+$.

(E)-N-(4-fluoro-2-(trifluoromethyl)benzyl)-2-(1,7,7-trimethylbicyclo[2.2.1]heptan-2-ylidene)hydrazine-1-carbothioamide (4s): A white acicular crystal, yield: 80.75%; m.p. 155-157 °C; FT-IR *v* (cm-1): 3220, 3167 (N-H), 3066 (Ar-H), 1641 (C=N), 1583, 1508, 1430 (Benzene ring), 1316, 1181 (C-F), 1129 (C=S), 843 (1,2,4-trisubstituted benzene ring); ^{1}H NMR (400 MHz, $CDCl_3$) δ 11.82 (s, 1H), 9.58 (s, 1H), 7.93 (s, 1H), 7.63 (s, 1H), 7.51 (s, 1H), 4.91 (s, 2H), 2.80 (s, 1H), 2.42 (s, 1H), 2.25 (s, 1H), 2.07 (s, 1H), 1.96 (s, 1H), 1.58 (s, 1H),

1.46 (s, 1H), 1.16 (s, 3H), 1.14 (s, 3H), 0.96 (s, 3H). ^{13}C NMR (101 MHz, $CDCl_3$) *δ* 176.37 (s), 171.03 (s), 135.64 (d, *J* = 8.0 Hz), 127.55 (s), 120.08 (d, *J* = 21.2 Hz), 114.96 (s), 114.76 (s), 54.16 (s), 48.75 (s), 44.49 (s), 36.92 (s), 32.79 (s), 32.40 (s), 27.25 (s), 19.92 (s), 18.94 (s), 11.20 (s). HRMS: *m*/*z*: $C_{19}H_{23}F_4N_4S$, calculated, 401.1549, found, 401.1641 $[M+H]^+$.

(E)-4-phenyl-2-(2-(1,7,7-trimethylbicyclo[2.2.1]heptan-2-ylidene)hydrazinyl)thiazole (5a): A white acicular crystal, yield: 69.51%; m.p. 153 – 157°C; FT-IR *v* (cm-1): 3448, 3237 (N-H), 3034 (Ar-H), 1623 (C=N), 1493, 1445 ((Benzene ring), 1020 (C-S-C); ^{1}H NMR (400 MHz, DMSO-d_6) *δ* 10.78 (s, 2H), 7.82 (d, *J* = 7.8 Hz, 2H), 7.41 (t, *J* = 7.5 Hz, 2H), 7.31 (t, *J* = 7.1 Hz, 1H), 7.25 (s, 1H), 2.04 (d, *J* = 17.7 Hz, 1H), 1.97 (s, 1H), 1.82 (s, 1H), 1.73 (s, 1H), 1.33 (s, 1H), 1.22 (s, 1H), 0.98 (s, 3H), 0.91 (s, 3H), 0.73 (s, 3H). ^{13}C NMR (101 MHz, DMSO-d_6) *δ* 170.39 (s), 129.10 (s), 128.19 (s), 126.06 (s), 103.86 (s), 52.71 (s), 48.16 (s), 43.88 (s), 35.30 (s), 32.90 (s), 27.30 (s), 19.71 (s), 18.98 (s), 11.68 (s). HRMS: *m*/*z*: $C_{19}H_{23}N_3S$, calculated, 325.1613, found, 325.1675 $[M+H]^+$.

(E)-4-(2-fluorophenyl)-2-(2-(1,7,7-trimethylbicyclo[2.2.1]heptan-2-ylidene)hydrazinyl)thiazole (5b): A white acicular crystal, yield: 88.34%; m.p. 120 – 125°C; FT-IR *v* (cm^{-1}): 3458, 3133 (N-H), 3068 (Ar-H), 1620 (C=N), 1511,1471 (Benzene ring), 1074 (C-S-C), 751 (o-disubstituted benzene ring); ^{1}H NMR (400 MHz, $CDCl_3$) *δ* 12.19 (s, 1H), 7.88 (t, *J* = 7.8 Hz, 1H), 7.46 – 7.38 (m, 1H), 7.32 (t, *J* = 7.6 Hz, 1H), 7.24 – 7.17 (m, 1H), 6.99 (s, 1H), 2.69 (s, 1H), 2.30 (s, 1H), 2.09 (s, 1H), 1.90 (s, 1H), 1.79 (s, 1H), 1.41 (s, 1H), 1.30 (s, 1H), 1.04 (s, 3H),

0.96 (s, 3H), 0.78 (s, 3H). ^{13}C NMR (101 MHz, $CDCl_3$) δ 175.88 (s), 168.70 (s), 131.54 (s), 127.71 (s), 125.48 (s), 116.74 (s), 116.52 (s), 105.09 (d, *J* = 14.8 Hz), 53.63 (s), 48.55 (s), 44.07 (s), 36.40 (s), 32.54 (s), 26.87 (s), 19.49 (s), 18.54 (s), 10.77 (s). HRMS: *m/z*: $C_{19}H_{22}FN_3S$, calculated, 343.1518, found, 343.1590 $[M+H]^+$.

(E)-4-(3-fluorophenyl)-2-(2-(1,7,7-trimethylbicyclo[2.2.1]heptan-2-ylidene)hydrazinyl)thiazole (5c): A yellowish acicular crystal, yield: 89.97%; m.p. 154 – 158°C; FT-IR *v* (cm^{-1}): 3450, 3240, 3158 (N-H), 3080 (Ar-H), 1622 (C=N), 1498,1421 (Benzene ring), 1037 (C-S-C), 867, 787, 744 (m-disubstituted benzene ring); ^1H NMR (400 MHz, $CDCl_3$) δ 12.07 (s, 1H), 7.50 (s, 1H), 7.41 (d, *J* = 17.6 Hz, 3H), 7.09 (s, 1H), 6.84 (s, 1H), 2.67 (d, *J* = 17.2 Hz, 1H), 2.25 (s, 1H), 2.06 (s, 1H), 1.83 (d, *J* = 38.9 Hz, 2H), 1.38 (s, 1H), 1.27 (s, 1H), 1.01 (s, 3H), 0.93 (s, 3H), 0.76 (s, 3H). ^{13}C NMR (101 MHz, $CDCl_3$) δ 170.80 (s), 165.82 (s), 163.35 (s), 140.50 (s), 133.11 (s), 130.84 (s), 122.96 (s), 114.38 (s), 114.14 (s), 103.79 (s), 55.23 (s), 50.16 (s), 45.62 (s), 37.90 (s), 34.11 (s), 28.46 (s), 21.08 (s), 20.14 (s), 12.37 (s). HRMS: *m/z*: $C_{19}H_{22}FN_3S$, calculated, 343.1518, found, 343.1590 $[M+H]^+$.

(E)-4-(4-fluorophenyl)-2-(2-(1,7,7-trimethylbicyclo[2.2.1]heptan-2-ylidene)hydrazinyl)thiazole (5d): A white acicular crystal, yield: 89.74%; m.p. 160 – 165°C; FT-IR *v* (cm^{-1}): 3419, 3235 (N-H), 3034 (Ar-H), 1623 (C=N), 1499 (Benzene ring), 1034 (C-S-C), 842 (p-disubstituted benzene ring); ^1H NMR (400 MHz, $CDCl_3$) δ 12.00 (s, 1H), 7.70 (d, *J* = 2.1 Hz, 2H), 7.15 (d, *J* = 8.3 Hz, 2H), 6.70 (s, 1H), 2.66 (s, 1H), 2.26 (s, 1H), 2.07 (s, 1H), 1.88 (s, 1H), 1.77 (d, *J* = 9.5 Hz, 1H), 1.39 (s, 1H), 1.26 (d, *J* = 9.5 Hz, 1H), 1.02 (s, 3H), 0.94 (s, 3H), 0.77 (s, 3H). ^{13}C

NMR (101 MHz, $CDCl_3$) δ 174.79 (s), 168.20 (s), 138.15 (s), 126.66 (d, J = 8.5 Hz), 116.19 (d, J = 16.9 Hz), 115.72 (d, J = 22.3 Hz), 99.46 (s), 52.60 (s), 47.53 (s), 43.02 (s), 35.27 (s), 31.50 (s), 25.85 (s), 18.47 (s), 17.52 (s), 9.74 (s). HRMS: *m*/*z*: $C_{19}H_{22}FN_3S$, calculated, 343.1518, found, 343.1596 $[M+H]^+$.

(E)-4-(3-bromophenyl)-2-(2-(1,7,7-trimethylbicyclo[2.2.1]heptan-2-ylidene)hydrazinyl)thiazole (5e): A white acicular crystal, yield: 92.11%; m.p. 140 – 143°C; FT-IR v (cm^{-1}): 3450, 3219 (N-H), 3098, 3039 (Ar-H), 1617 (C=N), 1478,1441 (Benzene ring), 1075 (C-S-C), 891, 798, 735 (m-disubstituted benzene ring); ^1H NMR (400 MHz, $CDCl_3$) δ 7.79 (s, 1H), 7.67 (d, J = 7.8 Hz, 1H), 7.52 (d, J = 7.3 Hz, 1H), 7.34 (d, J = 7.9 Hz, 1H), 6.82 (s, 1H), 2.66 (s, 1H), 2.26 (s, 1H), 2.07 (s, 1H), 1.88 (s, 1H), 1.78 (s, 1H), 1.39 (s, 1H), 1.28 (s, 1H), 1.02 (s, 3H), 0.94 (s, 3H), 0.77 (s, 3H). ^{13}C NMR (101 MHz, $CDCl_3$) δ 177.39 (s), 170.85 (s), 140.44 (s), 134.77 (s), 132.77 (s), 130.99 (s), 130.09 (s), 125.79 (s), 125.02 (s), 103.83 (s), 55.24 (s), 50.16 (s), 45.64 (s), 37.87 (s), 34.12 (s), 28.48 (s), 21.11 (s), 20.15 (s), 12.39 (s). HRMS: *m*/*z*: $C_{19}H_{22}BrN_3S$, calculated, 403.0718, found, 403.0780 $[M+H]^+$.

(E)-4-(4-bromophenyl)-2-(2-(1,7,7-trimethylbicyclo[2.2.1]heptan-2-ylidene)hydrazinyl)thiazole (5f): A white acicular crystal, yield: 49.48%; m.p. 173 – 177°C; FT-IR v (cm^{-1}): 3380, 3321, 3231 (N-H), 3052 (Ar-H), 1621 (C=N), 1483 (Benzene ring), 1072 (C-S-C), 824 (p-disubstituted benzene ring); ^1H NMR (400 MHz, $CDCl_3$) δ 12.06 (s, 1H), 7.60 (s, 4H), 6.75 (s, 1H), 2.70 (d, J = 17.8 Hz, 1H), 2.26 (d, J = 17.8 Hz, 1H), 2.09 (s, 1H), 1.89 (d, J = 12.9 Hz, 1H), 1.79 (d, J = 9.8 Hz, 1H), 1.41 (dd, J = 15.6, 6.5 Hz, 1H), 1.33 – 1.27 (m, 1H), 1.04 (s, 3H), 0.96

(s, 3H), 0.79 (s, 3H). ^{13}C NMR (101 MHz, $CDCl_3$) δ 174.92 (s), 168.16 (s), 138.06 (s), 131.83 (d, J = 23.3 Hz), 126.10 (d, J = 25.8 Hz), 125.19 (s), 123.58 (s), 100.38 (s), 52.63 (s), 47.55 (s), 43.01 (s), 35.27 (s), 31.50 (s), 25.86 (s), 18.49 (s), 17.53 (s), 9.77 (s). HRMS: *m/z*: $C_{19}H_{22}BrN_3S$, calculated, 403.0718, found, 403.0782 $[M+H]^+$.

(E)-4-(4-chlorophenyl)-2-(2-(1,7,7-trimethylbicyclo[2.2.1]heptan-2-ylidene)hydrazinyl)thiazole (5g): A white acicular crystal, yield: 72.04%; m.p. 190 – 193°C; FT-IR v (cm^{-1}): 3450, 3234 (N-H), 3040 (Ar-H), 1621 (C=N), 1484 (Benzene ring), 1093 (C-S-C), 836 (p-disubstituted benzene ring); ^1H NMR (400 MHz, $CDCl_3$) δ 12.05 (s, 1H), 7.65 (d, J = 8.5 Hz, 2H), 7.43 (d, J = 8.4 Hz, 2H), 6.75 (d, J = 1.8 Hz, 1H), 2.69 (d, J = 17.5 Hz, 1H), 2.25 (d, J = 17.8 Hz, 1H), 2.09 (t, J = 4.0 Hz, 1H), 1.88 (dd, J = 12.1, 2.8 Hz, 1H), 1.80 (td, J = 12.1, 3.2 Hz, 1H), 1.44 – 1.37 (m, 1H), 1.33 – 1.26 (m, 1H), 1.04 (s, 3H), 0.96 (s, 3H), 0.78 (s, 3H). ^{13}C NMR (101 MHz, $CDCl_3$) δ 174.94 (s), 168.19 (s), 138.08 (s), 135.37 (s), 128.87 (d, J = 16.5 Hz), 125.73 (d, J = 15.3 Hz), 124.78 (s), 100.11 (s), 52.63 (s), 47.56 (s), 43.02 (s), 35.30 (s), 31.51 (s), 25.86 (s), 18.49 (s), 17.53 (s), 9.76 (s). HRMS: *m/z*: $C_{19}H_{22}ClN_3S$, calculated, 359.1223, found, 359.1302 $[M+H]^+$.

(E)-4-(3-nitrophenyl)-2-(2-(1,7,7-trimethylbicyclo[2.2.1]heptan-2-ylidene)hydrazinyl)thiazole (5h): A viridescent acicular crystal, yield: 52.15%; m.p. 195 – 199°C; FT-IR v (cm^{-1}): 3275, (N-H), 3071 (Ar-H), 1616 (C=N), 1507, 1470 (Benzene ring), 1074 (C-S-C), 911, 771, 745 (m-disubstituted benzene ring); ^1H NMR (400 MHz, $CDCl_3$) δ 12.05 (s, 1H), 8.52 (s, 1H), 8.27 (dd, J = 8.0, 1.6 Hz, 1H), 8.16 (d, J = 7.8 Hz, 1H), 7.73 (t, J = 8.1 Hz, 1H), 7.00 (s, 1H), 2.70 (d, J = 17.5

Hz, 1H), 2.25 (d, *J* = 17.8 Hz, 1H), 2.10 (t, *J* = 4.2 Hz, 1H), 1.91 (s, 1H), 1.82 (d, *J* = 9.1 Hz, 1H), 1.40 (d, *J* = 9.0 Hz, 1H), 1.31 (d, *J* = 12.4 Hz, 1H), 1.04 (s, 3H), 0.96 (s, 3H), 0.79 (s, 3H). ^{13}C NMR (101 MHz, $CDCl_3$) δ 176.61 (s), 169.42 (s), 148.78 (s), 137.93 (s), 131.20 (d, *J* = 5.1 Hz), 129.01 (s), 124.62 (s), 120.65 (s), 103.52 (s), 53.79 (s), 48.66 (s), 44.08 (s), 36.30 (s), 32.56 (s), 26.91 (s), 19.54 (s), 18.58 (s), 10.80 (s). HRMS: *m*/*z*: $C_{19}H_{22}N_4O_2S$, calculated, 370.1463, found, 370.1538 $[M+H]^+$.

(E)-4-(4-nitrophenyl)-2-(2-(1,7,7-trimethylbicyclo[2.2.1]heptan-2-ylidene)hydrazinyl)thiazole (5i): A saffron acicular crystal, yield: 57.31%; m.p. 189 – 193°C; FT-IR *v* (cm^{-1}): 3379, 3297, 3109 (N-H), 3059 (Ar-H), 1625 (C=N), 1521, 1481 (Benzene ring), 1037 (C-S-C), 853 (p-disubstituted benzene ring); ^{1}H NMR (400 MHz, $CDCl_3$) δ 10.75 (s, 1H), 8.23 (d, *J* = 8.7 Hz, 2H), 8.05 (d, *J* = 8.6 Hz, 2H), 7.58 (s, 1H), 2.48 (s, 1H), 2.00 (d, *J* = 17.7 Hz, 1H), 1.93 (s, 1H), 1.78 (s, 1H), 1.68 (s, 1H), 1.30 (d, *J* = 9.0 Hz, 1H), 1.16 (d, *J* = 9.1 Hz, 1H), 0.93 (s, 3H), 0.86 (s, 3H), 0.68 (s, 3H). ^{13}C NMR (101 MHz, $CDCl_3$) δ 175.34 (s), 168.59 (s), 156.20 (s), 137.74 (s), 131.61 (s), 121.29 (s), 116.08 (s), 111.80 (s), 102.32 (s), 56.19 (s), 53.60 (s), 48.49 (s), 44.16 (s), 37.09 (s), 32.61 (s), 26.89 (s), 19.53 (s), 18.61 (s), 10.87 (s). HRMS: *m*/*z*: $C_{19}H_{22}N_4O_2S$, calculated, 370.1463, found, 370.1543 $[M+H]^+$.

(E)-4-(2-methoxyphenyl)-2-(2-(1,7,7-trimethylbicyclo[2.2.1]heptan-2-ylidene)hydrazinyl)thiazole (5j): A white acicular crystal, yield: 87.71%; m.p. 190 – 193°C; FT-IR *v* (cm^{-1}): 3450, 3296, 3160 (N-H), 3086, 3047 (Ar-H), 1610 (C=N), 1473 (Benzene ring), 1013 (C-S-C), 746 (o-disubstituted benzene ring); ^{1}H NMR (400 MHz, $CDCl_3$) δ 12.91 (s, 1H), 7.59 (d, *J* = 7.7 Hz, 1H), 7.39 (t, *J* = 7.8

Hz, 1H), 7.02 (t, *J* = 8.6 Hz, 2H), 6.80 (s, 1H), 4.07 (s, 3H), 2.74 (d, *J* = 17.8 Hz, 1H), 2.33 (d, *J* = 18.0 Hz, 1H), 2.06 (s, 1H), 1.89 – 1.81 (m, 1H), 1.80 – 1.72 (m, 1H), 1.39 (dd, *J* = 10.9, 7.5 Hz, 1H), 1.32 – 1.25 (m, 1H), 1.02 (s, 3H), 0.93 (s, 3H), 0.76 (s, 3H). ^{13}C NMR (101 MHz, $CDCl_3$) *δ* 175.34 (s), 168.59 (s), 156.20 (s), 137.74 (s), 131.61 (s), 128.09 (s), 121.29 (s), 116.08 (s), 111.80 (s), 102.32 (s), 56.19 (s), 53.60 (s), 48.49 (s), 44.16 (s), 37.09 (s), 32.61 (s), 26.89 (s), 19.53 (s), 18.61 (s), 10.87 (s). HRMS: *m*/*z*: $C_{20}H_{25}N_3OS$, calculated, 355.1718, found, 355.1797 $[M+H]^+$.

(E)-4-(4-methoxyphenyl)-2-(2-(1,7,7-trimethylbicyclo[2.2.1]heptan-2-ylidene)hydrazinyl)thiazole (5k): A white acicular crystal, yield: 85.23%; m.p. 154 – 158°C; FT-IR *v* (cm^{-1}): 3425, 3215, 3142 (N-H), 3072 (Ar-H), 1620 (C=N), 1513 (Benzene ring), 1027 (C-S-C), 835 (p-disubstituted benzene ring); ^{1}H NMR (400 MHz, $CDCl_3$) *δ* 12.00 (s, 1H), 7.66 – 7.61 (m, 2H), 6.96 (d, *J* = 8.9 Hz, 2H), 6.57 (d, *J* = 2.9 Hz, 1H), 3.82 (d, *J* = 2.8 Hz, 3H), 2.74 – 2.65 (m, 1H), 2.25 (d, *J* = 17.8 Hz, 1H), 2.07 (s, 1H), 1.89 (s, 1H), 1.79 (s, 1H), 1.40 (s, 1H), 1.29 (s, 1H), 1.03 (s, 3H), 0.95 (s, 3H), 0.77 (s, 3H). ^{13}C NMR (101 MHz, $CDCl_3$) *δ* 176.98 (s), 170.69 (s), 162.64 (s), 141.72 (s), 128.69 (s), 128.43 (s), 121.62 (s), 116.43 (d, *J* = 12.6 Hz), 100.01 (s), 57.05 (s), 55.17 (s), 50.13 (s), 45.65 (s), 37.90 (s), 34.14 (s), 28.49 (s), 21.10 (s), 20.16 (s), 12.41 (s). HRMS: *m*/*z*: $C_{20}H_{25}N_3OS$, calculated, 355.1718, found, 355.1784 $[M+H]^+$.

(E)-4-(4-methylphenyl)-2-(2-(1,7,7-trimethylbicyclo[2.2.1]heptan-2-ylidene)hydrazinyl)thiazole (5l): A white acicular crystal, yield: 87.75%; m.p. 170 – 175°C; FT-IR *v* (cm^{-1}): 3379, 3231 (N-H), 3043 (Ar-H), 1620 (C=N), 1488,

1445 (Benzene ring), 1035 (C-S-C), 814 (p-disubstituted benzene ring); ^{1}H NMR (400 MHz, $CDCl_3$) *δ* 12.03 (s, 1H), 7.55 (d, *J* = 8.1 Hz, 2H), 7.21 (d, *J* = 8.1 Hz, 2H), 6.69 (s, 1H), 2.67 (d, *J* = 17.2 Hz, 1H), 2.33 (s, 3H), 2.23 (d, *J* = 17.8 Hz, 1H), 2.04 (t, *J* = 4.2 Hz, 1H), 1.86 (s, 1H), 1.77 (s, 1H), 1.37 (s, 1H), 1.26 (s, 1H), 1.00 (s, 3H), 0.92 (s, 3H), 0.75 (s, 3H). ^{13}C NMR (101 MHz, $CDCl_3$) *δ* 177.01 (s), 170.71 (s), 142.15 (s), 141.79 (s), 131.64 (d, *J* = 16.2 Hz), 127.02 (s), 126.72 (s), 126.17 (s), 101.44 (s), 55.15 (s), 50.11 (s), 45.63 (s), 37.95 (s), 34.12 (s), 28.47 (s), 22.96 (s), 21.09 (s), 20.15 (s), 12.39 (s). HRMS: m/z: $C_{20}H_{25}N_3S$, calculated, 339.1769, found, 339.1847 $[M+H]^+$.

(E)-4-(2,4-difluorophenyl)-2-(2-(1,7,7-trimethylbicyclo[2.2.1]heptan-2-ylidene)hydrazinyl)thiazole (5m): A brown acicular crystal, yield: 77.21%; m.p. 160 – 164°C; FT-IR *v* (cm^{-1}): 3450, 3234 (N-H), 3030 (Ar-H), 1620 (C=N), 1495 (Benzene ring), 1073 (C-S-C), 858, 788 (1,2,4-trisubstituted benzene ring); ^{1}H NMR (400 MHz, $CDCl_3$) *δ* 12.02 (s, 1H), 7.87 (d, *J* = 5.9 Hz, 1H), 7.03 (s, 1H), 6.96 (d, *J* = 8.2 Hz, 2H), 2.68 (d, *J* = 17.7 Hz, 1H), 2.23 (d, *J* = 17.8 Hz, 1H), 2.07 (s, 1H), 1.88 (s, 1H), 1.78 (s, 1H), 1.39 (s, 1H), 1.27 (s, 1H), 1.02 (s, 3H), 0.94 (s, 3H), 0.76 (s, 3H). ^{13}C NMR (101 MHz, $CDCl_3$) *δ* 177.48 (s), 170.33 (s), 134.90 (s), 130.72 (s), 114.52 (d, *J* = 21.8 Hz), 114.11 (d, *J* = 7.3 Hz), 107.13 (s), 106.38 (s), 55.23 (s), 50.14 (s), 45.67 (s), 37.90 (s), 34.13 (s), 28.47 (s), 21.07 (s), 20.13 (s), 12.34 (s). HRMS: *m/z*: $C_{19}H_{21}F_2N_3S$, calculated, 361.1424, found, 362.1498 $[M+H]^+$.

(E)-4-(3,4-dichlorophenyl)-2-(2-(1,7,7-trimethylbicyclo[2.2.1]heptan-2-ylidene)hydrazinyl)thiazole (5n): A white acicular crystal, yield: 90.20%; m.p.

198 – 203°C; FT-IR *v* (cm^{-1}): 3450, 3219 (N-H), 3067, 3038 (Ar-H), 1618 (C=N), 1489 (Benzene ring), 1041 (C-S-C), 823, 744(1,3,4-trisubstituted benzene ring); ^{1}H NMR (400 MHz, $CDCl_3$) δ 12.03 (s, 1H), 7.76 (s, 1H), 7.59 (s, 1H), 7.55 (s, 1H), 6.78 (s, 1H), 2.65 (s, 1H), 2.23 (d, *J* = 17.8 Hz, 1H), 2.07 (s, 1H), 1.89 (s, 1H), 1.79 (s, 1H), 1.39 (s, 1H), 1.28 (s, 1H), 1.02 (s, 3H), 0.94 (s, 3H), 0.77 (s, 3H). ^{13}C NMR (101 MHz, $CDCl_3$) δ 176.35 (s), 169.28 (s), 138.02 (s), 134.69 (s), 133.98 (s), 131.69 (s), 127.38 (s), 127.18 (s), 124.74 (s), 102.22 (s), 53.72 (s), 48.62 (s), 44.07 (s), 36.33 (s), 32.54 (s), 26.89 (s), 19.52 (s), 18.56 (s), 10.78 (s). HRMS: *m*/*z*: $C_{19}H_{21}Cl_2N_3S$, calculated, 393.0833, found, 393.0917 $[M+H]^+$.

(E)-4-(4-(trifluoromethyl)phenyl)-2-(2-(1,7,7-trimethylbicyclo[2.2.1]heptan-2-ylidene)hydrazinyl)thiazole (5o): A white acicular crystal, yield: 90.09%; m.p. 170 – 174°C; FT-IR *v* (cm^{-1}): 3318, 3113 (N-H), 3042 (Ar-H), 1618 (C=N), 1584, 1511, 1483 (Benzene ring), 1069 (C-S-C), 842 (p-disubstituted benzene ring); ^{1}H NMR (400 MHz, $CDCl_3$) δ 11.98 (s, 1H), 7.83 (s, 2H), 7.70 (s, 2H), 6.96 (s, 1H), 2.65 (s, 1H), 2.25 (s, 1H), 2.06 (s, 1H), 1.88 (s, 1H), 1.79 (s, 1H), 1.39 (s, 1H), 1.27 (s, 1H), 1.02 (s, 3H), 0.94 (s, 3H), 0.76 (s, 3H). ^{13}C NMR (101 MHz, $CDCl_3$) δ 177.74 (s), 170.91 (s), 140.31 (s), 133.67 (s), 133.34 (s), 132.22 (s), 128.12 (d, *J* = 3.4 Hz), 127.56 (s), 123.73 (s), 104.87 (s), 55.27 (s), 50.17 (s), 45.61 (s), 37.84 (s), 34.08 (s), 28.46 (s), 21.06 (s), 20.11 (s), 12.33 (s). HRMS: *m*/*z*: $C_{20}H_{22}F_3N_3S$, calculated, 393.1478, found, 393.1566 $[M+H]^+$.

Spectrum of the title compounds

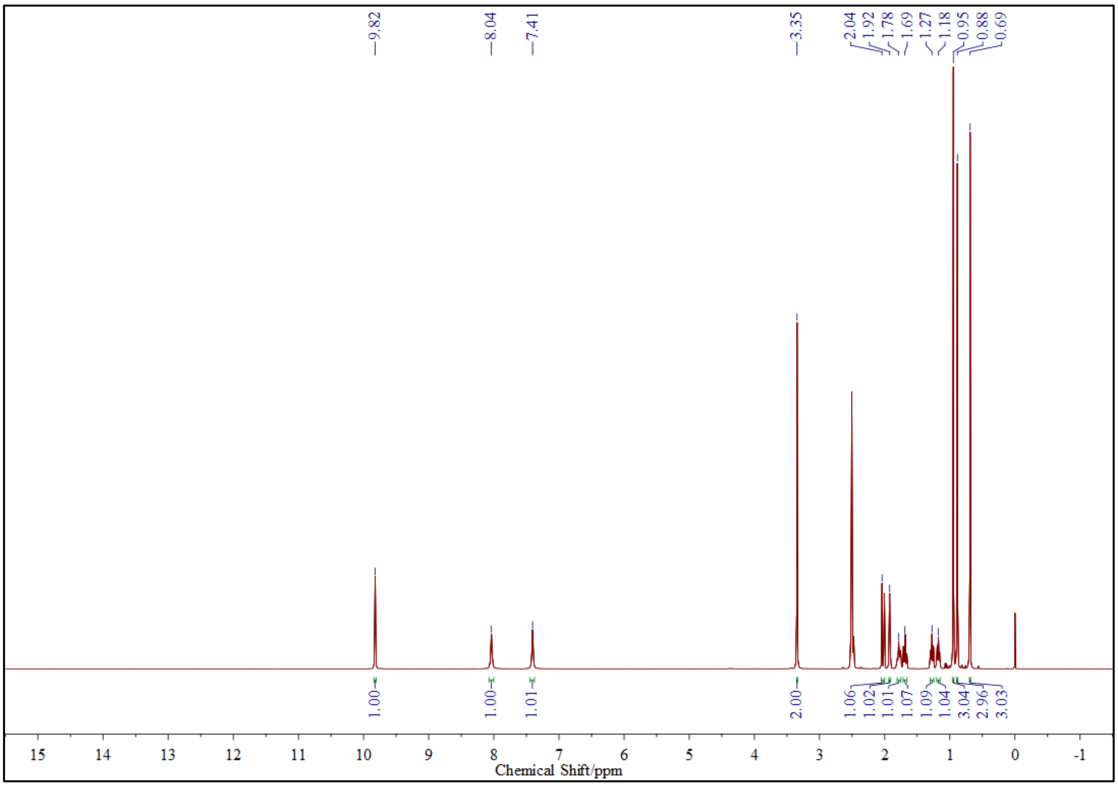

Figure S1. ¹H-NMR spectrum of compound 3a.

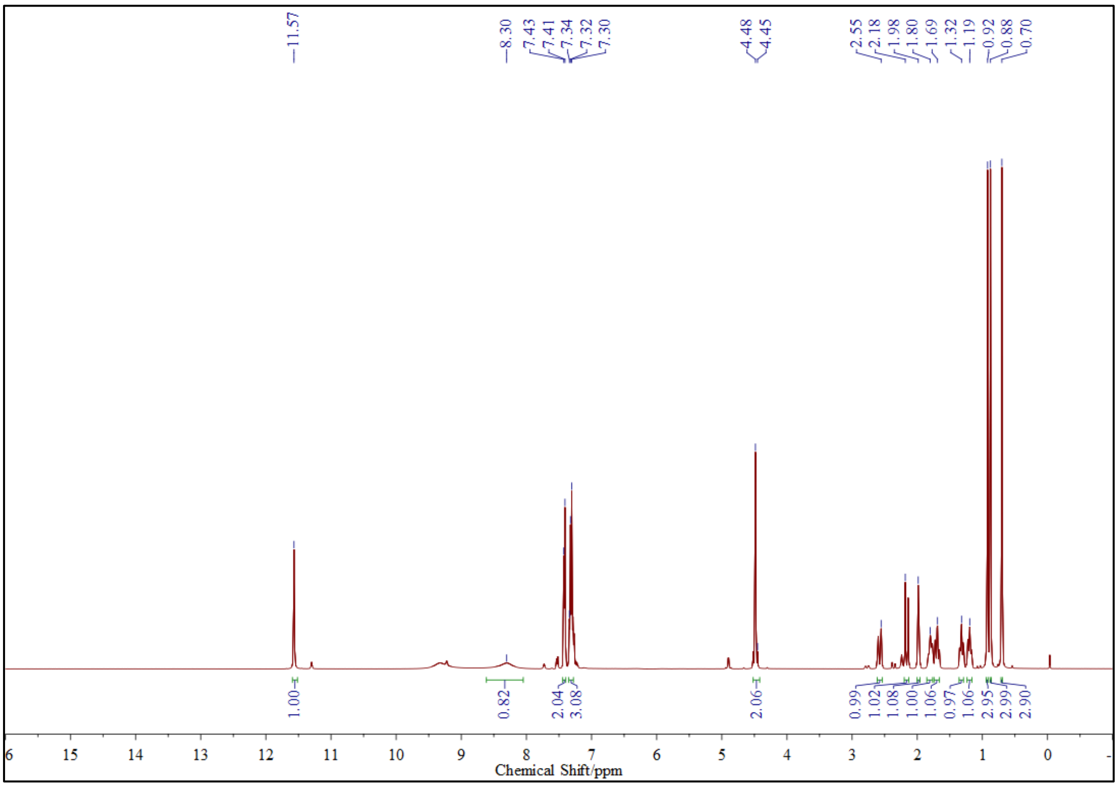

Figure S2. ¹H-NMR spectrum of compound 4a.

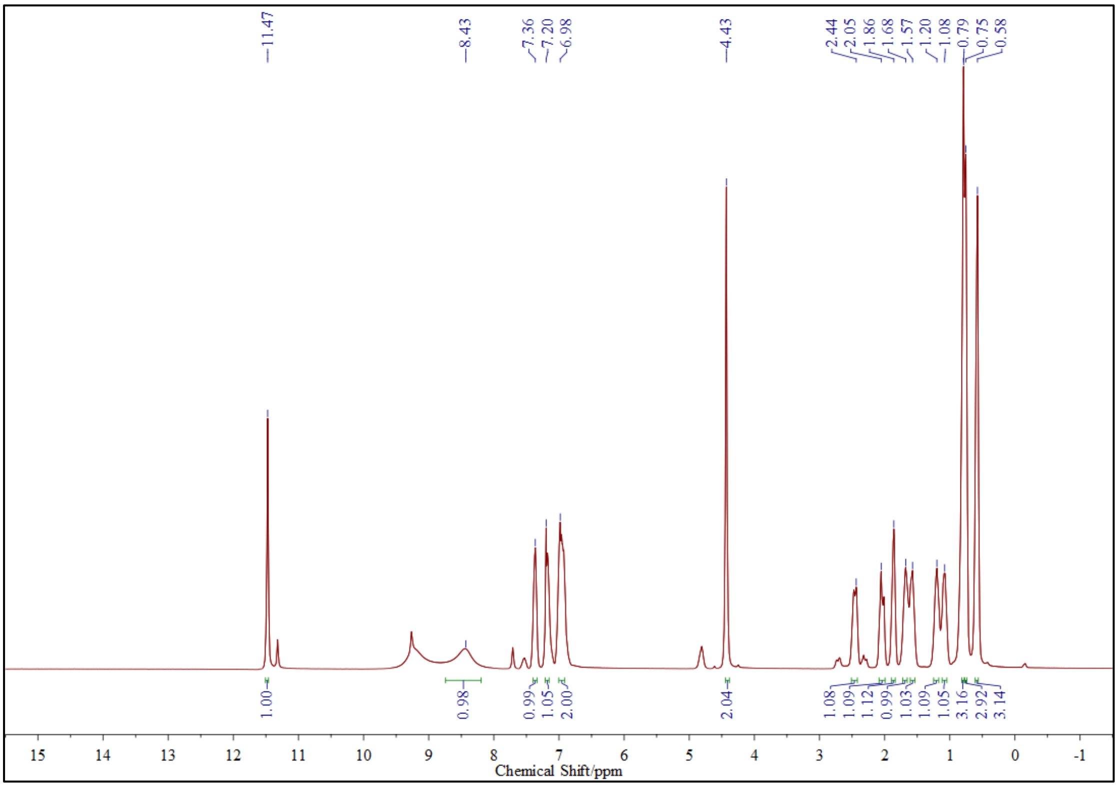

Figure S3. ¹H-NMR spectrum of compound 4b.

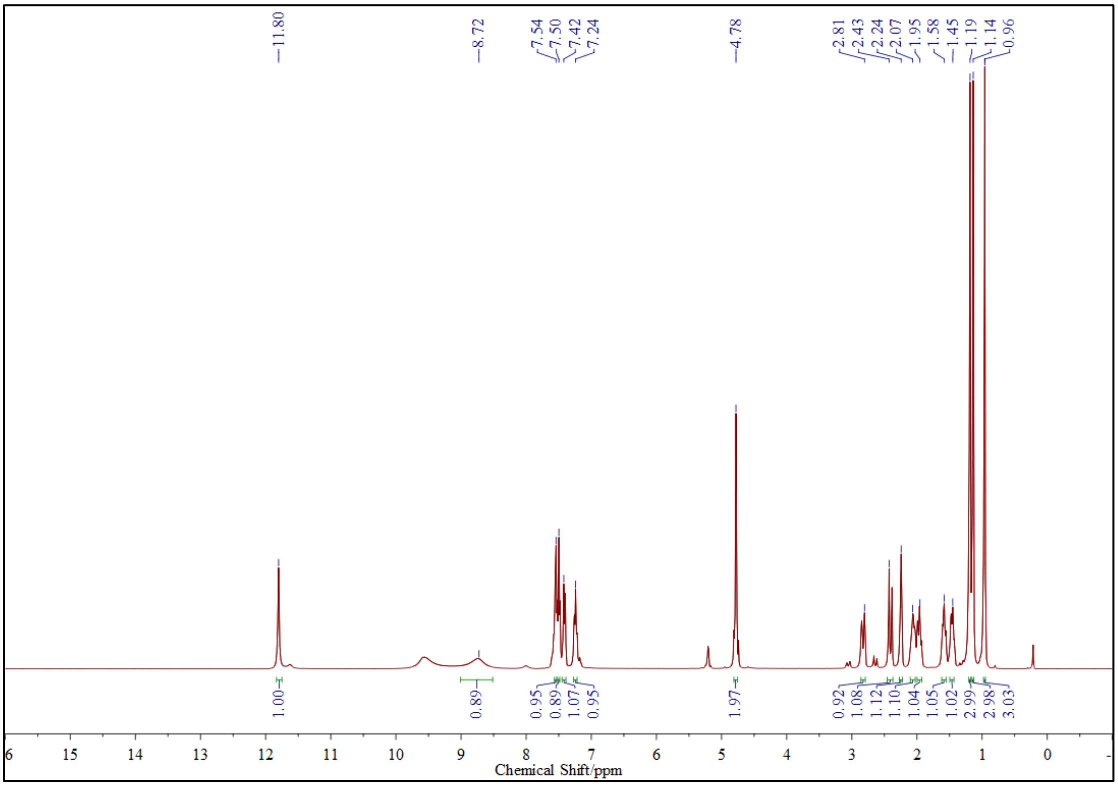

Figure S4. ¹H-NMR spectrum of compound 4c.

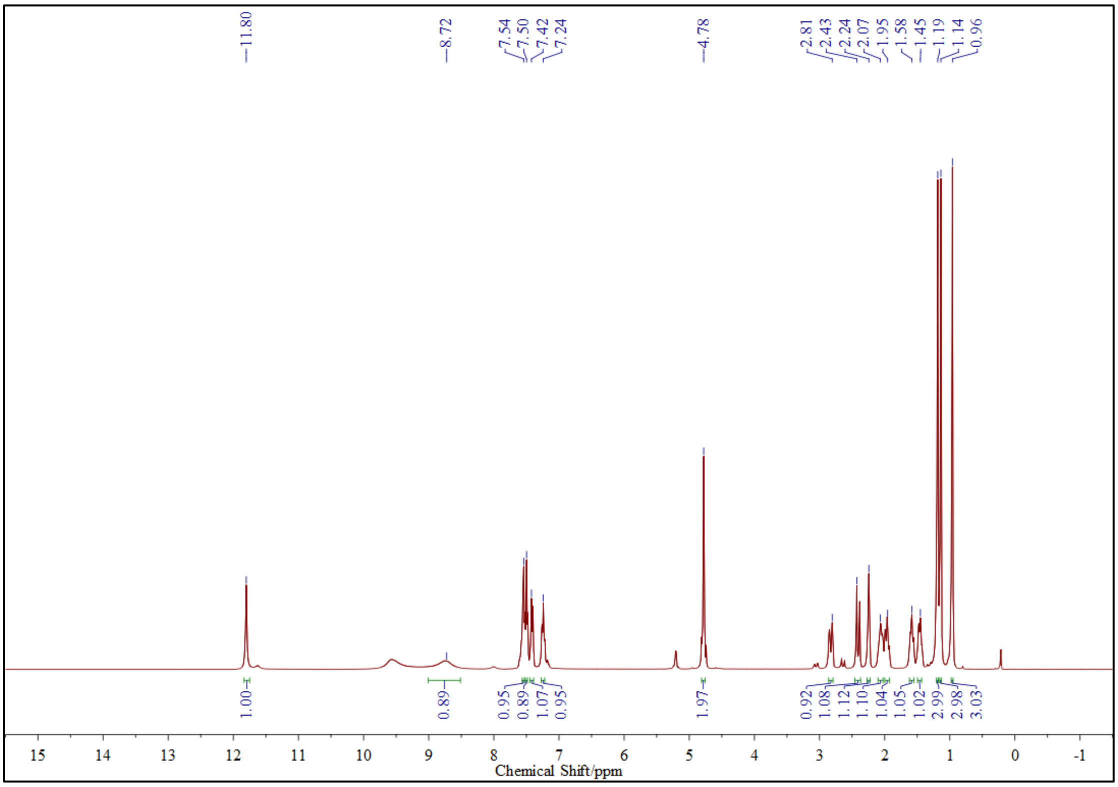

Figure S5. ^{1}H-NMR spectrum of compound 4d.

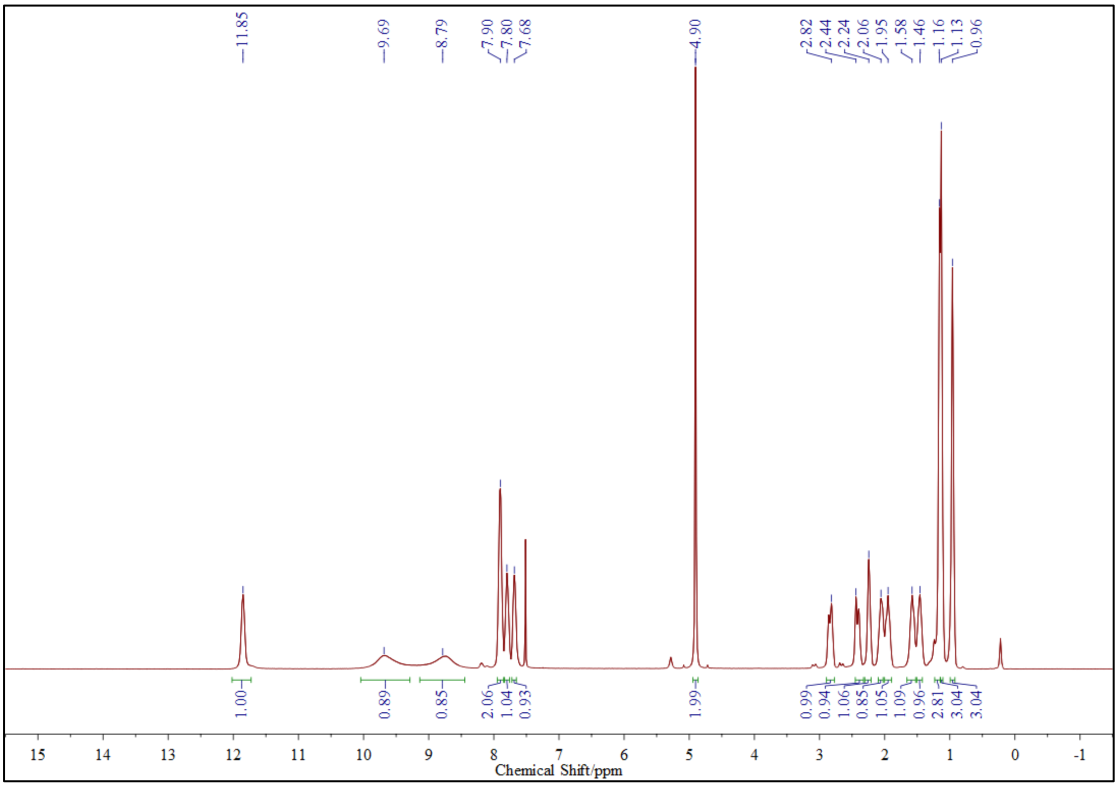

Figure S6. ^{1}H-NMR spectrum of compound 4e.

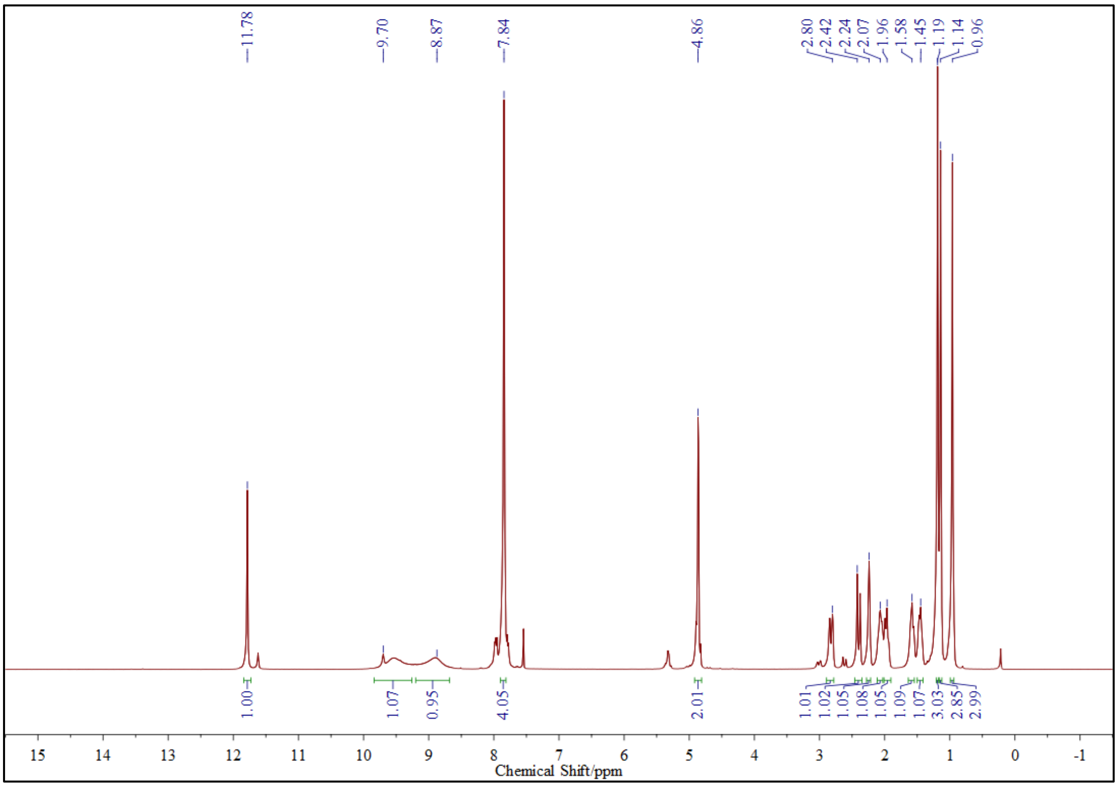

Figure S7. ¹H-NMR spectrum of compound 4f.

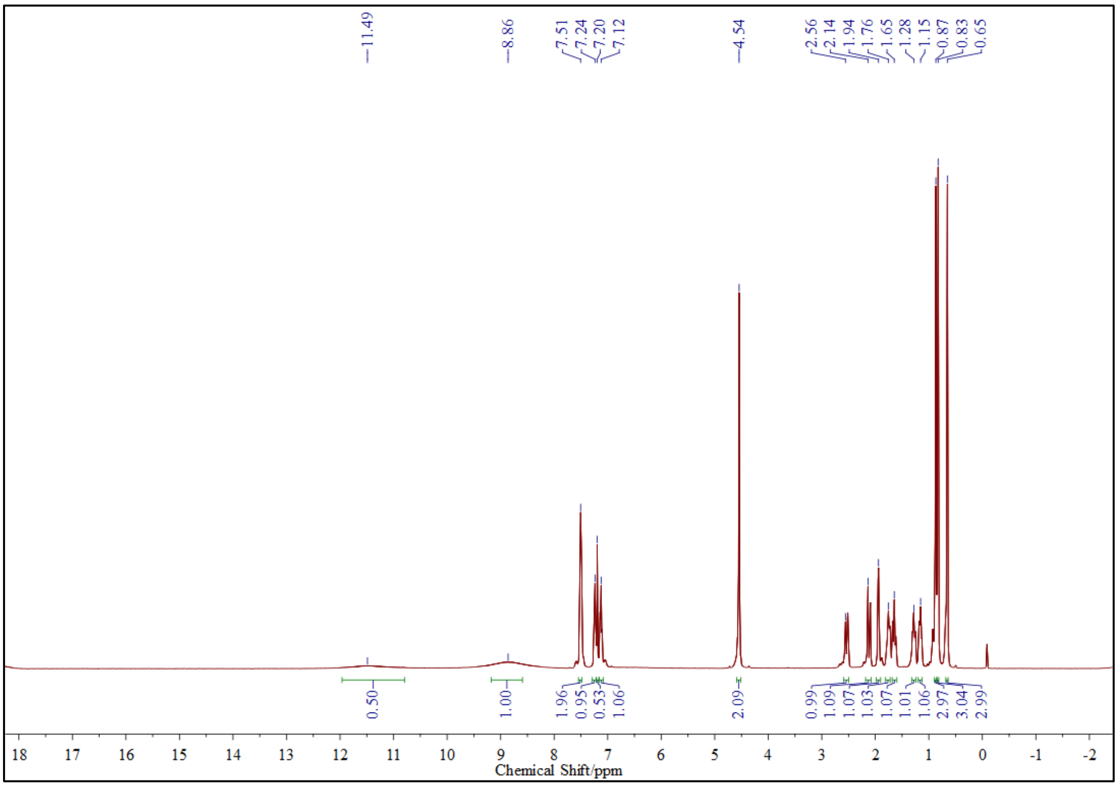

Figure S8. ¹H-NMR spectrum of compound 4g.

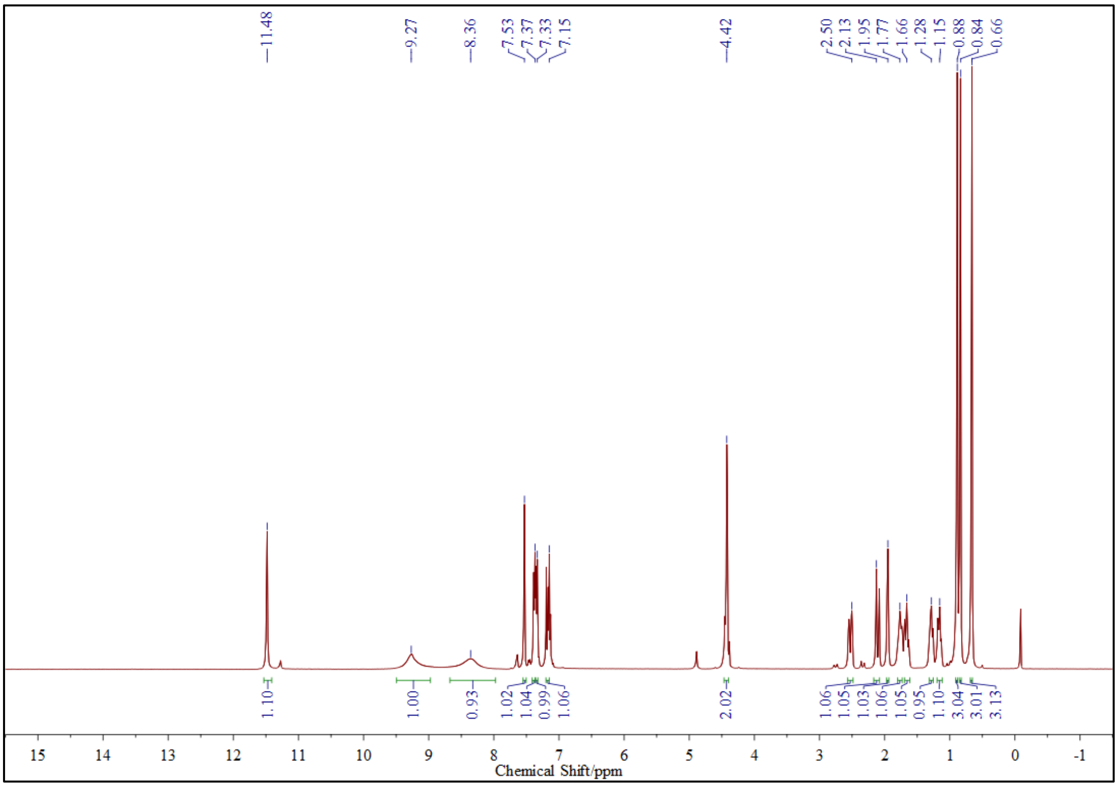

Figure S9. ^{1}H-NMR spectrum of compound 4h.

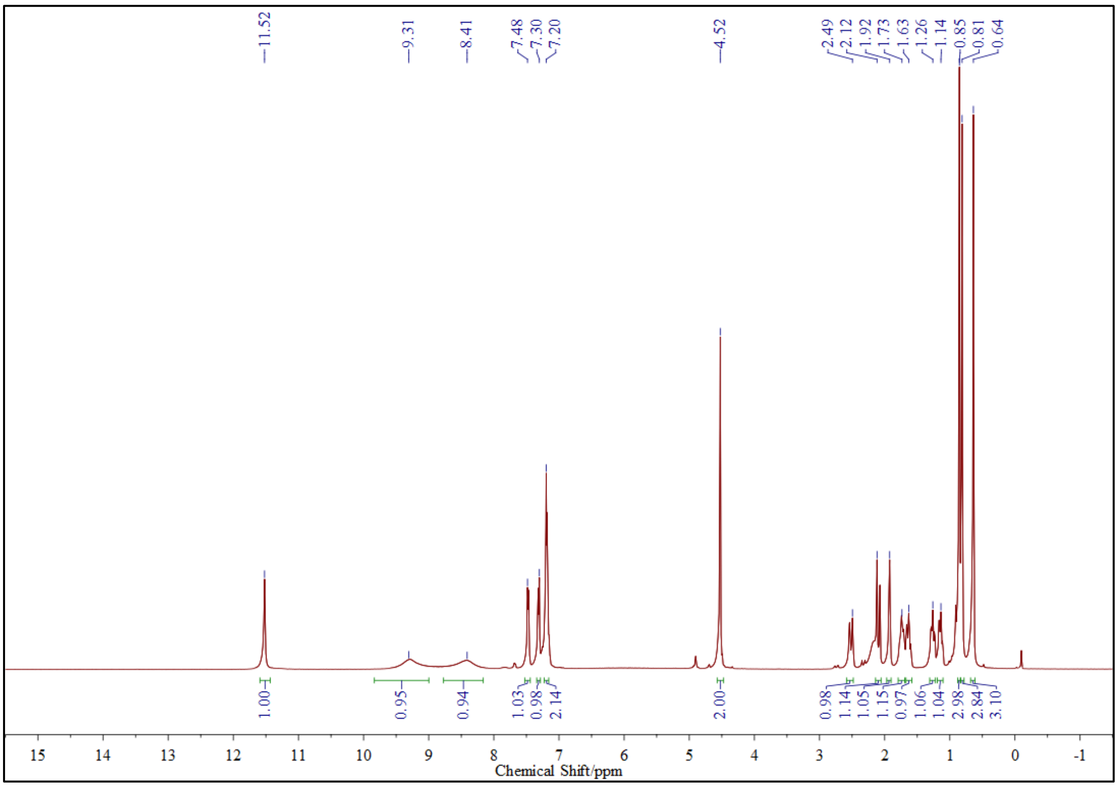

Figure S10. ^{1}H-NMR spectrum of compound 4i.

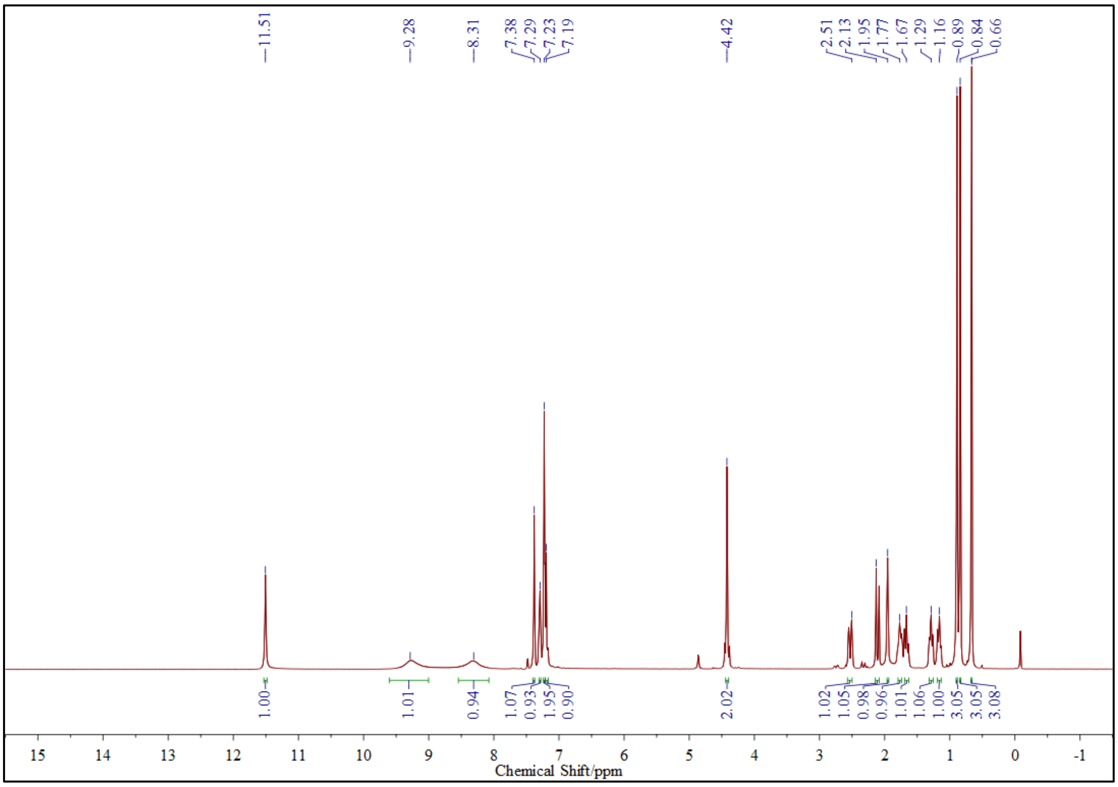

Figure S11. ^{1}H-NMR spectrum of compound 4j.

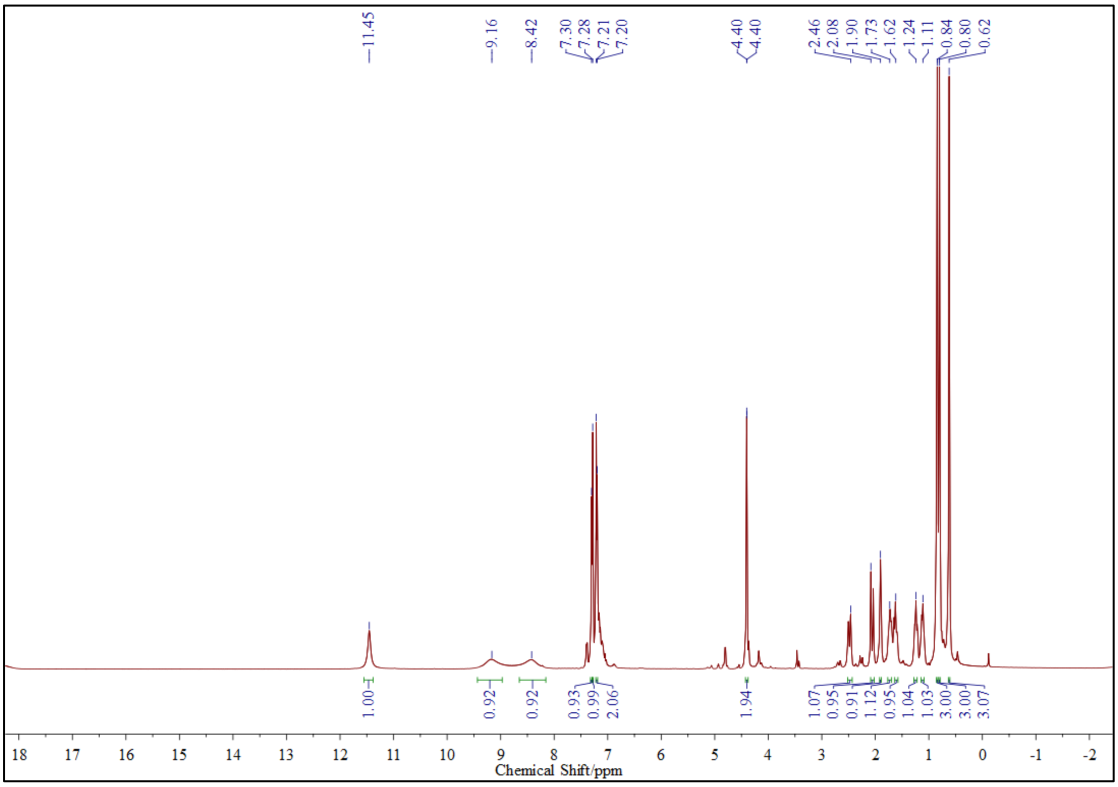

Figure S12. ^{1}H-NMR spectrum of compound 4k.

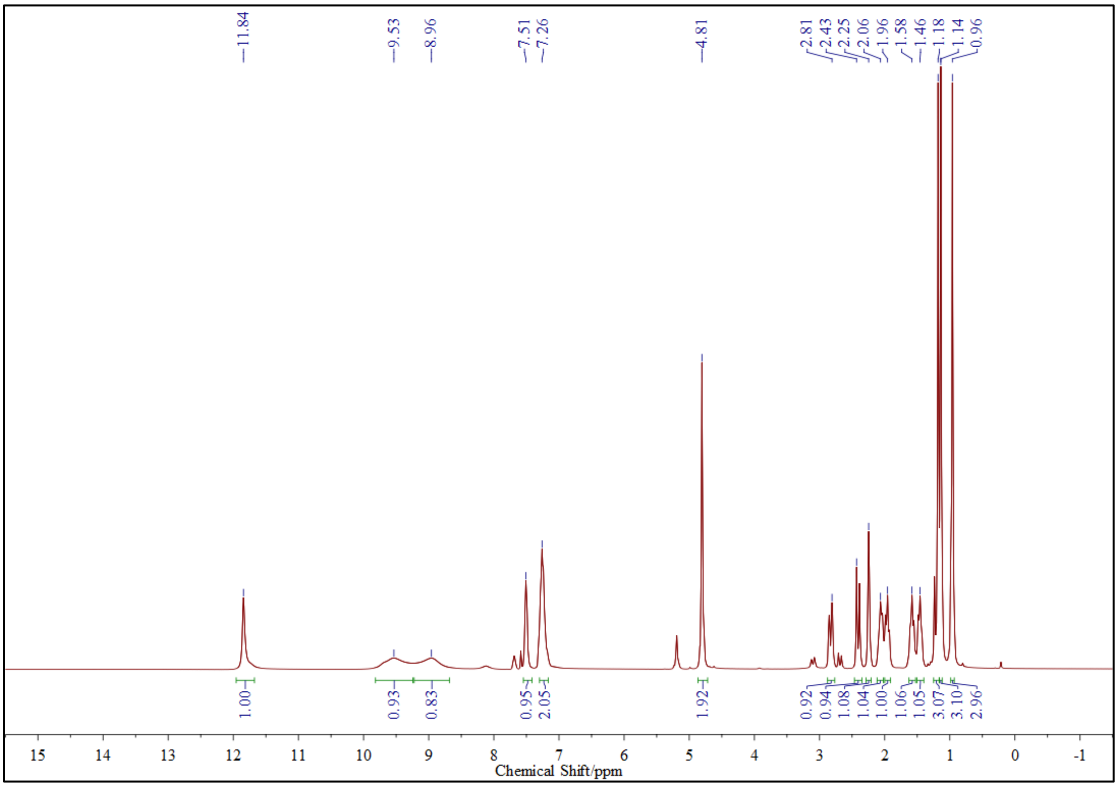

Figure S13. ¹H-NMR spectrum of compound 4l.

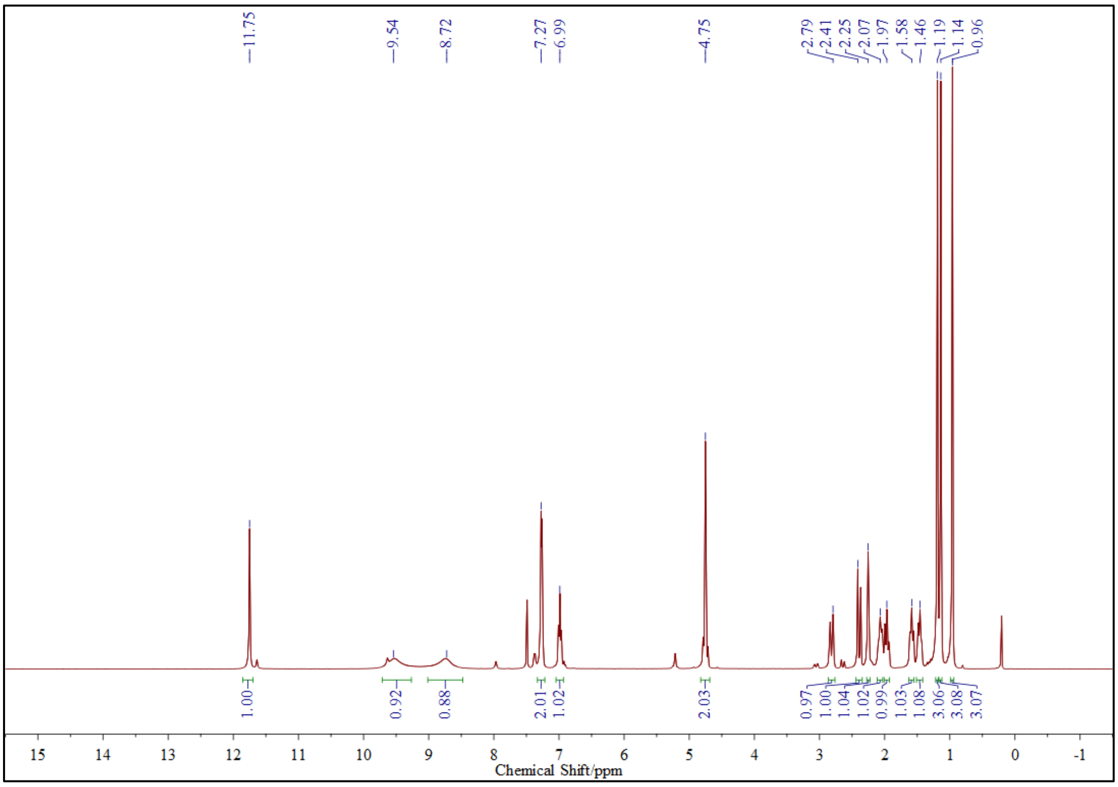

Figure S14. ¹H-NMR spectrum of compound 4m.

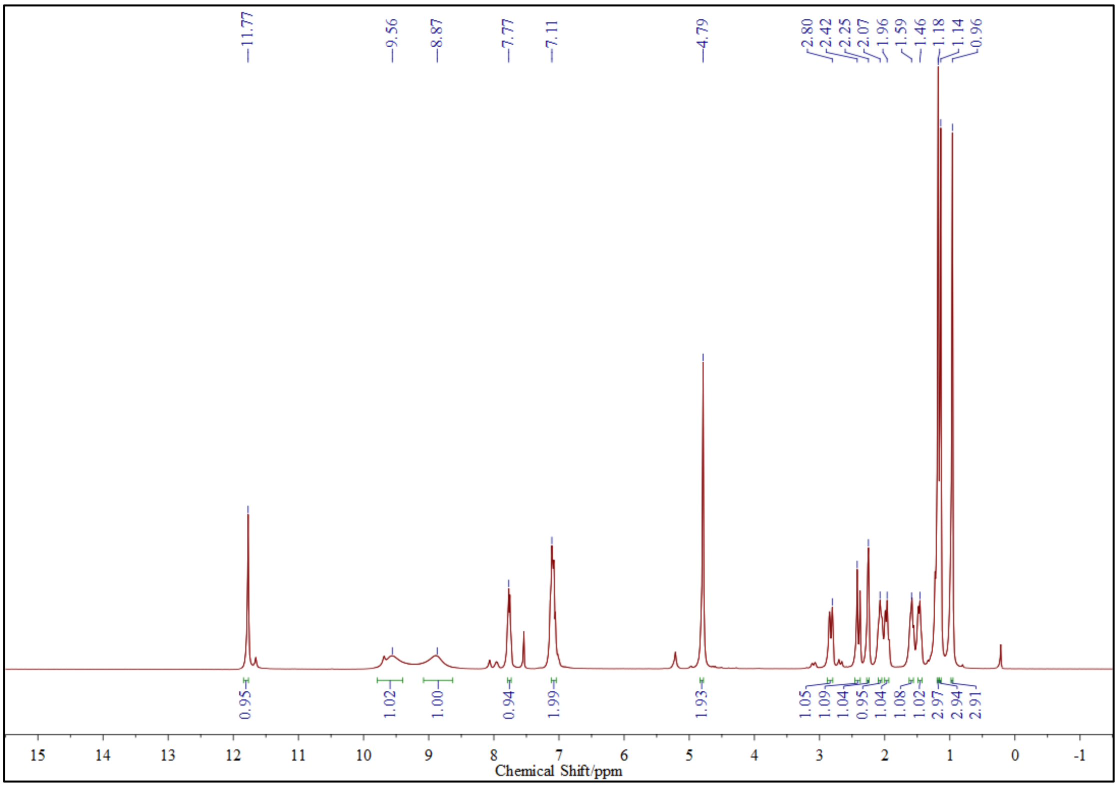

Figure S15. ¹H-NMR spectrum of compound 4n.

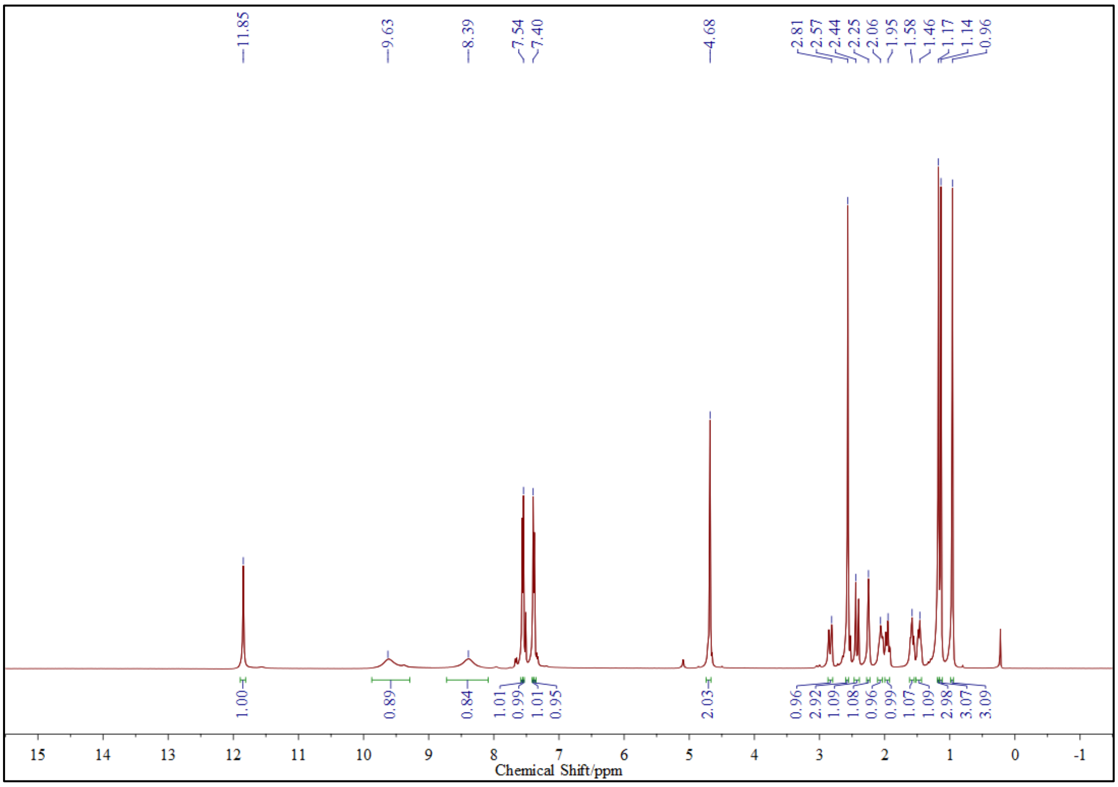

Figure S16. ¹H-NMR spectrum of compound 4o.

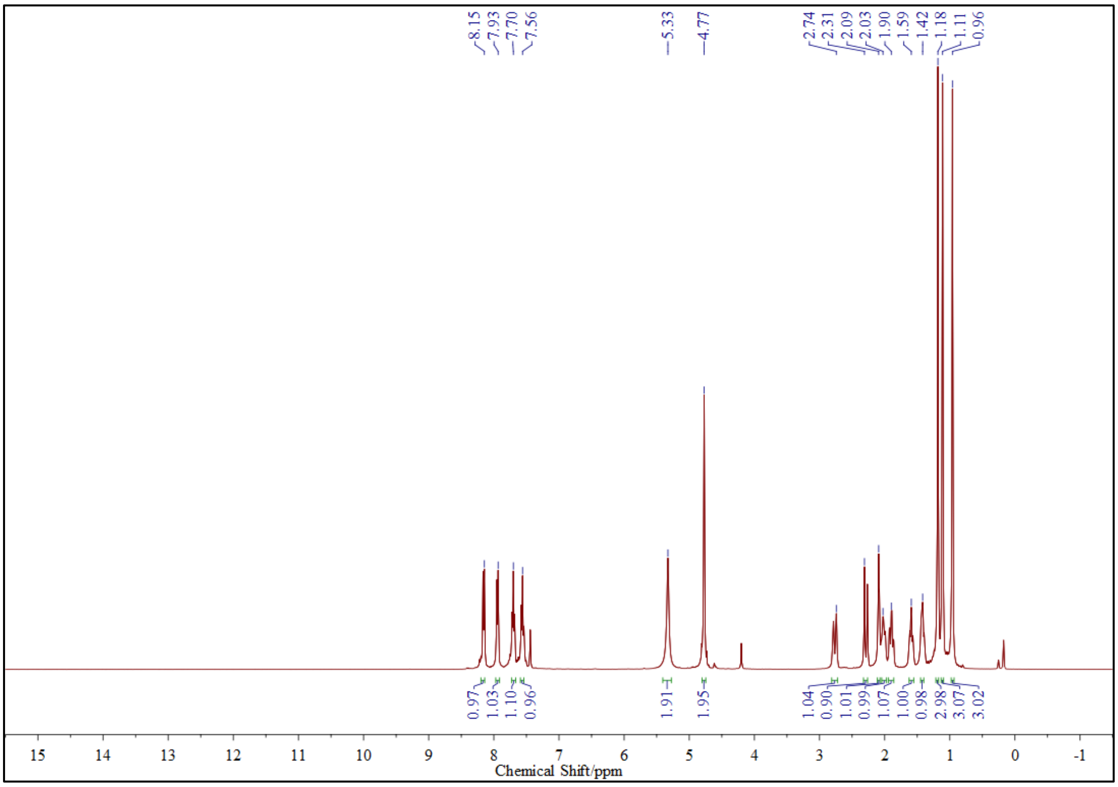

Figure S17. ¹H-NMR spectrum of compound 4p.

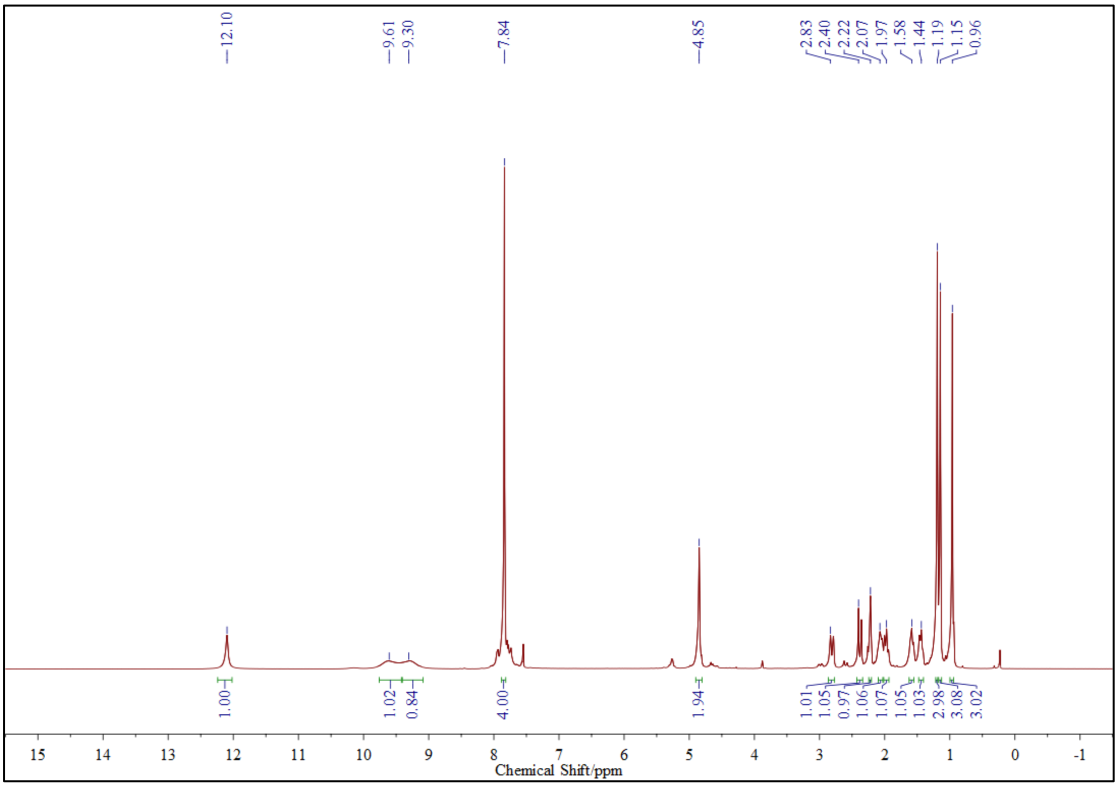

Figure S18. ¹H-NMR spectrum of compound 4q.

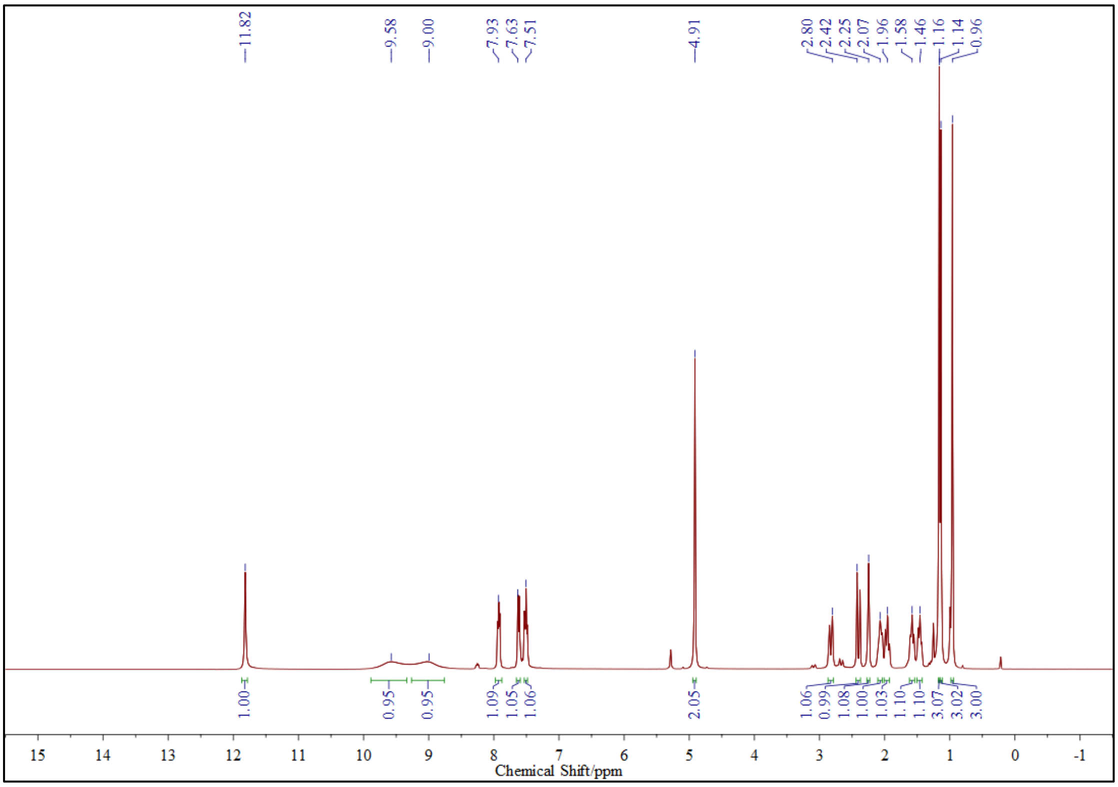

Figure S19. ^{1}H-NMR spectrum of compound 4r.

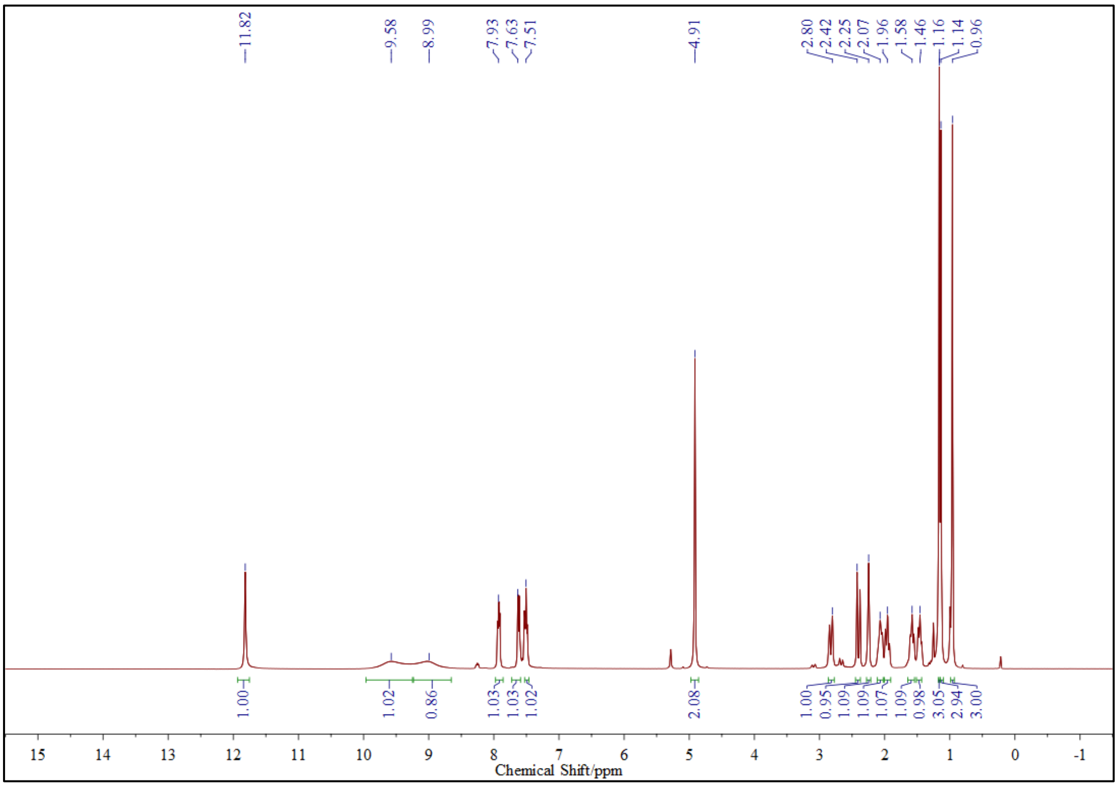

Figure S20. ^{1}H-NMR spectrum of compound 4s.

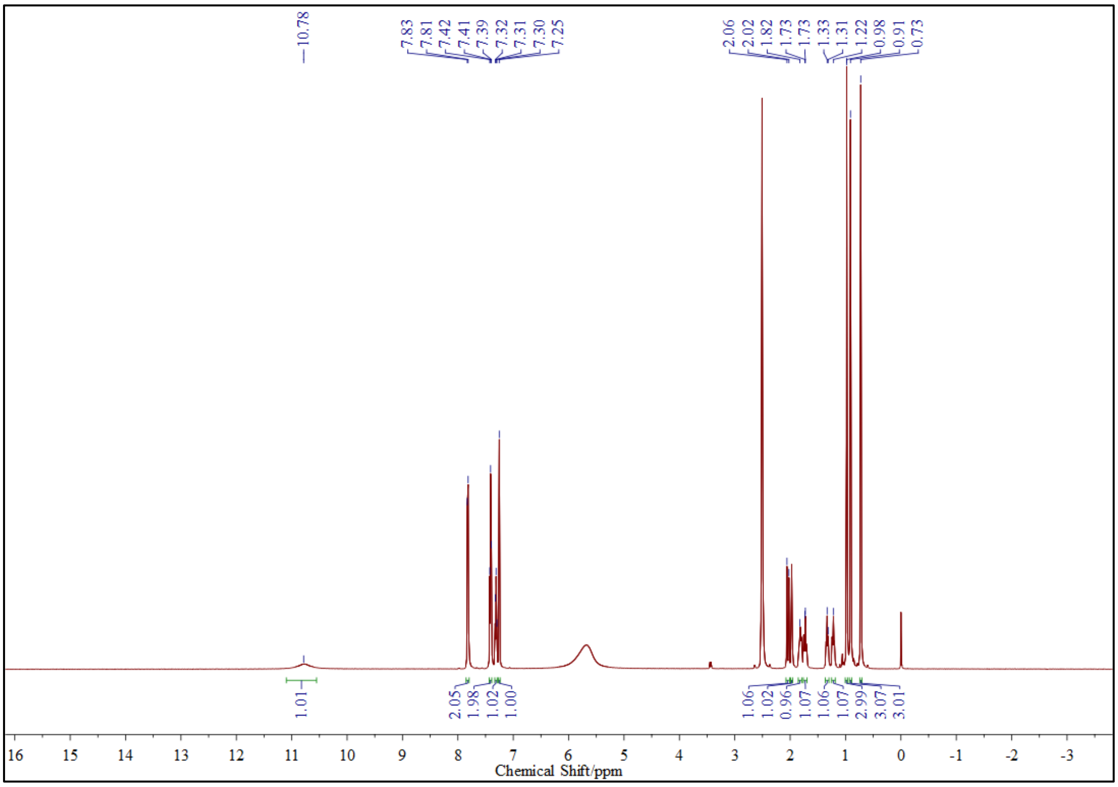

Figure S21. ^{1}H-NMR spectrum of compound 5a.

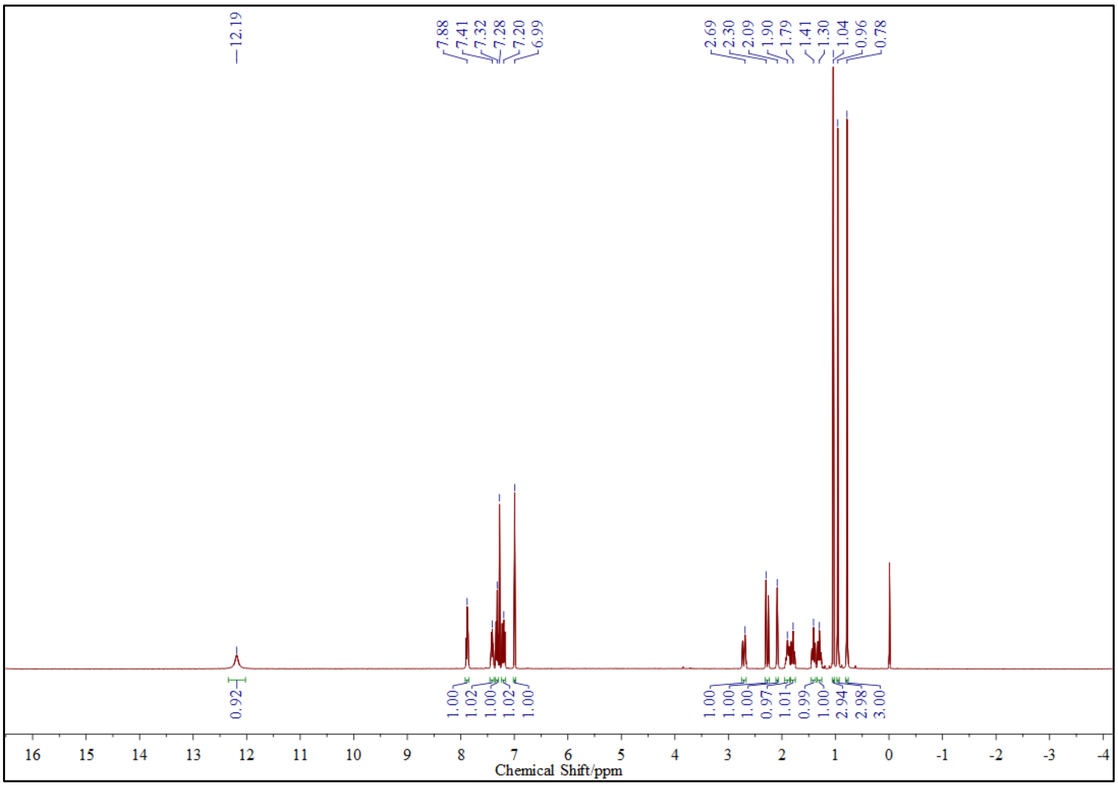

Figure S22. ^{1}H-NMR spectrum of compound 5b.

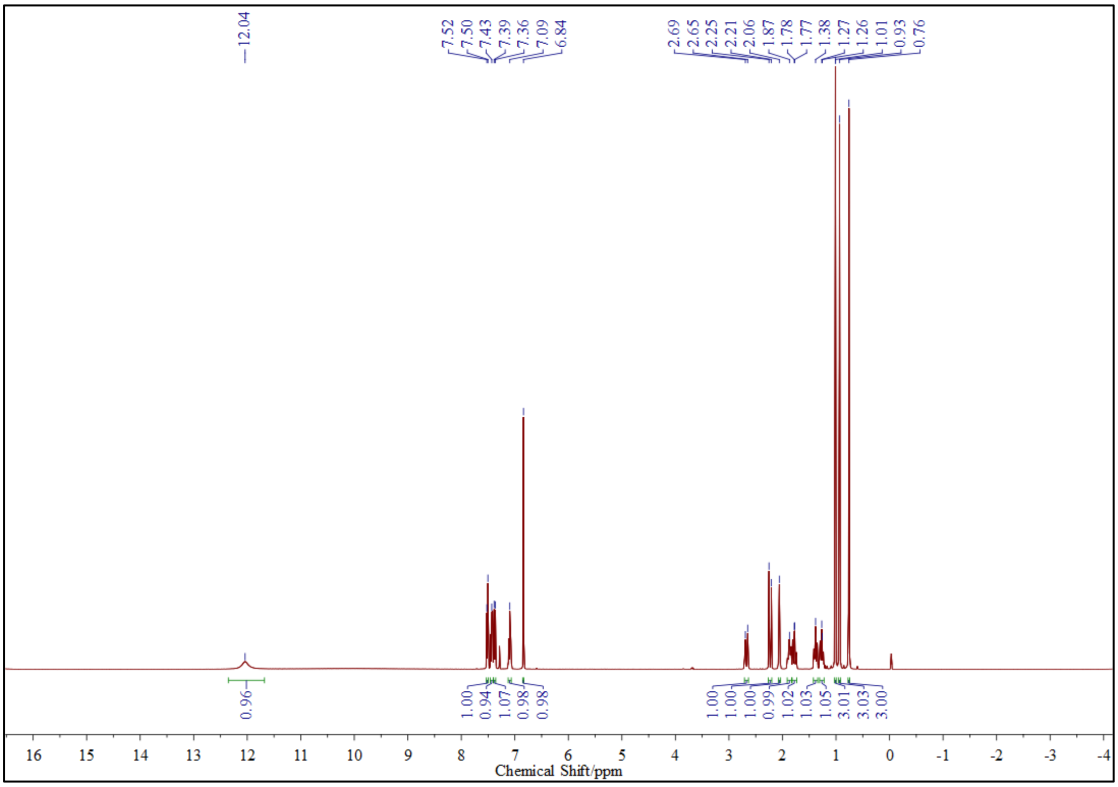

Figure S23. ¹H-NMR spectrum of compound 5c.

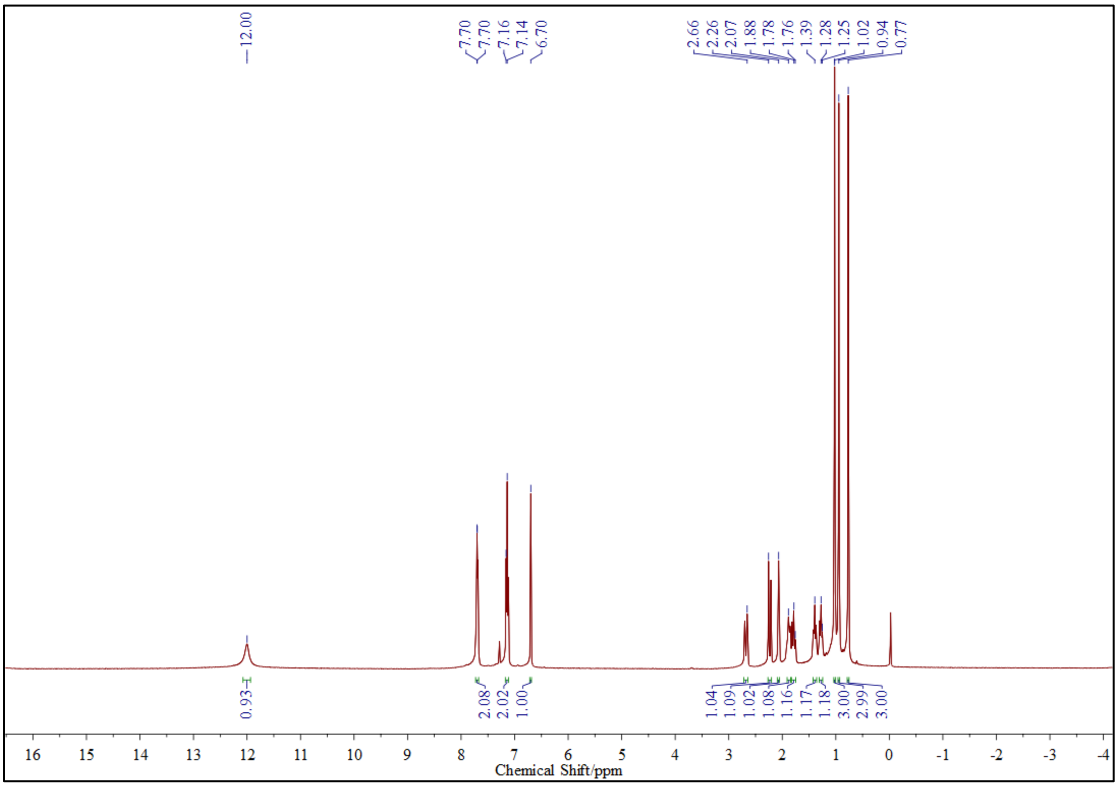

Figure S24. ¹H-NMR spectrum of compound 5d.

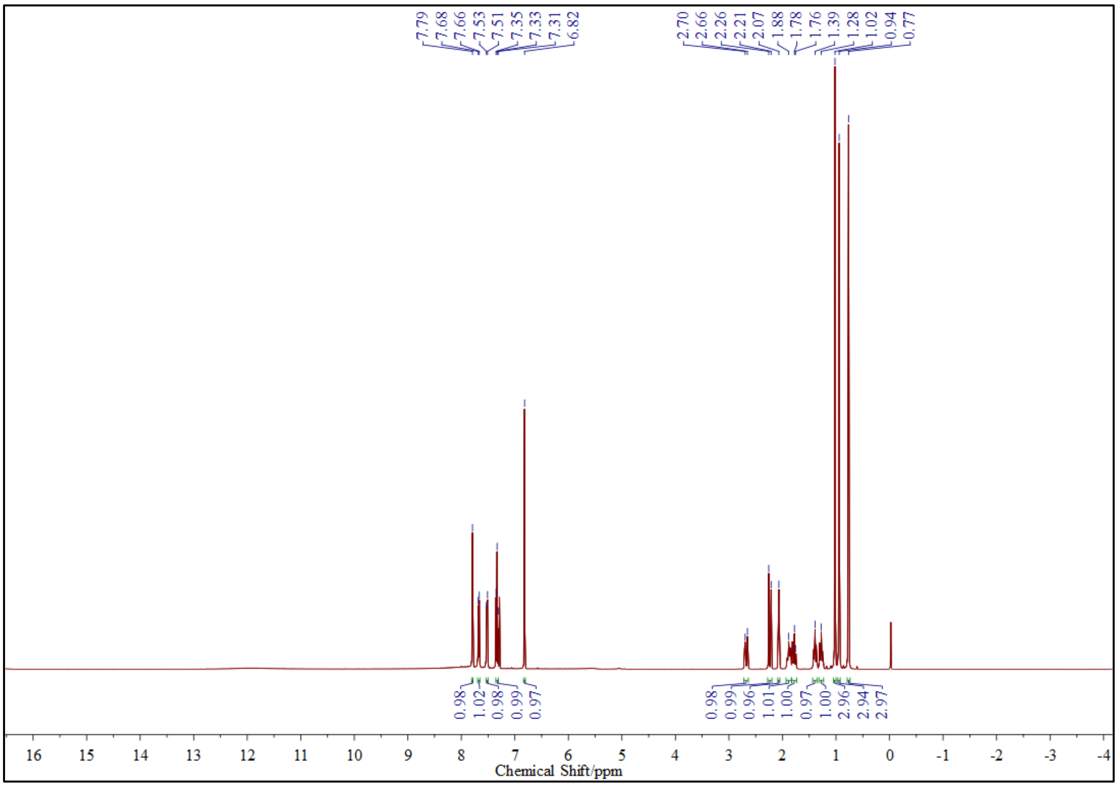

Figure S25. ¹H-NMR spectrum of compound 5e.

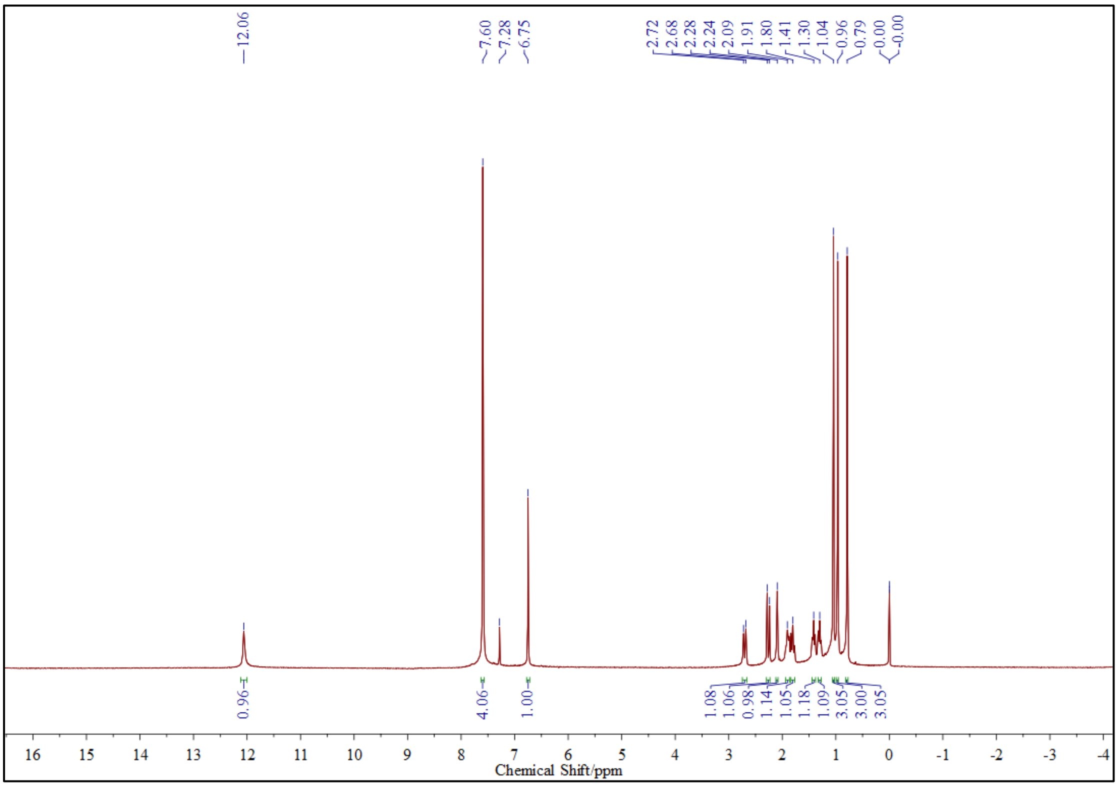

Figure S26. ¹H-NMR spectrum of compound 5f.

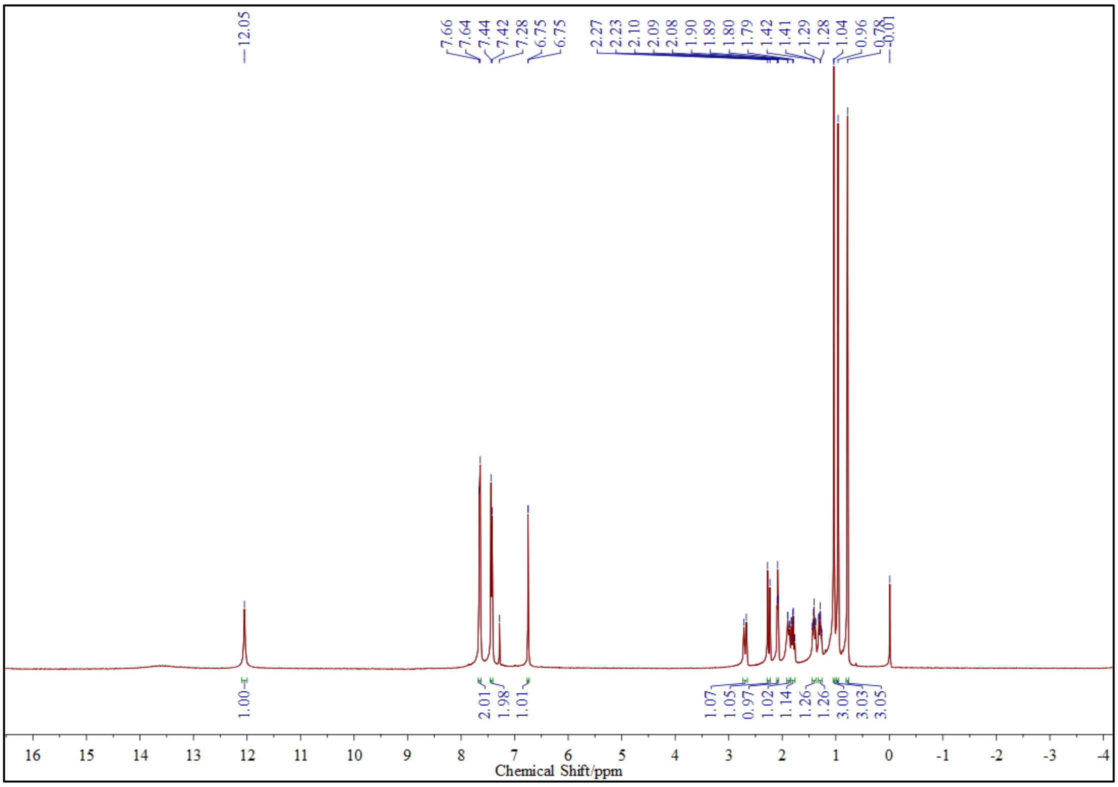

Figure S27. ¹H-NMR spectrum of compound 5g.

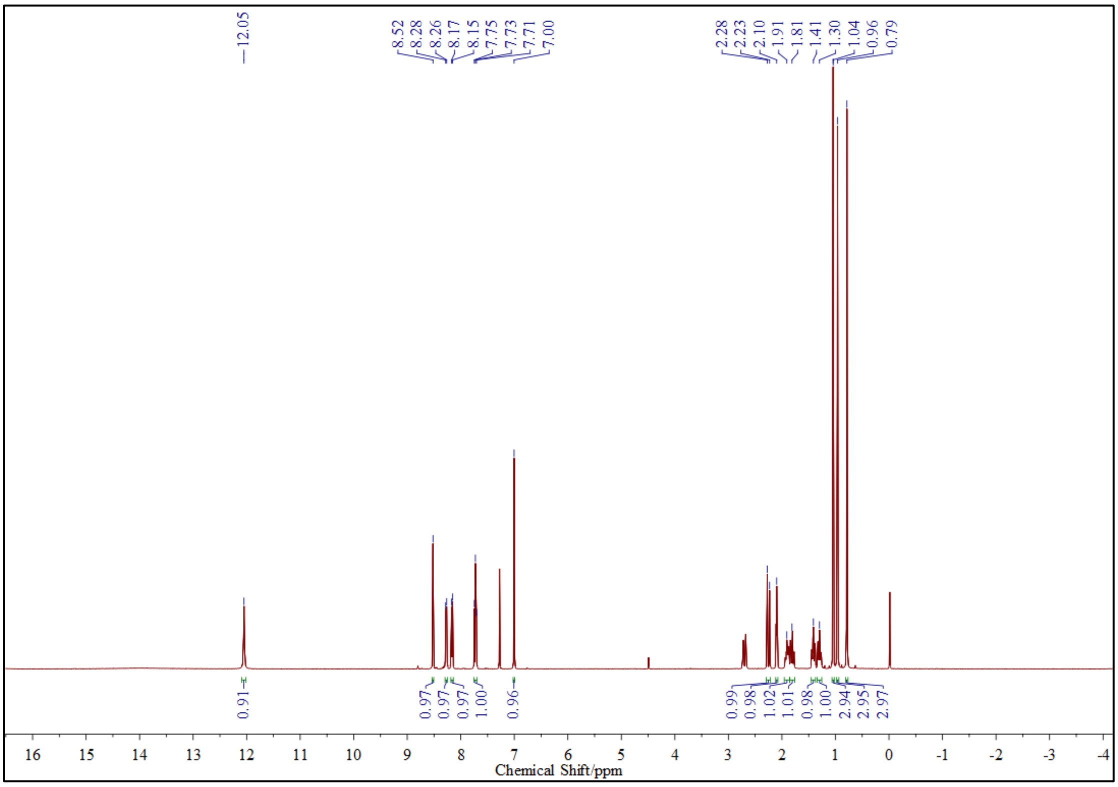

Figure S28. ¹H-NMR spectrum of compound 5h.

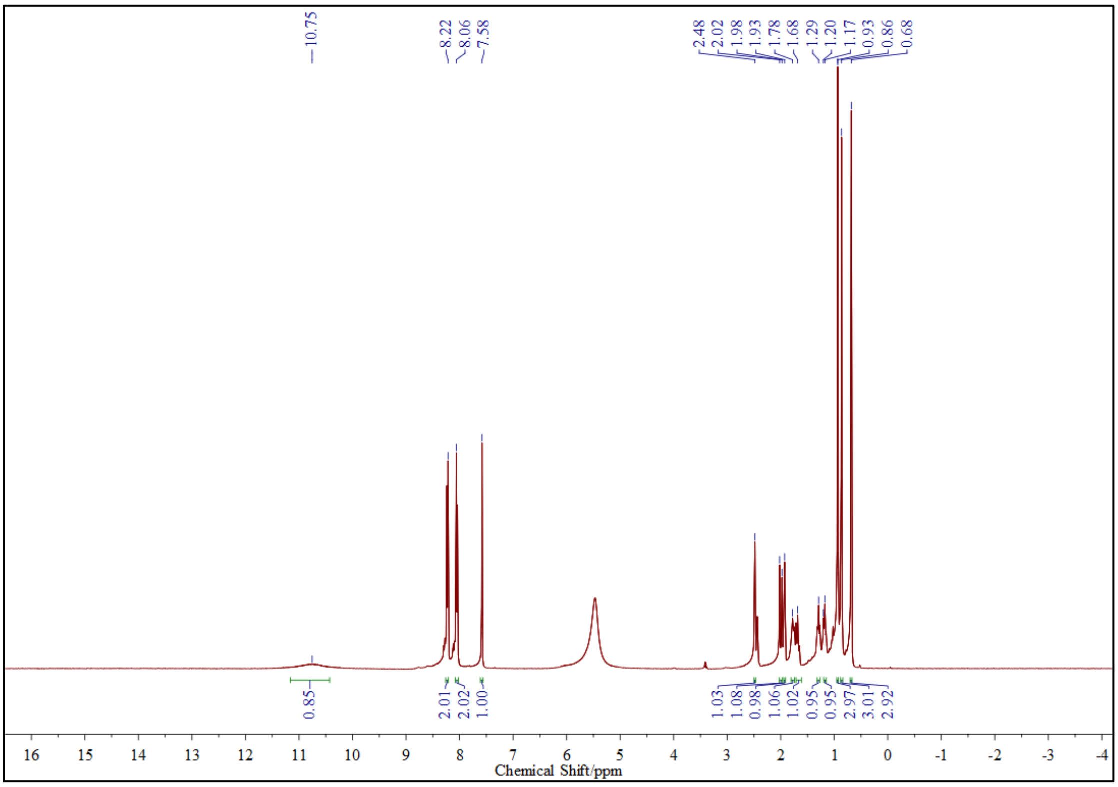

Figure S29. ¹H-NMR spectrum of compound 5i.

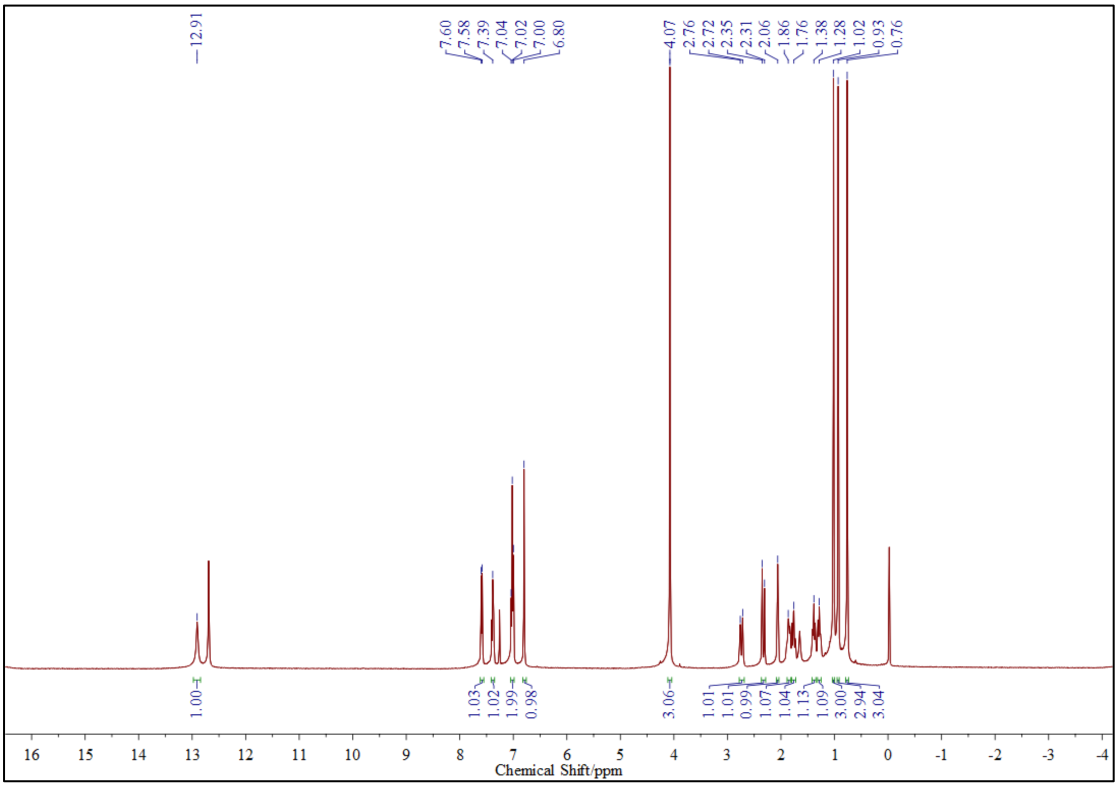

Figure S30. ¹H-NMR spectrum of compound 5j.

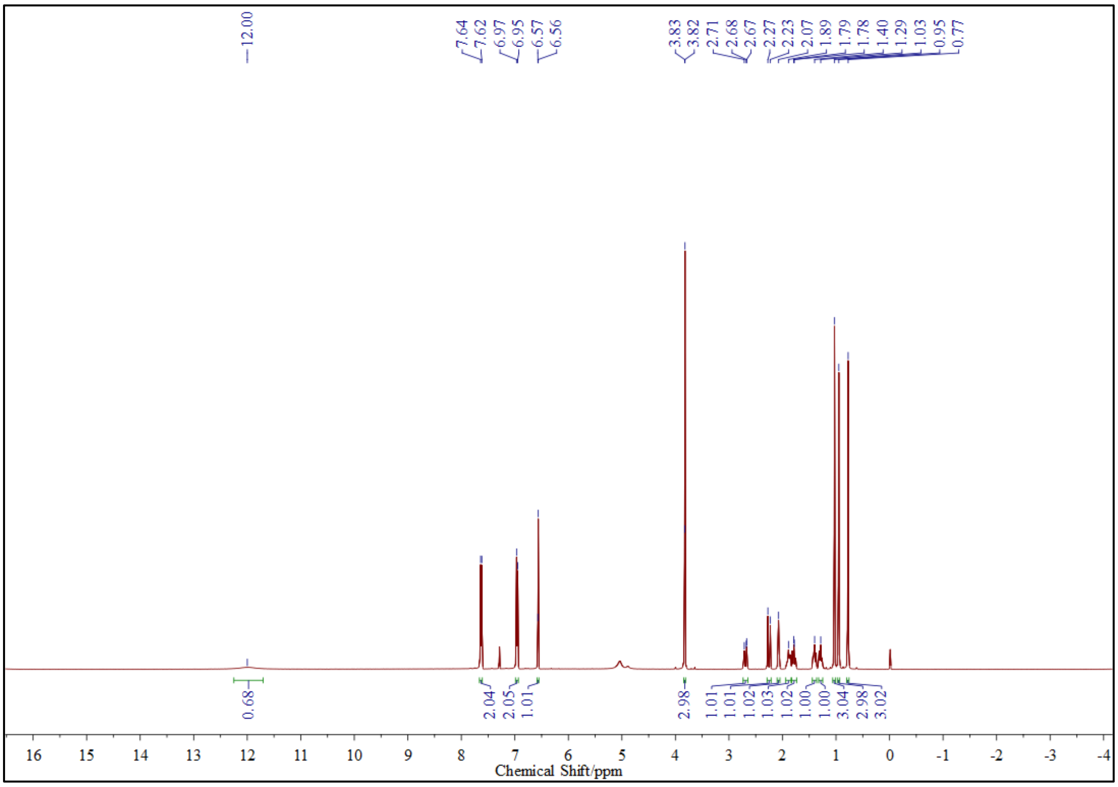

Figure S31. ¹H-NMR spectrum of compound 5k.

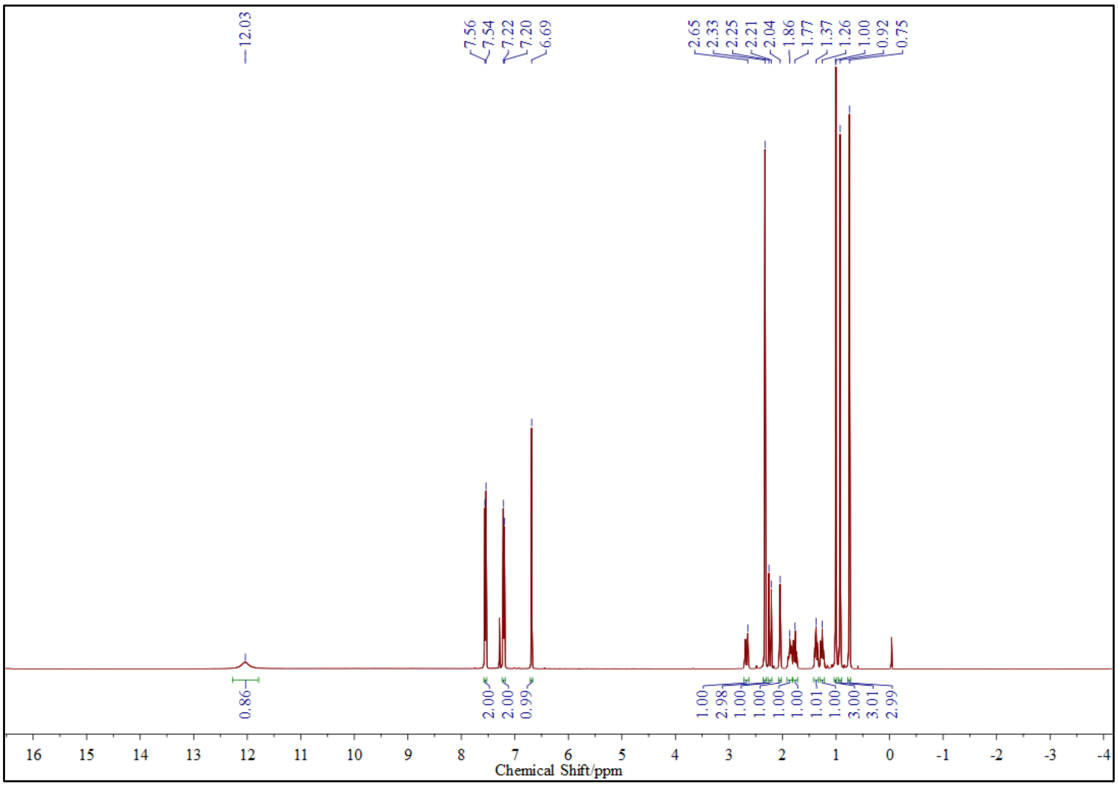

Figure S32. ¹H-NMR spectrum of compound 5l.

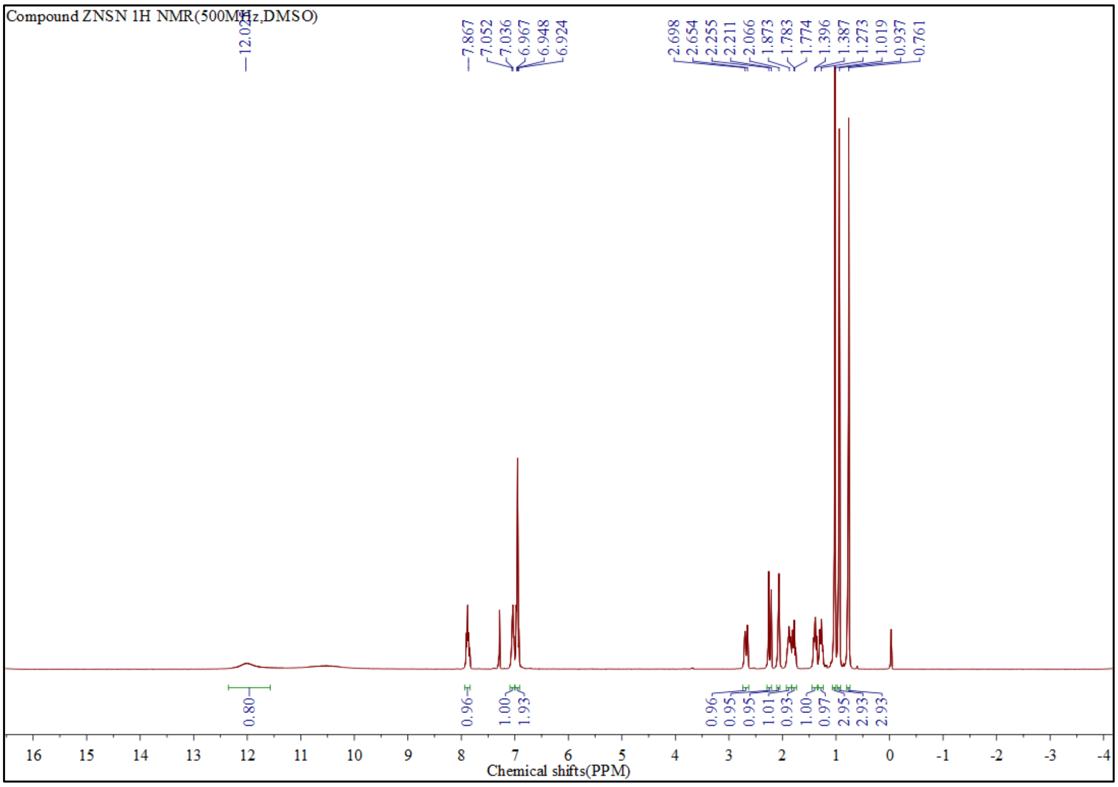

Figure S33. ^{1}H-NMR spectrum of compound 5m.

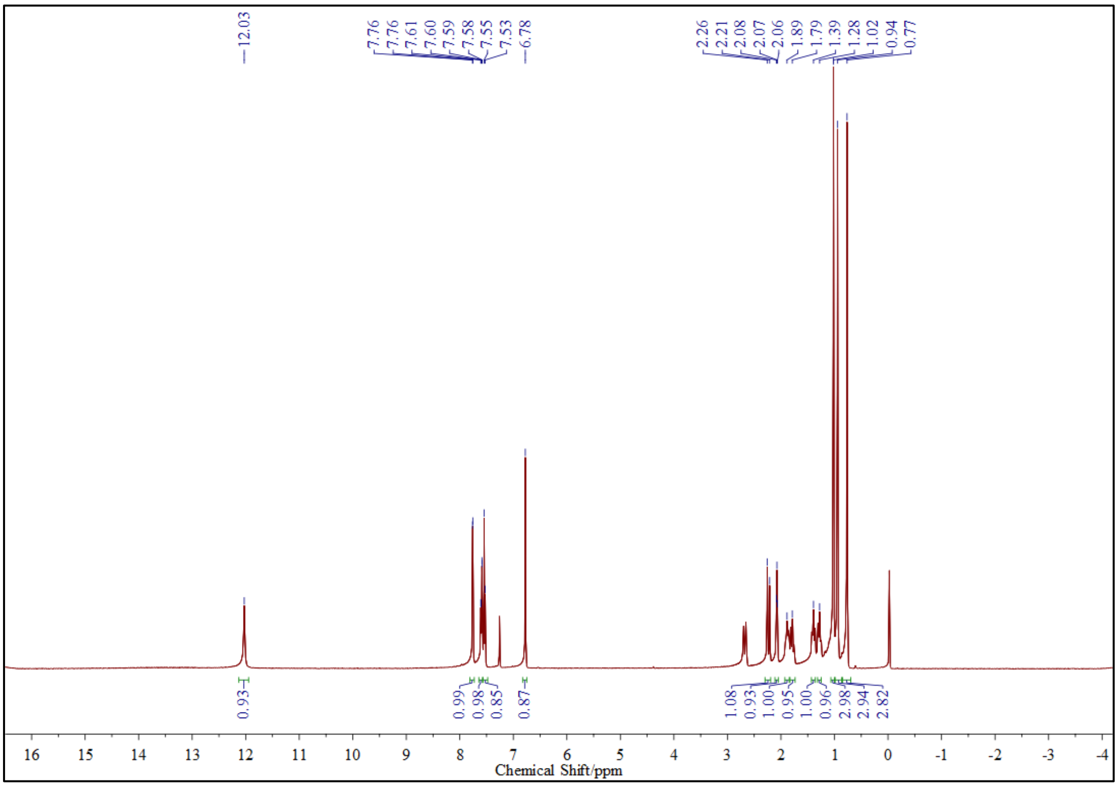

Figure S34. ^{1}H-NMR spectrum of compound 5n.

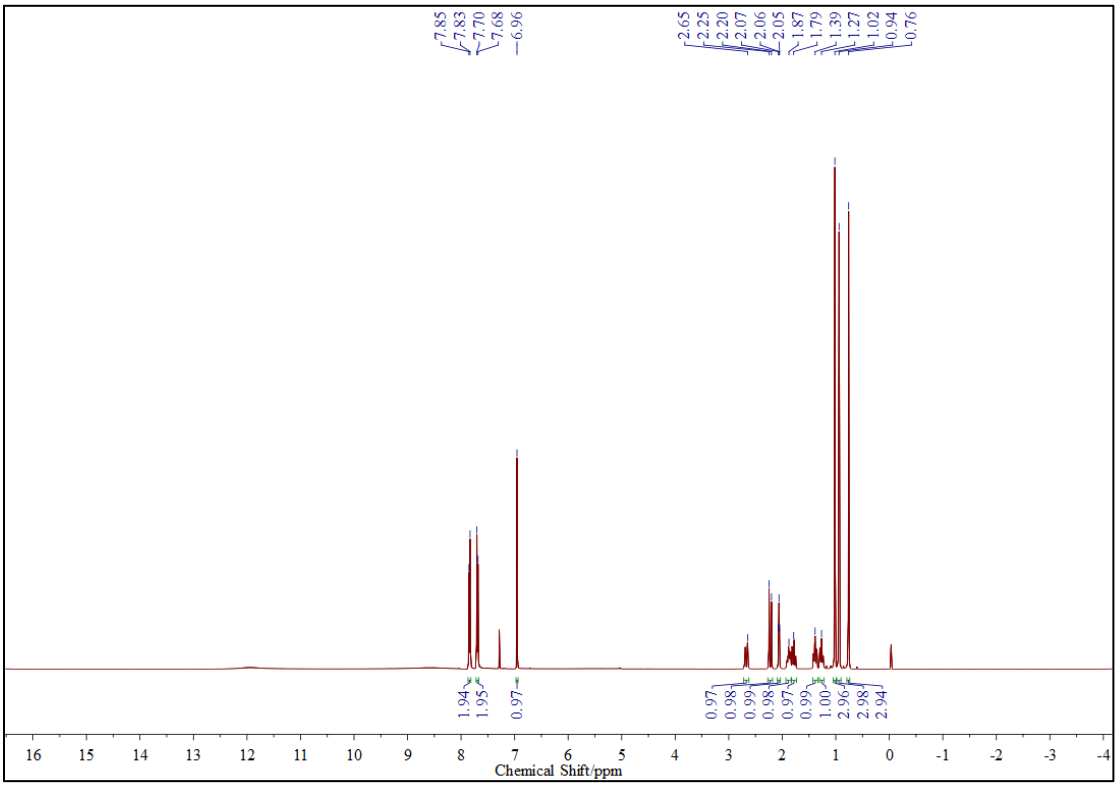

Figure S35. ^{1}H-NMR spectrum of compound 5o.

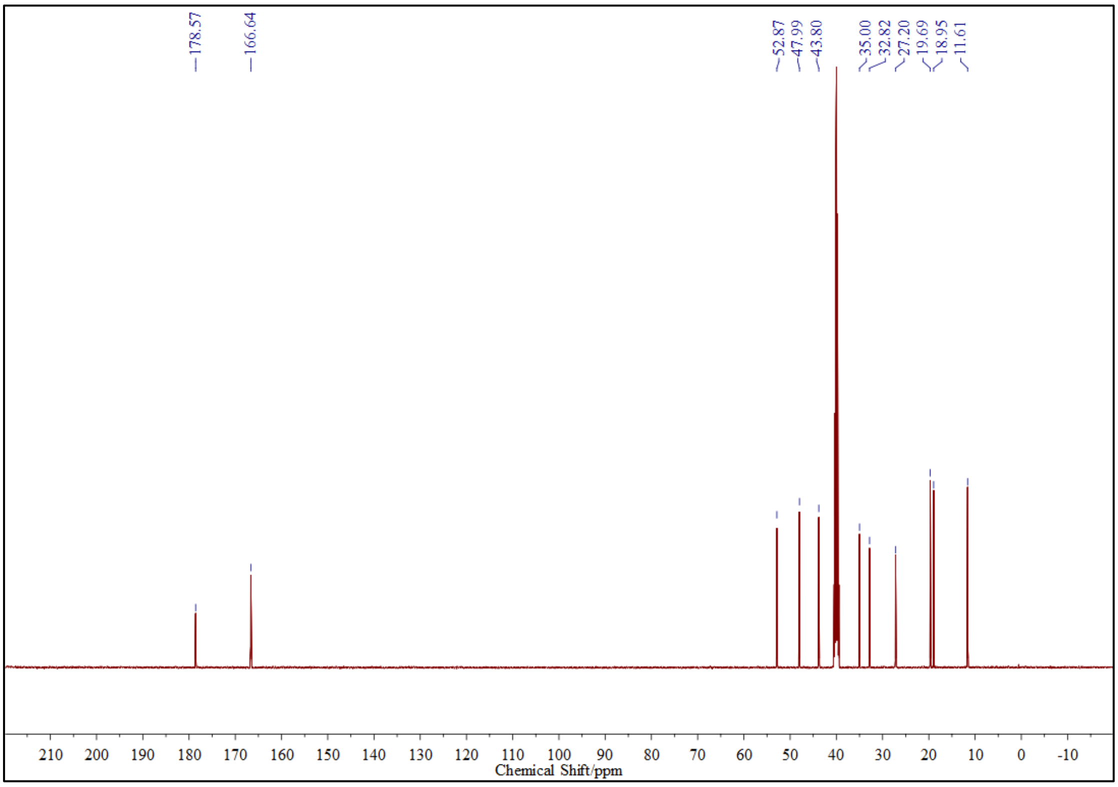

Figure S36. ^{13}C-NMR spectrum of compound 3a.

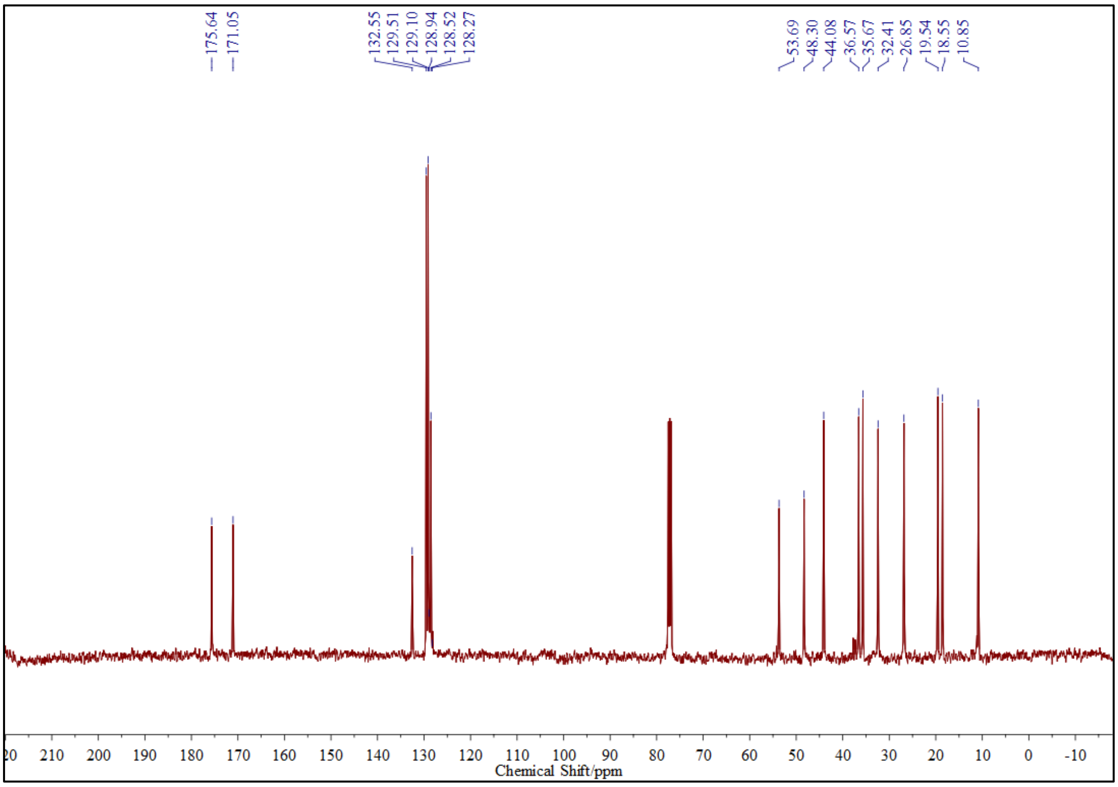

Figure S37. ^{13}C-NMR spectrum of compound 4a.

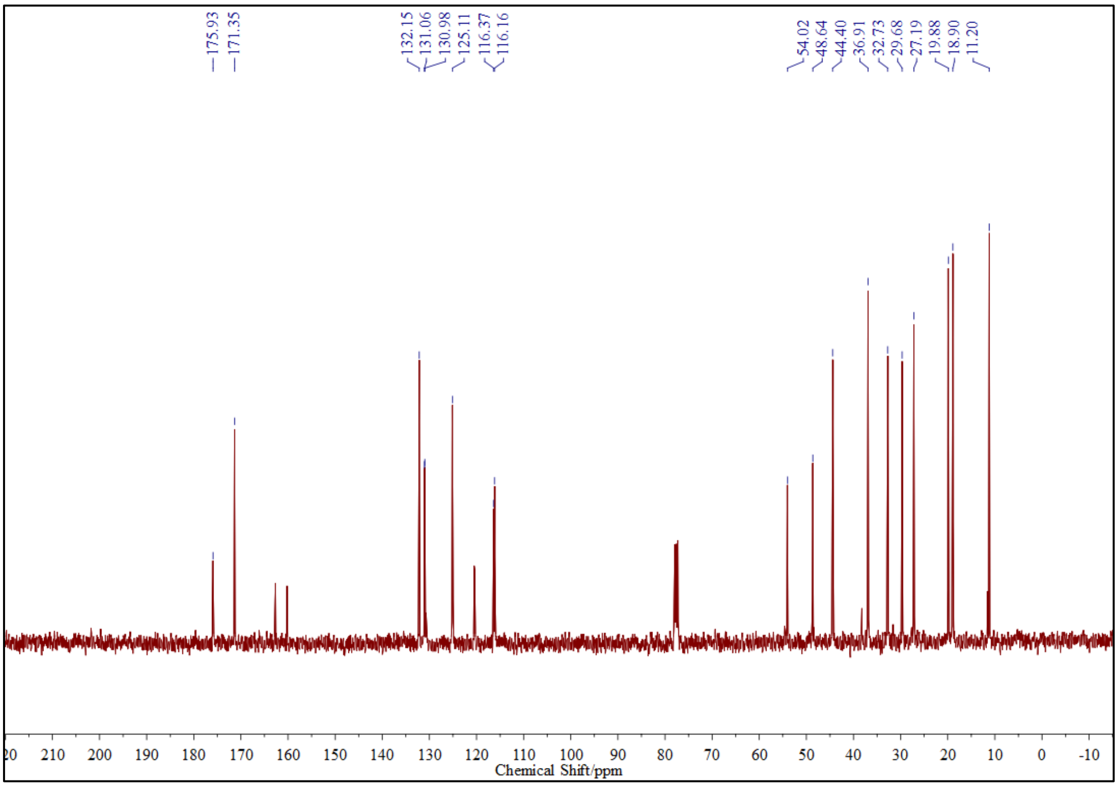

Figure S38. ^{13}C-NMR spectrum of compound 4b.

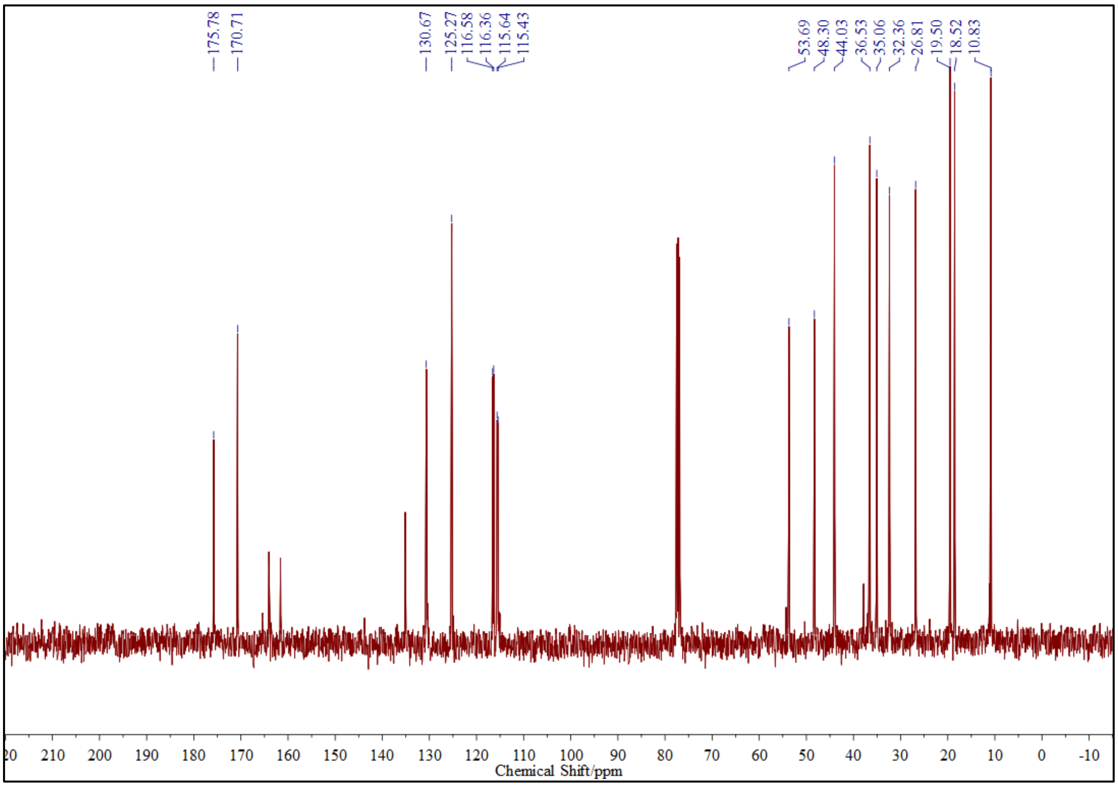

Figure S39. ^{13}C-NMR spectrum of compound 4c.

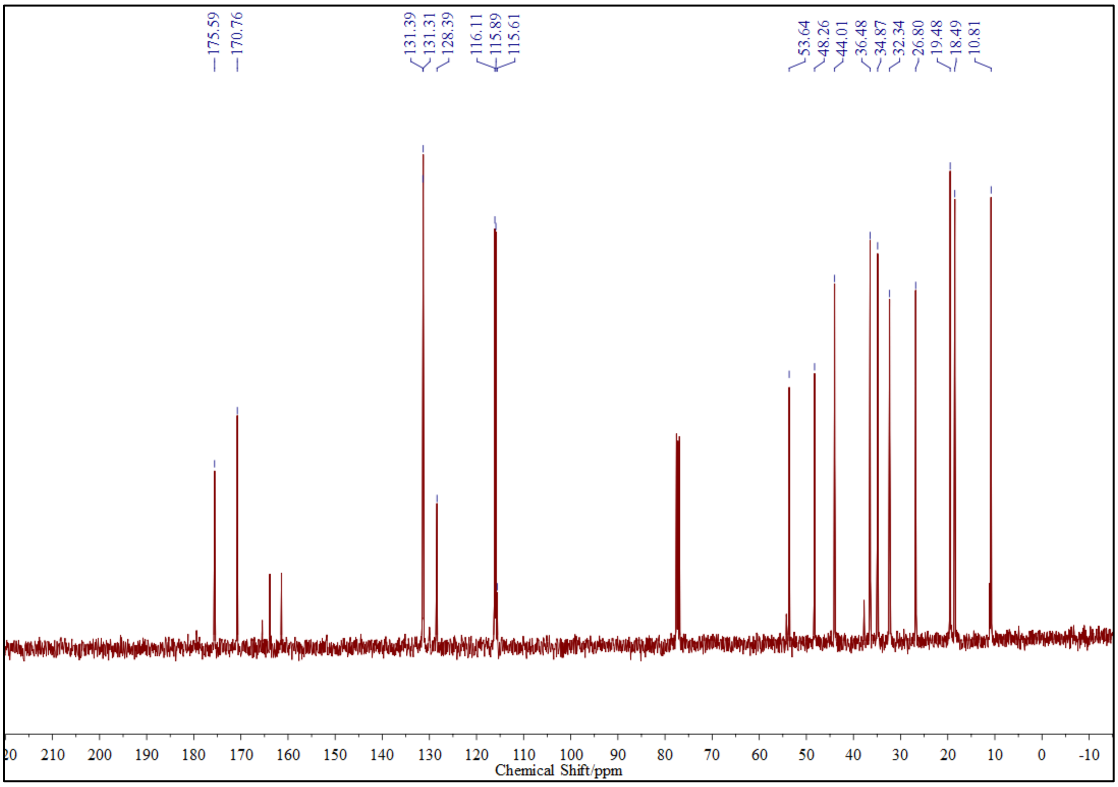

Figure S40. ^{13}C-NMR spectrum of compound 4d.

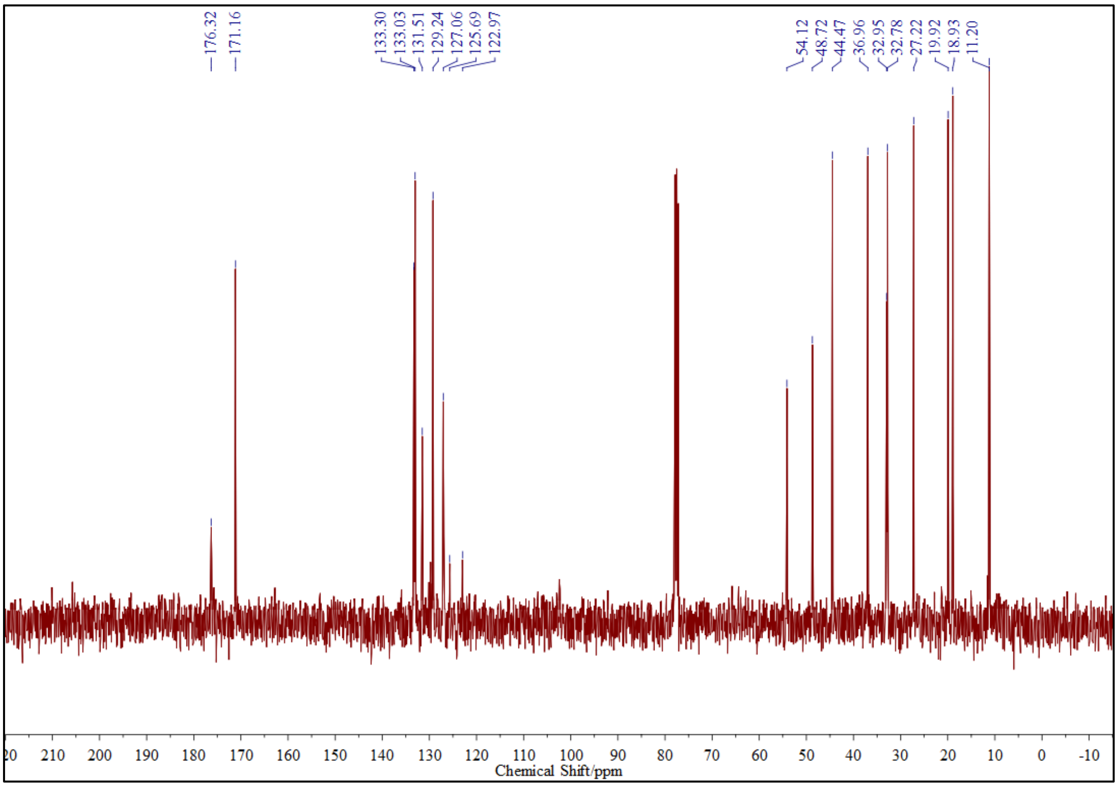

Figure S41. ^{13}C-NMR spectrum of compound 4e.

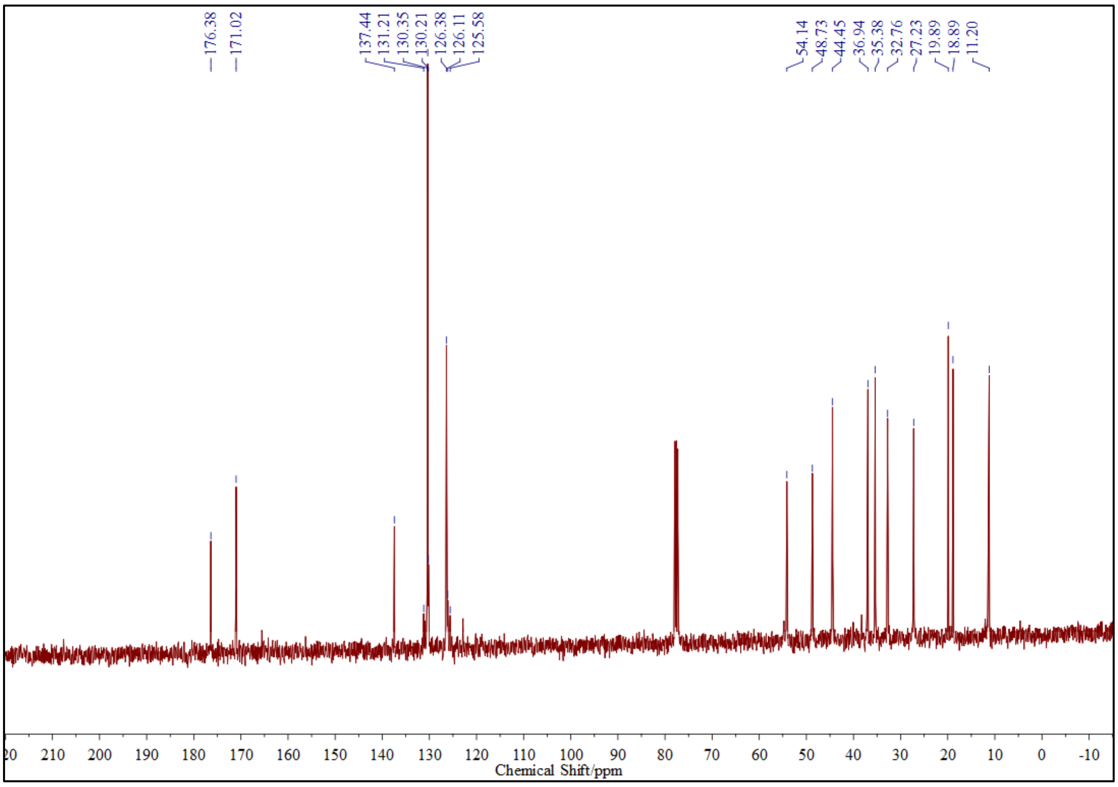

Figure S42. ^{13}C-NMR spectrum of compound 4f.

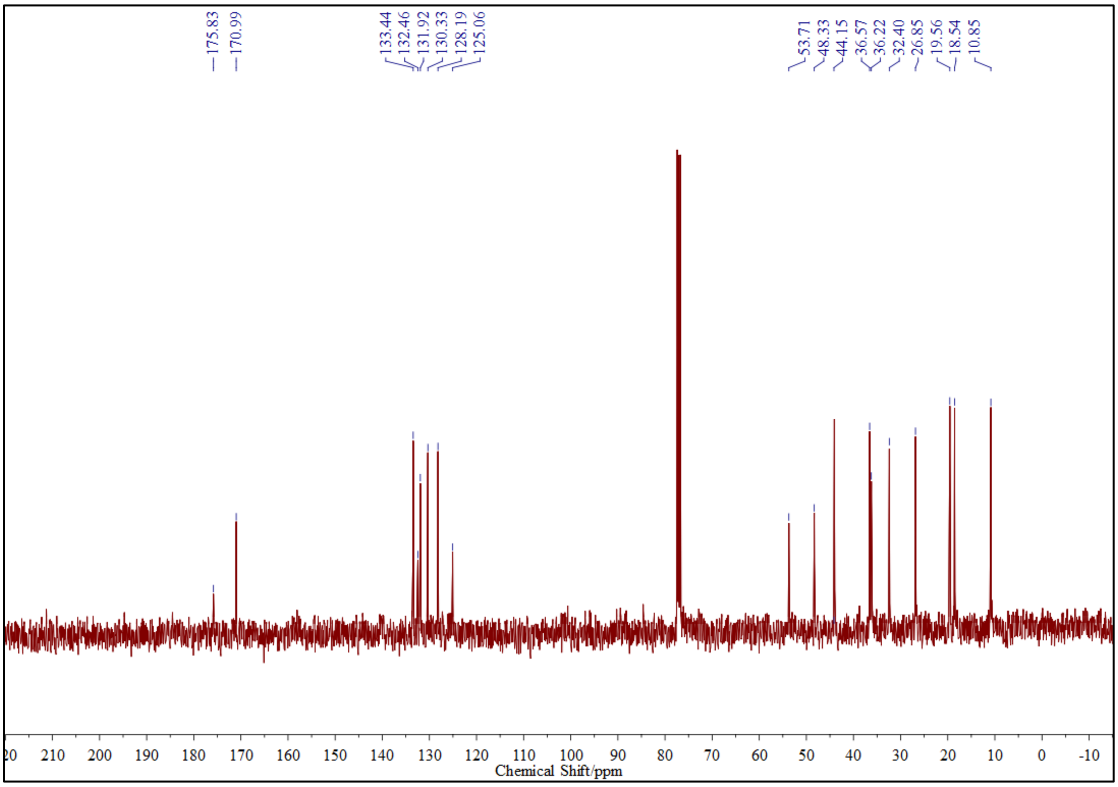

Figure S43. ^{13}C-NMR spectrum of compound 4g.

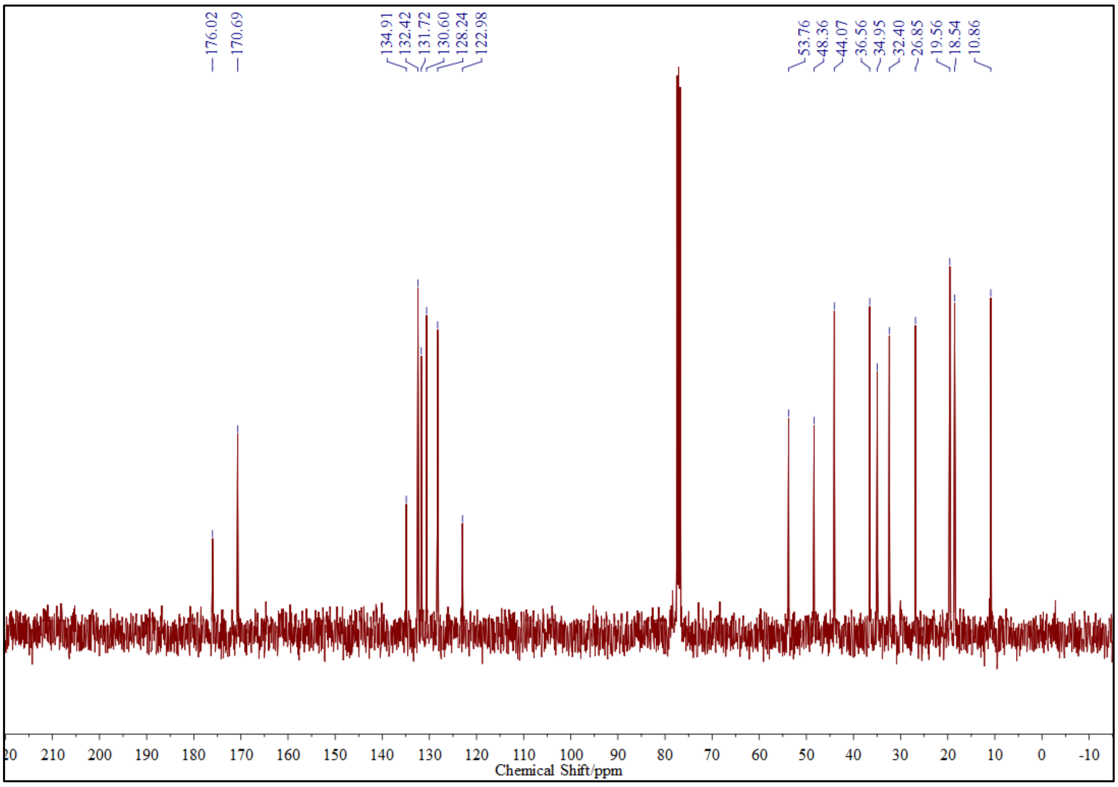

Figure S44. ^{13}C-NMR spectrum of compound 4h.

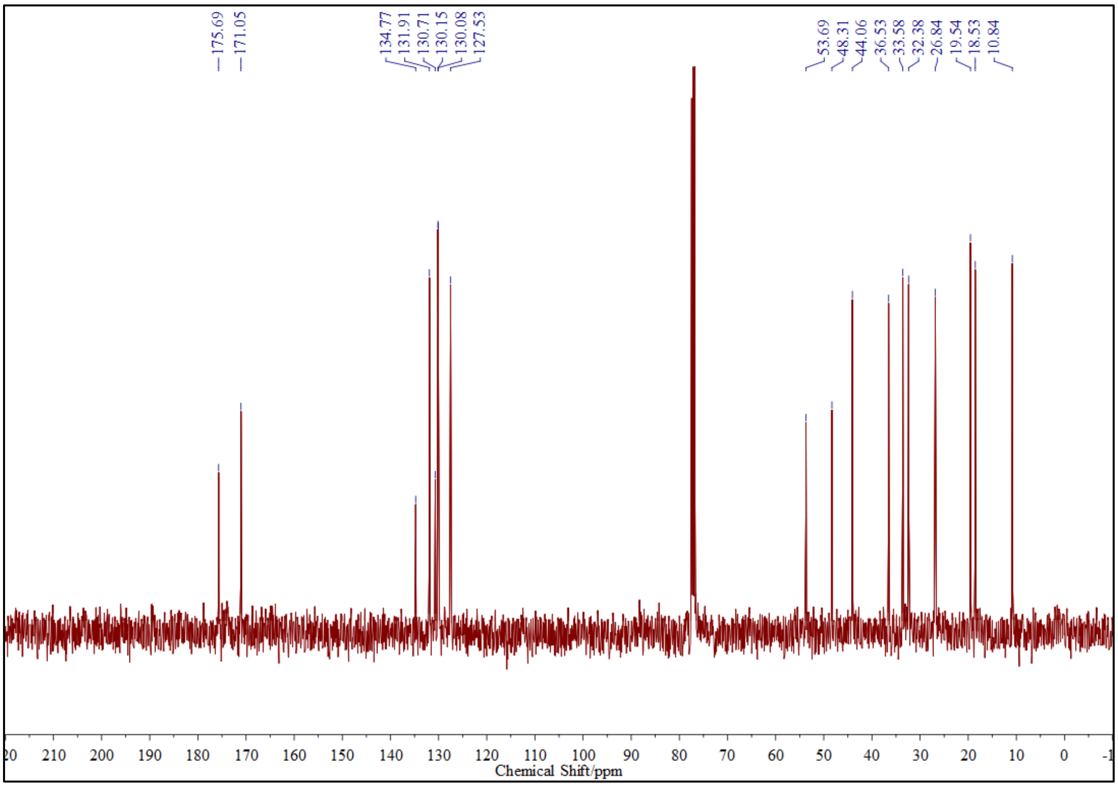

Figure S45. ^{13}C-NMR spectrum of compound 4i.

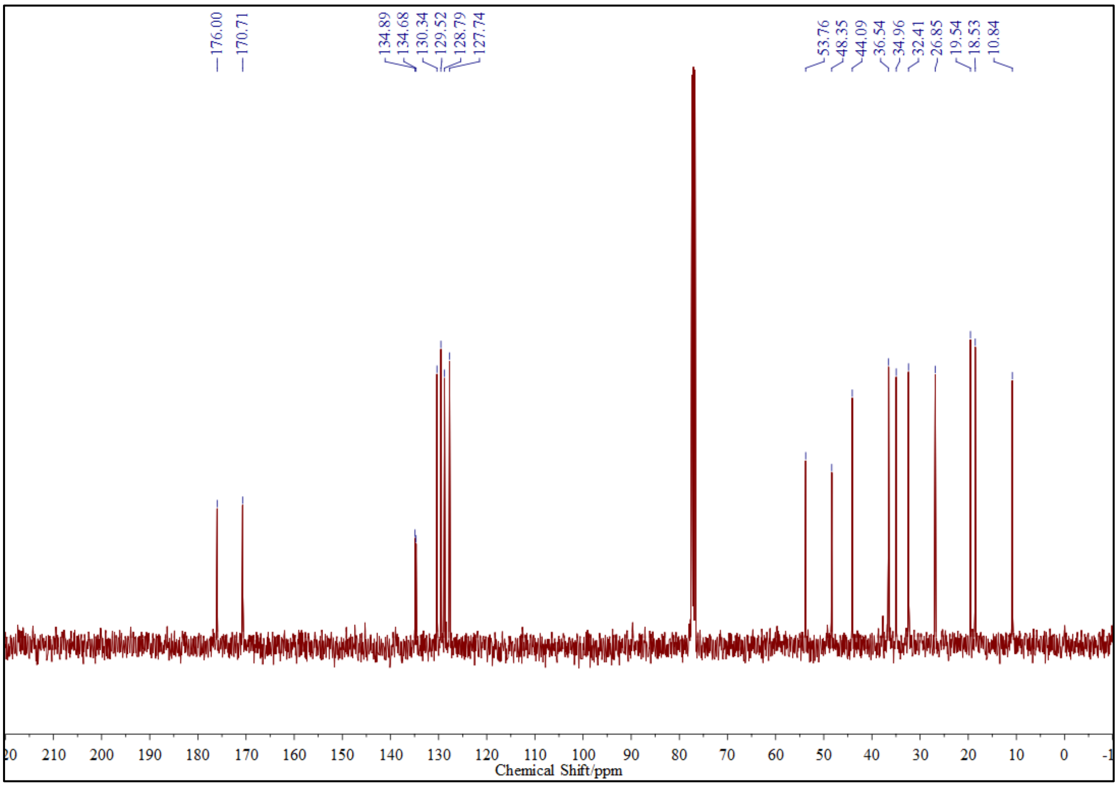

Figure S46. ^{13}C-NMR spectrum of compound 4j.

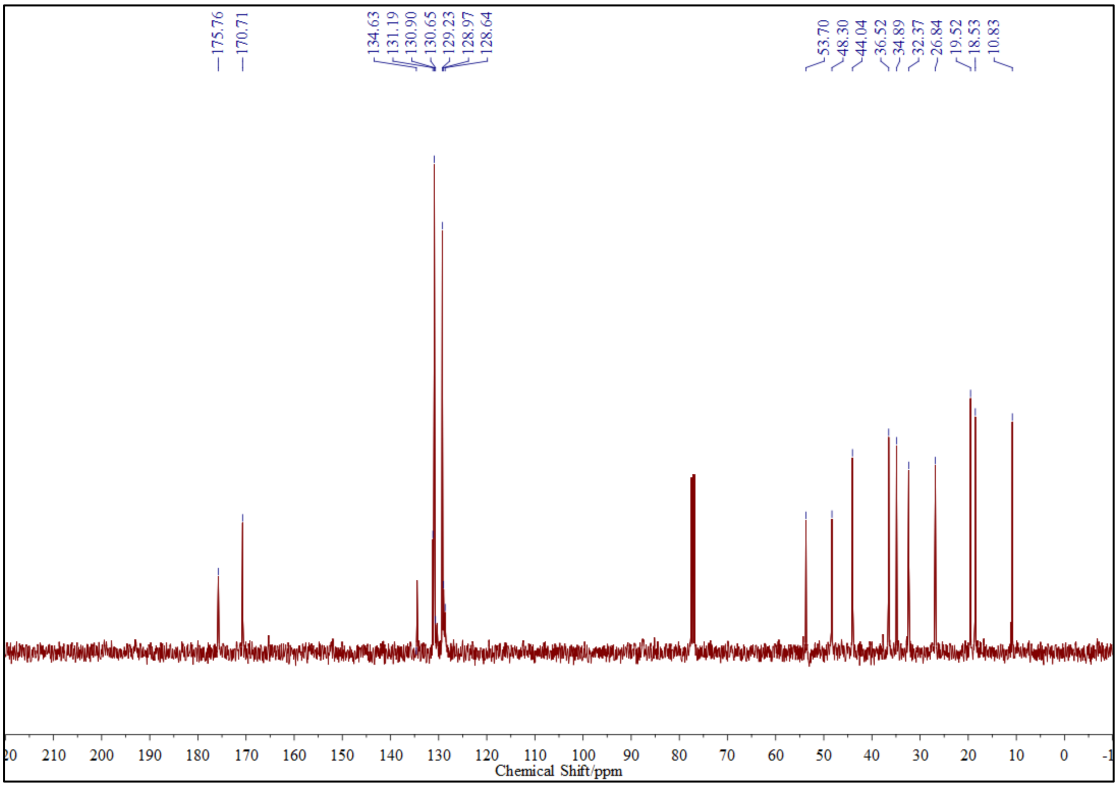

Figure S47. ^{13}C-NMR spectrum of compound 4k.

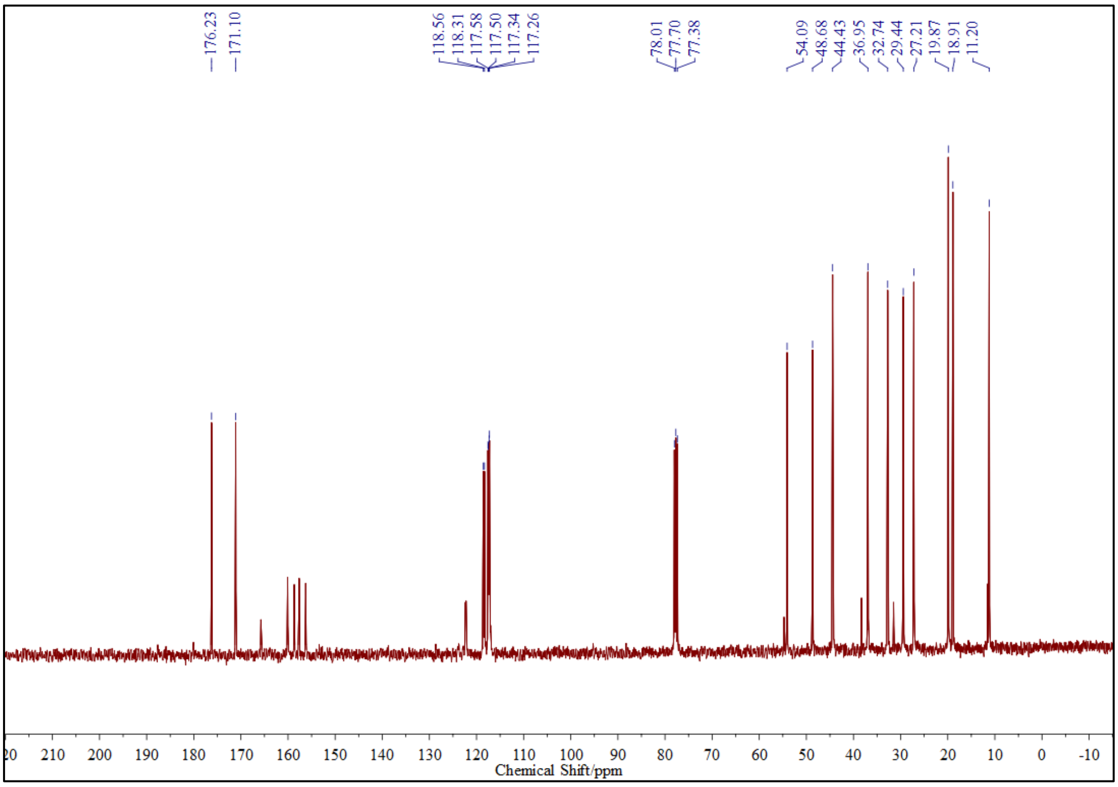

Figure S48. ^{13}C-NMR spectrum of compound 4l.

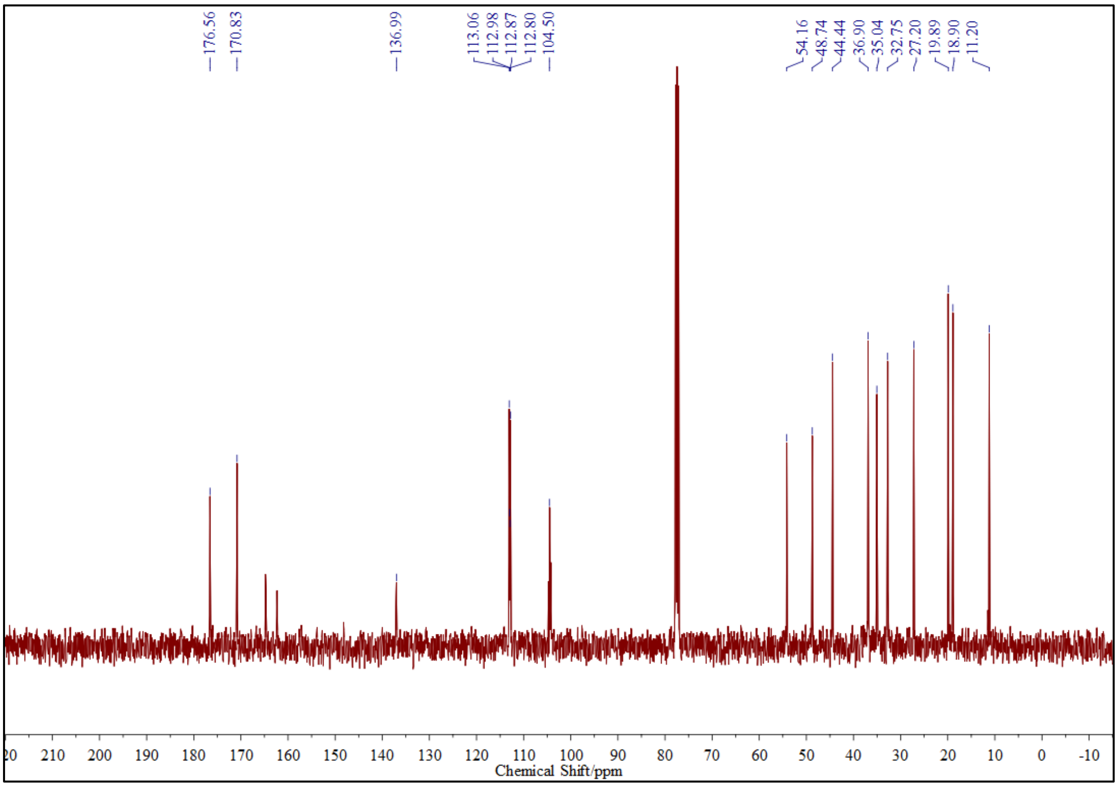

Figure S49. 13C-NMR spectrum of compound 4m.

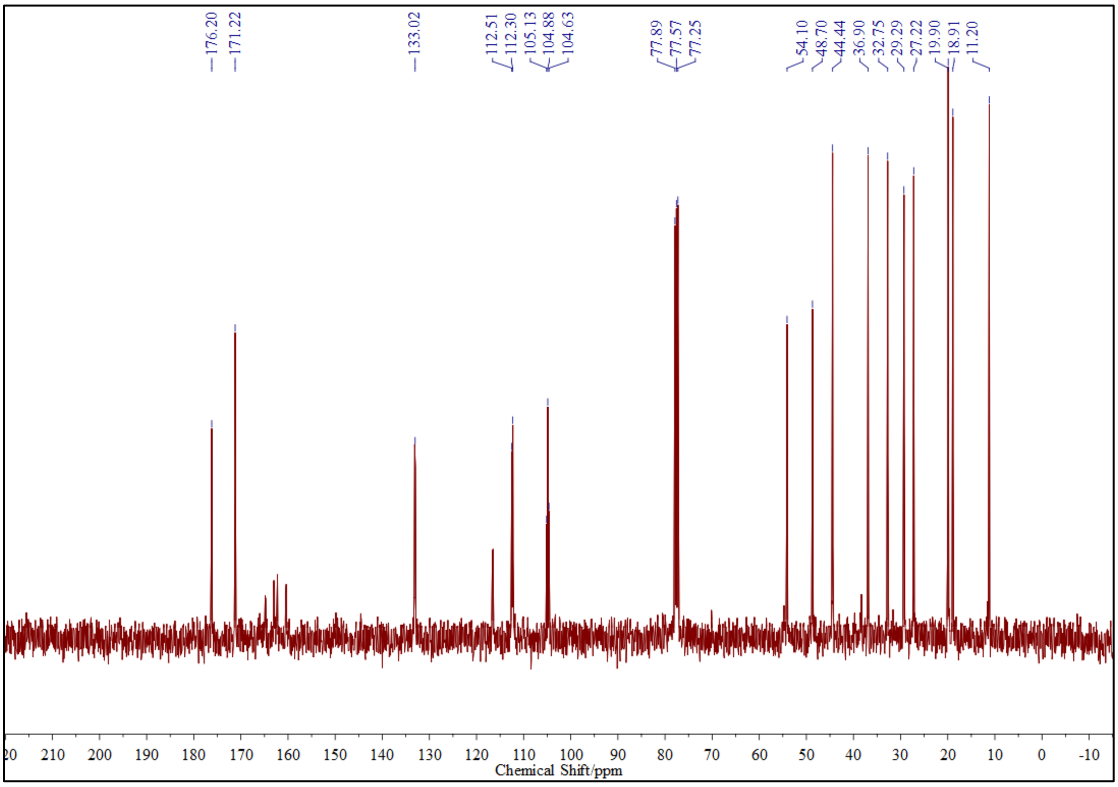

Figure S50. 13C-NMR spectrum of compound 4n.

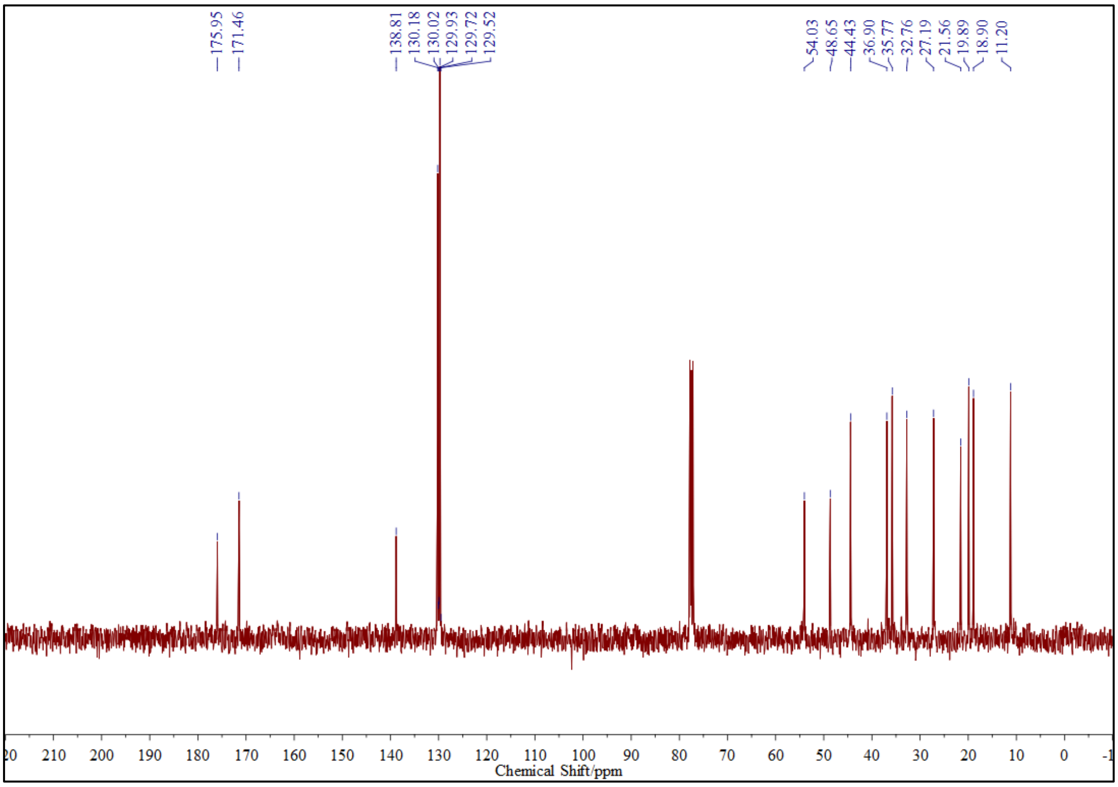

Figure S51. ^{13}C-NMR spectrum of compound 4o.

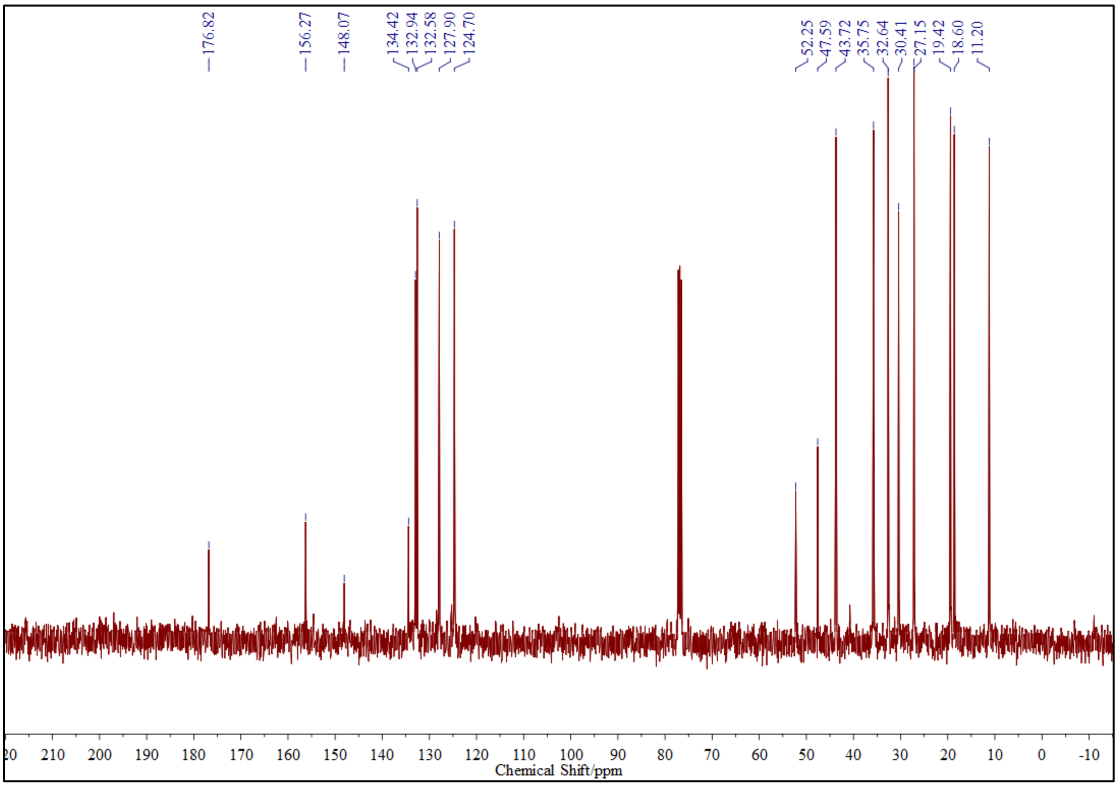

Figure S52. ^{13}C-NMR spectrum of compound 4p.

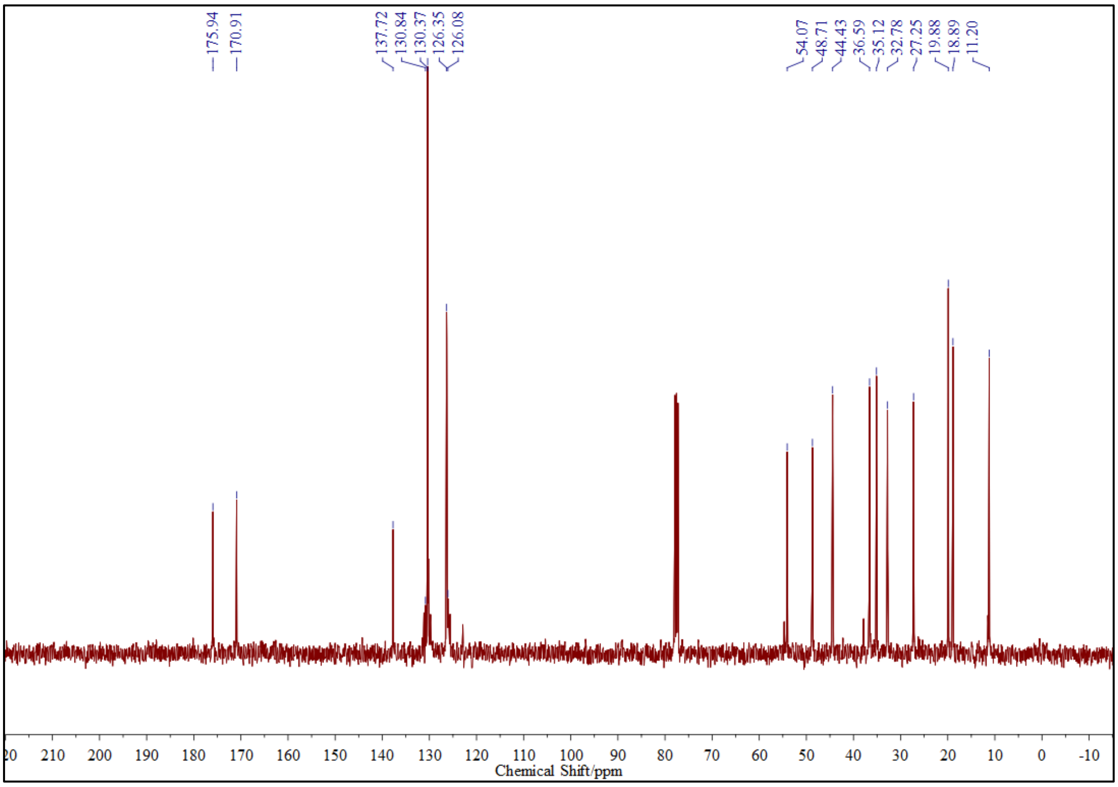

Figure S53. ^{13}C-NMR spectrum of compound 4q.

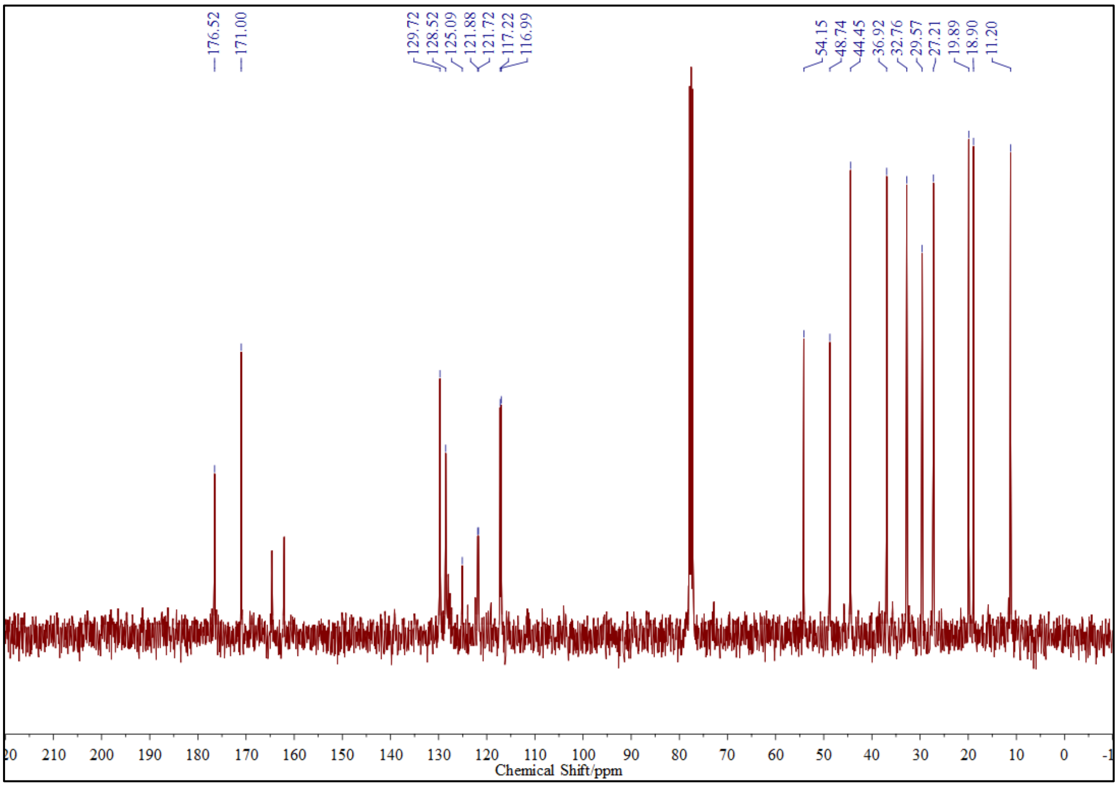

Figure S54. ^{13}C-NMR spectrum of compound 4r.

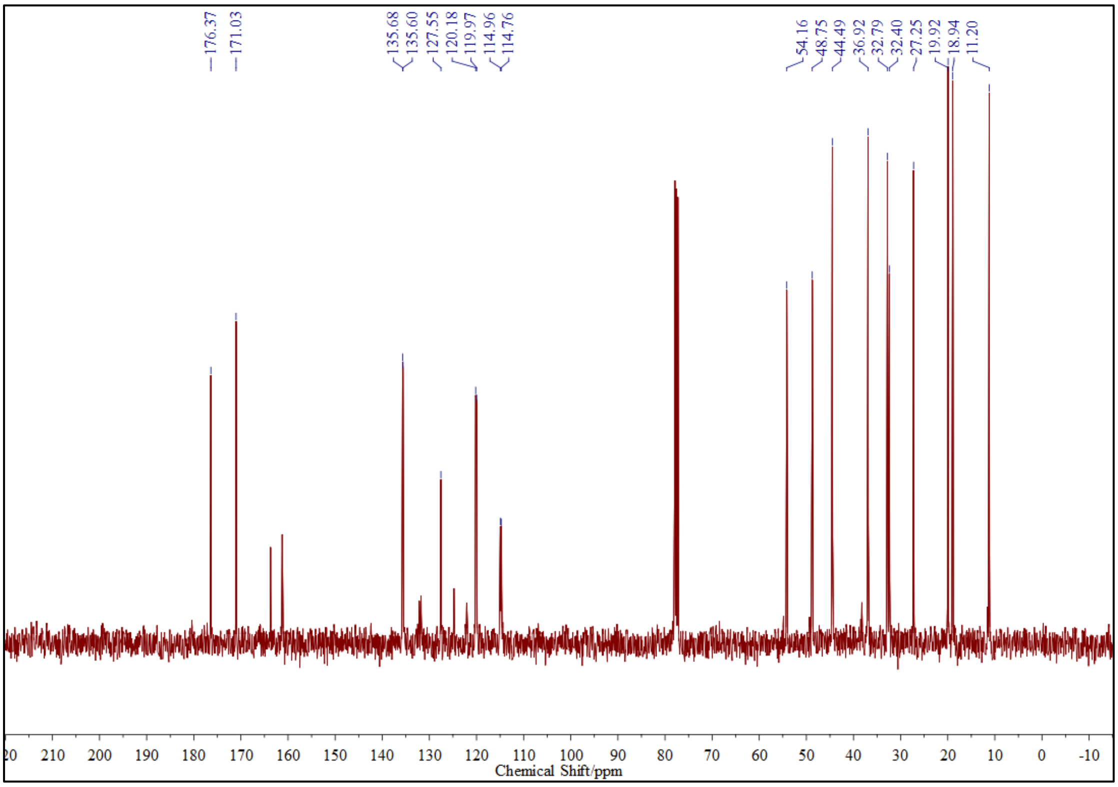

Figure S55. ^{13}C-NMR spectrum of compound 4s.

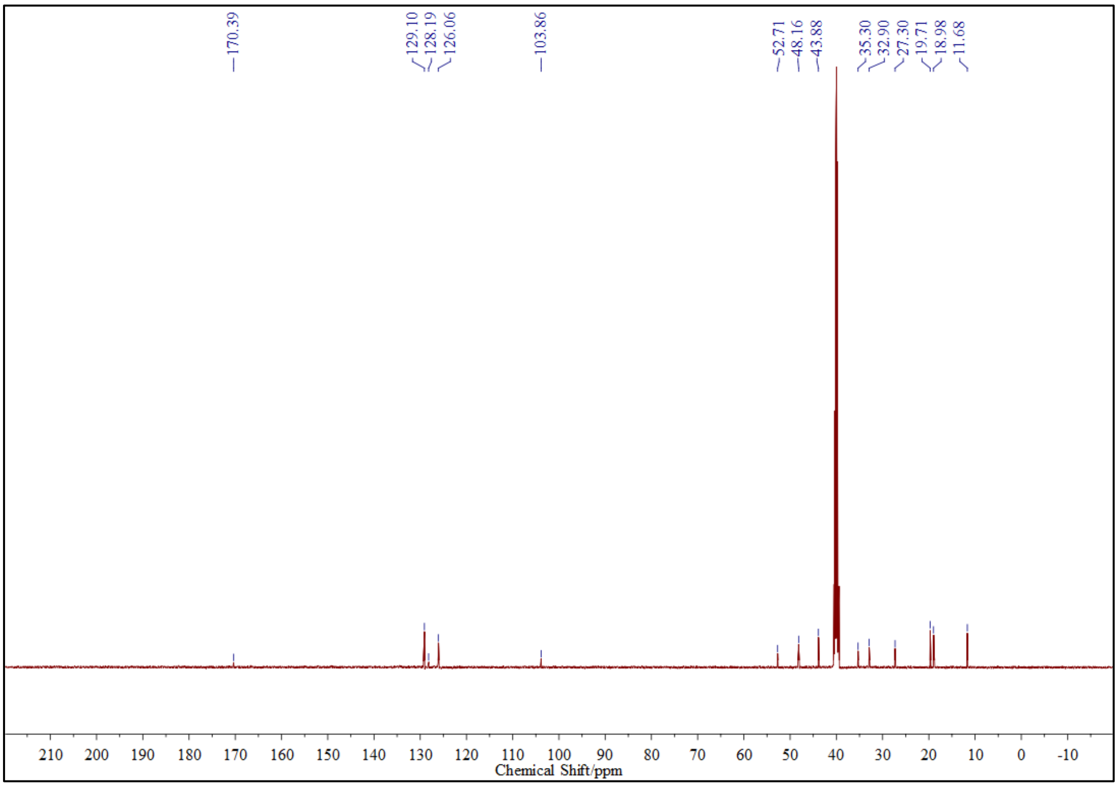

Figure S56. ^{13}C-NMR spectrum of compound 5a.

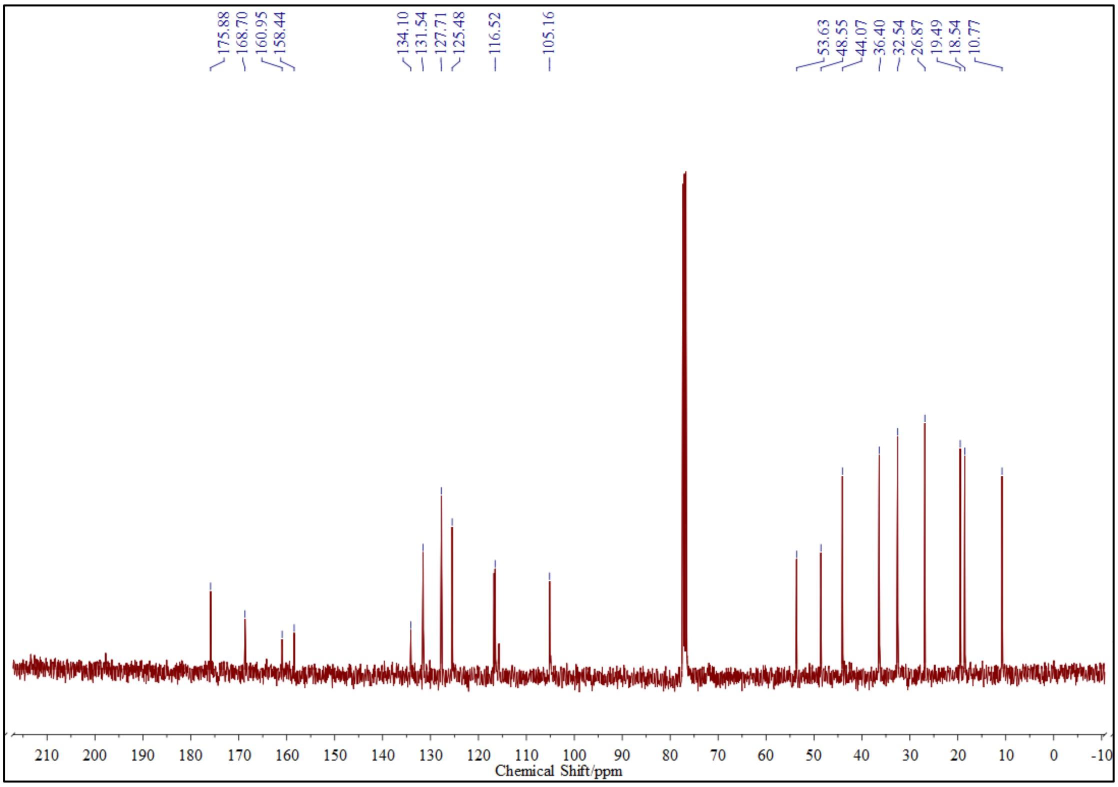

Figure S57. ^{13}C-NMR spectrum of compound 5b.

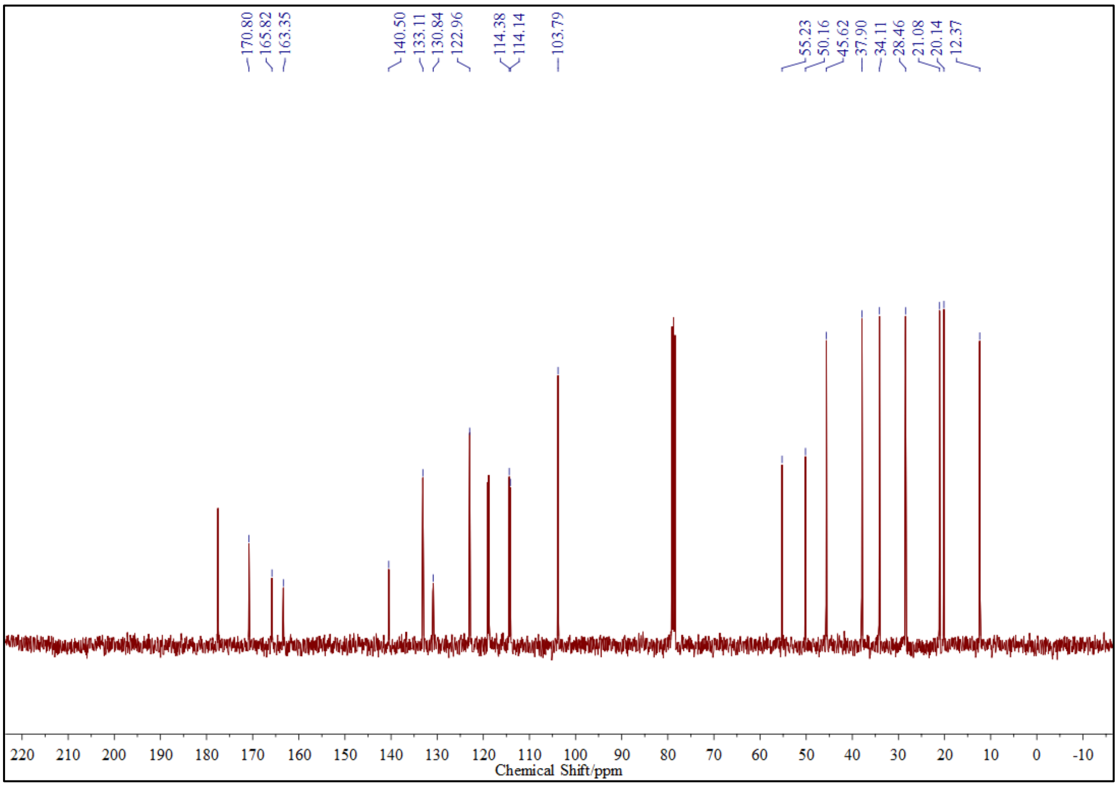

Figure S58. ^{13}C-NMR spectrum of compound 5c.

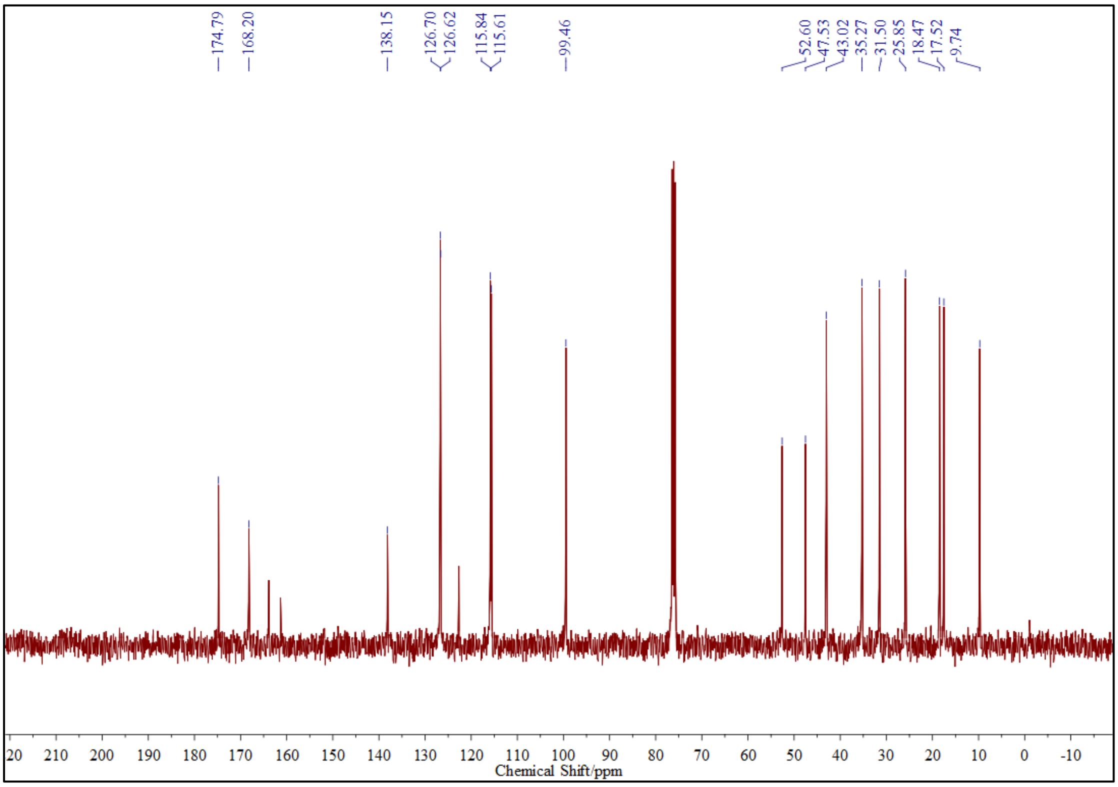

Figure S59. ^{13}C-NMR spectrum of compound 5d.

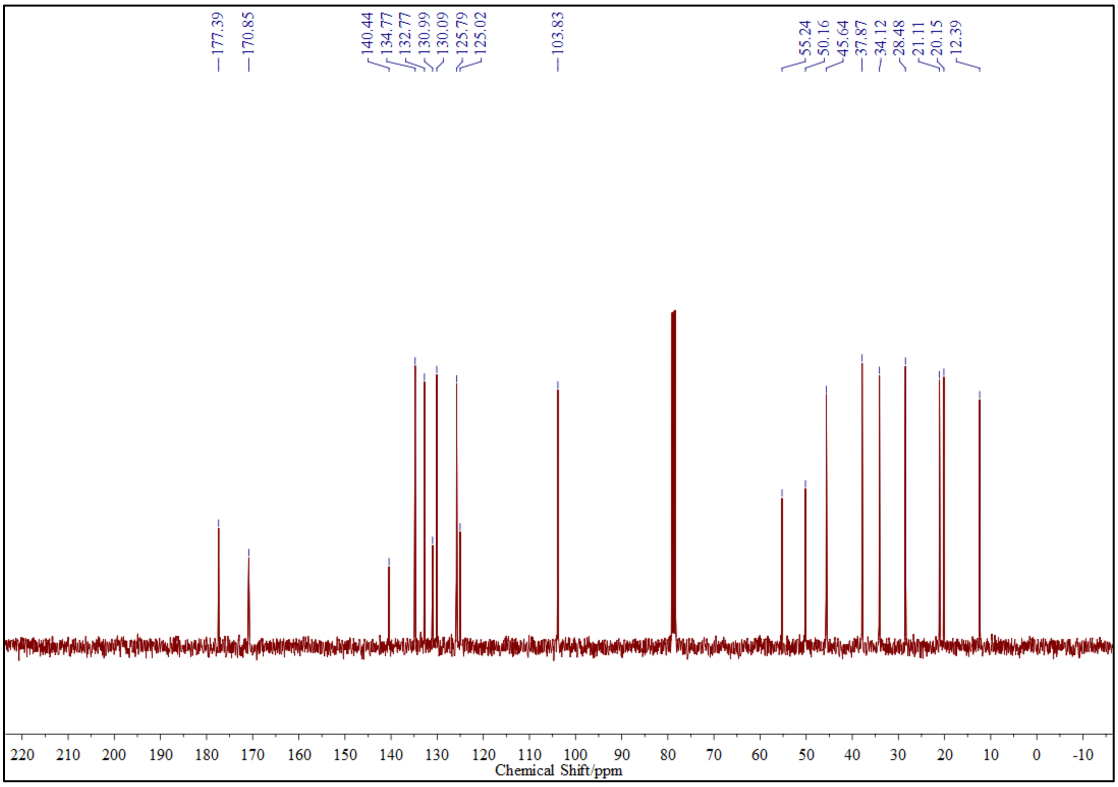

Figure S60. ^{13}C-NMR spectrum of compound 5e.

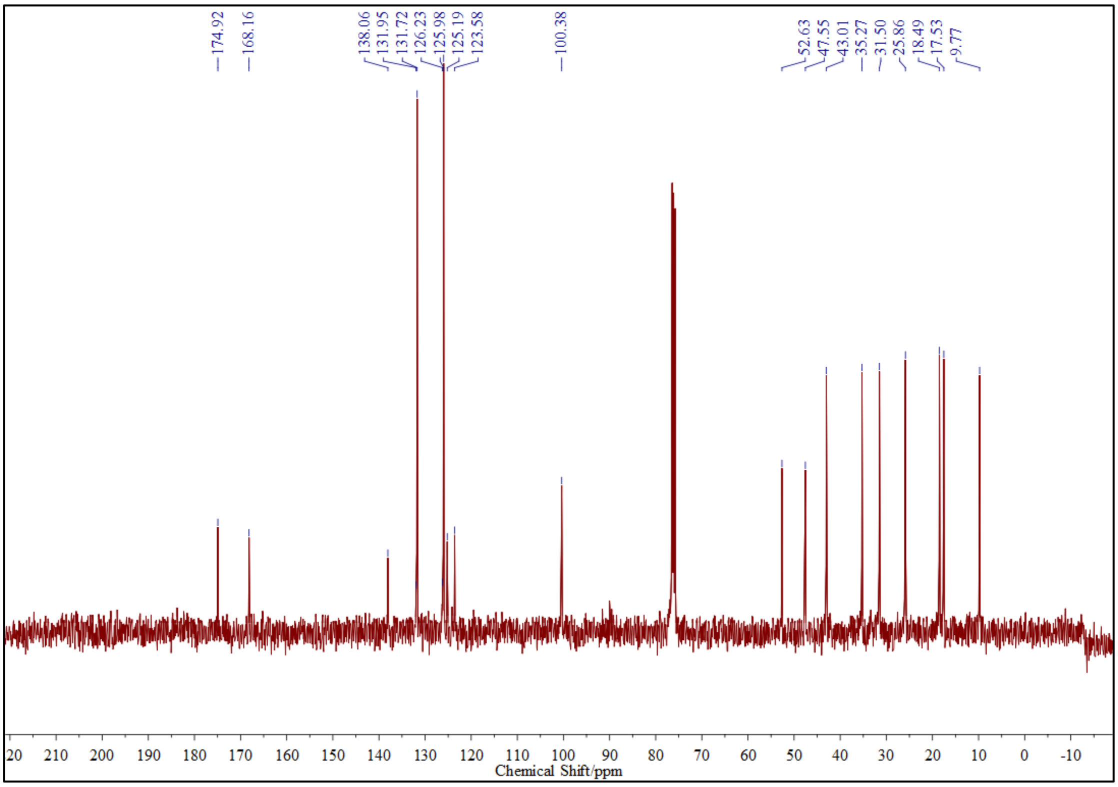

Figure S61. ^{13}C-NMR spectrum of compound 5f.

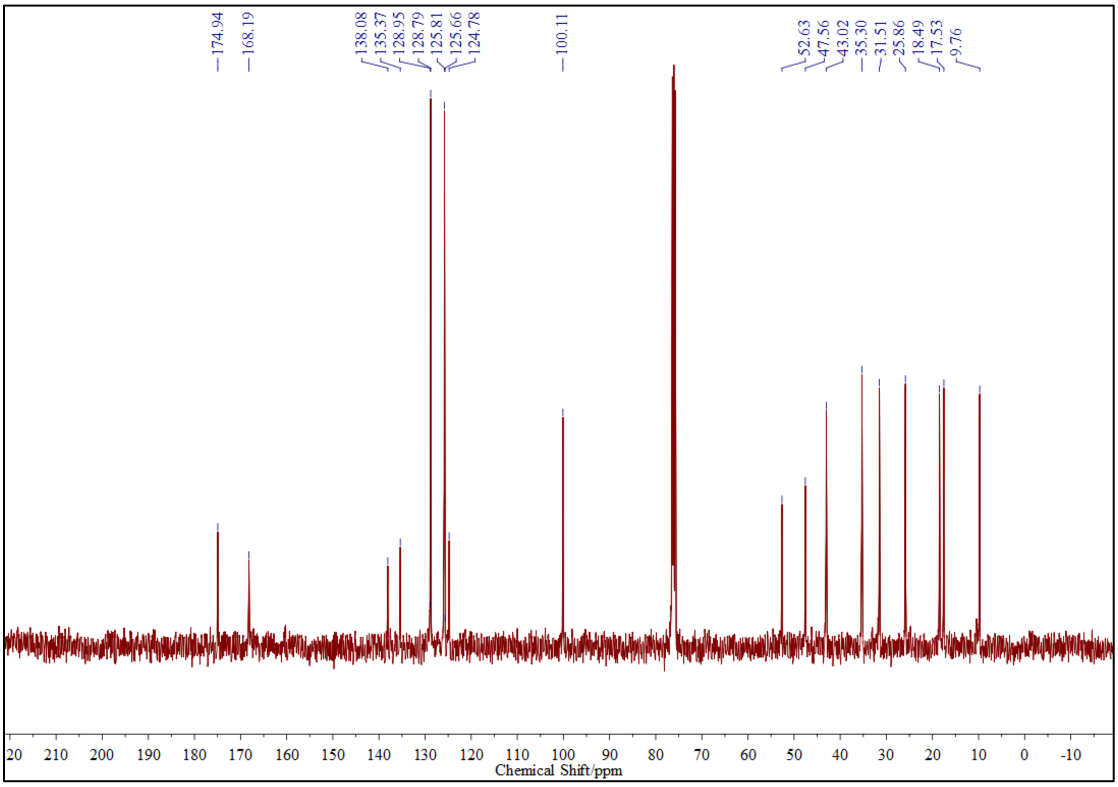

Figure S62. ^{13}C-NMR spectrum of compound 5g.

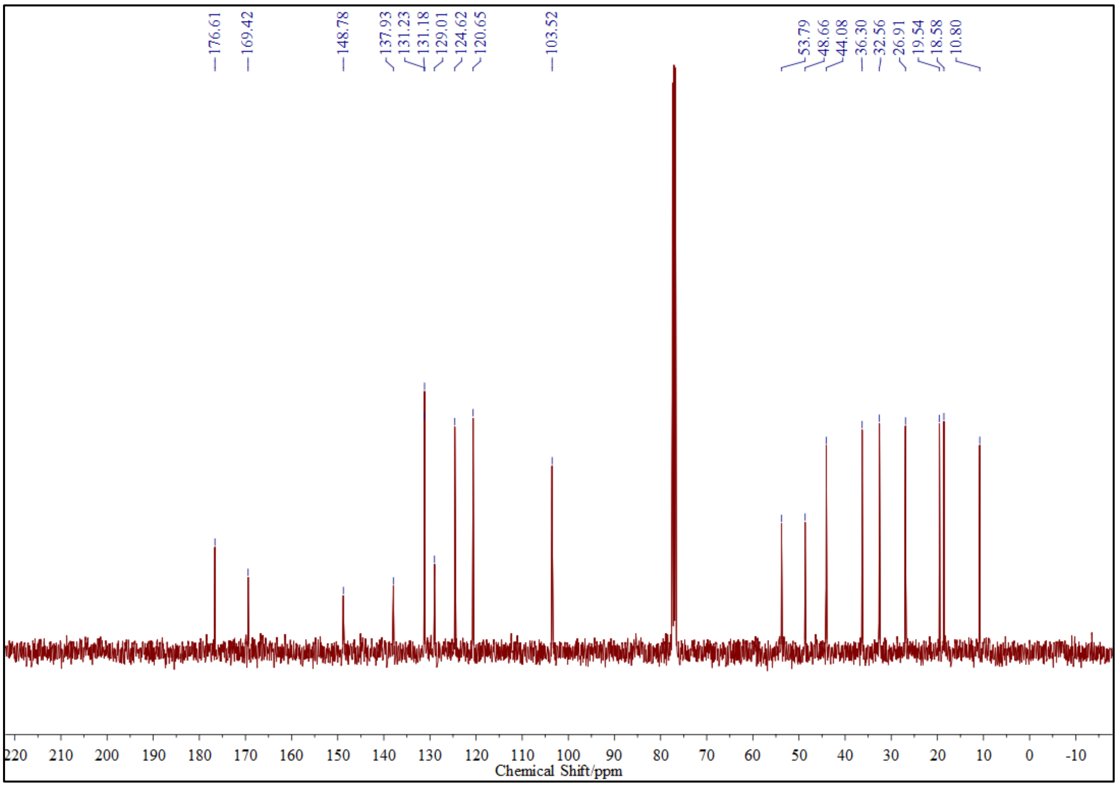

Figure S63. ^{13}C-NMR spectrum of compound 5h.

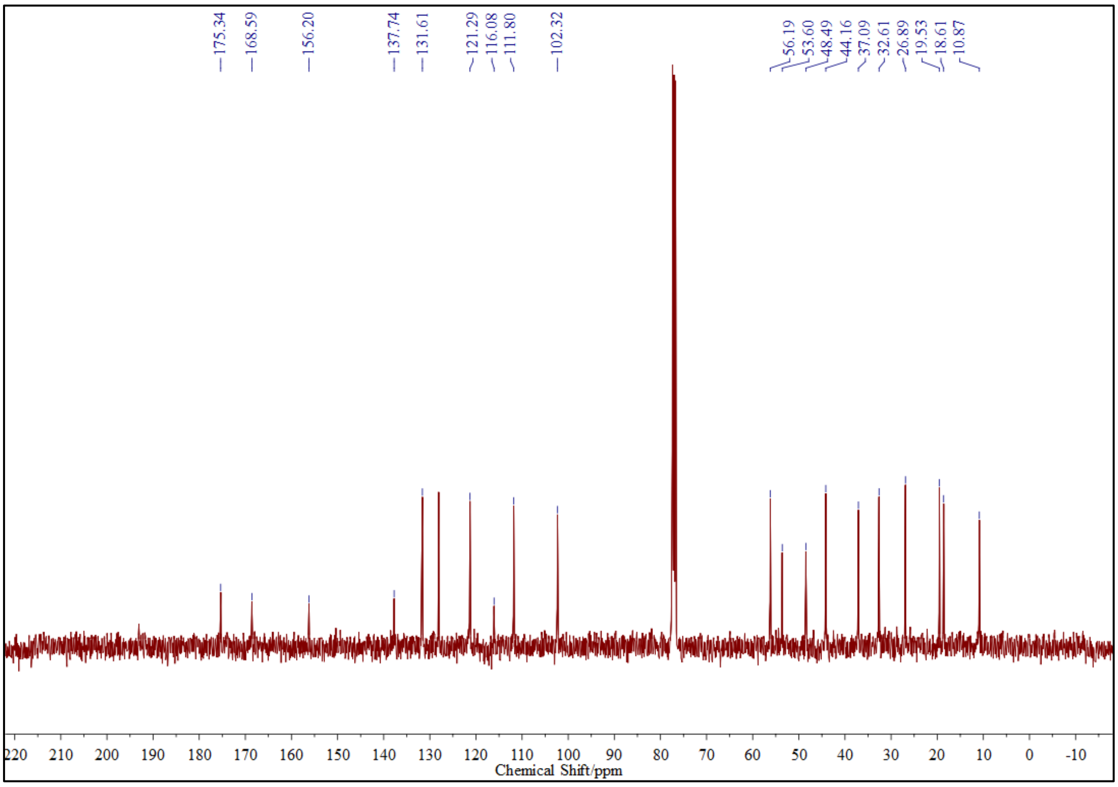

Figure S64. ^{13}C-NMR spectrum of compound 5i.

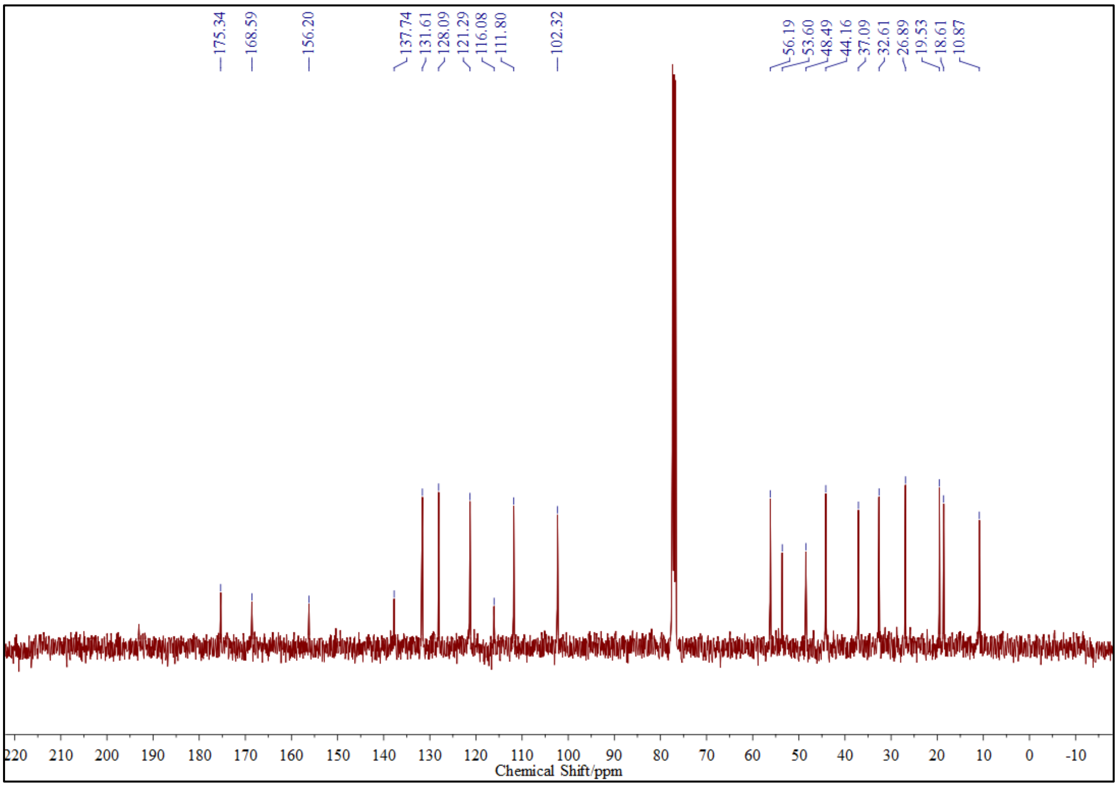

Figure S65. ^{13}C-NMR spectrum of compound 5j.

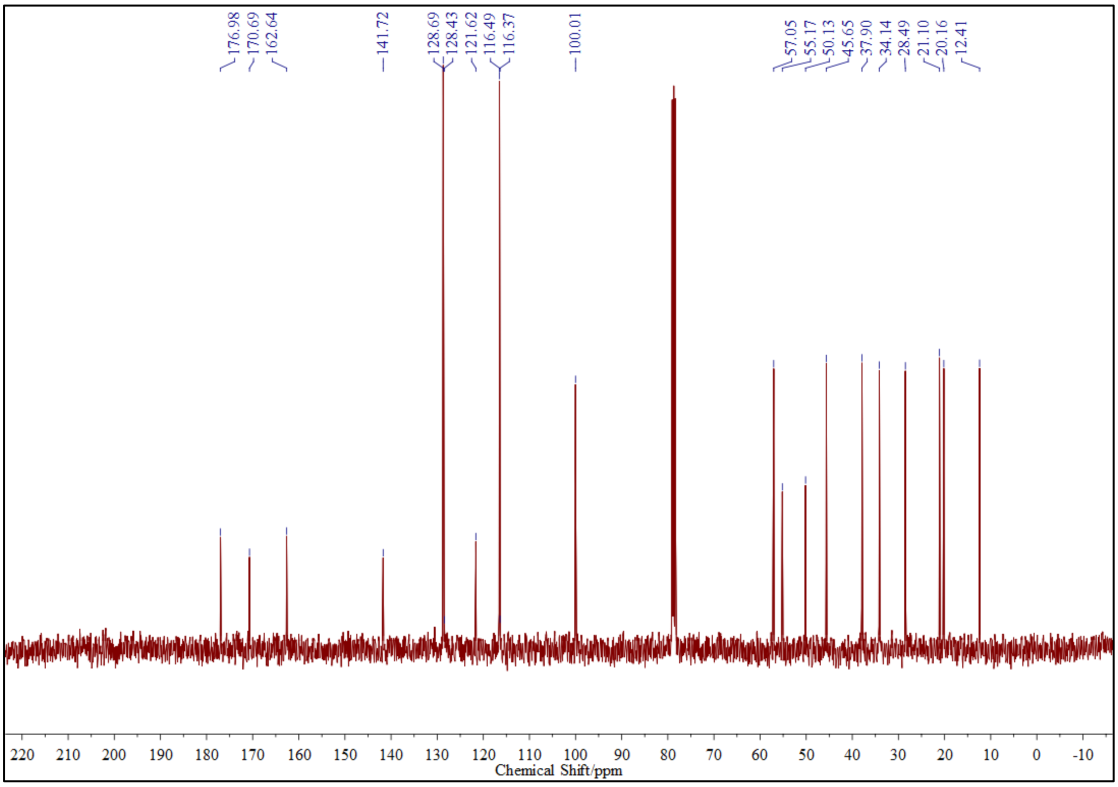

Figure S66. ^{13}C-NMR spectrum of compound 5k.

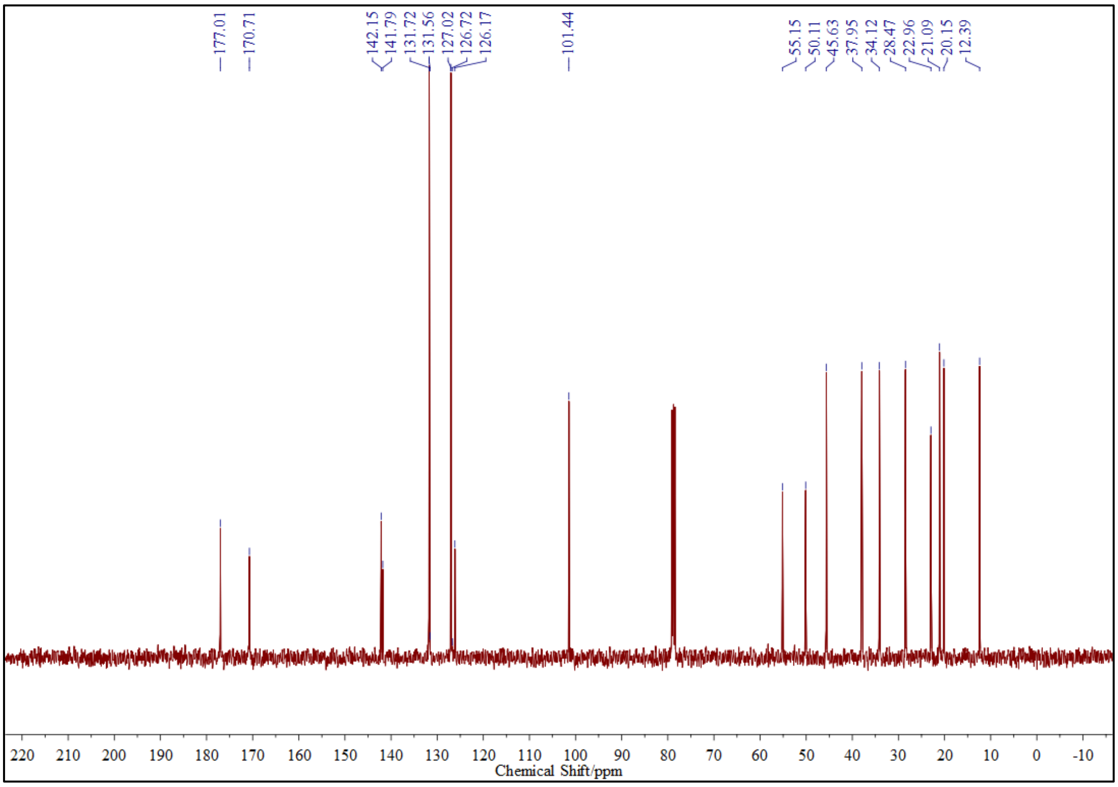

Figure S67. ^{13}C-NMR spectrum of compound 5l.

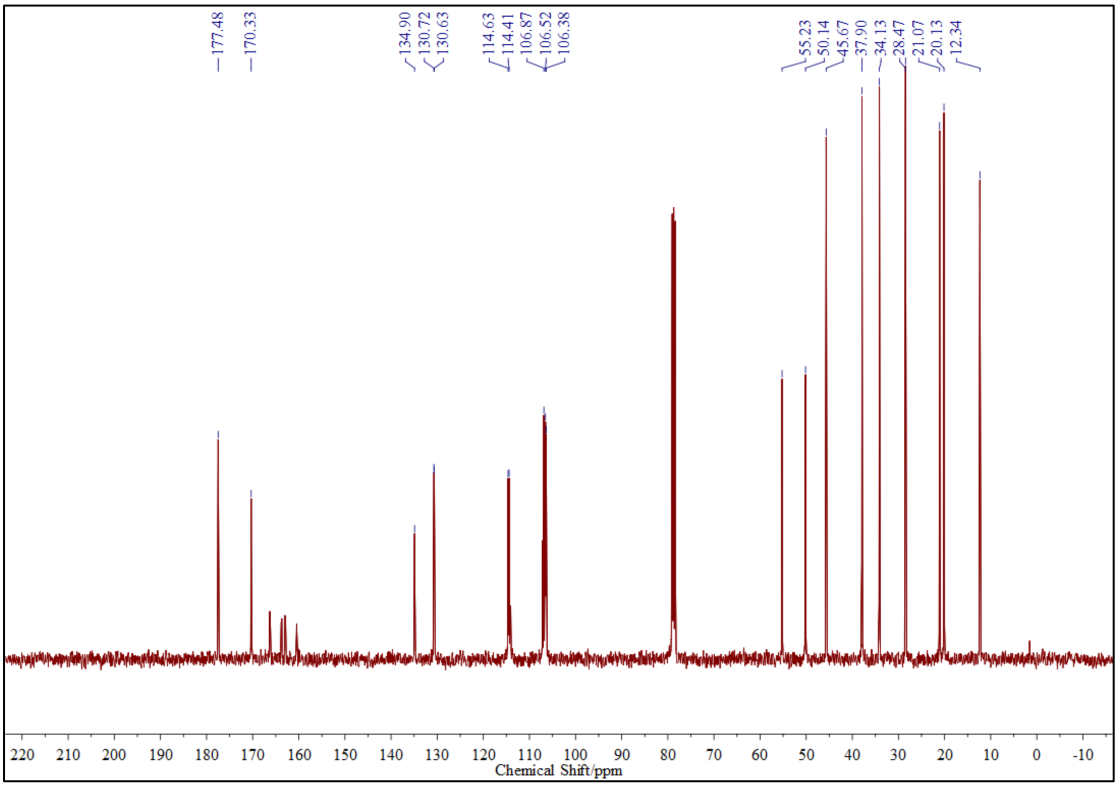

Figure S68. ^{13}C-NMR spectrum of compound 5m.

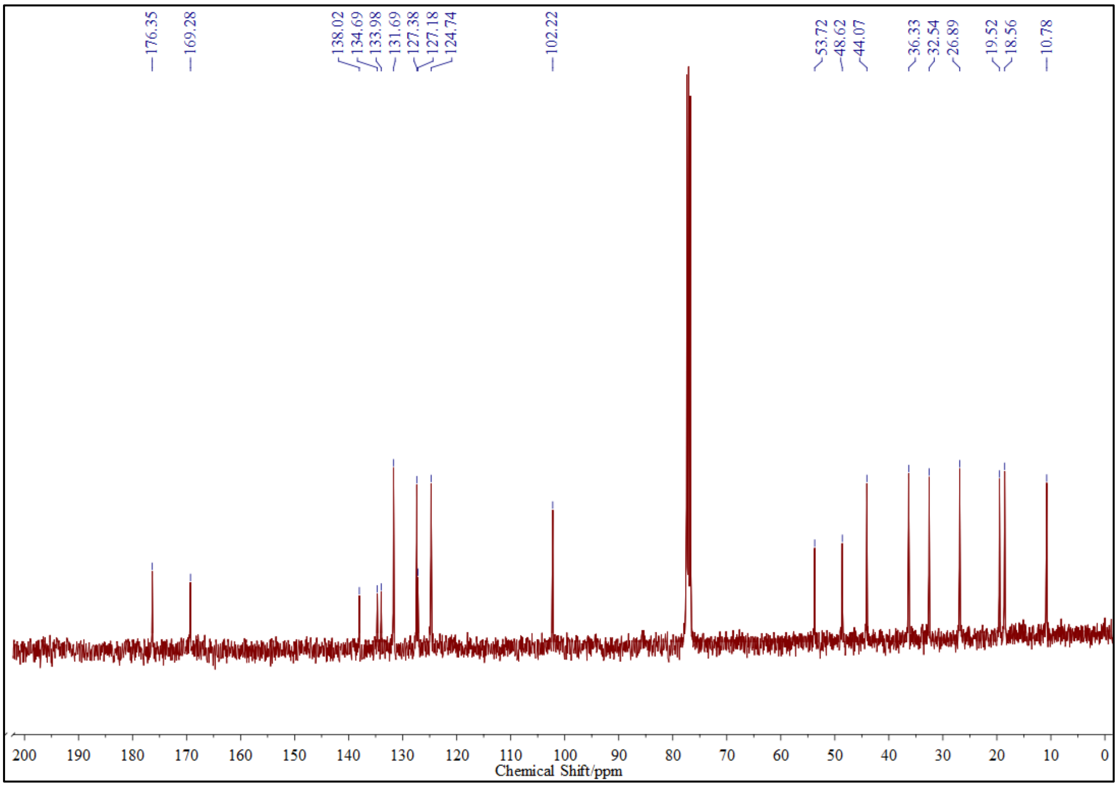

Figure S69. ^{13}C-NMR spectrum of compound 5n.

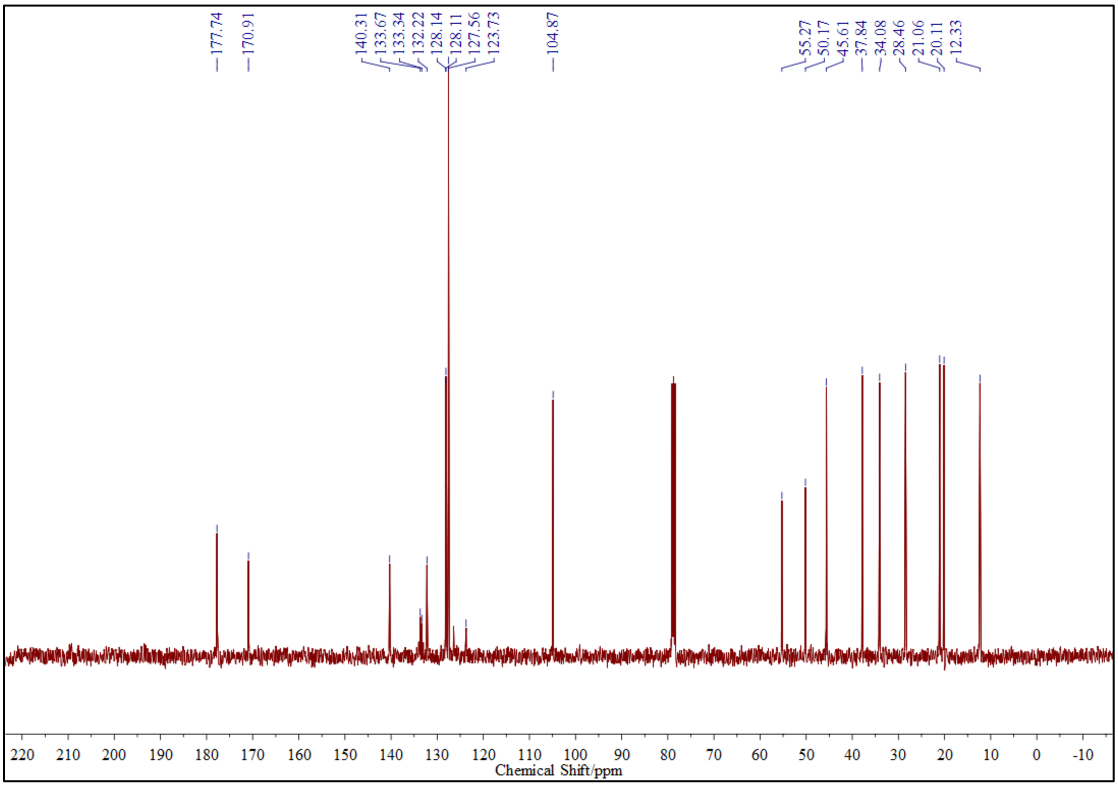

Figure S70. ^{13}C-NMR spectrum of compound 5o.

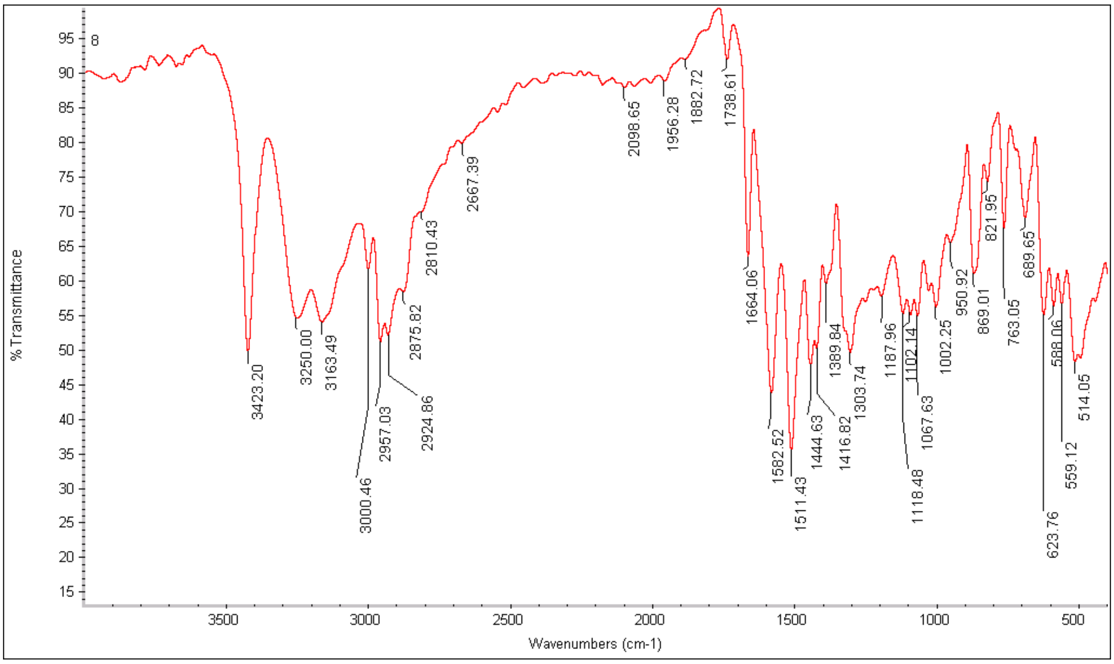

Figure S71. FT-IR spectrum of compound 3a.

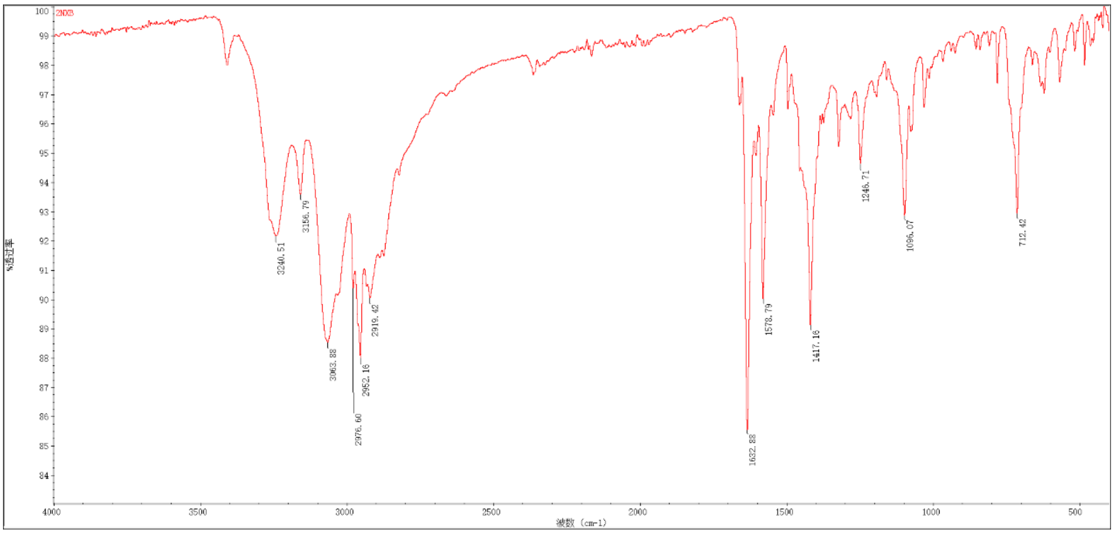

Figure S72. FT-IR spectrum of compound 4a.

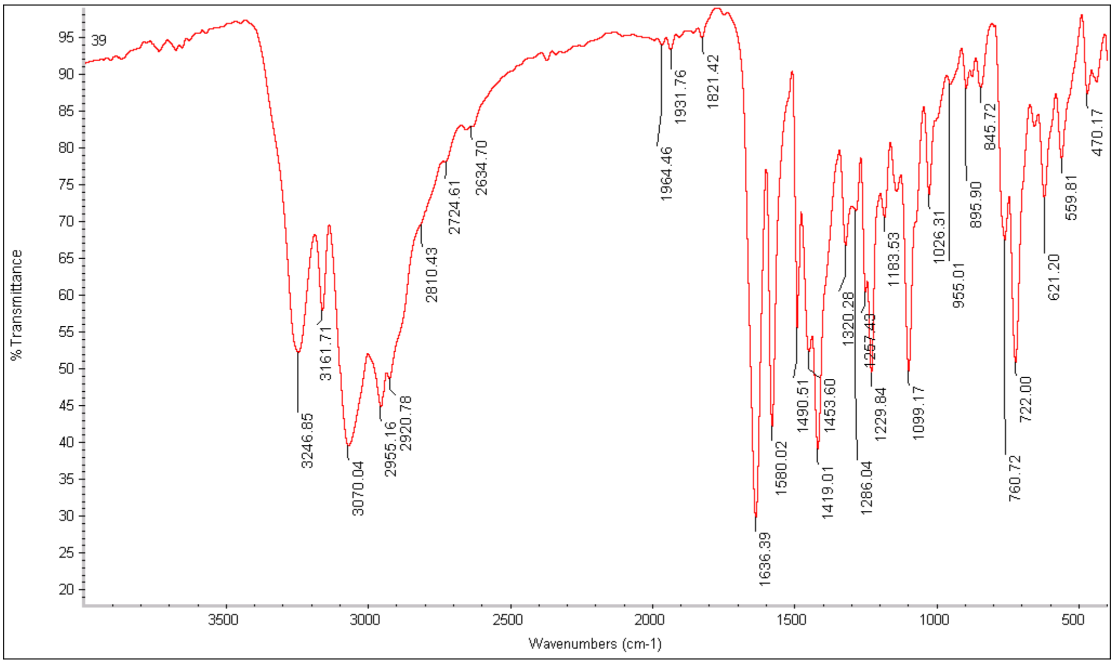

Figure S73. FT-IR spectrum of compound 4b.

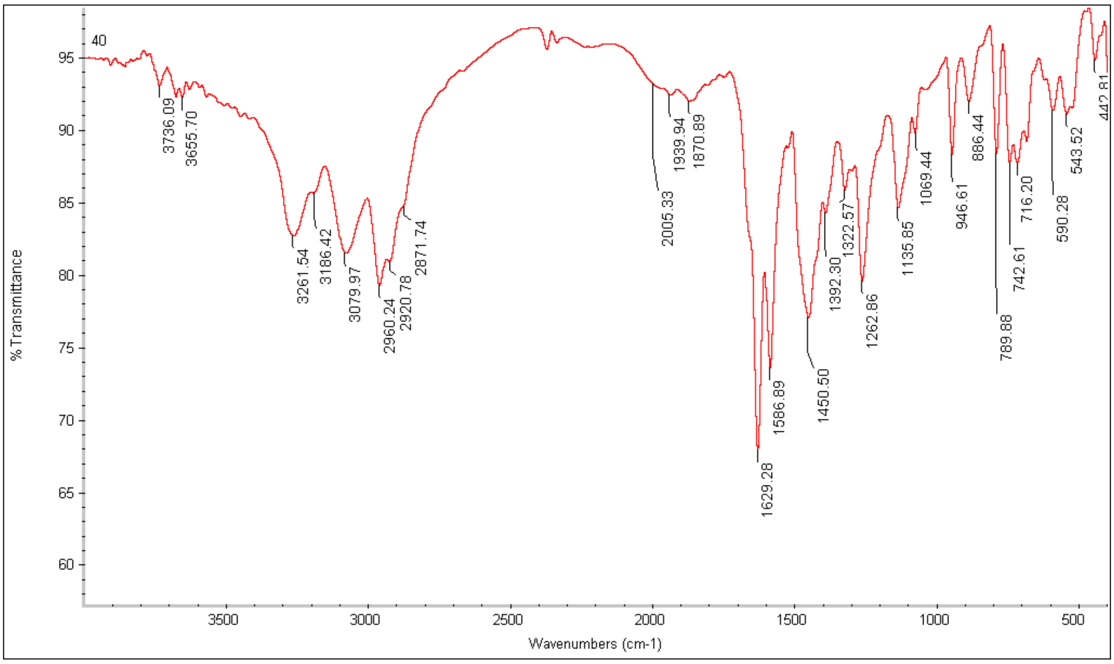

Figure S74. FT-IR spectrum of compound 4c.

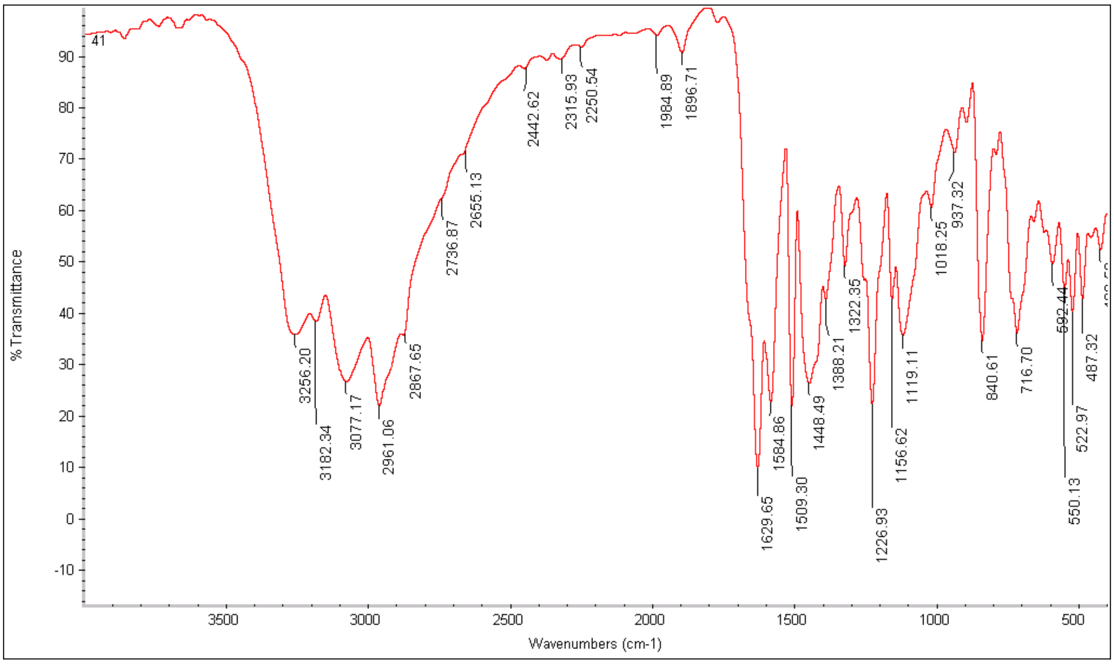

Figure S75. FT-IR spectrum of compound 4d.

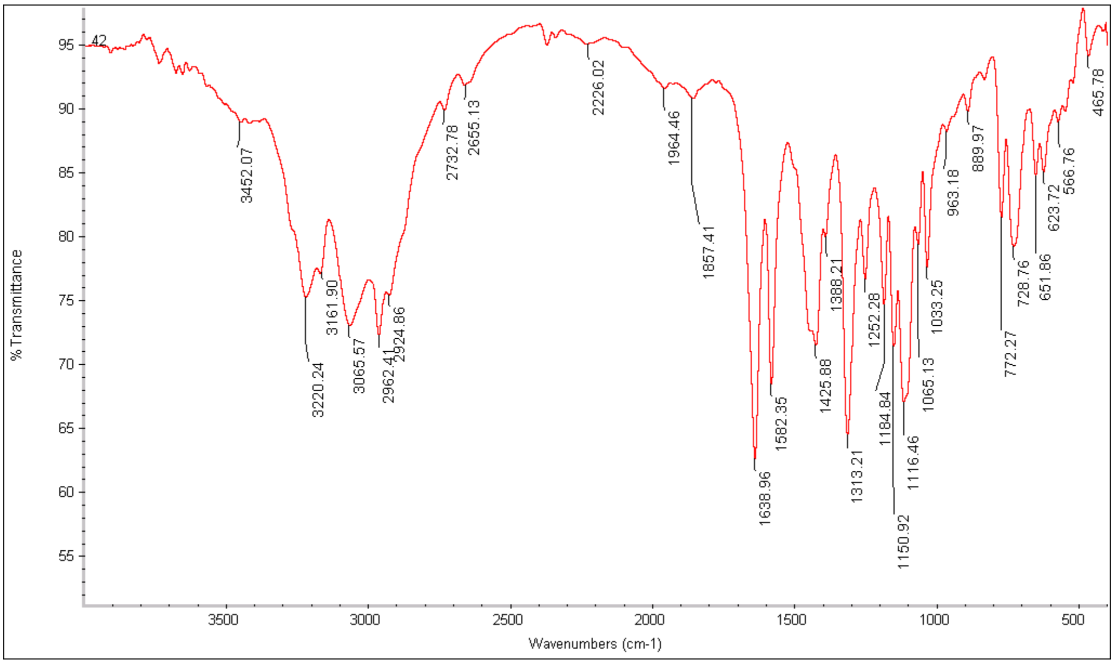

Figure S76. FT-IR spectrum of compound 4e.

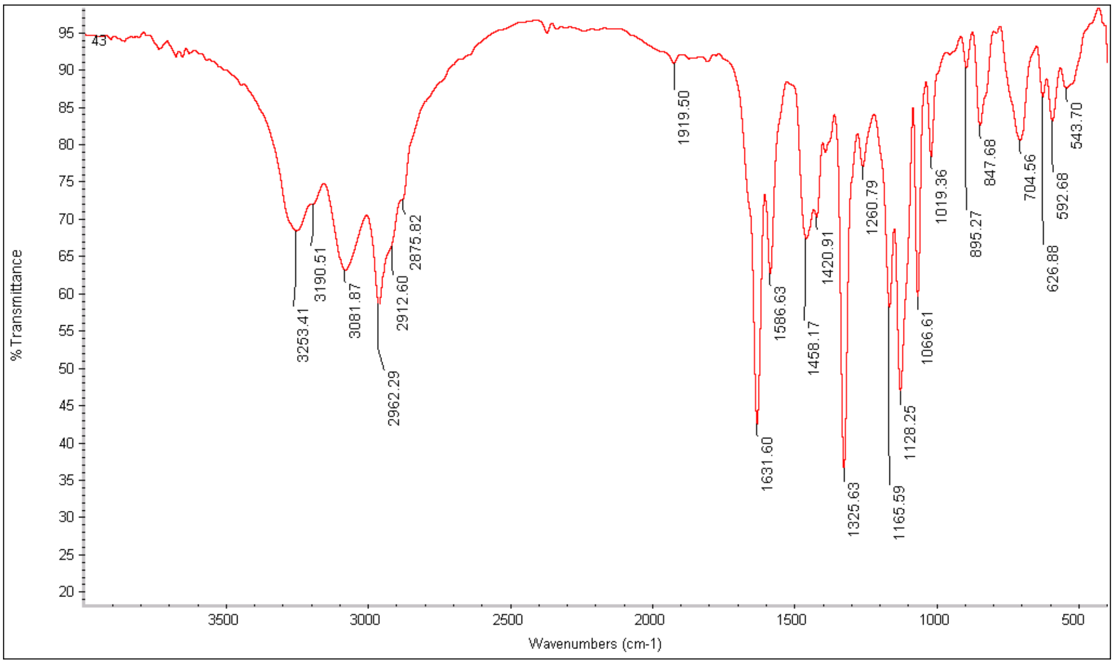

Figure S77. FT-IR spectrum of compound 4f.

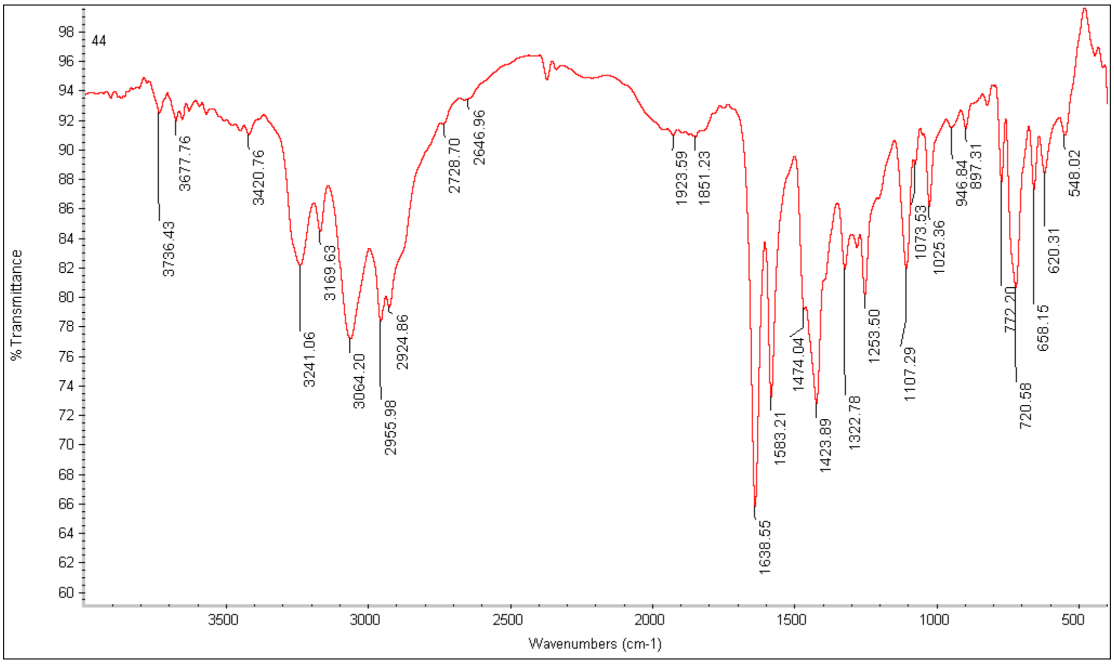

Figure S78. FT-IR spectrum of compound 4g.

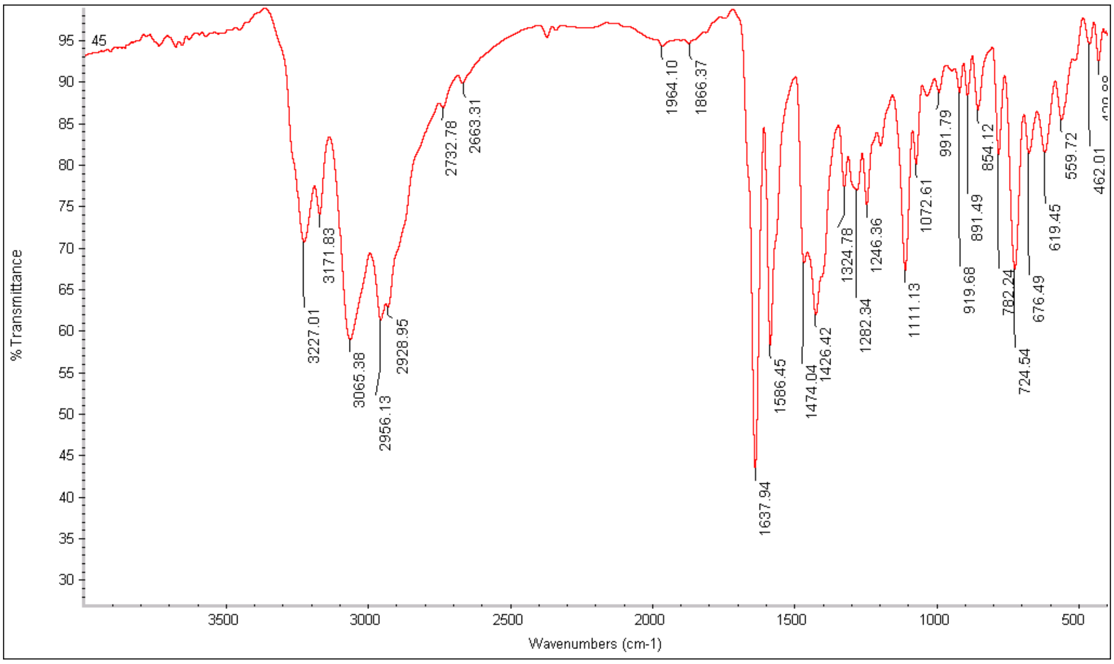

Figure S79. FT-IR spectrum of compound 4h.

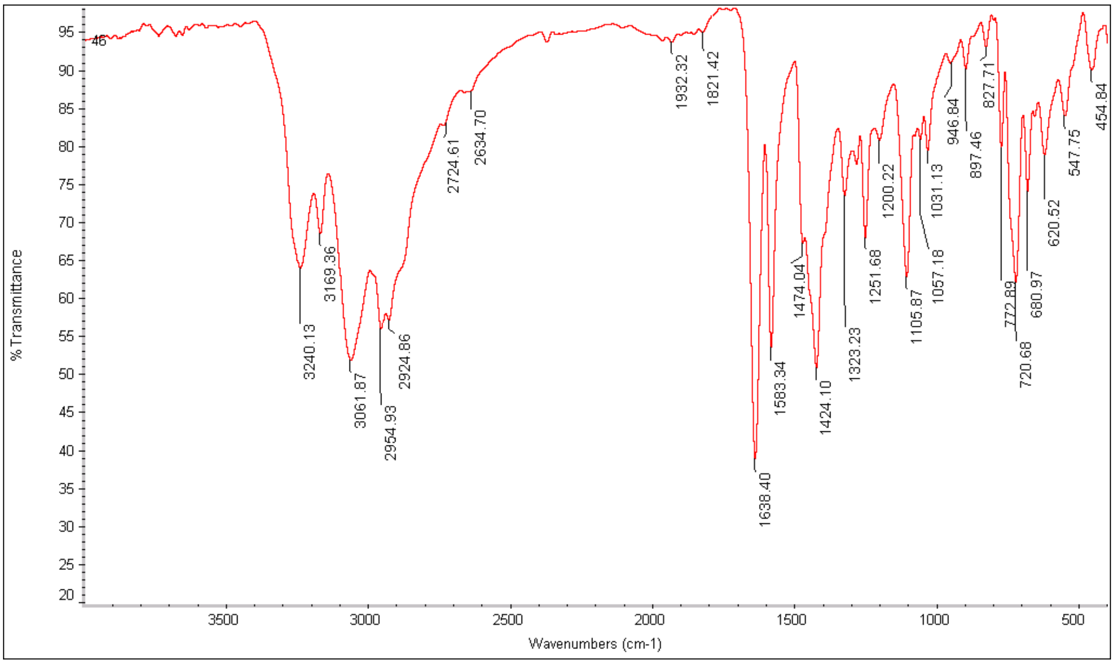

Figure S80. FT-IR spectrum of compound 4i.

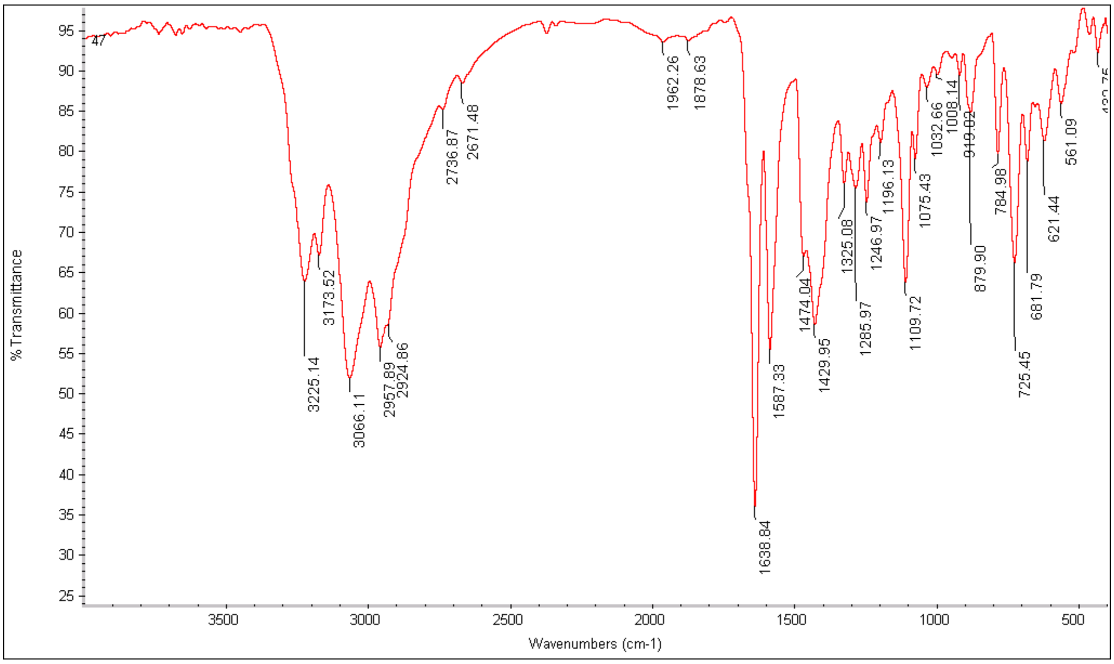

Figure S81. FT-IR spectrum of compound 4j.

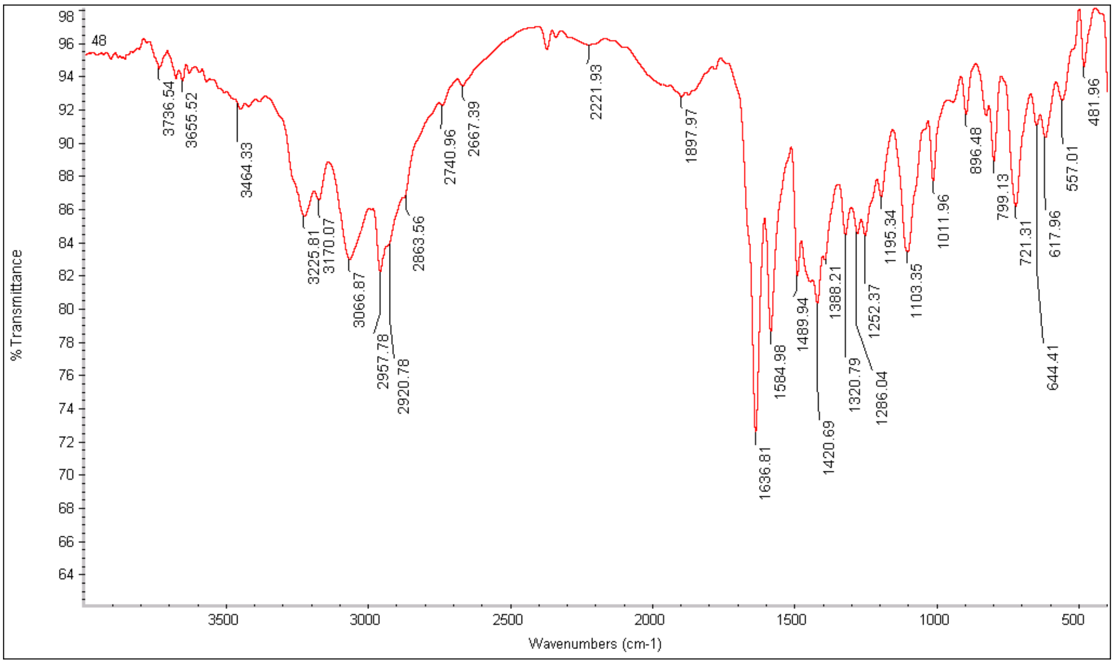

Figure S82. FT-IR spectrum of compound 4k.

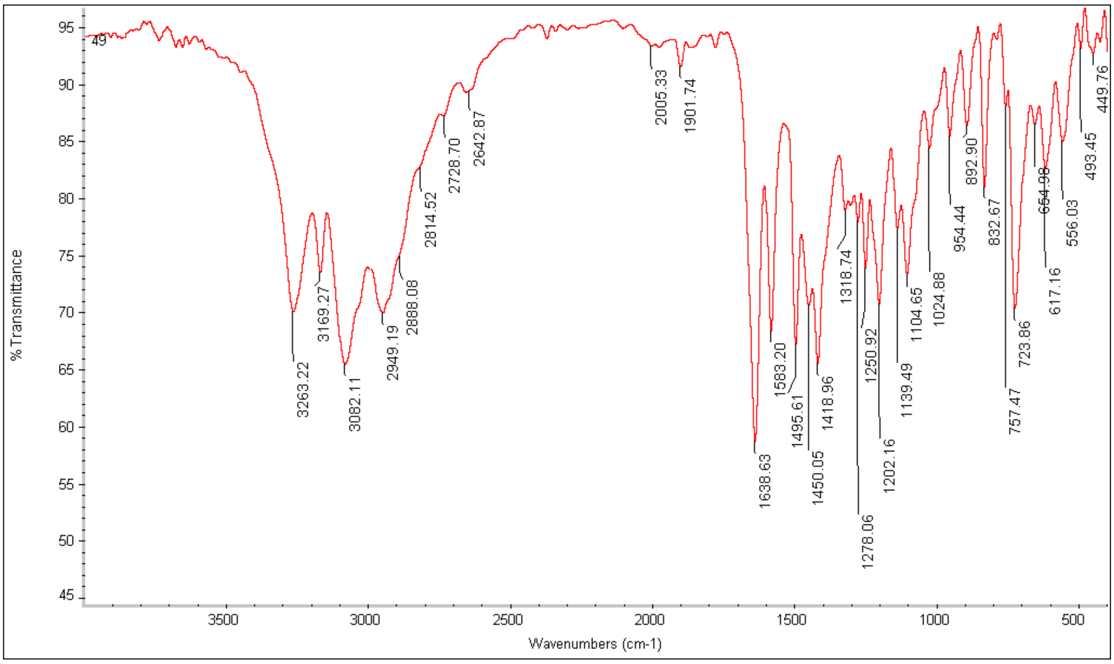

Figure S83. FT-IR spectrum of compound 4l.

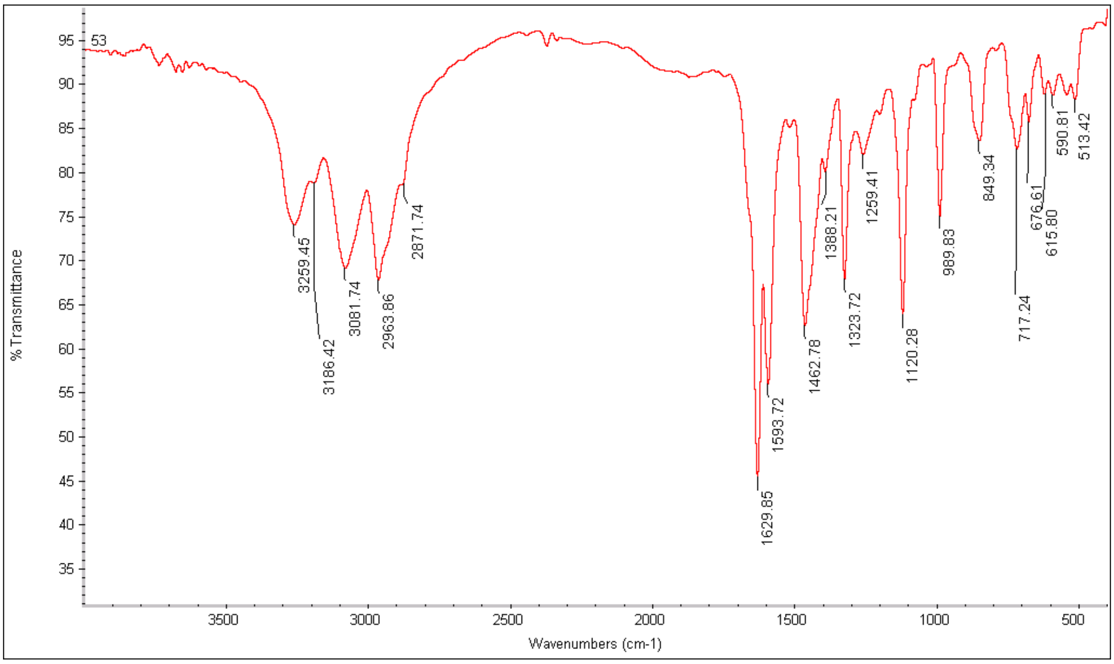

Figure S84. FT-IR spectrum of compound 4m.

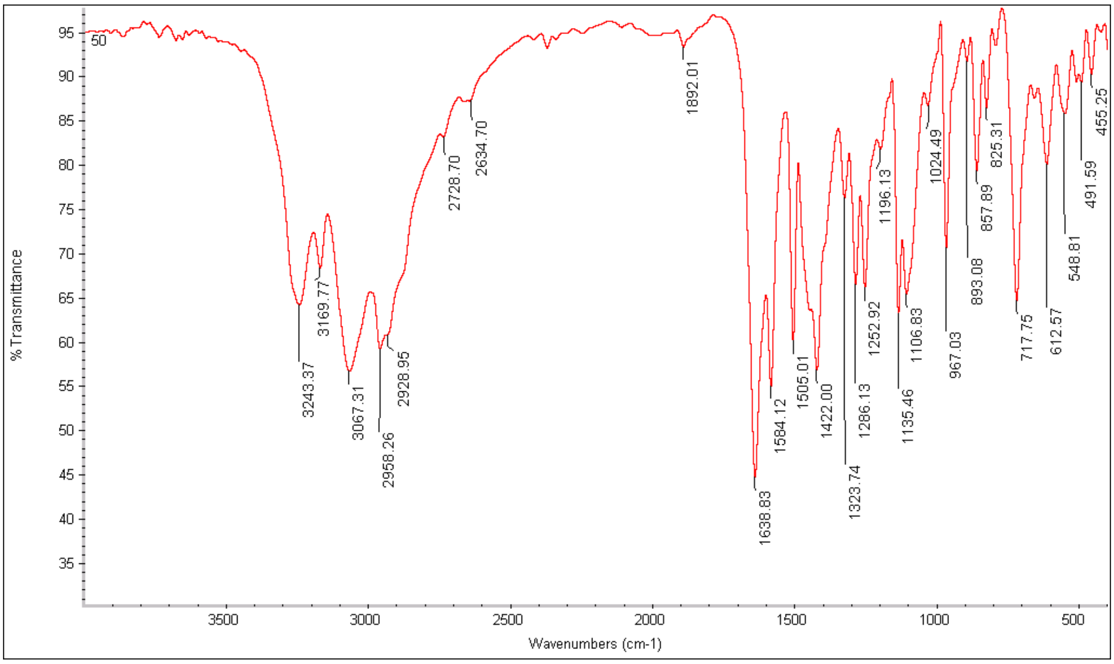

Figure S85. FT-IR spectrum of compound 4n.

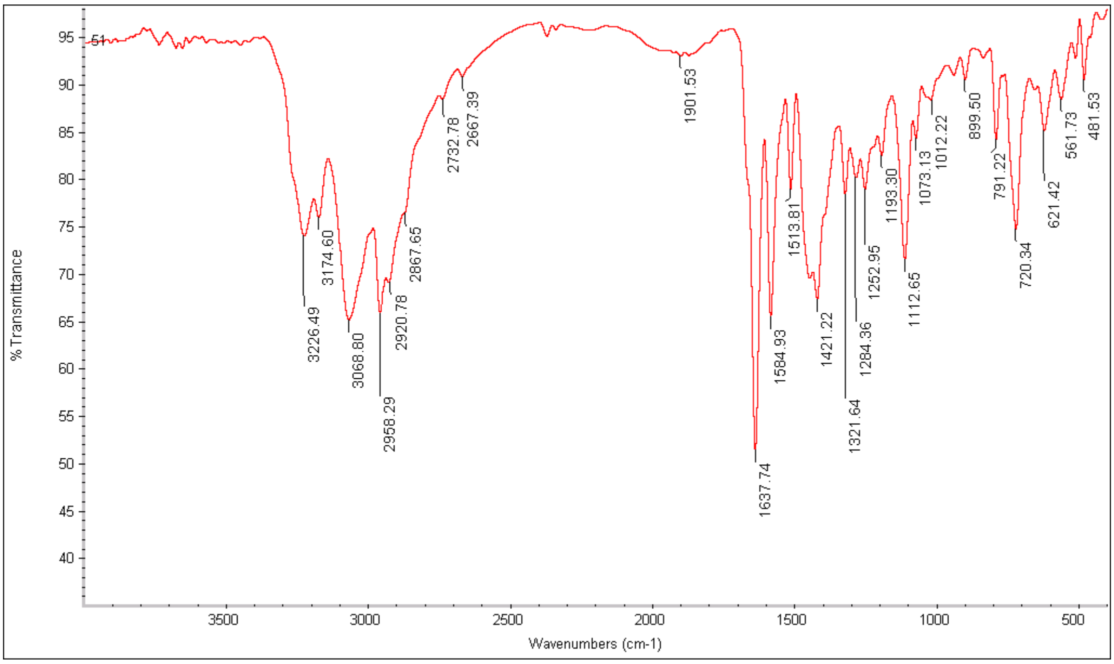

Figure S86. FT-IR spectrum of compound 4o.

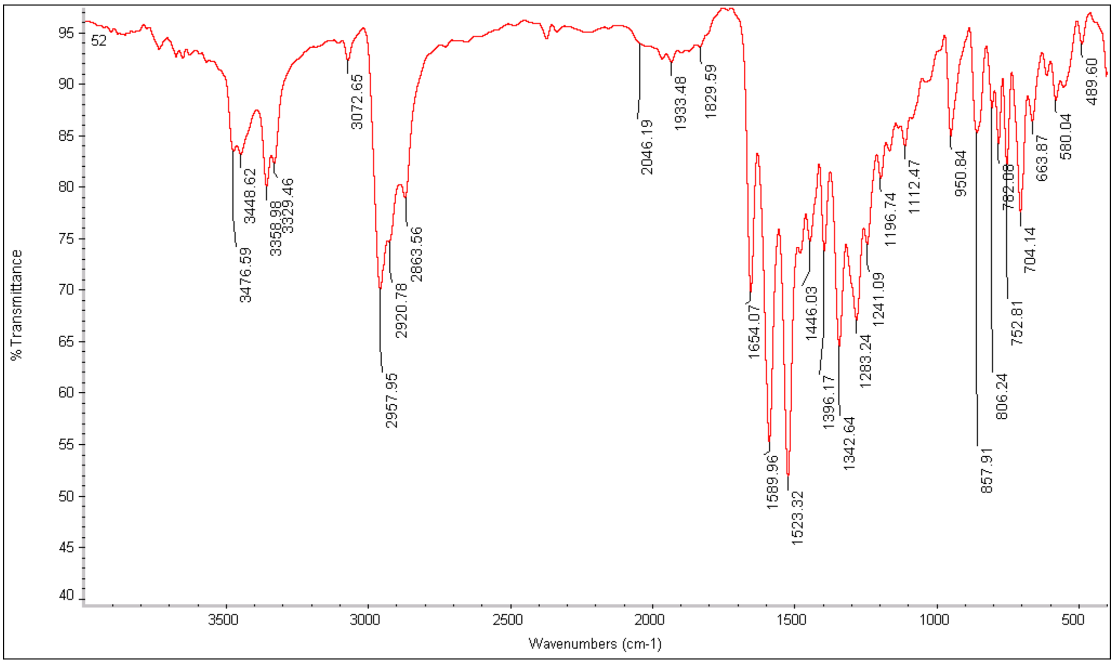

Figure S87. FT-IR spectrum of compound 4p.

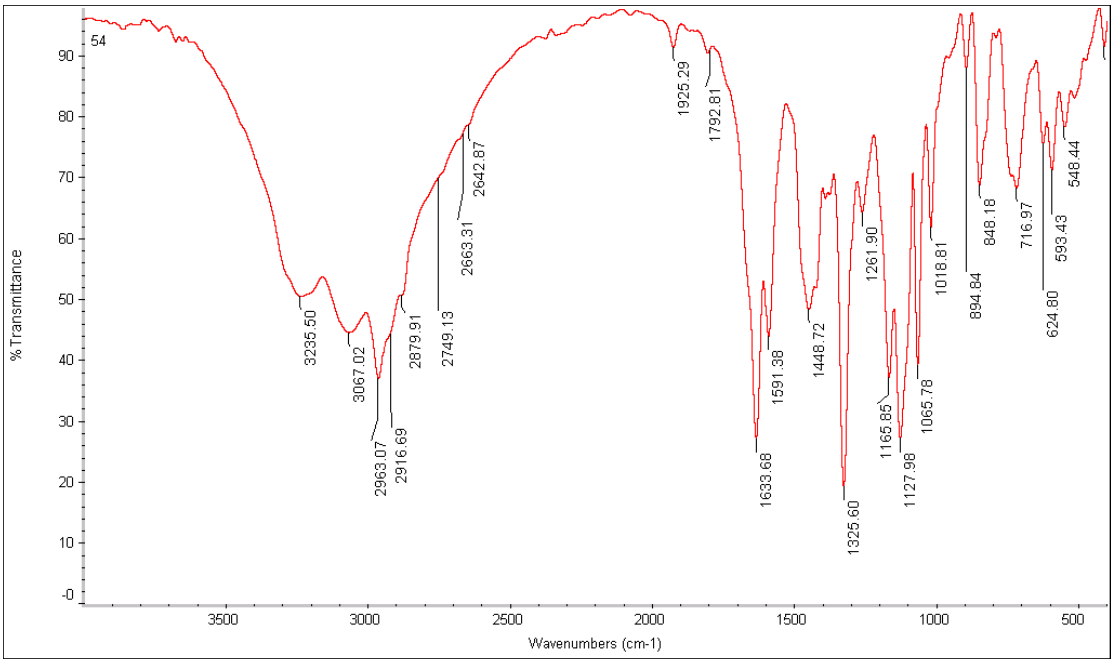

Figure S88. FT-IR spectrum of compound 4q.

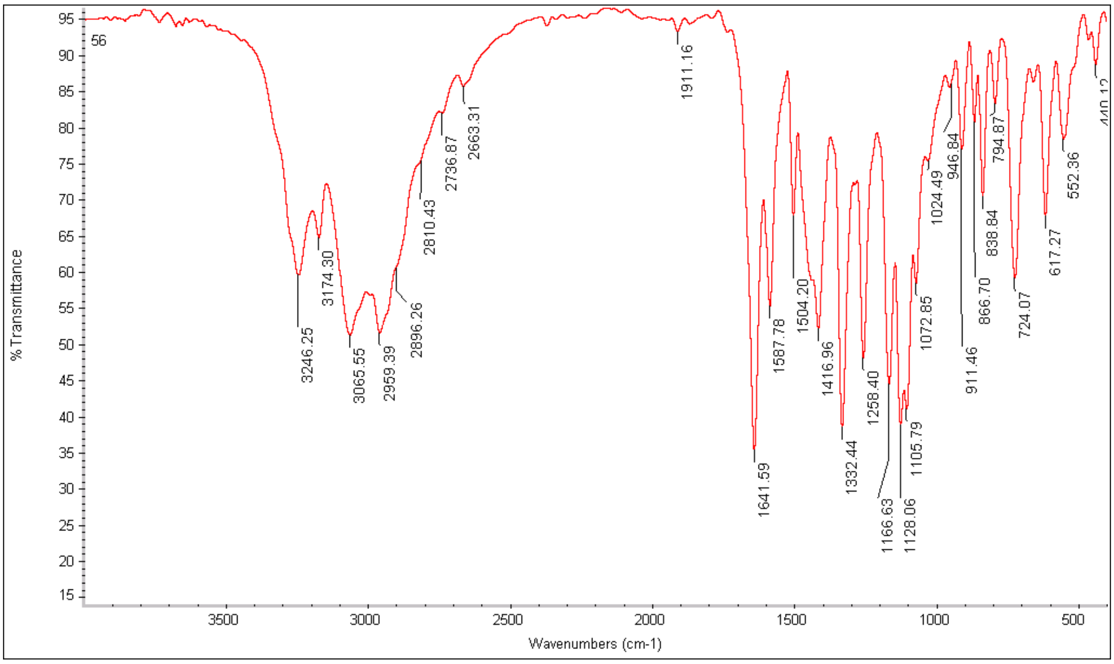

Figure S89. FT-IR spectrum of compound 4r.

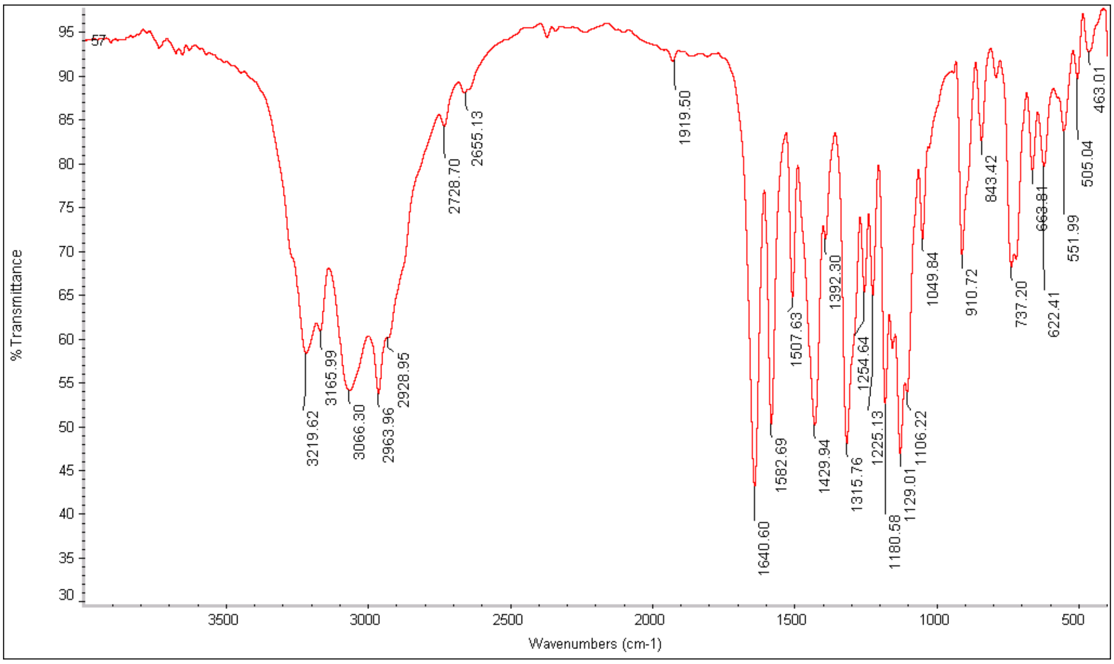

Figure S90. FT-IR spectrum of compound 4s.

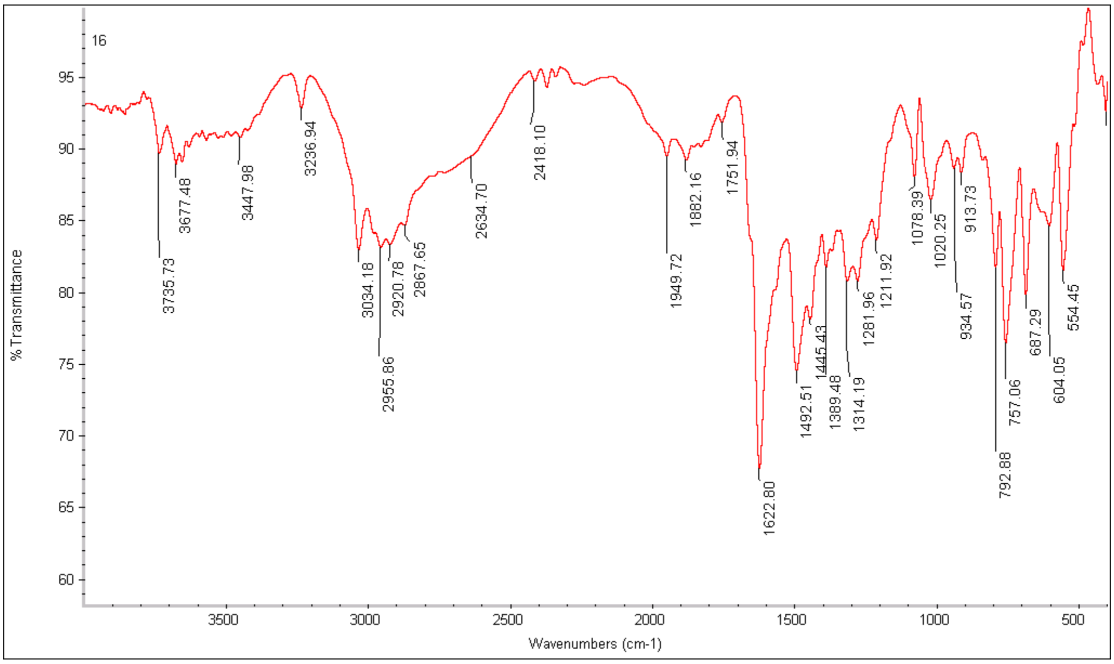

Figure S91. FT-IR spectrum of compound 5a.

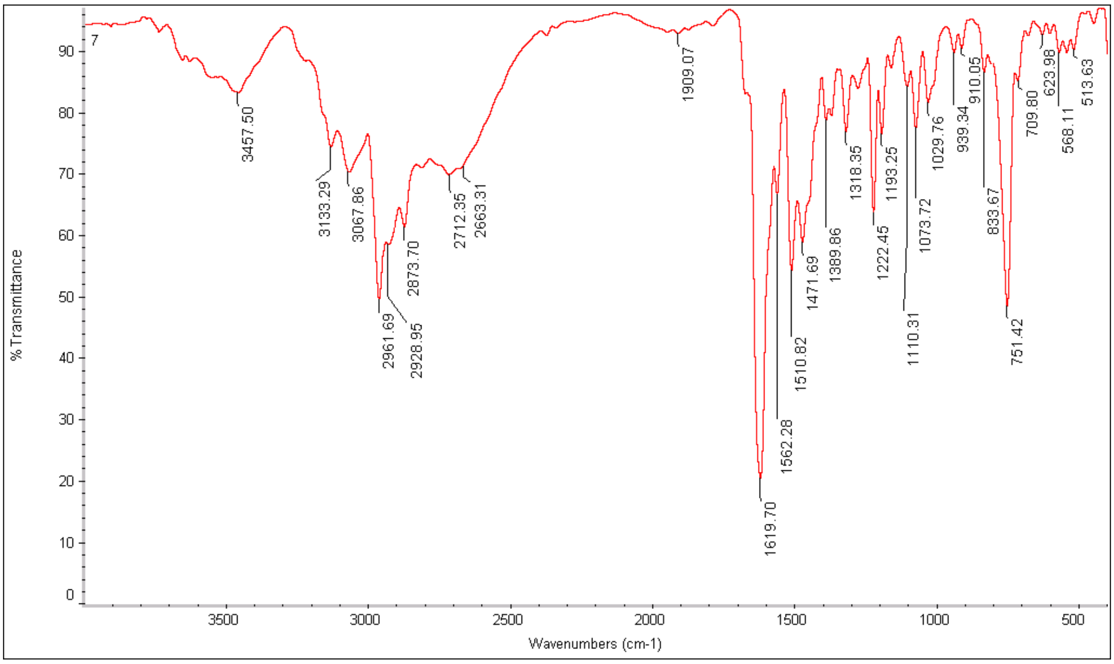

Figure S92. FT-IR spectrum of compound 5b.

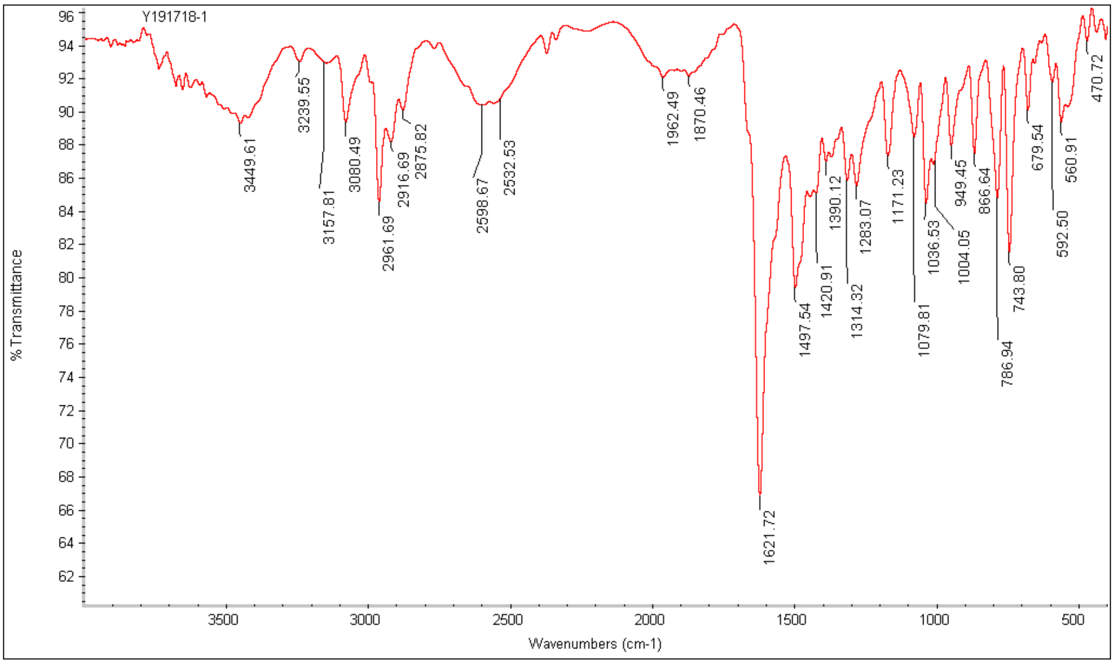

Figure S93. FT-IR spectrum of compound 5c.

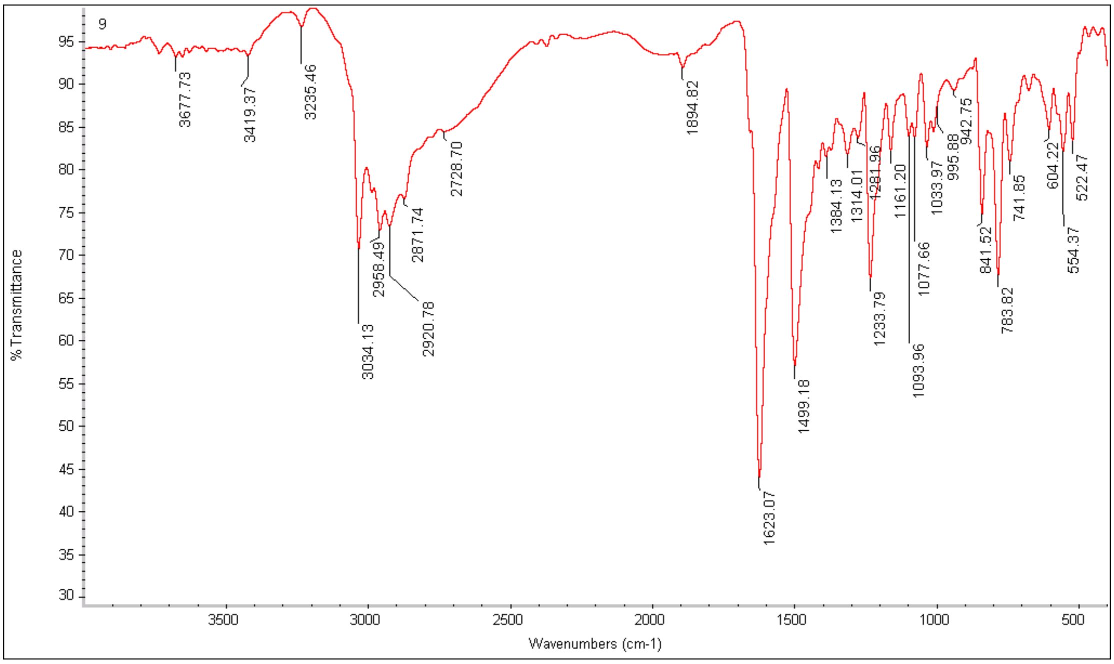

Figure S94. FT-IR spectrum of compound 5d.

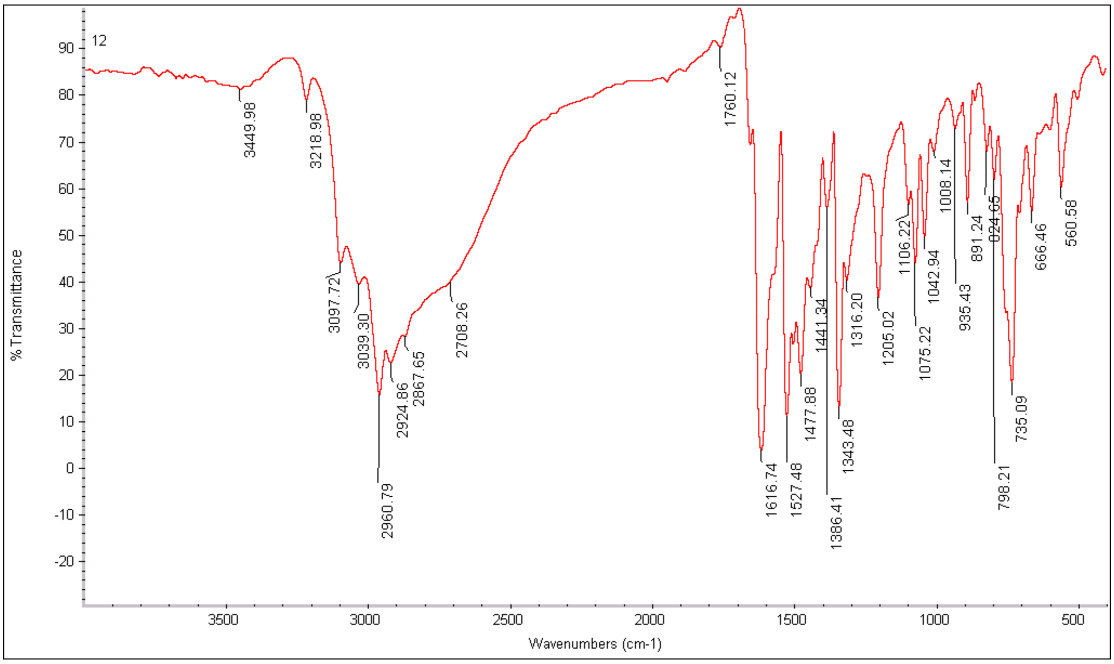

Figure S95. FT-IR spectrum of compound 5e.

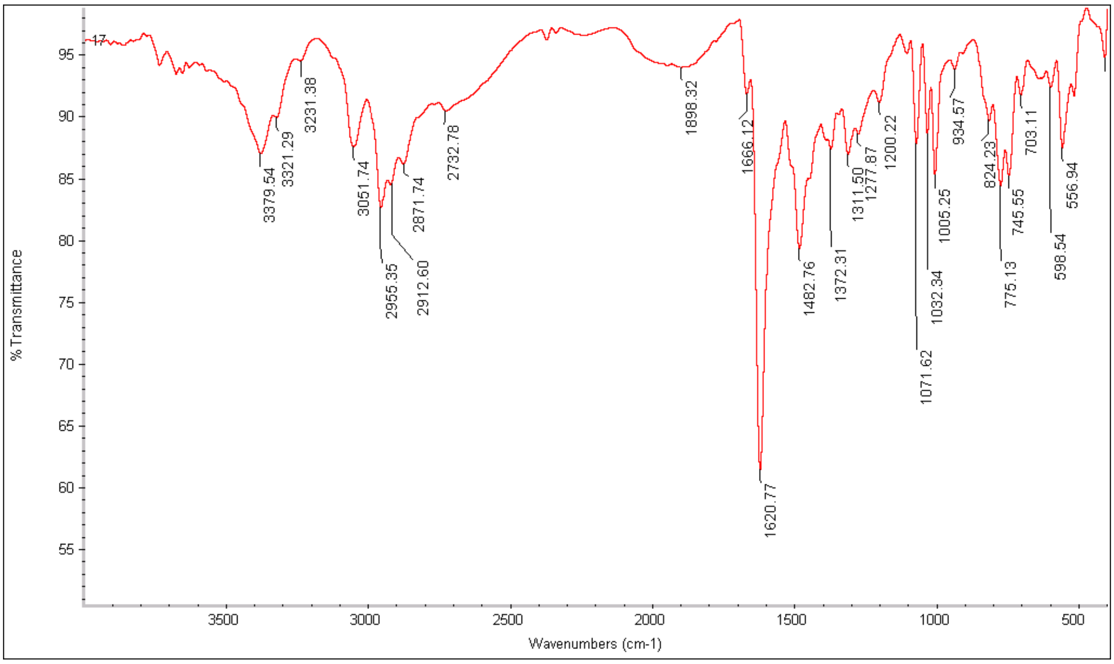

Figure S96. FT-IR spectrum of compound 5f.

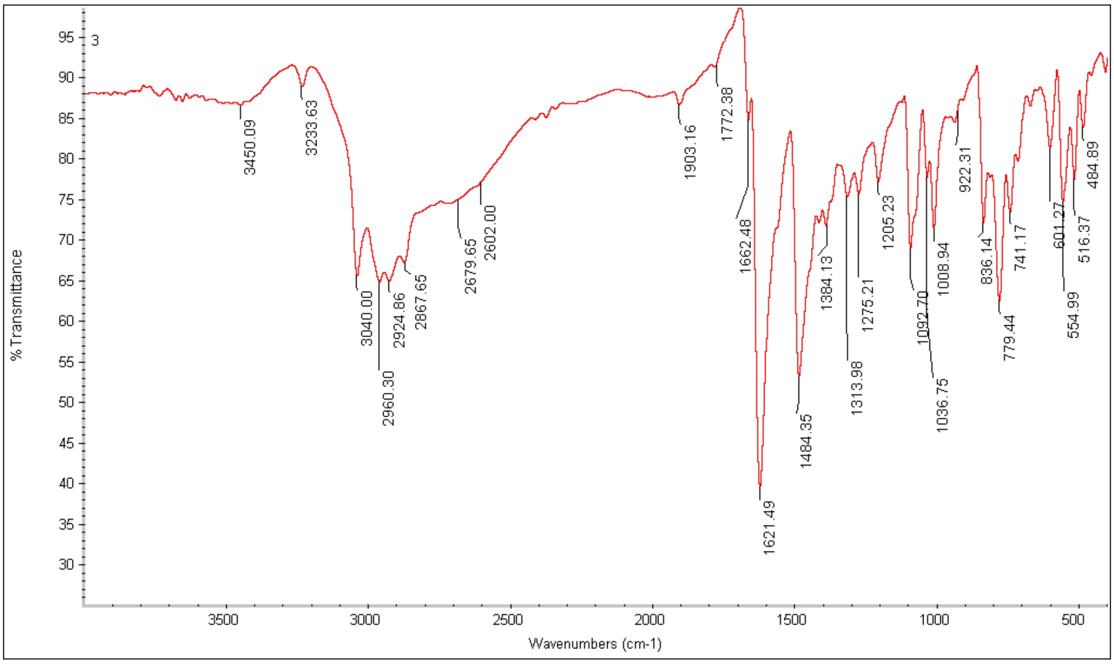

Figure S97. FT-IR spectrum of compound 5g.

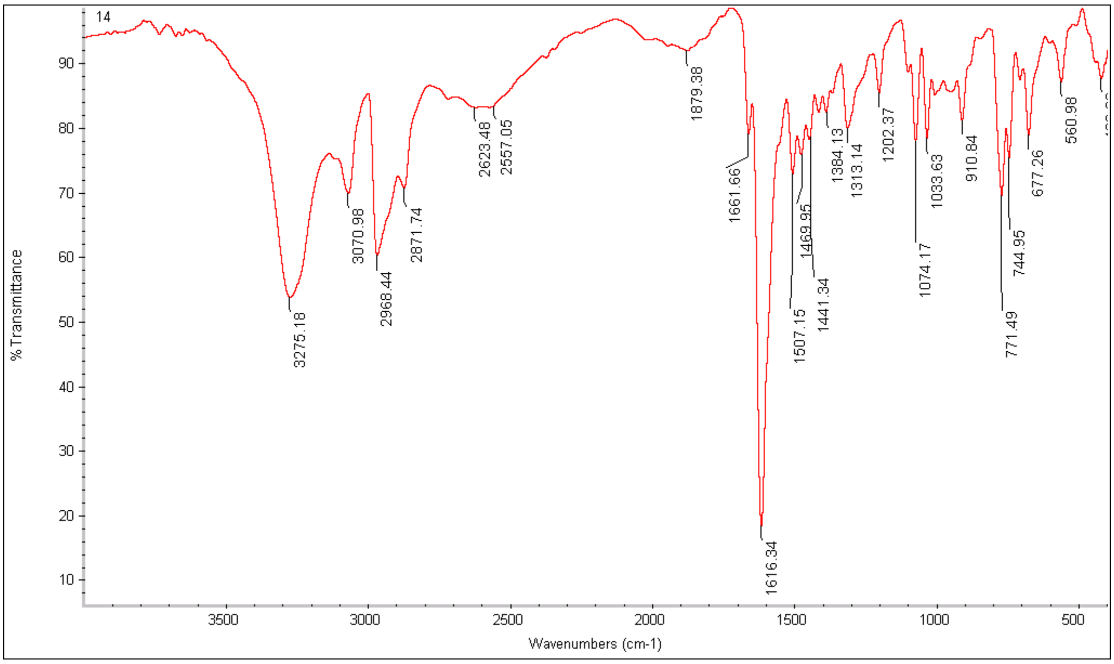

Figure S98. FT-IR spectrum of compound 5h.

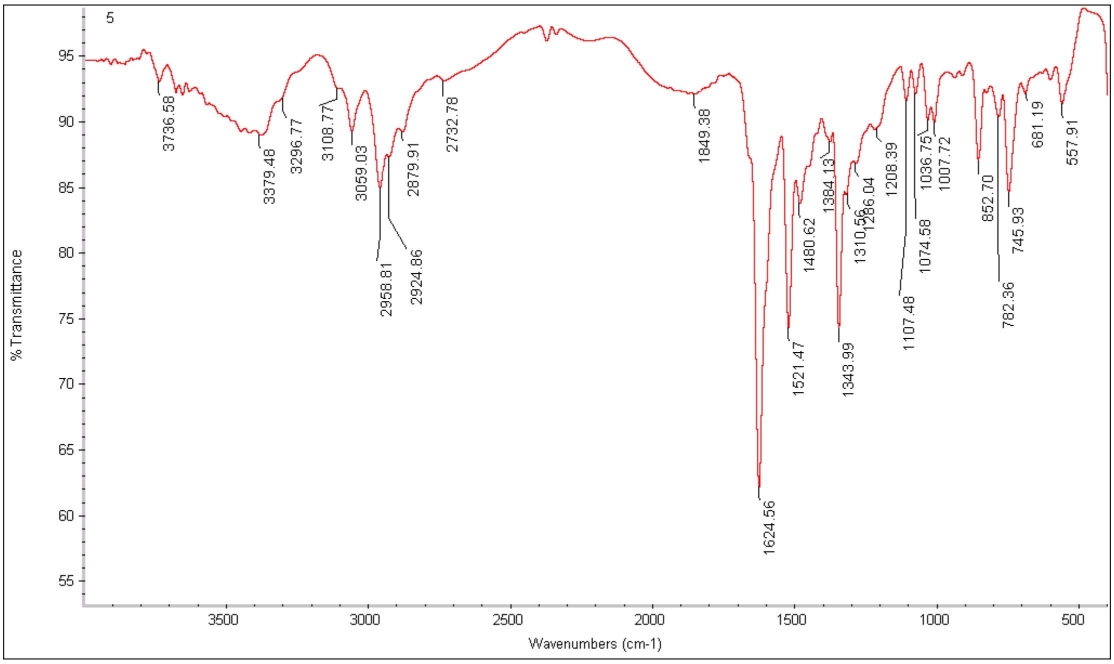

Figure S99. FT-IR spectrum of compound 5i.

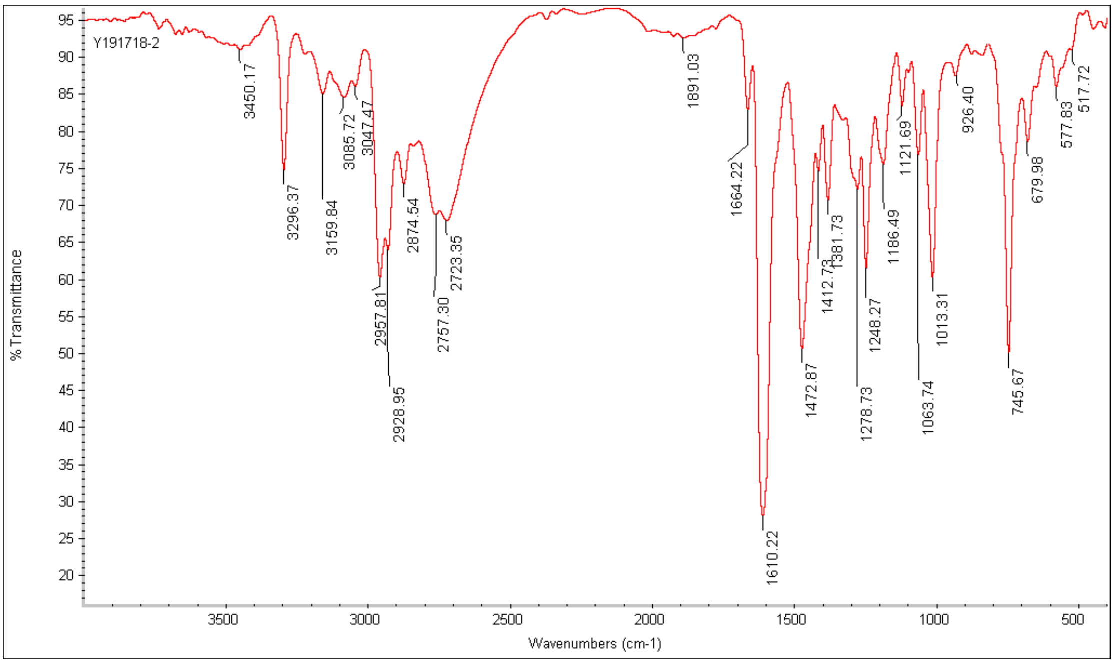

Figure S100. FT-IR spectrum of compound 5j.

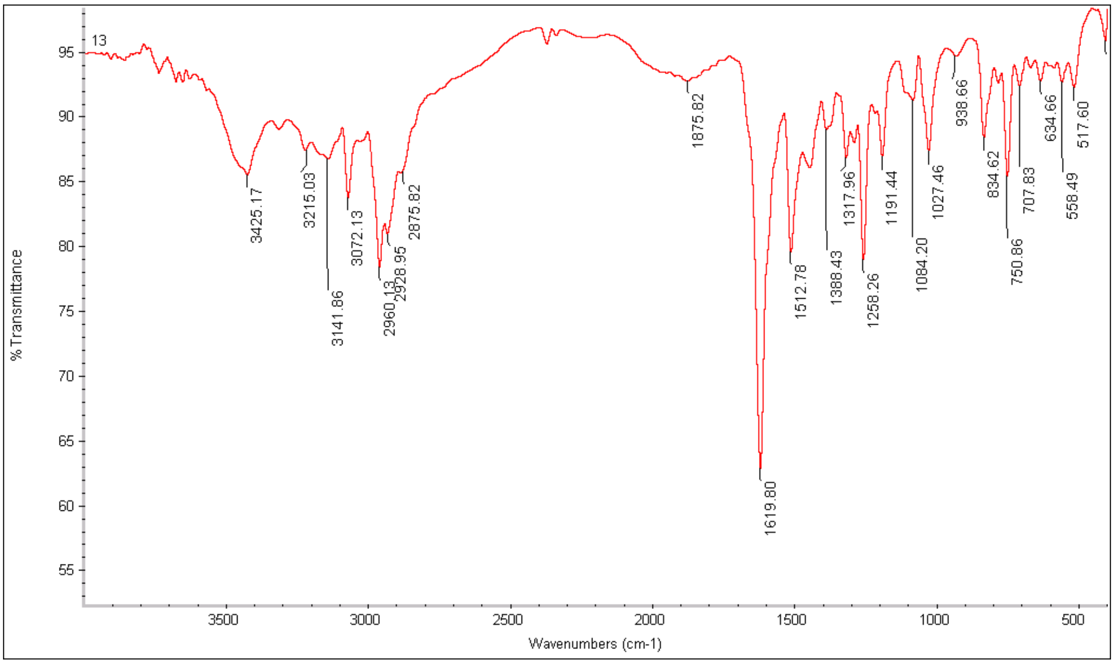

Figure S101. FT-IR spectrum of compound 5k.

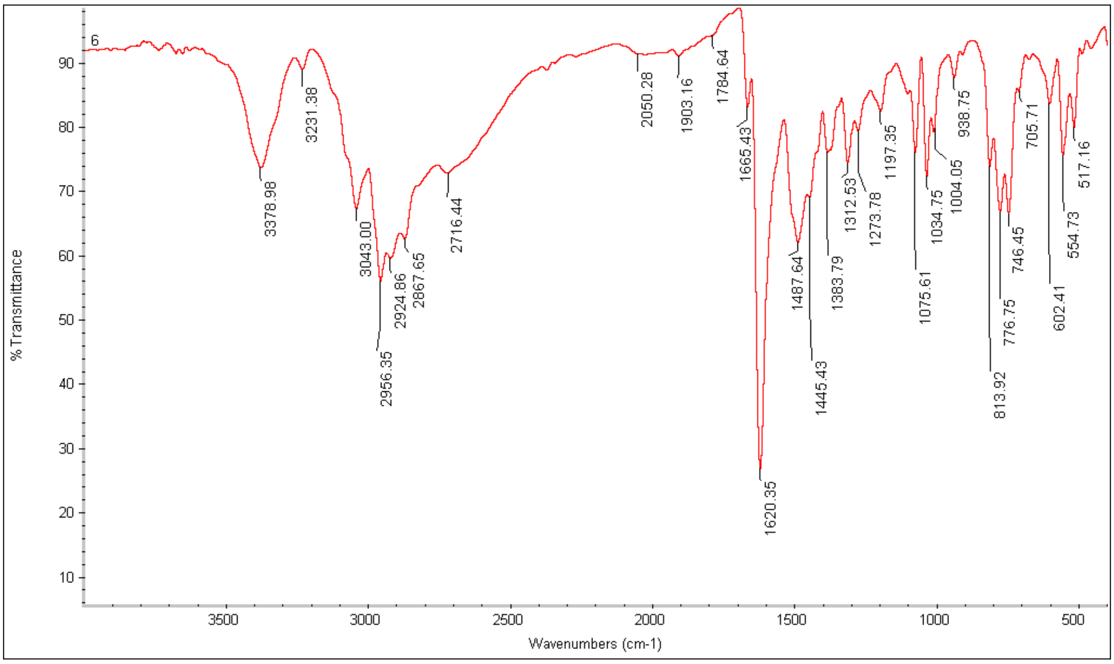

Figure S102. FT-IR spectrum of compound 5l.

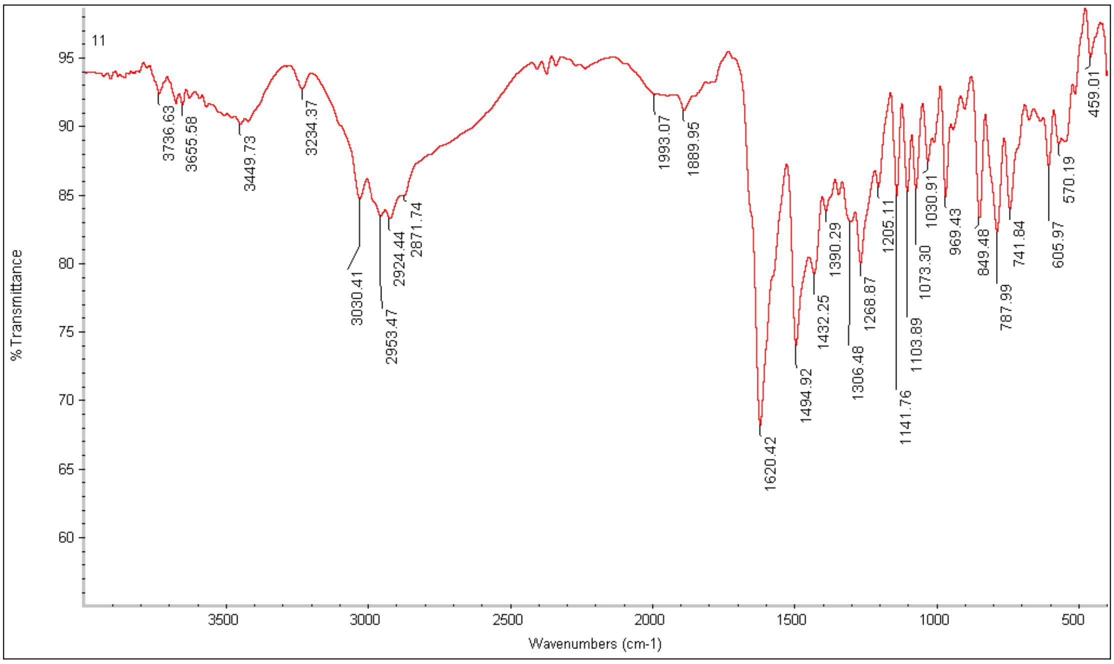

Figure S103. FT-IR spectrum of compound 5m.

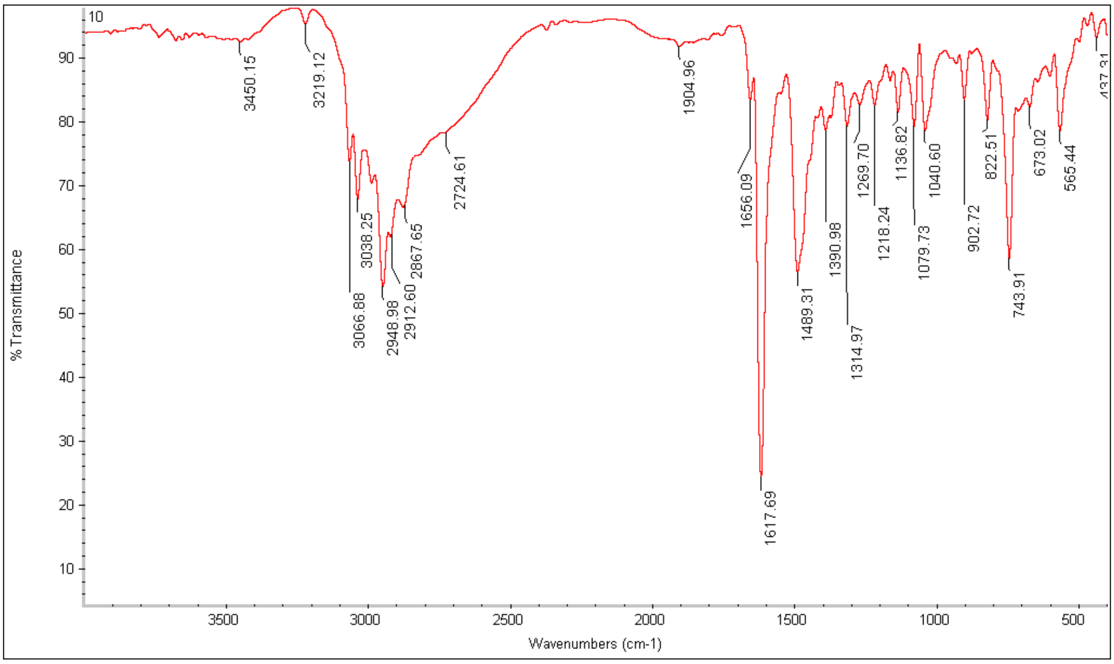

Figure S104. FT-IR spectrum of compound 5n.

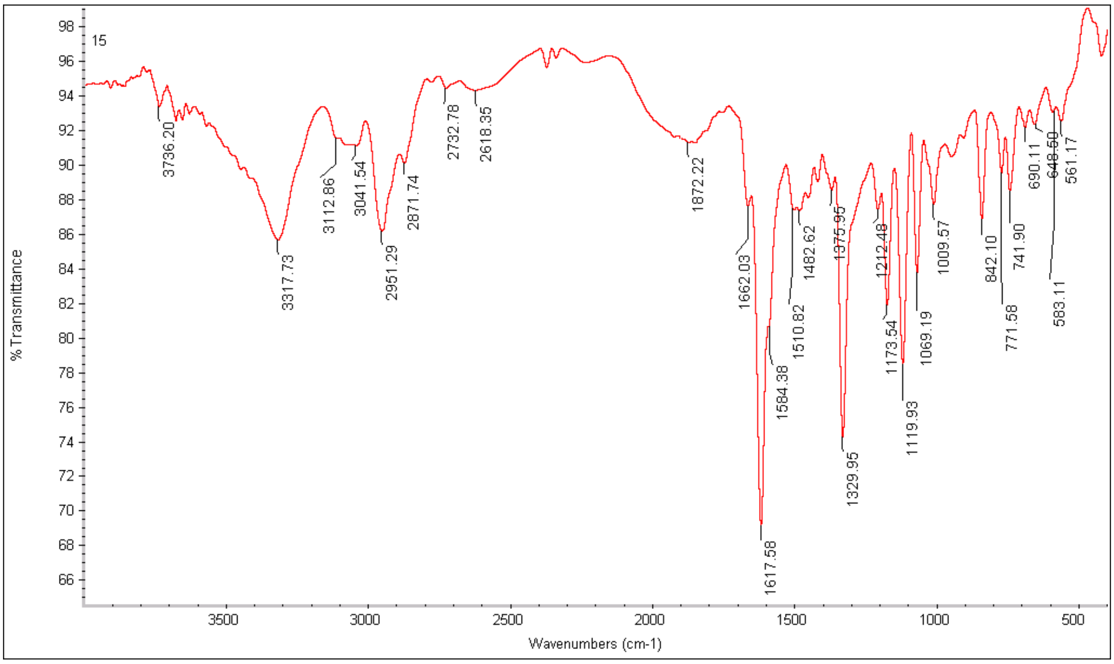

Figure S105. FT-IR spectrum of compound 5o.

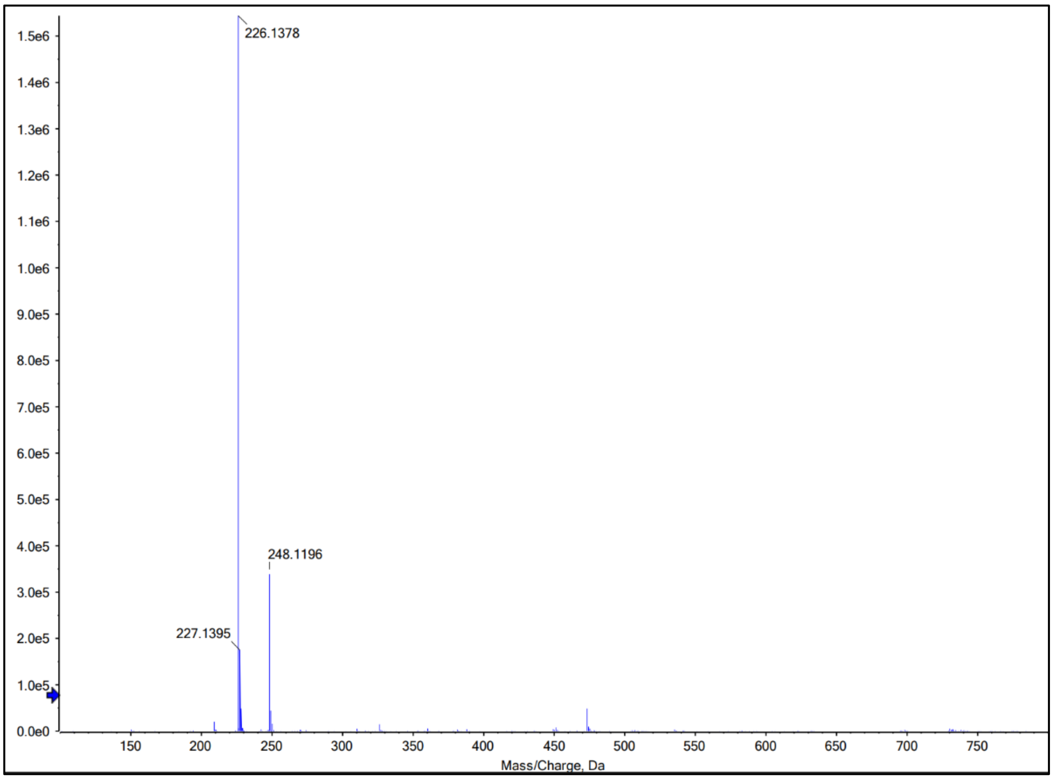

Figure S106. HRMS spectrum of compound 3a.

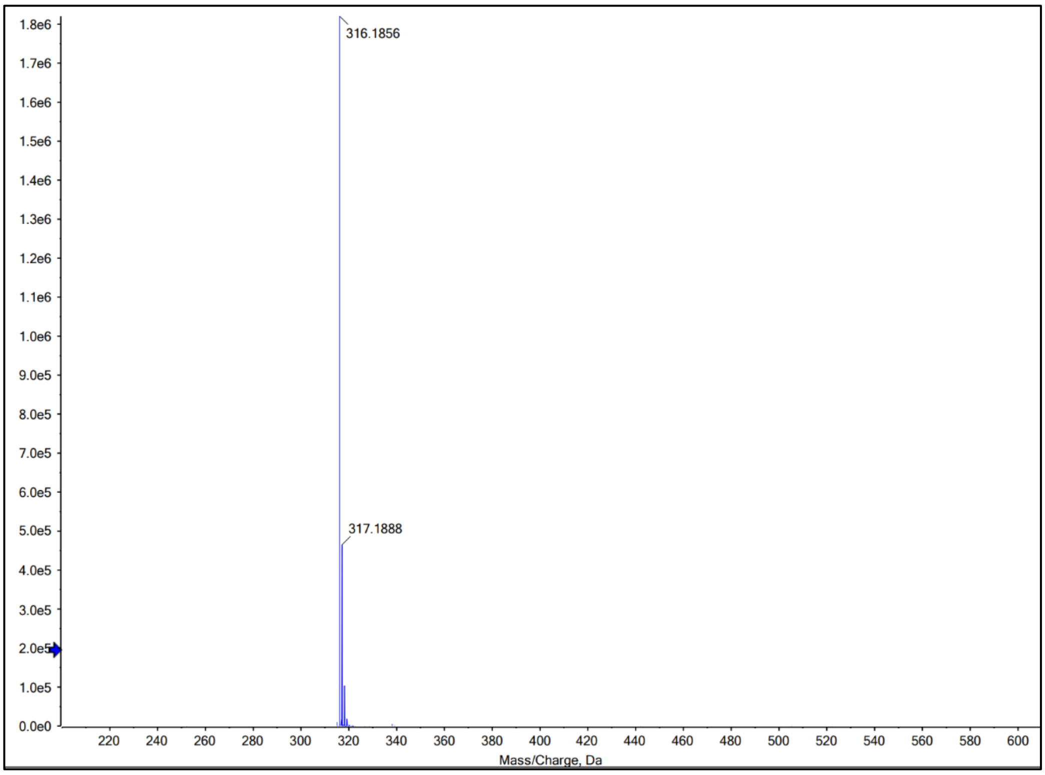

Figure S107. HRMS spectrum of compound 4a.

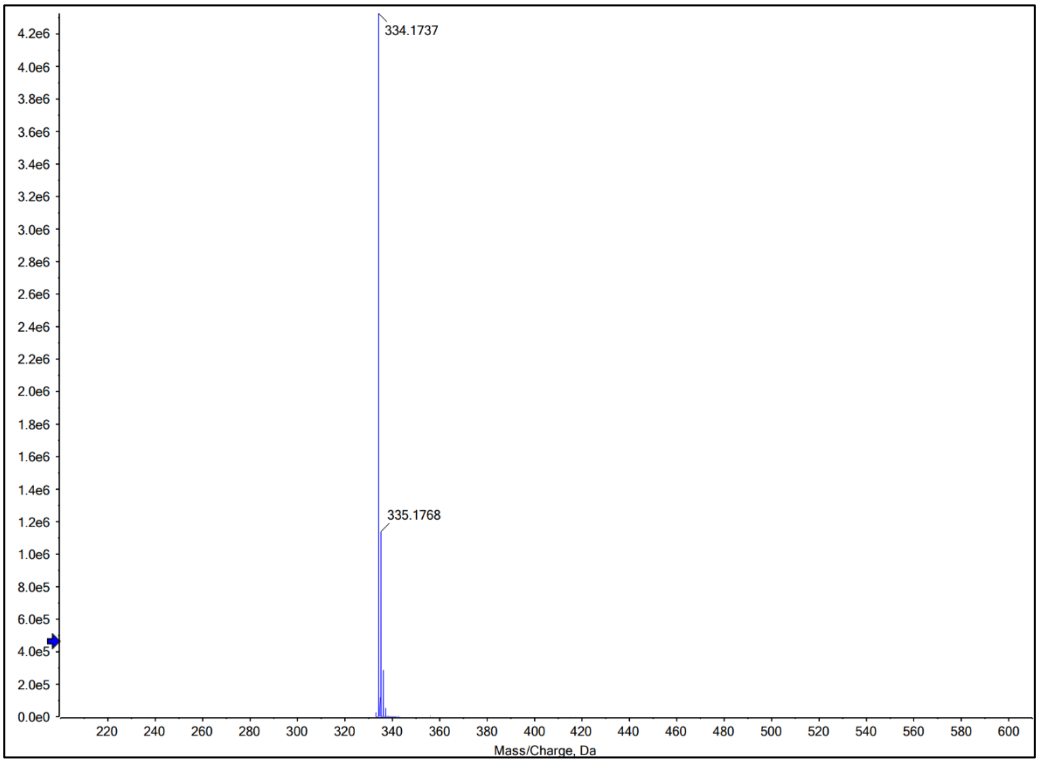

Figure S108. HRMS spectrum of compound 4b.

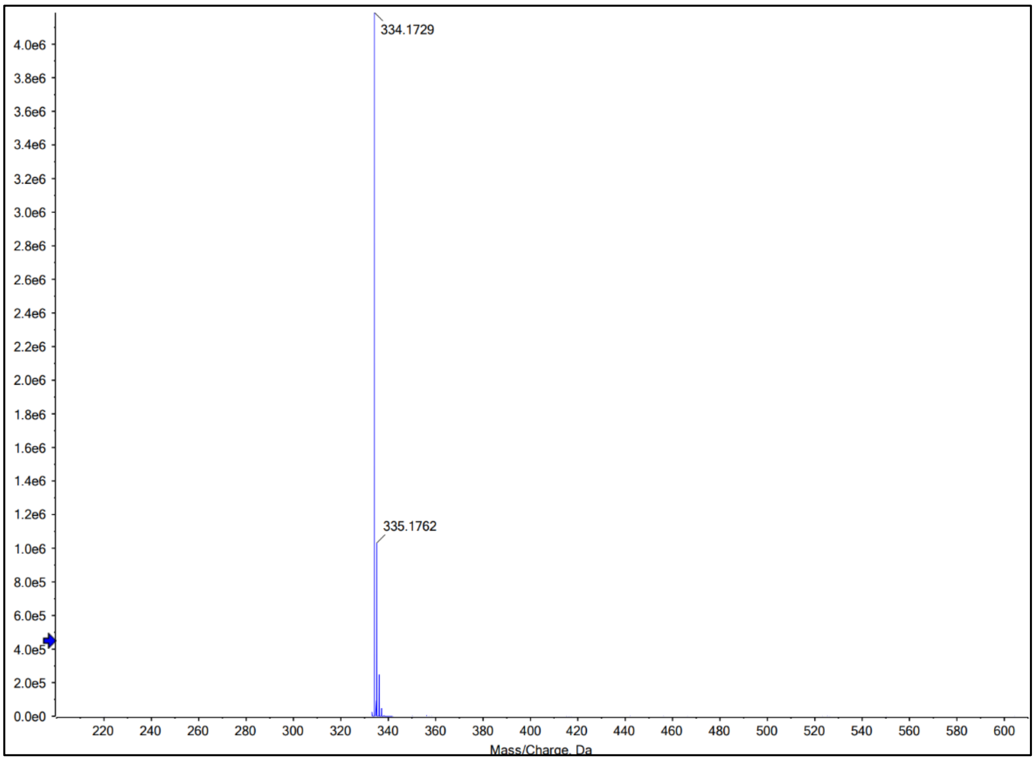

Figure S109. HRMS spectrum of compound 4c.

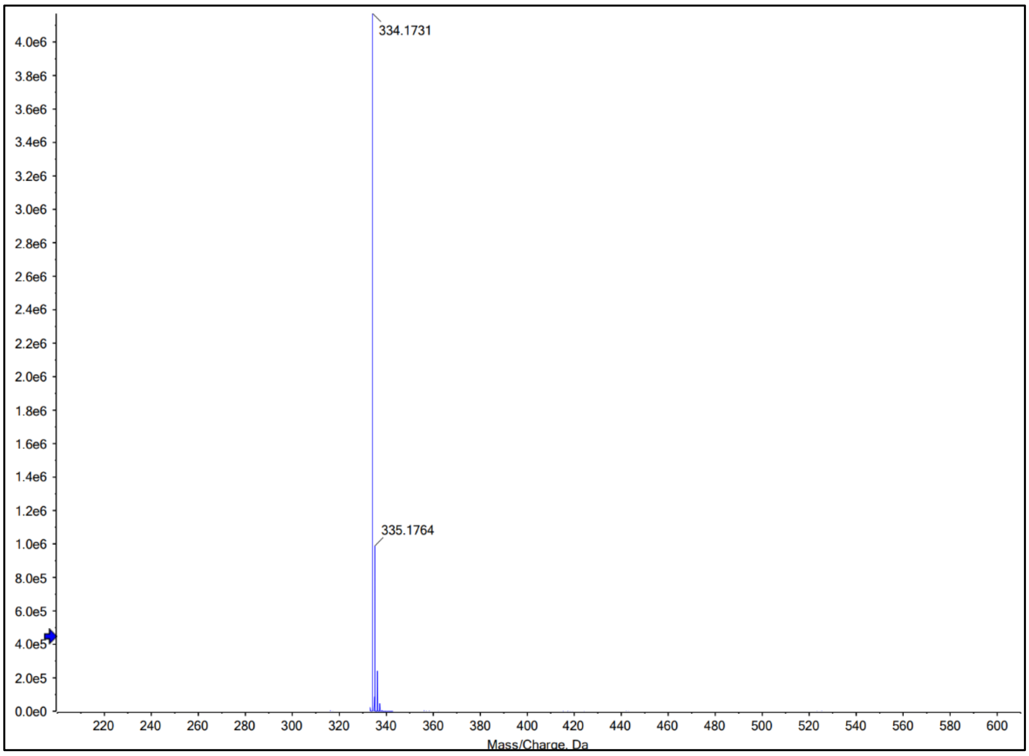

Figure S110. HRMS spectrum of compound 4d.

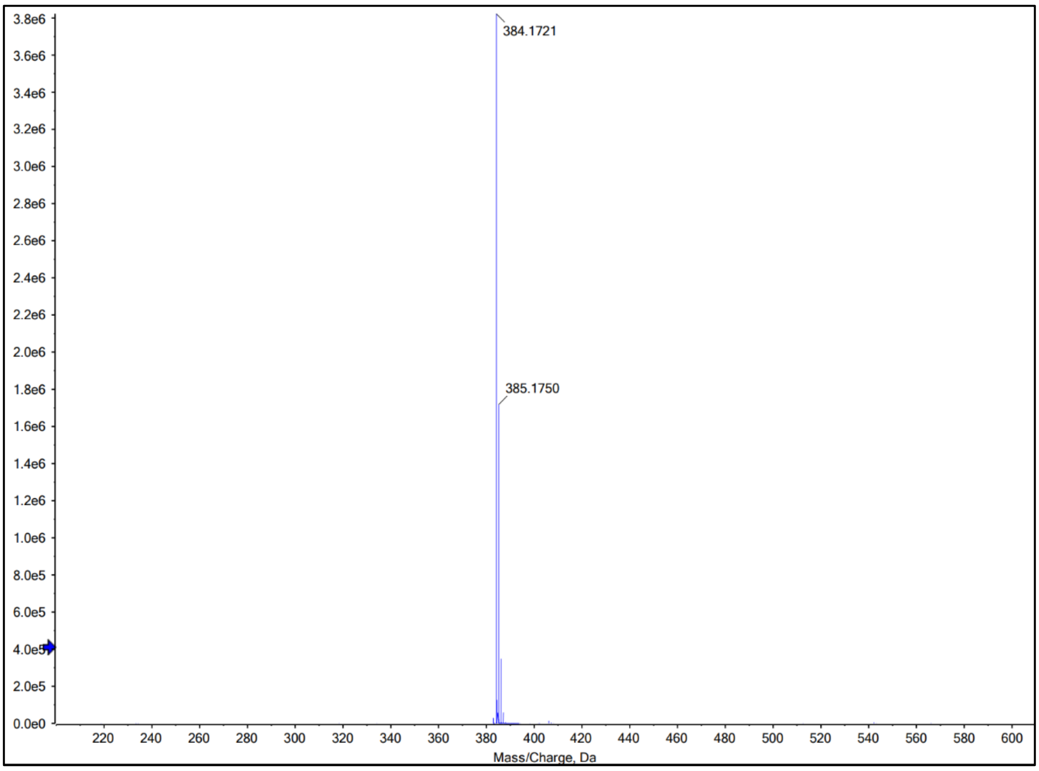

Figure S111. HRMS spectrum of compound 4e.

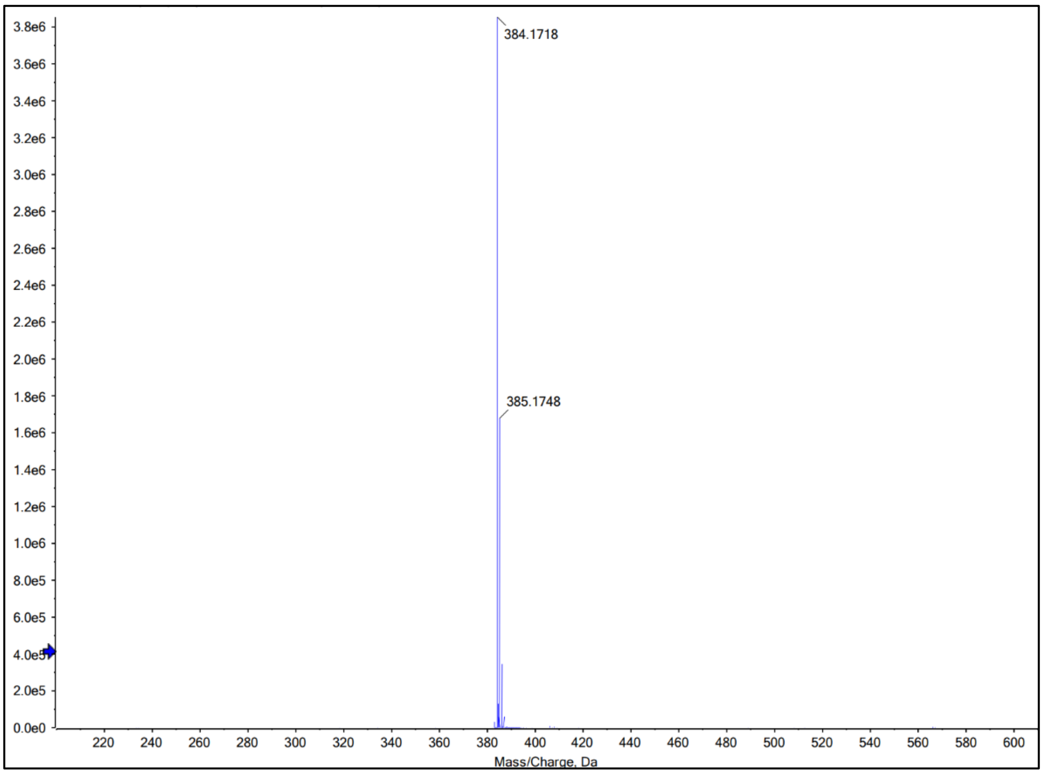

Figure S112. HRMS spectrum of compound 4f.

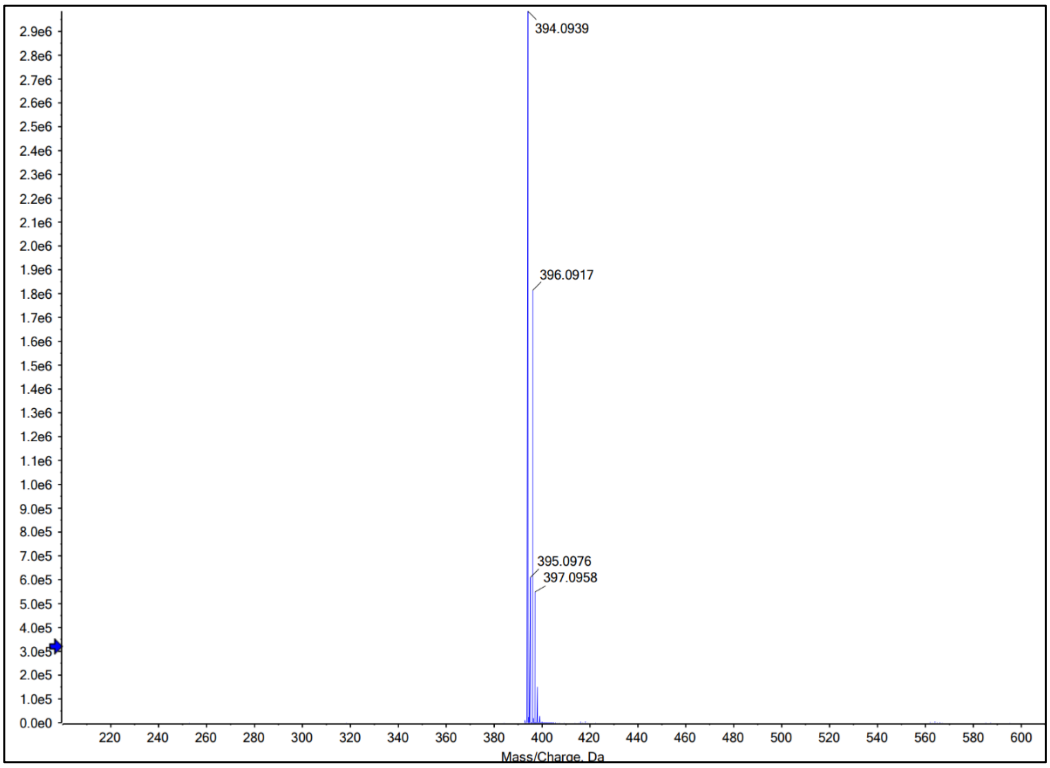

Figure S113. HRMS spectrum of compound 4g.

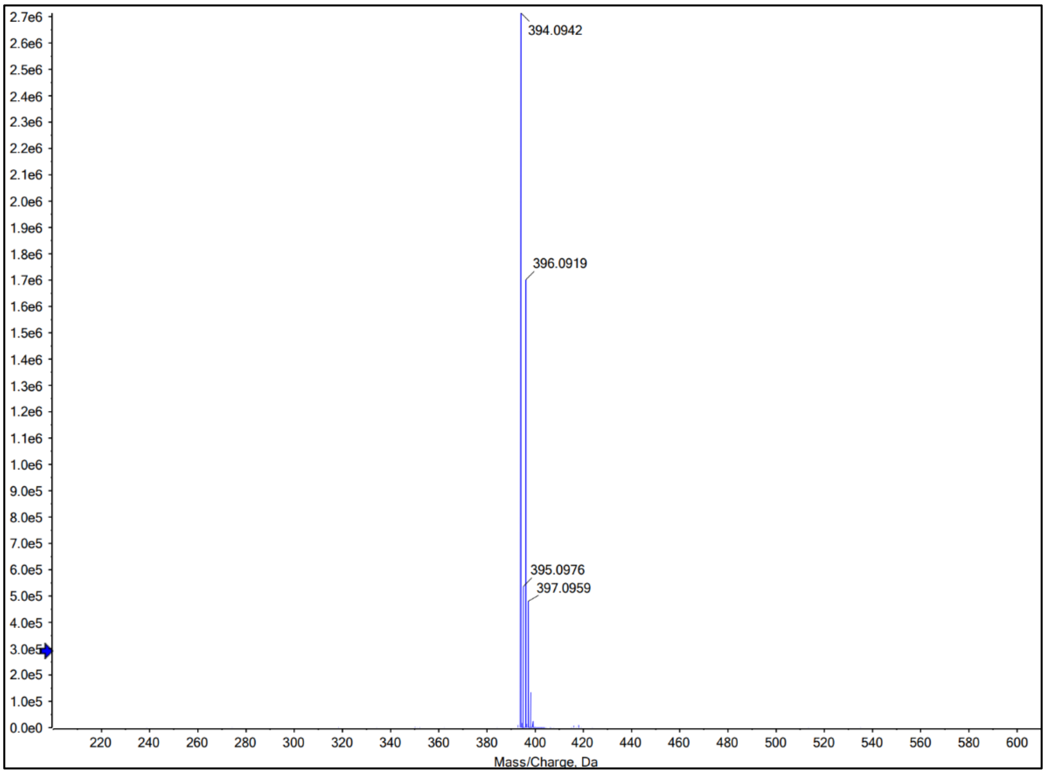

Figure S114. HRMS spectrum of compound 4h.

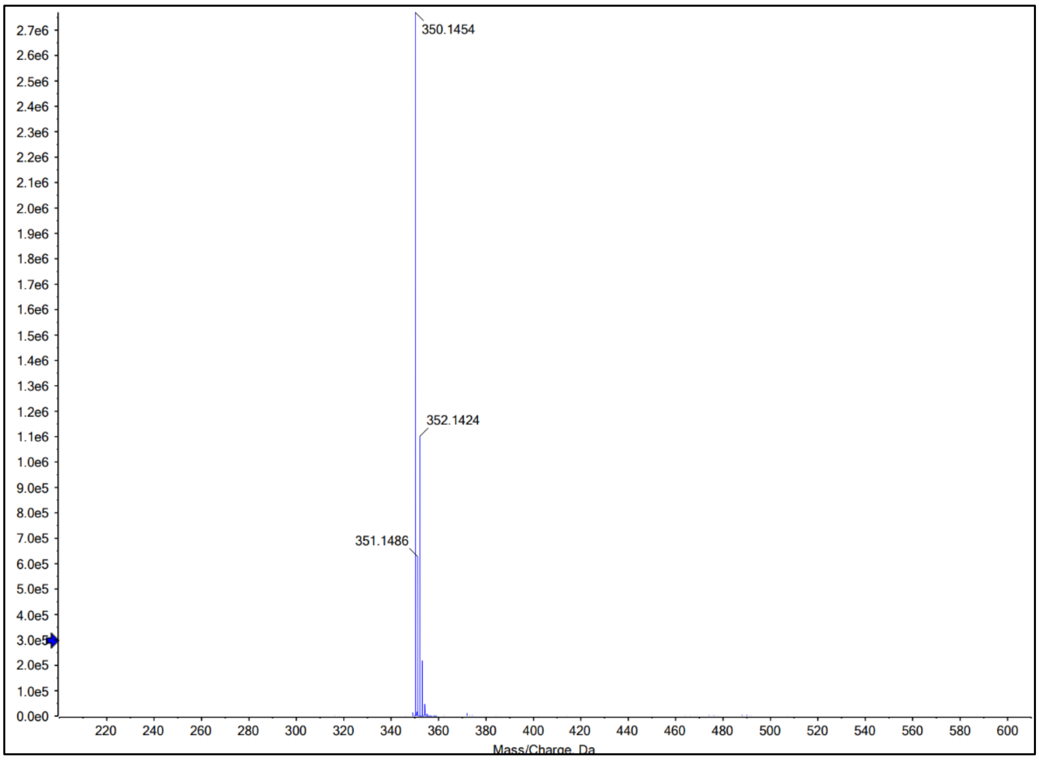

Figure S115. HRMS spectrum of compound 4i.

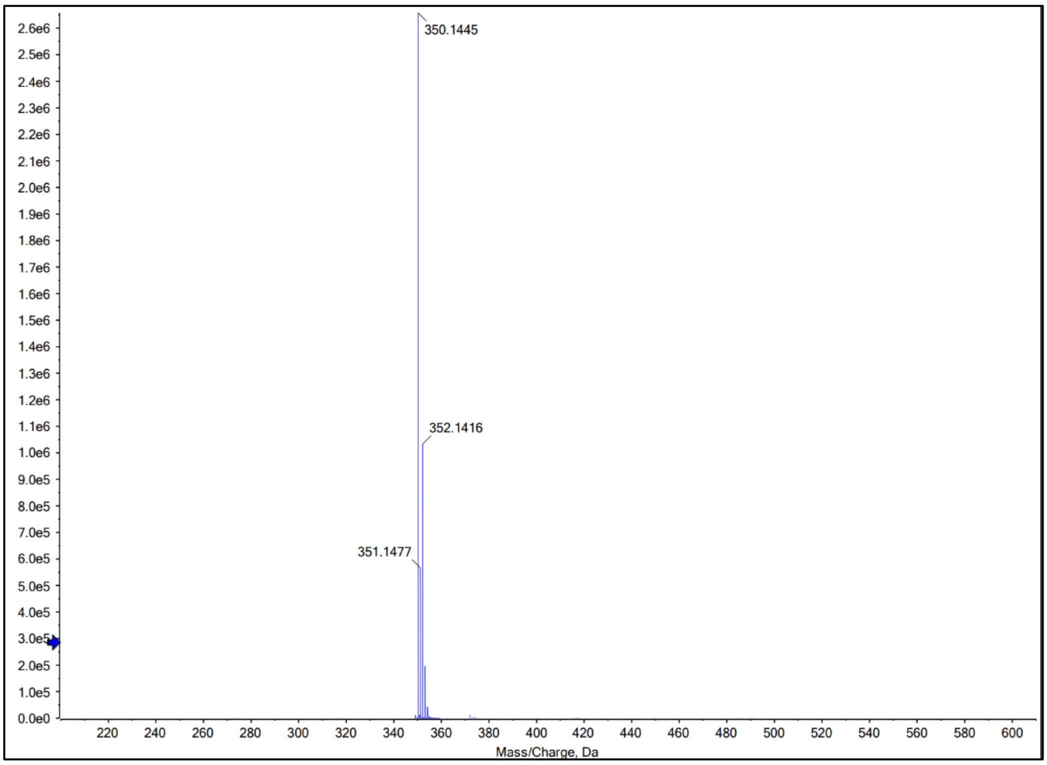

Figure S116. HRMS spectrum of compound 4j.

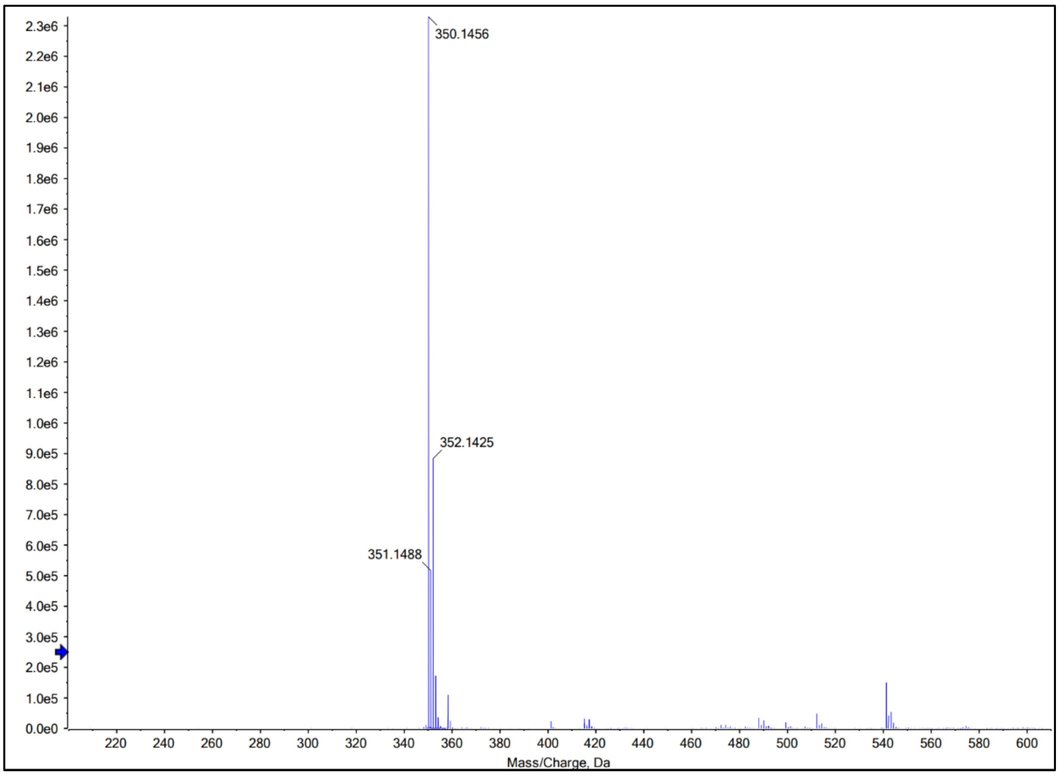

Figure S117. HRMS spectrum of compound 4k.

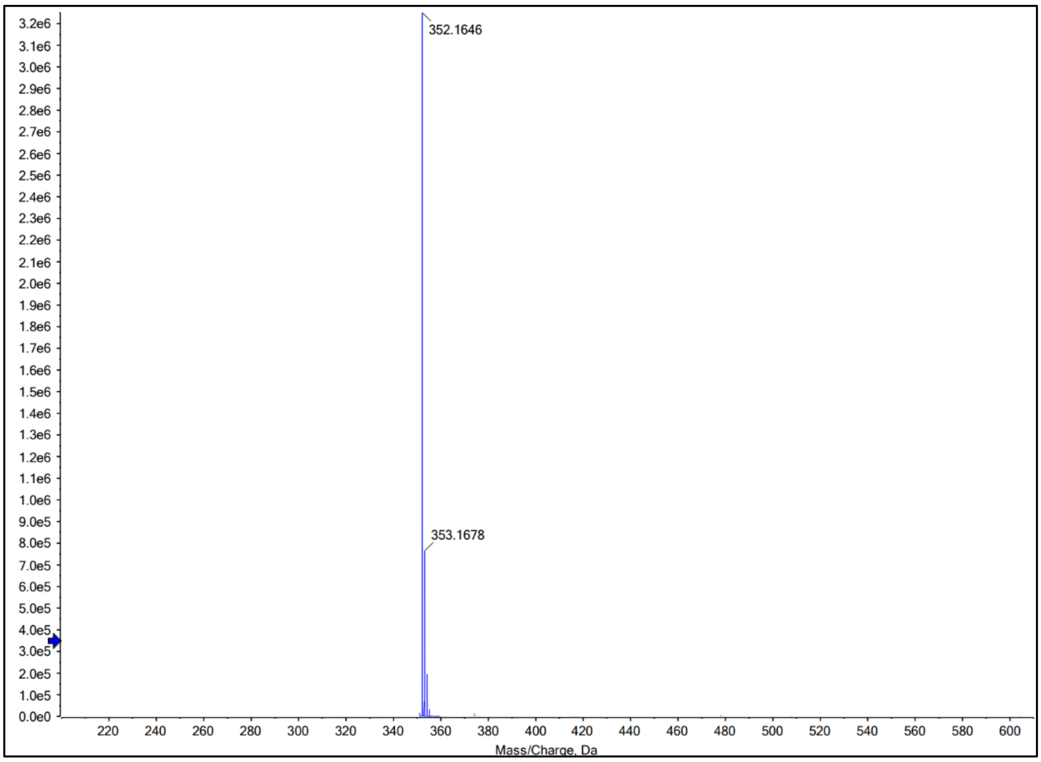

Figure S118. HRMS spectrum of compound 4l.

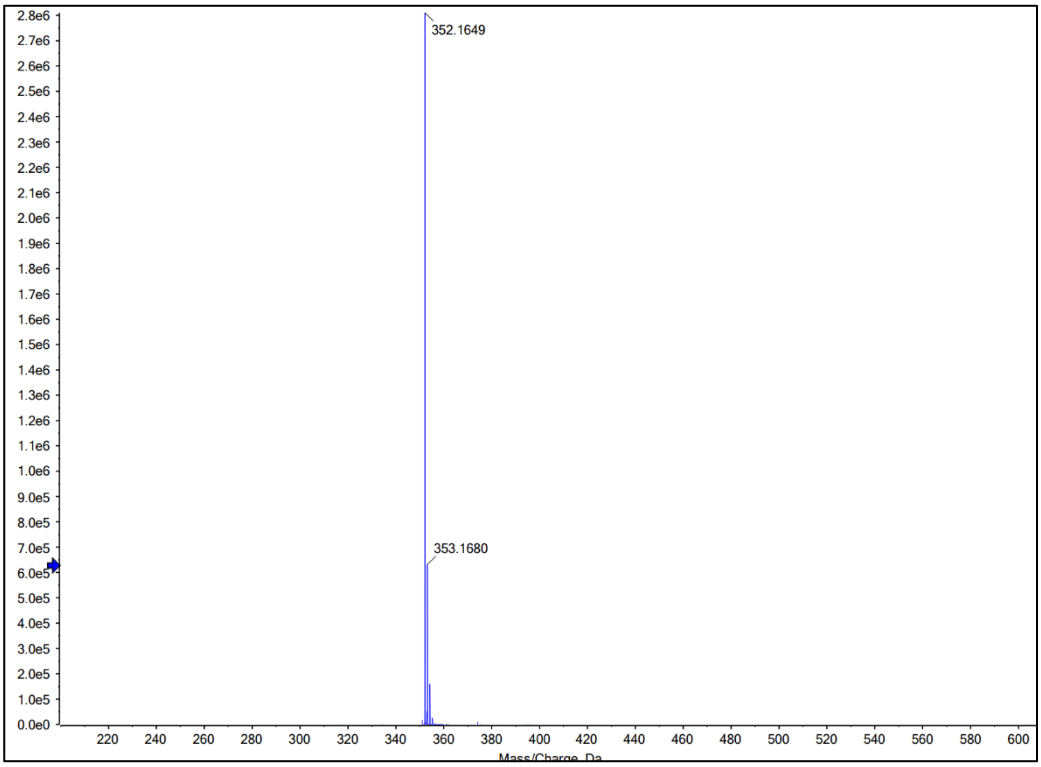

Figure S119. HRMS spectrum of compound 4m.

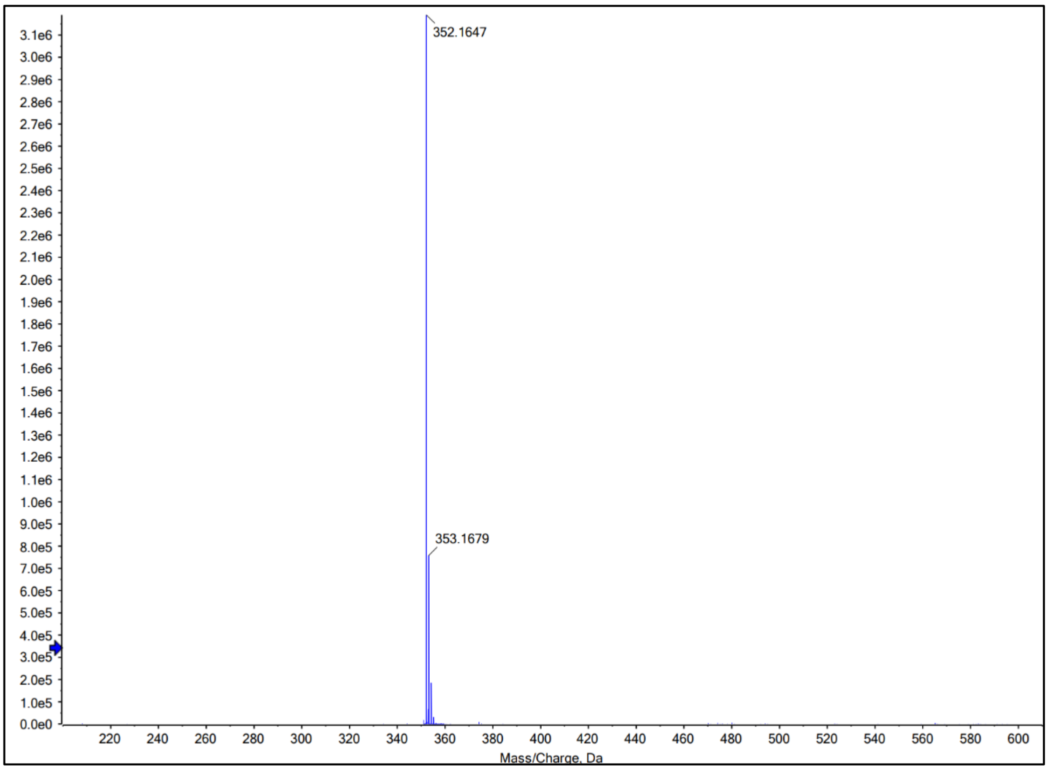

Figure S120. HRMS spectrum of compound 4n.

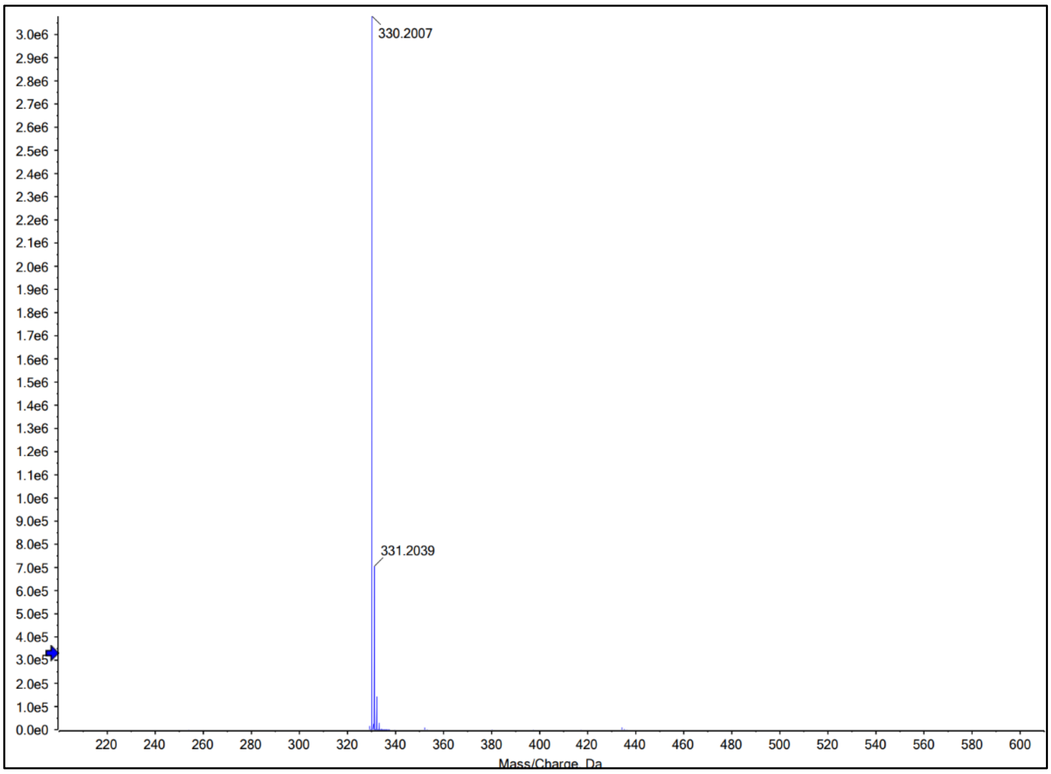

Figure S121. HRMS spectrum of compound 4o.

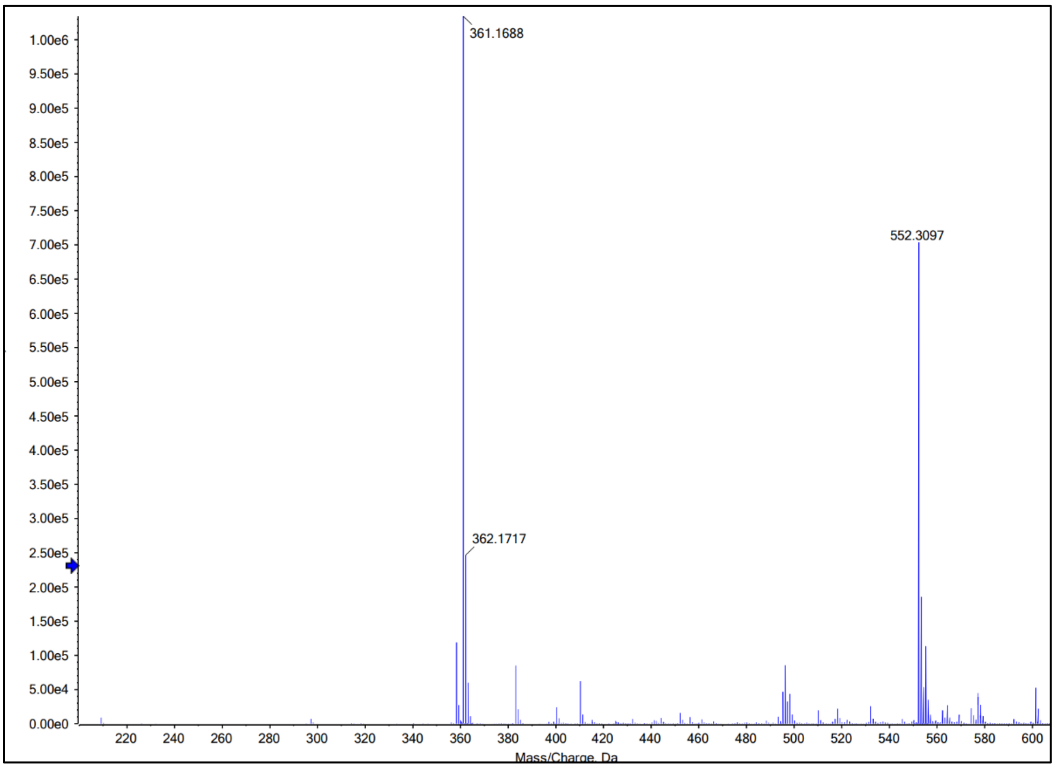

Figure S122. HRMS spectrum of compound 4p.

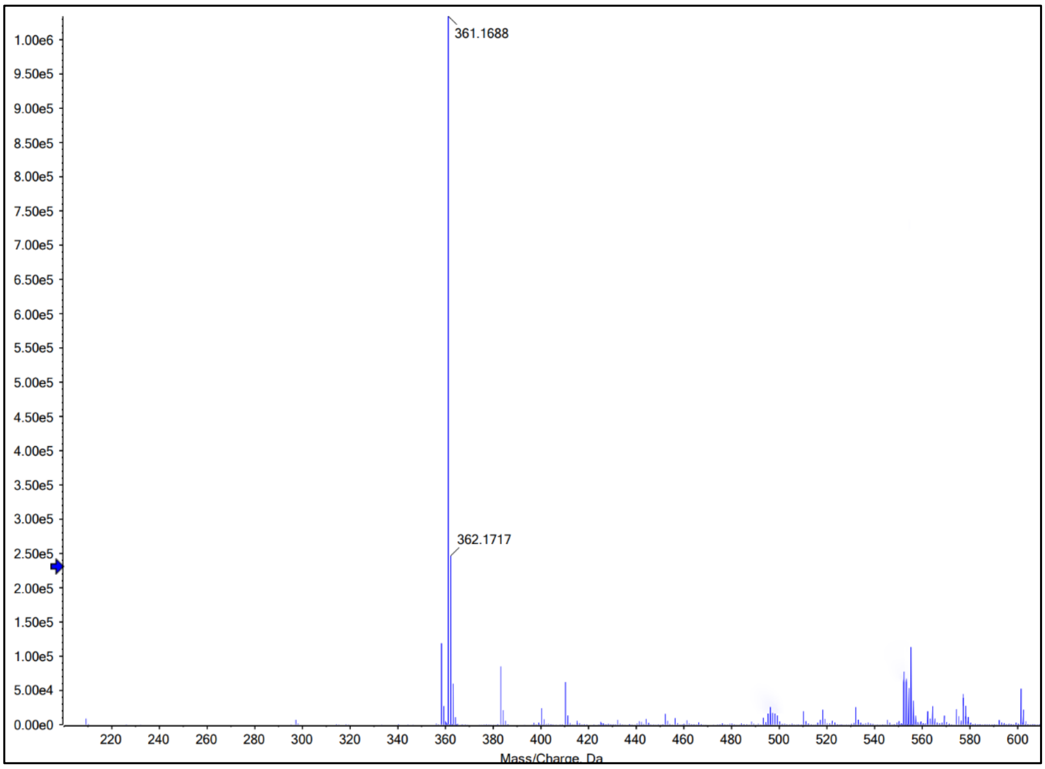

Figure S123. HRMS spectrum of compound 4q.

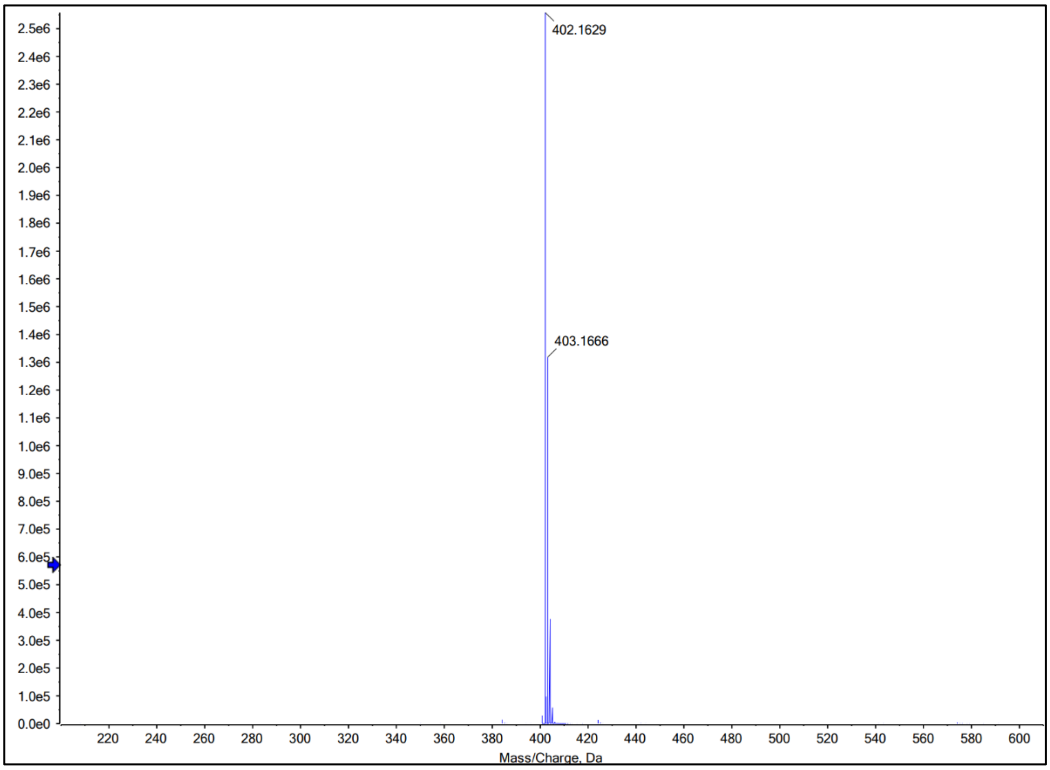

Figure S124. HRMS spectrum of compound 4r.

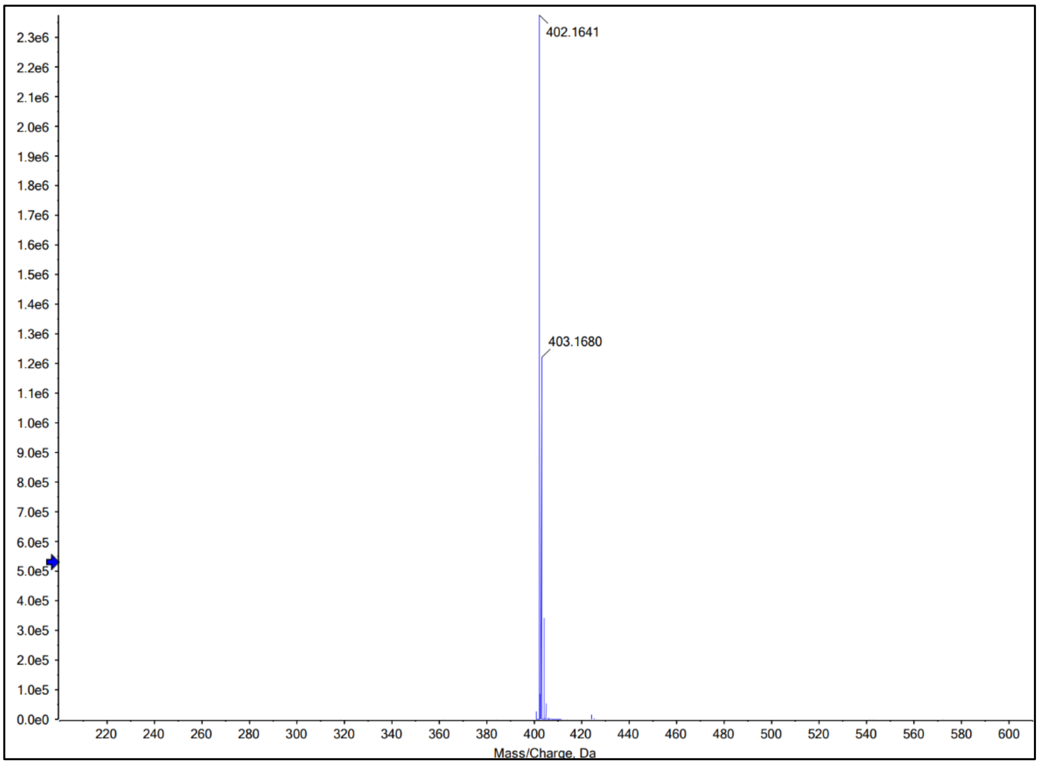

Figure S125. HRMS spectrum of compound 4s.

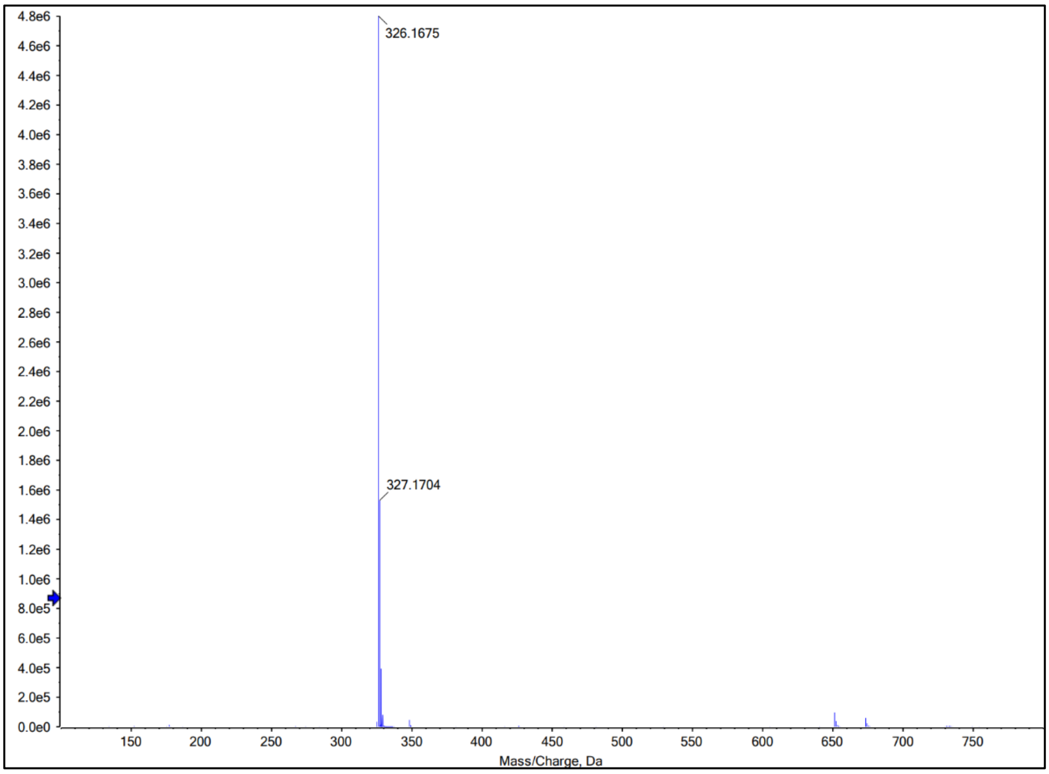

Figure S126. HRMS spectrum of compound 5a.

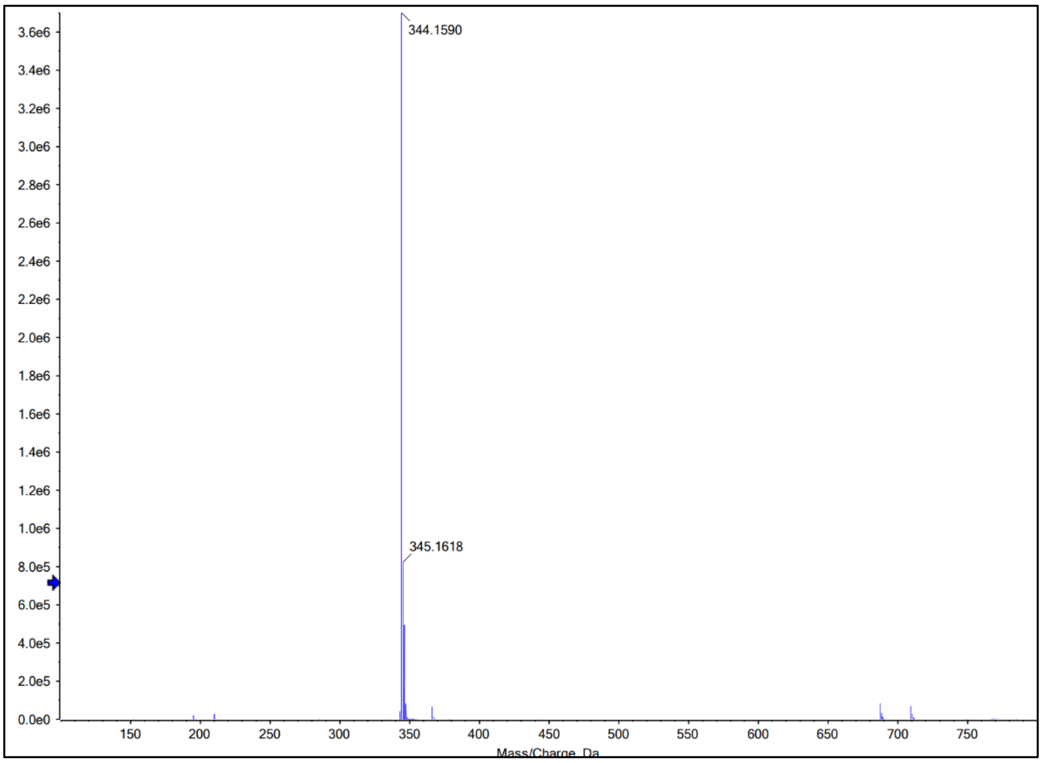

Figure S127. HRMS spectrum of compound 5b.

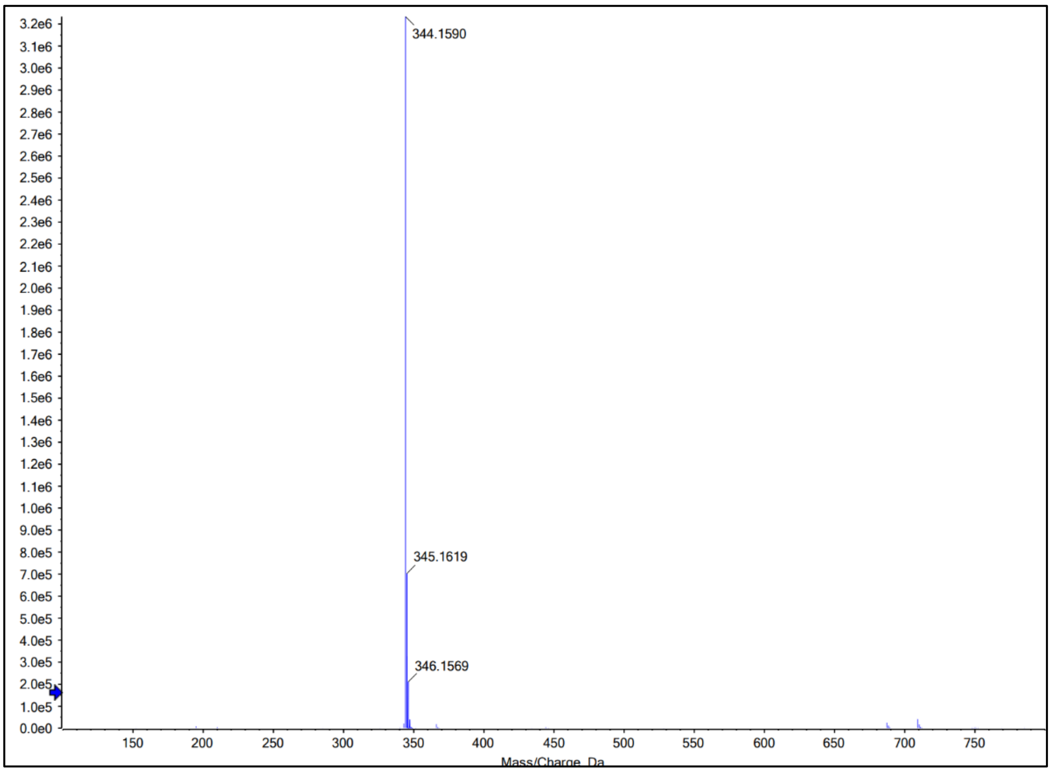

Figure S128. HRMS spectrum of compound 5c.

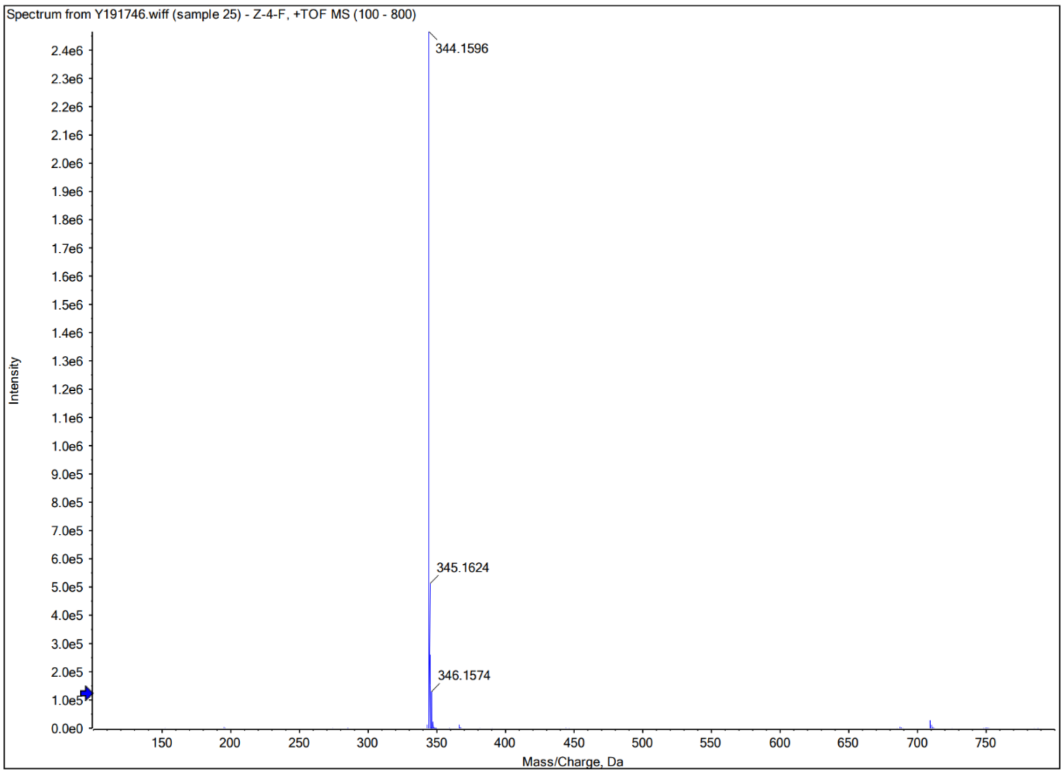

Figure S129. HRMS spectrum of compound 5d.

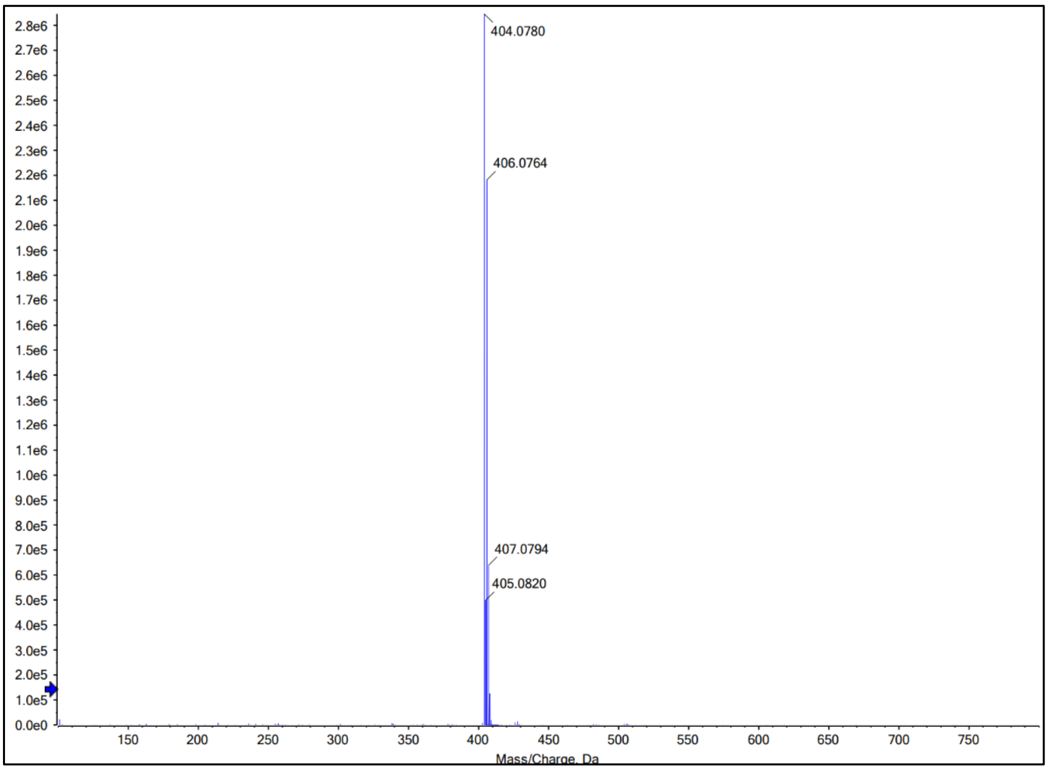

Figure S130. HRMS spectrum of compound 5e.

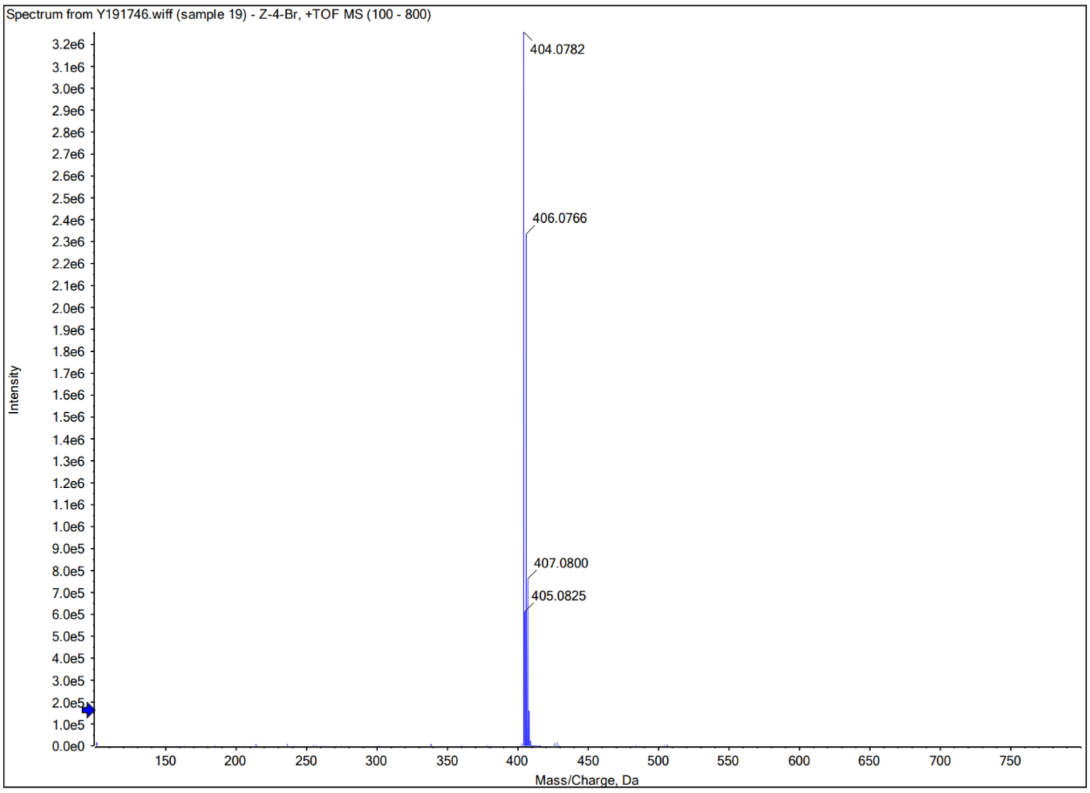

Figure S131. HRMS spectrum of compound 5f.

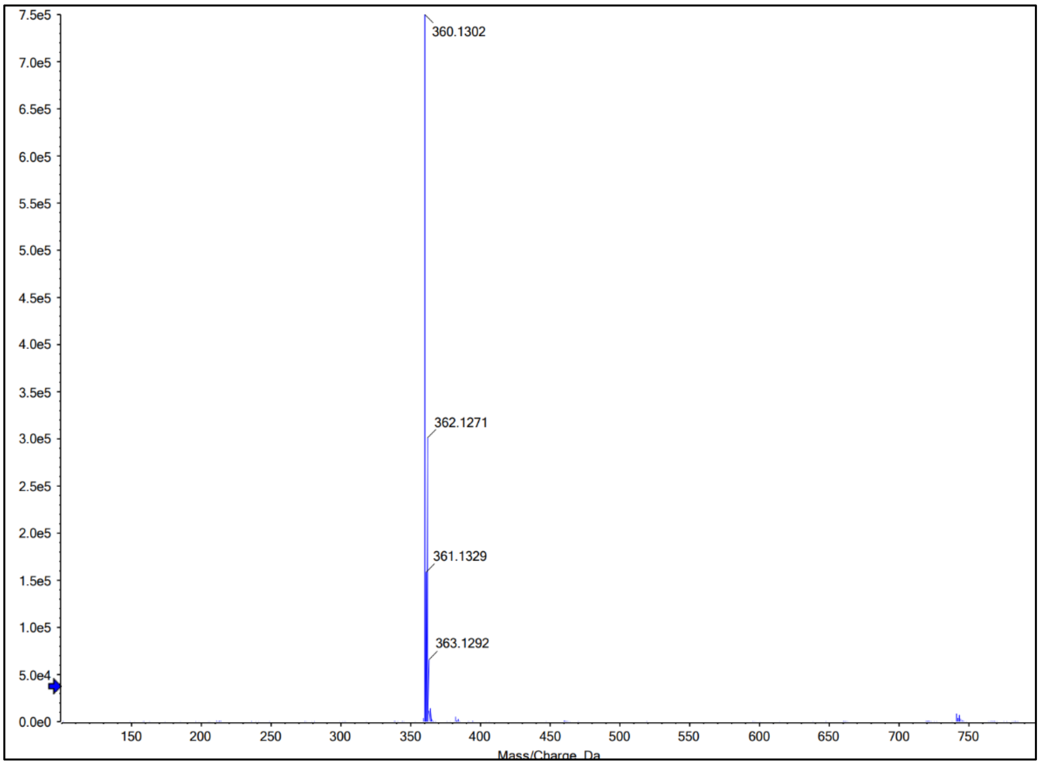

Figure S132. HRMS spectrum of compound 5g.

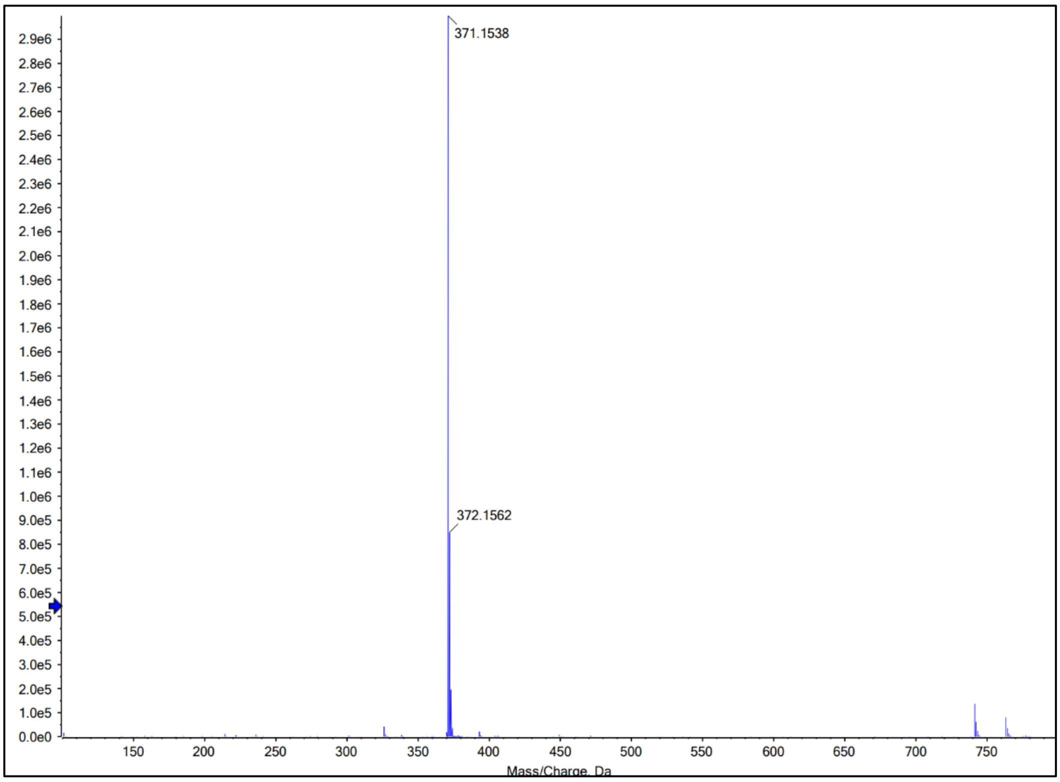

Figure S133. HRMS spectrum of compound 5h.

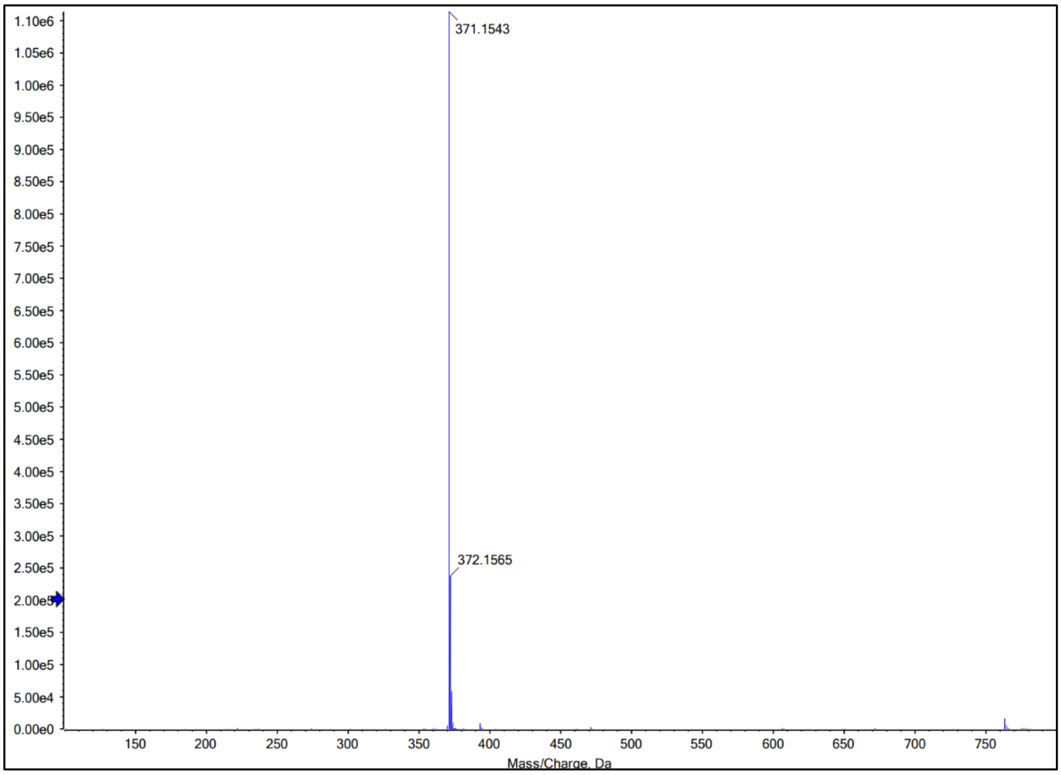

Figure S134. HRMS spectrum of compound 5i.

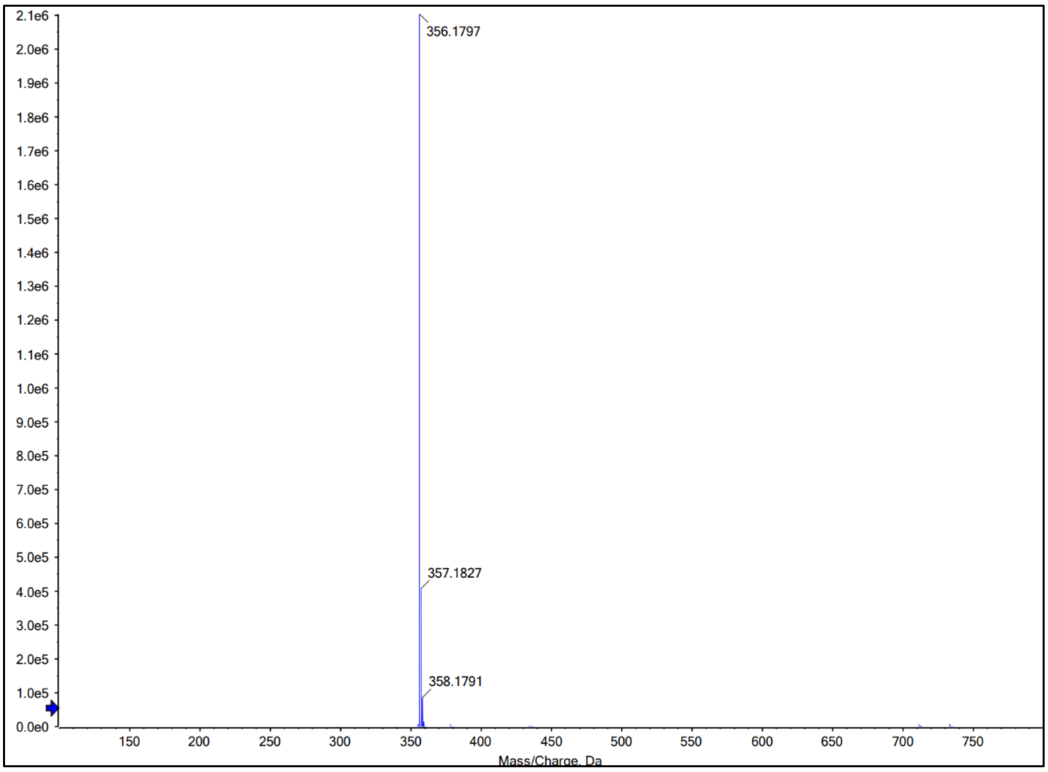

Figure S135. HRMS spectrum of compound 5j.

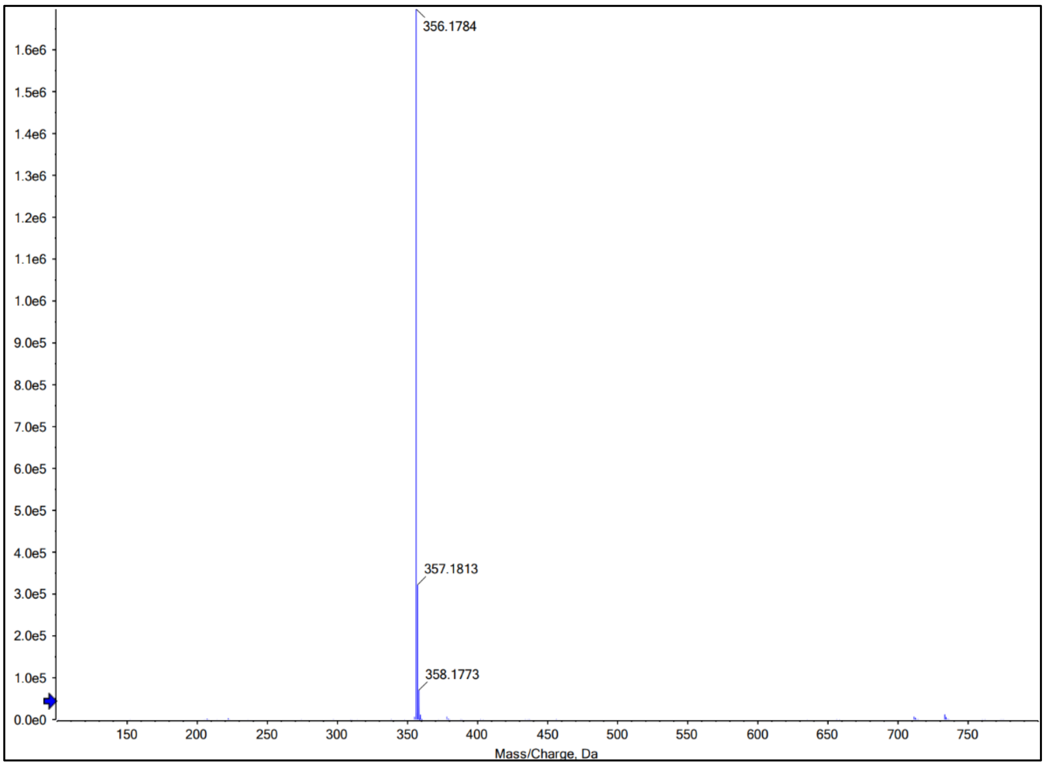

Figure S136. HRMS spectrum of compound 5k.

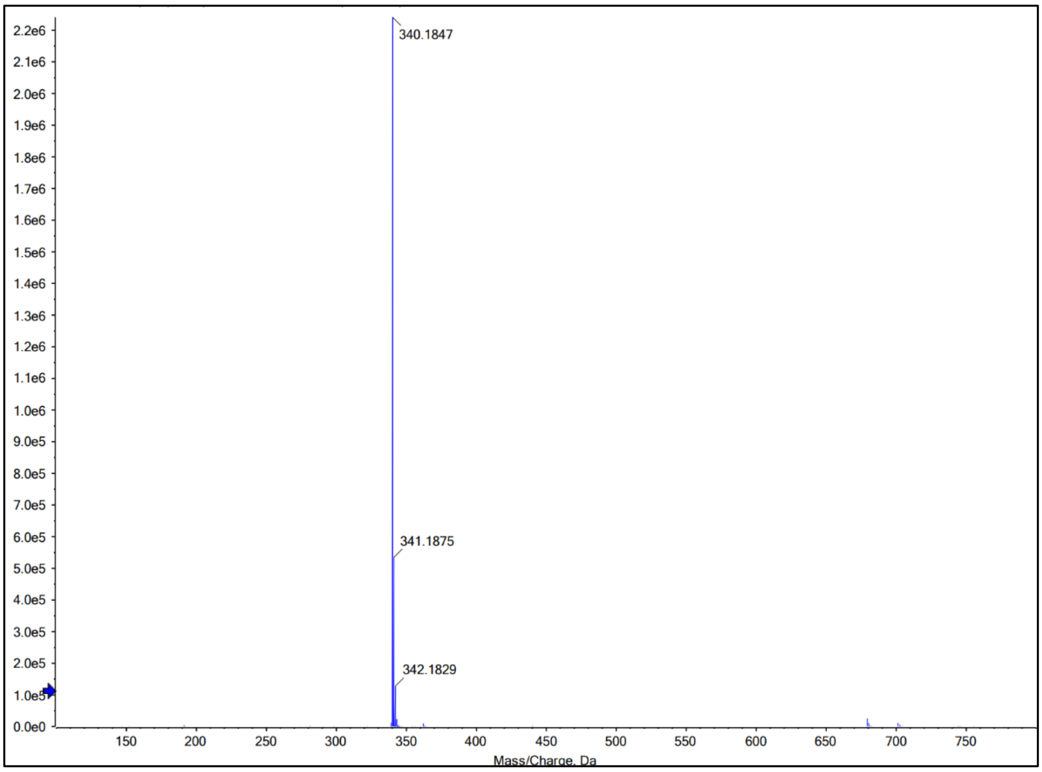

Figure S137. HRMS spectrum of compound 5l.

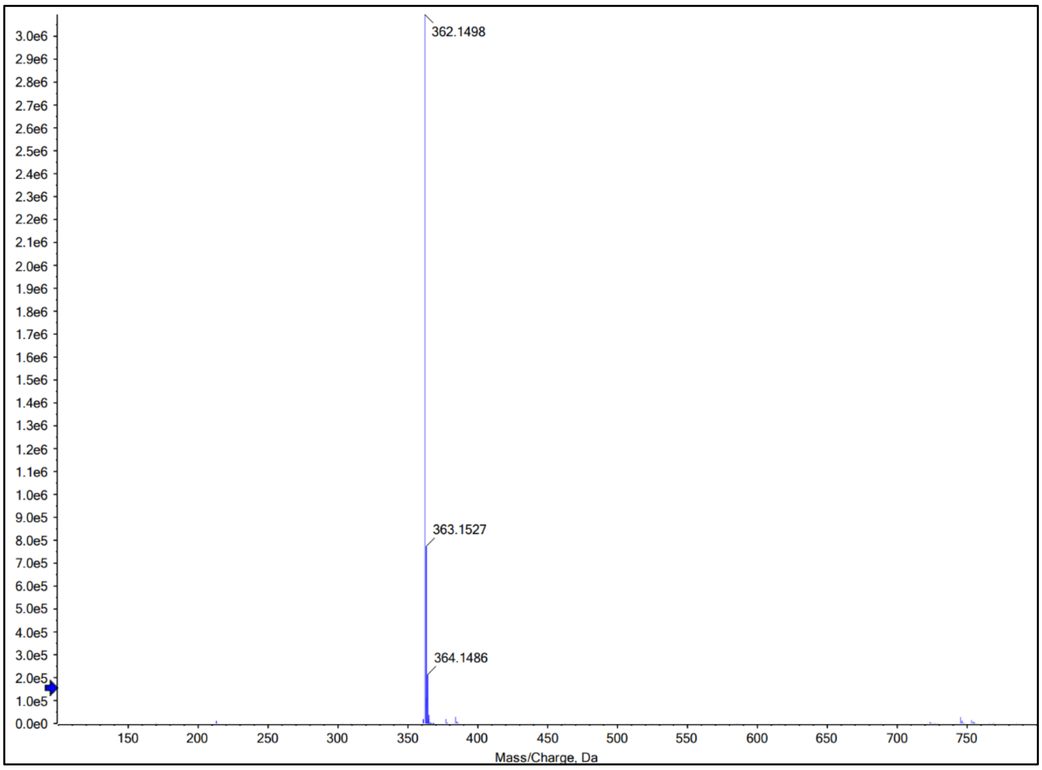

Figure S138. HRMS spectrum of compound 5m.

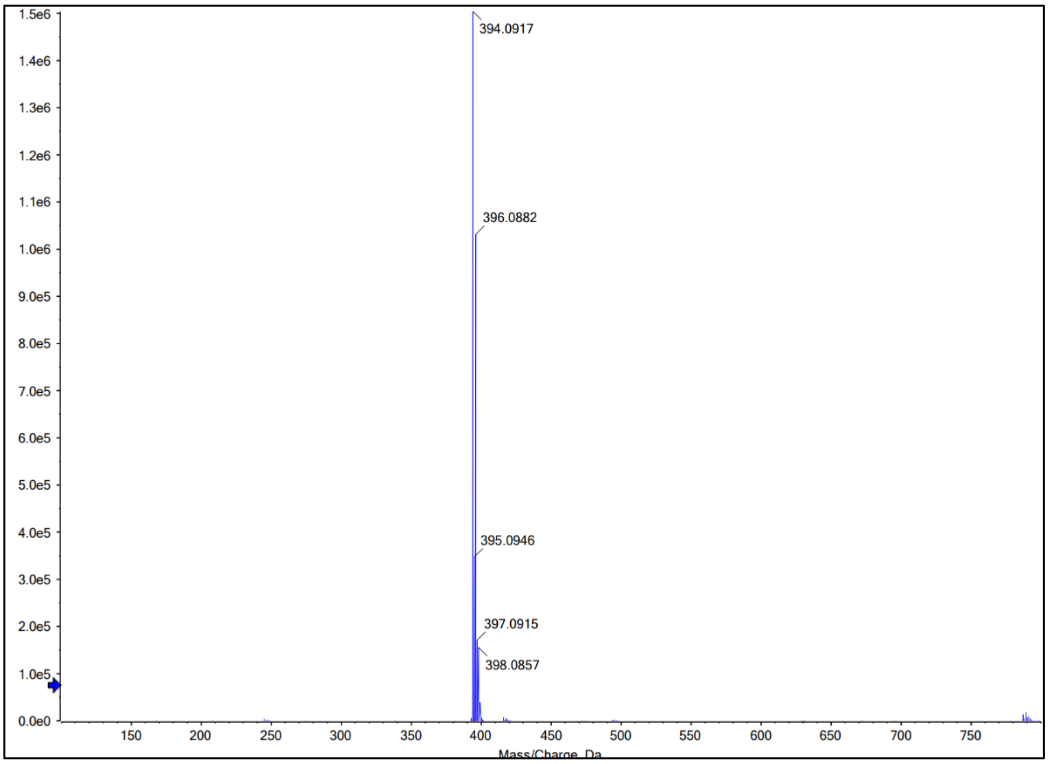

Figure S139. HRMS spectrum of compound 5n.

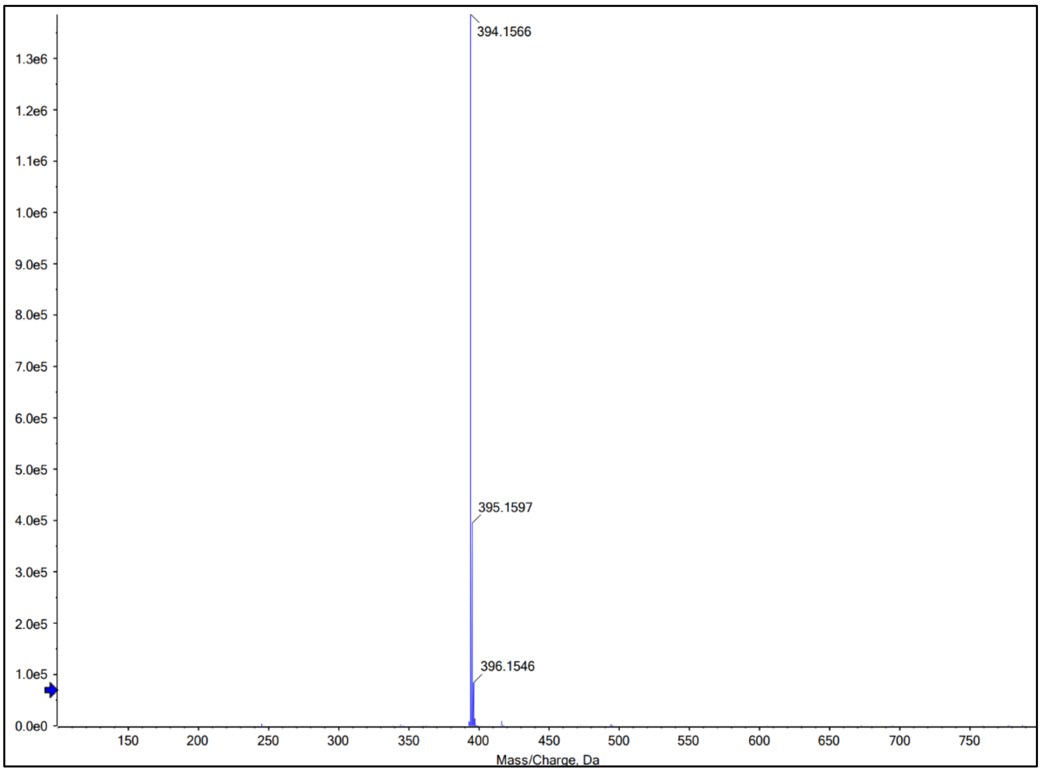

Figure S140. HRMS spectrum of compound 5o.

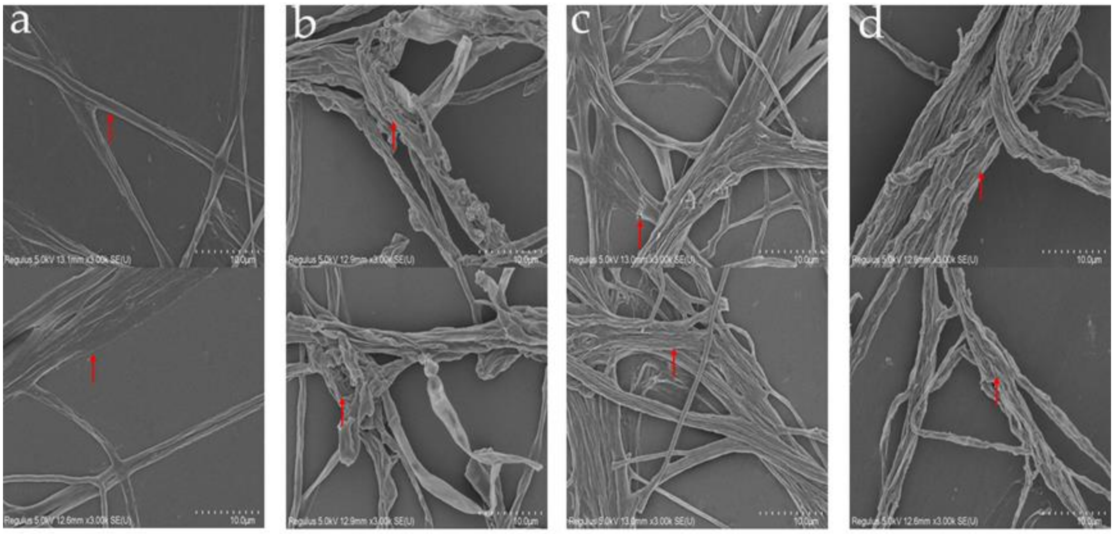

Figure S141. Scanning electron micrographs of mycelial morphology of *T. versicolor*: [a]blank control, ×3000; [b]treated with compound **3a** at 0.43 mg/L (EC_{50}), ×3000; [c]treated with compound **4a** at 6.80 mg/L (EC_{50}), ×3000; [d]treated with compound 5k at 4.86 mg/L (EC_{50}), ×3000

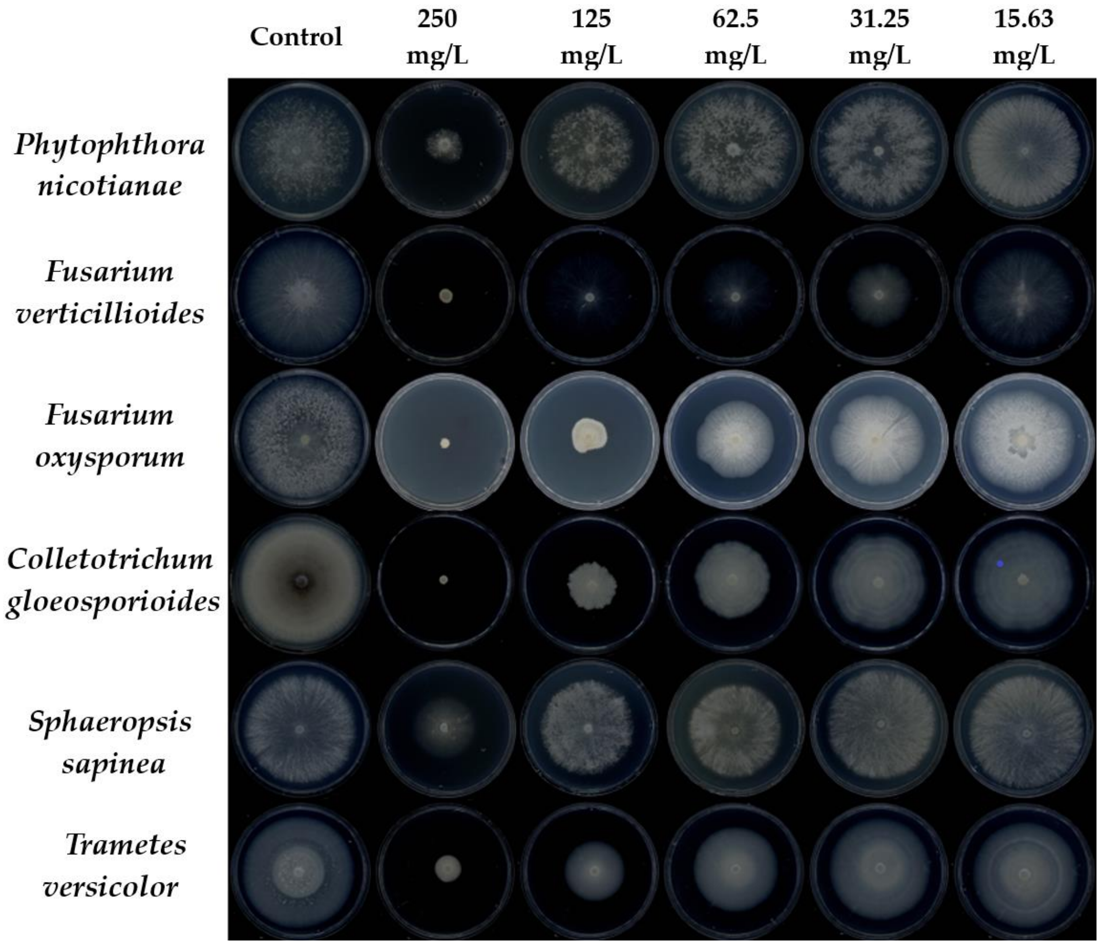

Figure S142. Photograph of antifungal experiments of tricyclazole against six fungi

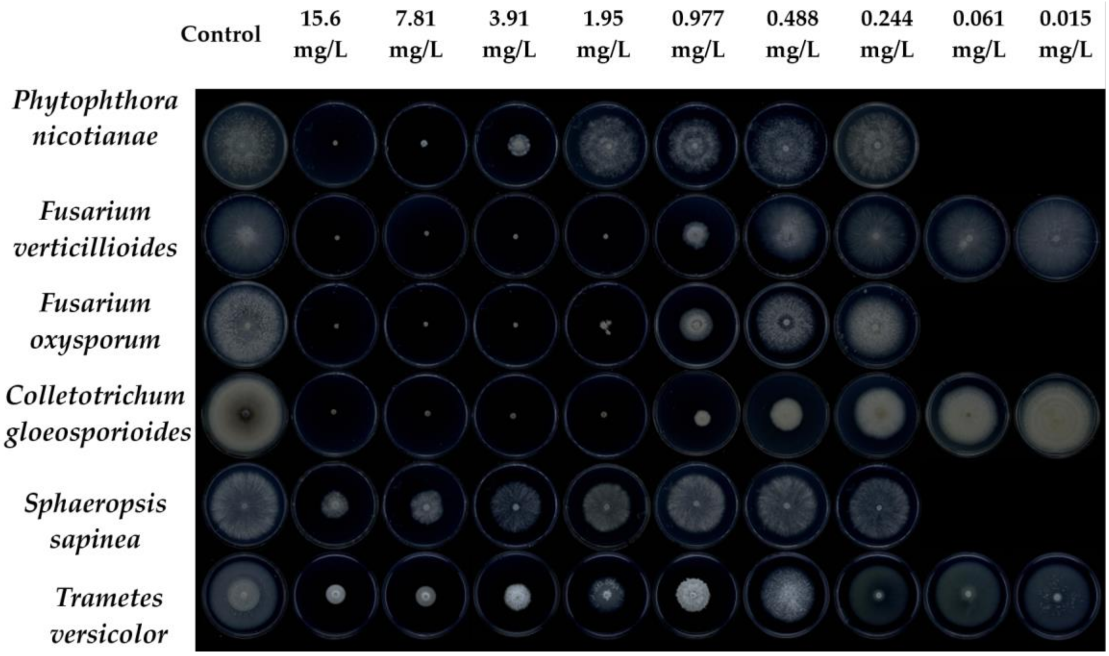

Figure S143. Photograph of antifungal experiments of carbendazim against six fungi

Table S1. EC_{50} values and toxicity regression equation of camphor derivatives against *Phytophthora nicotianae* (mg/L)

Compd.	Testing the concentration(mg/L)					EC_{50}(mg/L)	toxicity regression equation
	250	125	62.5	31.3	15.6		
1	24.82±2.13	18.80±1.49	18.07±1.80	15.66±1.80	10.84±1.70	>1000	y=-2.90+0.740x r=0.929
2	66.28±1.73	20.55±3.77	10.16±0.86	9.24±0.57	7.16±0.00	215.66	y=-5.95+2.46x r=0.789
3a	100.00±0.00	95.66±0.00	87.95±0.68	59.28±1.23	28.43±1.56	25.08	y=-6.33+4.54x r=0.995
4a	54.46±1.02	40.24±1.80	31.08±0.68	20.48±1.18	16.63±1.70	207.80	y=-3.52+1.51x r=0.987
4b	50.60±1.23	40.24±1.80	27.71±0.00	20.48±1.18	17.35±2.66	253.14	y=-3.31+1.37x r=0.978
4c	58.07± 0.59	42.89±2.7	28.19±0.68	17.83±2.07	13.01±0.90	176.72	y=-4.25+1.89 r=0.992
4d	60.84±0.68	44.82±0.34	29.40±0.90	18.31±1.77	13.98±1.56	158.81	y=-4.25+1.93x r=0.987
4e	66.02±1.02	61.45±1.23	46.99±1.80	28.92±2.07	11.81±0.59	89.92	y=-4.39+2.23x r=0.939
4f	32.05±1.56	20.24±1.90	17.83±0.90	14.70±1.18	13.25±1.18	>1000	y=-3.03+0.880x r=0.896
4g	9.40±0.68	8.43±0.68	7.71±1.49	6.75±1.02	6.27±1.23	>1000	y=-3.16+0.370x r=0.994

Compd.	Testing the concentration(mg/L)					EC_{50}(mg/L)	toxicity regression equation
	250	125	62.5	31.3	15.6		
4h	68.68±0.68	63.37±0.68	49.64±0.90	26.99±1.56	18.80±1.49	79.78	y=-3.83+2.01x r=0.963
4i	10.12±0.34	8.43±0.34	7.95±0.68	6.99±0.68	6.75±0.59	>1000	y=-3.10+0.360x r=0.946
4j	66.75±0.00	59.04±0.34	40.72±1.18	21.93±1.18	13.74±0.68	101.81	y=-4.48+2.23x r=0.982
4k	67.71±1.23	57.11±0.68	44.82±0.34	27.71±1.18	19.04±1.18	91.87	y=-3.67+1.87x r=0.992
4l	55.20±2.36	46.42±0.65	34.87±0.00	27.25±0.57	24.94±1.18	178.16	y=-2.59+1.15x r=0.969
4m	61.43±1.42	47.58±1.31	35.57±1.70	28.18±0.86	25.40±1.99	137.26	y=-2.79+1.30x r=0.955
4n	56.81±0.33	45.27±0.98	34.18±0.57	26.10±0.65	24.48±0.57	174.28	y=-2.73+1.21x r=0.957
4o	24.71±1.31	24.25±0.65	22.17±1.63	21.02±1.96	18.01±1.18	>1000	y=-1.86+0.330x r=0.931
4p	45.04±0.65	44.34±0.86	40.65±1.18	30.72±0.00	27.48±1.73	341.97	y=-1.79+0.710x r=0.911
4q	36.72±1.31	35.34±1.82	30.72±2.26	30.25±0.65	20.55±1.99	>1000	y=-1.93+0.610x r=0.841
4r	64.20±1.82	59.58±2.29	47.58±2.14	36.72±0.65	31.87±0.86	73.59	y=-2.25+1.20x r=0.980

Compd.	Testing the concentration(mg/L)					EC_{50}(mg/L)	toxicity regression equation
	250	125	62.5	31.3	15.6		
4s	58.43±1.13	51.96±0.65	44.80±1.82	33.72±0.86	27.71±0.33	112.39	y=-2.29+1.11x r=0.990
tricyclazole	91.91±1.35	60.48±2.43	35.71±2.33	21.43±3.50	8.10±3.09	80.58	y=-7.12+3.80x r=0.963

Table S2. EC_{50} values and toxicity regression equation of camphor derivatives against *Fusarium verticillioides* (mg/L)

Compd.	Testing the concentration(mg/L)					EC_{50}(mg/L)	toxicity regression equation
	250	125	62.5	31.3	15.6		
1	25.55±4.04	13.66±2.75	11.01±2.02	9.69±2.02	7.05±1.53	>1000	y=-3.47+0.780x r=0.974
2	62.78±5.54	42.51±5.16	24.23±4.25	13.66±2.02	7.93±0.76	172.24	y=-5.34+2.38x r=0.995
3a	77.97±1.53	69.16±2.02	59.91±5.00	44.93±1.53	32.60±1.32	40.18	y=-2.78+1.73x r=0.995
4a	63.88±0.76	58.81±0.38	43.39±1.32	31.72±2.02	16.30±2.02	82.34	y=-4.12+2.15x r=0.987
4b	67.84±2.75	55.29±2.98	44.05±2.02	35.24±0.00	24.67±3.50	88.85	y=-2.82+1.45x r=0.997
4c	68.94±1.98	60.13±0.38	50.44±3.43	38.77±4.25	28.19±4.25	63.91	y=-2.71+1.5x r=0.998
4d	68.72±0.38	61.24±0.38	47.14±1.75	36.56±3.43	24.45±1.66	69.10	y=-3.17+1.72x r=0.997
4e	72.03±1.38	64.32±3.50	56.17±1.66	31.72±2.02	16.30±2.02	62.16	y=-4.59+2.55x r=0.966
4f	40.09±2.02	18.07±0.00	16.08±0.66	13.66±2.02	12.34±2.02	>1000	y=-2.58+0.510x r=0.992
4g	16.74±0.00	11.01±0.76	9.26±1.53	8.37±0.76	7.27±2.12	>1000	y=-3.14+0.49x r=0.989

Compd.	Testing the concentration(mg/L)					EC_{50}(mg/L)	toxicity regression equation
	250	125	62.5	31.3	15.6		
4h	74.89±0.00	72.03±1.01	59.91±0.76	48.68±1.01	36.12±2.02	34.39	y=-2.55+1.66x r=0.999
4i	67.62±0.66	59.25±1.01	52.20±1.31	45.15±0.63	42.07±1.31	45.97	y=-1.31+0.790x r=0.973
4j	73.11±2.62	68.49±1.26	54.20±1.46	47.48±1.93	36.13±1.93	39.68	y=-2.29+1.43x r=0.980
4k	78.57±1.26	68.49±2.52	57.14±1.26	44.96±1.93	34.87±0.73	40.54	y=-2.51+1.56x r=0.999
4l	68.91±2.62	62.40±1.93	50.42±0.73	39.08±2.62	31.09±0.73	58.83	y=-2.57+1.45x r=0.995
4m	79.20±0.63	68.91±0.36	59.03±0.63	45.80±1.26	43.70±0.73	31.32	y=-1.83+1.22x r=0.938
4n	71.01±1.26	63.03±0.73	48.53±1.31	35.50±1.59	21.43±0.73	66.43	y=-3.65+2.01x r=0.997
4o	55.88±1.26	44.12±3.17	30.25±3.85	21.43±0.73	18.49±0.73	206.14	y=-3.26+1.40x r=0.953
4p	58.40±1.26	49.58±4.55	39.92±1.93	35.29±1.46	25.63±1.26	131.69	y=-2.35+1.11x r=0.980
4q	42.86±3.17	29.83±0.73	23.11±1.26	21.01±0.73	19.75±0.73	>1000	y=-2.16+0.590x r=0.887
4r	54.62±3.34	42.01±3.34	34.87±1.93	32.35±0.73	30.67±1.26	645.97	y=-1.50+0.530x r=0.902

Compd.	Testing the concentration(mg/L)					EC_{50}(mg/L)	toxicity regression equation
	250	125	62.5	31.3	15.6		
4s	44.54±5.04	40.96±3.37	38.24±3.34	34.03±1.46	31.51±3.17	723.36	y=-1.34+0.470x<br>r=0.991
5a	50.84±1.26	47.48±1.93	42.02±1.26	31.93±1.26	19.33±1.26	125.46	y=-3.07+1.47x<br>r=0.950
5b	32.35±1.93	30.67±2.18	29.41±2.18	27.73±1.46	25.63±1.26	>1000	y=-1.38+0.280x<br>r=0.984
5c	44.12±1.46	36.98±1.26	33.19±1.26	30.67±0.00	24.37±2.52	816.15	y=-1.84+0.640x<br>r=0.954
5d	28.76±4.27	26.10±0.77	21.68±2.30	18.14±2.03	13.27±0.77	>1000	y=-2.92+0.910x<br>r=0.986
5e	22.12±2.03	19.91±1.53	18.14±3.98	17.70±2.03	16.36±3.07	>1000	y=-1.93+0.250x<br>r=0.958
5f	20.35±1.33	18.14±2.03	16.37±1.33	14.60±0.77	13.72±0.00	>1000	y=-2.30+0.380x<br>r=0.986
5g	22.12±1.33	21.24±0.77	17.70±2.03	13.72±1.33	11.95±0.77	>1000	y=-2.96+0.780x<br>r=0.987
5h	35.40±2.03	32.74±1.53	32.30±2.30	30.09±2.76	26.11±5.36	>1000	y=-1.42+0.35x<br>r=0.878
5i	19.47±1.53	16.81±0.77	13.72±1.33	12.83±0.77	11.06±0.00	>1000	y=-2.70+0.510x<br>r=0.963
5j	38.94±1.33	35.84±1.53	34.07±1.01	31.42±0.77	31.42±0.77	>1000	y=-1.09+0.240x<br>r=0.900

Compd.	Testing the concentration(mg/L)					EC_{50}(mg/L)	toxicity regression equation
	250	125	62.5	31.3	15.6		
5k	43.80±3.34	39.82±3.07	35.84±0.38	33.63±0.00	32.30±0.00	>1000	y=-1.19+0.360x r=0.950
5l	30.97±2.30	29.20±3.83	21.24±0.77	19.91±0.77	19.03±1.33	>1000	y=-2.23+0.590x r=0.800
5m	22.12±0.77	19.47±1.53	14.16±0.77	13.27±0.77	10.62±0.77	>1000	y=-3.01+0.73x r=0.938
5n	19.47±2.03	17.25±2.03	14.16±2.03	14.16±0.77	11.50±0.77	>1000	y=-2.58+0.470x r=0.900
5o	15.49±2.03	12.39±1.33	11.06±1.33	10.18±0.77	9.74±1.33	>1000	y=-2.61+0.350x r=0.963
tricyclazole	88.05±0.00	46.02±5.03	25.66±2.30	16.37±2.64	15.93±2.03	185.93	y=-3.91+1.69x r=0.871

Table S3. EC_{50} values and toxicity regression equation of camphor derivatives against *Colletotrichum gloeosporioides* (mg/L)

Compd.	Testing the concentration(mg/L)							EC_{50}(mg/L)	toxicity regression equation
	250	125	62.5	31.3	15.6	7.81	3.91		
1	21.87±0.41	18.57±1.42	13.37±0.41	8.18±1.08	5.35±0.41	/	/	>1000	y=-4.71+1.47x r=0.974
2	71.68±1.42	57.76±0.41	36.27±0.71	32.50±1.08	18.33±4.09	/	/	92.44	y=-3.89+1.98x r=0.973
3a	100.00±0.00	95.04±0.71	86.55±0.71	78.76±0.00	63.89±4.96	25.15±0.41	18.77±4.09	12.85	y=-3.35+3.01x r=0.975
4a	82.54±0.82	75.22±0.71	65.30±1.87	50.43±0.00	34.29±1.12	14.52±2.16	9.33±7.89	36.51	y=-3.48+2.20x r=0.980
4b	80.41±0.41	75.69±0.41	65.78±1.47	51.38±1.64	38.63±0.82	15.70±0.71	9.56±0.41	34.94	y=-3.31+2.10x r=0966
4c	84.66±1.08	76.87±2.16	68.84±1.23	57.75±0.41	41.70±1.47	19.00±1.78	10.27±0.82	28.91	y=-3.19+2.14x r=0.971
4d	83.71±1.42	79.47±1.78	63.18±1.42	48.31±0.71	39.10±0.71	23.49±2.83	14.99±0.00	30.62	y=-2.85+1.92x r=0.992
4e	68.38±1.64	61.06±0.71	45.48±1.87	32.26±3.19	24.48±3.35	/	/	80.49	y=-3.19+1.67x r=0.990
4f	70.73±3.35	55.15±0.82	49.73±0.71	32.73±4.96	31.78±1.08	/	/	74.00	y=-2.64+1.41x r=0.942
4g	35.10±2.04	16.21±1.08	12.91±3.09	9.84±1.64	7.48±0.82	/	/	771.99	y=-4.58+1.52x r=0.908

Compd.	Testing the concentration(mg/L)							EC50(mg/L)	toxicity regression equation
	250	125	62.5	31.3	15.6	7.81	3.91		
4h	71.68±0.71	70.97±3.54	50.91±1.47	30.84±3.90	19.75±2.16	14.75±0.41	6.49±2.45	`	y=-3.79+2.08x r=0.977
4i	47.13±2.16	30.14±0.82	18.33±1.64	11.96±1.08	10.54±2.28	/	/	330.96	y=-4.56+1.78x r=0.956
4j	83.01±3.75	79.70±0.82	62.94±1.67	50.67±0.41	39.81±1.23	28.69±4.97	20.66±3.75	26.90	y=-2.42+1.70x r=0.989
4k	85.60±1.08	81.59±0.00	71.20±4.15	59.64±2.12	43.35±0.71	23.49±4.83	14.99±0.00	24.09	y=-2.83+2.02x r=0.984
4l	76.87±1.08	69.55±1.42	53.74±1.08	35.56±1.42	21.40±2.12	/	/	59.23	y=-3.83+2.16x r=0.988
4m	86.79±0.82	76.63±2.04	65.07±1.08	52.56±0.71	40.76±1.47	11.21±1.08	4.12±4.55	34.65	y=-4.26+2.68x r=0.949
4n	83.25±0.82	71.91±0.71	57.28±2.04	48.78±1.47	37.45±0.41	/	/	35.03	y=-2.69+1.74x r=0.984
4o	60.11±0.82	51.61±3.90	33.91±0.82	27.30±5.36	19.75±0.41	/	/	136.85	y=-3.36+1.57x r=0.983
4p	78.76±2.55	71.44±1.08	61.29±3.56	48.55±2.68	38.40±1.23	/	/	33.22	y=-2.31+1.52x r=0.998
4q	70.97±3.09	66.25±1.78	53.97±3.24	40.99±2.95	32.50±2.16	/	/	52.24	y=-2.47+1.44x r=0.985
4r	61.06±1.87	56.57±1.08	49.49±1.08	38.87±3.49	32.50±2.95	/	/	80.55	y=-1.97+1.03x r=0.983

Compd.	Testing the concentration(mg/L)							EC_{50}(mg/L)	toxicity regression equation
	250	125	62.5	31.3	15.6	7.81	3.91		
4s	64.84±1.47	57.04±0.82	48.31±0.71	36.98±1.23	25.18±1.78	/	/	82.85	y=-2.72+1.42x r=0.986
5a	42.44±0.82	30.07±2.45	15.97±4.97	9.14±1.08	8.90±1.08	/	/	371.75	y=-4.77+1.83x r=0.938
5b	40.06±8.17	27.21±1.42	10.07±0.71	6.76±0.82	4.85±0.82	/	/	364.55	y=-5.9+2.25x r=0.942
5c	46.72±2.95	26.97±4.82	22.58±2.95	21.98±2.95	20.08±1.42	/	/	639.90	y=-2.66+0.920x r=0.737
5d	34.11±1.08	25.32±2.68	19.04±2.16	14.13±3.19	8.66±1.42	/	/	769.85	y=-3.93+1.37x r=0.994
5e	29.59±2.95	14.37±0.00	6.53±0.71	5.33±3.27	0.57±0.82	/	/	522.47	y=-8.46+3.21x r=0.911
5f	28.17±0.82	21.03±0.82	12.67±2.95	8.90±1.78	8.18±0.82	/	/	>1000	y=-4.17+1.32x r=0.942
5g	34.11±1.47	13.90±1.08	7.95±0.00	6.76±0.82	5.33±1.64	/	/	652.17	y=-5.23+1.74x r=0.848
5h	50.76±2.55	39.35±2.12	26.83±2.95	20.32±3.49	10.80±2.12	/	/	233.73	y=-4.10+1.73x r=0.989
5i	20.79±	12.47±3.27	10.07±1.87	7.23±4.25	2.48±4.09	/	/	>1000	y=-5.50+1.75x r=0.932
5j	44.10±3.49	27.21±1.42	19.99±0.71	14.85±0.41	13.42±2.28	/	/	474.68	y=-2.55+2.56x r=0.921

Compd.	Testing the concentration(mg/L)							EC50(mg/L)	toxicity regression equation
	250	125	62.5	31.3	15.6	7.81	3.91		
5k	40.06±4.91	34.35±3.24	24.94±1.42	15.32±0.82	14.85±2.95	/	/	489.08	y=-3.36+1.25x r=0.952
5l	33.64±0.71	18.65±4.27	15.97±0.41	9.61±2.49	8.18±2.64	/	/	33.64	y=-4.17+1.32x r=0.942
5m	35.78±2.55	26.03±3.90	20.22±1.08	19.13±1.47	14.37±0.00	/	/	>1000	y=-2.92+0.930x r=0.939
5n	27.93±0.71	19.36±0.71	12.67±2.16	10.09±3,75	5.09±2.55	/	/	>1000	y=-4.71+1.57x r=0.978
5o	26.26±2.16	23.65±6.75	11.73±2.95	5.81±1.42	2.99±3.74	/	/	634.71	y=-6.41+2.36x r=0.959
tricyclazole	100.00±0.00	60.68±1.08	34.77±1.08	15.46±4.91	6.59±0.71	/	/	79.39	y=-6.78+3.43x r=0.999

Note: "/" means no antifungal activity.

Table S4. EC_{50} values and toxicity regression equation of camphor derivatives against *Sphaeropsis sapinea* (mg/L)

Compd.	Testing the concentration(mg/L)							EC_{50}(mg/L)	toxicity regression equation
	250	125	62.5	31.3	15.6	7.81	3.91		
1	13.94±0.68	12.50±0.68	6.97±0.59	5.77±0.68	5.29±0.68	/	/	>1000	y=-4.18+0.99x r=0.915
2	59.55±0.00	46.29±0.34	27.64±3.79	14.16±4.14	7.42±0.68	/	/	159.50	y=-5.47+2.48x r=0.995
3a	87.50±0.68	84.38±0.34	73.80±0.90	66.59±0.34	53.13±1.56	28.10±0.67	22.62±0.34	17.09	y=-2.31+1.86x r=0.975
4a	68.03±0.34	60.10±2.97	42.79±2.72	26.20±1.23	13.94±0.68	/	/	93.51	y=-4.33+2.19x r=0.984
4b	63.70±0.90	54.09±0.34	40.39±1.48	26.20±1.89	19.47±2.90	/	/	109.93	y=-3.5+1.72x r=0.993
4c	73.56±0.90	61.06±0.59	43.75±2.04	25.48±0.68	12.50±1.36	/	/	86.14	y=-4.81+2.43x r=0.993
4d	68.99±2.12	56.73±0.00	40.39±0.90	23.32±0.34	12.26±3.35	/	/	101.23	y=-4.67+2.32x r=0.994
4e	71.39±0.34	51.68±0.59	38.46±0.90	27.89±0.00	18.27±0.90	/	/	100.08	y=-3.87+1.94x r=0.987
4f	23.08±0.90	22.12±1.18	20.91±0.34	15.39±1.48	13.94±1.36	/	/	>1000	y=-2.46+0.560x r=0.902
4g	6.25±0.88	3.85±1.80	3.85±1.80	0.72±0.90	0.72±0.90	/	/	>1000	y=-7.46+2.04x r=0.857

Compd.	Testing the concentration(mg/L)							EC_{50}(mg/L)	toxicity regression equation
	250	125	62.5	31.3	15.6	7.81	3.91		
4h	77.64±0.88	74.04±3.58	50.96±1.18	22.60±0.34	12.50±0.68	/	/	69.47	y=-5.34+2.88x r=0.961
4i	13.88±0.62	9.69±1.65	9.03±0.31	6.39±0.62	4.85±1.43	/	/	>1000	y=-4.05+0.920x r=0.973
4j	71.15±1.36	61.89±0.62	53.52±0.82	42.95±1.12	30.62±1.08	/	/	54.04	y=-2.43+1.4x r=0.994
4k	72.25±1.43	59.91±1.36	46.26±0.31	31.94±0.54	20.26±1.12	/	/	77.58	y=-3.65+1.93x r=0.999
4l	62.78±0.31	50.66±1.12	39.87±0.54	25.33±2.35	16.96±1.73	/	/	120.53	y=-3.69+1.77x r=0.996
4n	60.79±1.65	50.00±0.31	36.78±1.12	31.94±3.37	12.12±1.43	/	/	127.07	y=-3.91+2.86x r=0.929
4o	62.78±0.54	23.13±1.36	16.30±0.82	16.08±0.54	14.32±0.82	/	/	>1000	y=-2.98+0.880x r=0.861
4p	54.63±0.54	48.24±0.82	33.04±0.62	17.84±1.36	6.61±1.12	/	/	162.35	y=-5.2+2.37x r=0.951
4q	23.79±0.54	14.32±1.12	14.10±1.08	12.12±0.54	7.71±0.31	/	/	>1000	y=-3.53+0.940x r=0.896
4r	63.44±0.54	55.73±1.08	44.93±0.62	29.96±1.43	14.98±0.62	/	/	101.04	y=-3.77+1.88x r=0.961
4s	66.52±0.54	59.03±0.93	50.88±1.12	37.67±0.62	19.60±1.36	/	/	76.73	y=-3.19+1.68x r=0.945

Compd.	Testing the concentration(mg/L)							EC_{50}(mg/L)	toxicity regression equation
	250	125	62.5	31.3	15.6	7.81	3.91		
5a	17.75±1.98	15.06±1.10	14.61±0.64	14.38±0.95	13.48±0.84	/	/	>1000	y=-2.16+0.230x r=0.836
5b	19.10±0.00	17.98±0.84	17.75±1.10	15.73±0.95	12.81±0.64	/	/	>1000	y=-2.28+0.370x r=0.871
5c	22.70±0.84	21.35±3.18	20.45±1.10	19.10±1.10	15.96±2.77	/	/	>1000	y=-2.00+0.340x r=0.916
5d	22.47±0.95	21.57±0.32	20.90±0.84	18.88±0.84	17.53±2.08	/	/	>1000	y=-1.84+0.260x r=0.959
5e	14.38±0.00	13.48±1.15	13.26±0.84	12.81±0.64	11.91±0.64	/	/	>1000	y=-2.18+0.160x r=0.974
5f	15.06±1.10	14.16±1.27	11.01±2.91	9.21±0.64	8.32±1.10	/	/	>1000	y=-3.15+0.610x r=0.970
5g	17.08±1.46	16.18±2.22	13.26±0.64	12.14±0.32	11.46±1.68	/	/	>1000	y=-2.58+0.420x r=0.954
5h	21.57±1.15	16.85±1.27	16.18±1.39	14.83±0.32	12.58±0.32	/	/	>1000	y=-2.51+0.480x r=0.933
5i	16.85±0.64	13.03±0.95	8.54±1.15	6.52±1.27	5.62±0.00	/	/	>1000	y=-4.19+1.07x r=0.974
5j	13.48±0.84	12.36±1.91	11.91±0.64	11.24±0.84	9.66±0.00	/	/	>1000	y=-2.54+0.290x r=0.944
5k	15.73±0.95	13.26±1.27	13.03±0.95	12.36±0.00	12.36±0.00	/	/	>1000	y=-2.26+0.210x r=0.773

Compd.	Testing the concentration(mg/L)							EC_{50}(mg/L)	toxicity regression equation
	250	125	62.5	31.3	15.6	7.81	3.91		
5l	16.40±0.00	15.96±2.54	15.06±2.20	14.16±1.68	13.26±1.68	/	/	>1000	y=-2.12+0.210x r=0.984
5m	14.38±1.65	13.26±0.64	12.36±0.00	11.46±0.64	10.56±1.27	/	/	>1000	y=-2.48+0.290x r=0.999
5n	11.69±0.95	11.69±1.91	10.34±0.55	9.66±1.10	8.32±0.00	/	/	>1000	y=-2.75+0.320x r=0.929
5o	12.58±2.08	11.01±1.10	9.66±1.10	9.66±1.10	8.32±1.10	/	/	>1000	y=-2.82+0.360x r=0.944
tricyclazole	50.56±1.68	23.82±0.95	8.76±1.27	6.52±2.54	3.15±1.59	/	/	268.37	y=-6.93+2.79x r=0.960

Note: "/" means no antifungal activity.

Table S5. EC_{50} values and toxicity regression equation of camphor derivatives against *Fusarium oxysporum* (mg/L)

Compd.	Testing the concentration(mg/L)							EC_{50}(mg/L)	toxicity regression equation
	250	125	62.5	31.3	15.6	7.81	3.91		
1	23.72±3.45	22.45±4.92	8.92±0.77	4.08±0.88	0.51±0.00	/	/	597.01	y=-8.69+3.37x r=0.897
2	70.15±3.06	52.29±1.93	25.51±3.53	24.23±5.98	22.19±4.35	/	/	118.14	y=-3.76+1.81x r=0.851
3a	94.64±1.33	93.11±0.77	90.05±1.33	72.45±0.77	35.97±3.78	/	/	19.30	y=-3.48+2.83x r=0.896
4a	94.13±1.17	82.14±1.17	70.92±7.38	48.47±7.67	14.54±1.17	/	/	38.56	y=-5.7+3.55x r=0.974
4b	94.39±1.17	86.48±5.94	86.22±8.10	73.98±5.02	46.94±6.90	26.80±4.42	19.38±2.64	15.89	y=-2.91+2.42x r=0.971
4c	87.75±1.33	87.24±1.93	84.18±4.22	75.84±5.44	46.89±3.84	26.80±0.34	19.38±0.34	16.24	y=-2.57+2.21x r=0.926
4d	88.52±3.83	83.93±0.77	83.67±3.53	69.13±4.22	46.94±2.34	24.17±0.90	9.33±0.34	19.97	y=-3.23+2.41x r=0.934
4e	91.84±2.34	78.83±3.93	64.54±2.69	62.24±5.21	46.17±5.74	/	/	20.05	y=-2.62+1.98x r=0.926
4f	41.58±4.22	20.91±0.88	18.11±2.76	14.28±1.53	13.77±1.77	/	/	734.52	y=-3.42+1.15x r=0.807
4g	8.16±0.00	4.09±4.68	3.07±5.43	3.06±0.88	1.78±0.00	/	/	>1000	y=-5.38+1.16x r=0.900

Compd.	Testing the concentration(mg/L)							EC_{50}(mg/L)	toxicity regression equation
	250	125	62.5	31.3	15.6	7.81	3.91		
4h	92.86±2.21	89.54±6.19	81.12±4.98	47.45±0.59	27.80±4.61	/	/	29.85	y=-4.52+3.09x r=0.958
4i	24.74±2.90	8.16±2.65	5.35±4.22	4.84±1.17	2.04±0.00	/	/	899.39	y=-6.25+2.02x r=0.908
4j	88.27±4.22	86.99±5.98	74.74±2.76	39.79±4.68	18.87±3.34	/	/	38.55	y=-4.90+3.08x r=0.934
4k	88.52±7.95	77.04±3.98	68.37±1.17	61.22±3.53	42.85±3.19	/	/	21.03	y=-2.39+1.80x r=0.975
4l	87.75±2.02	86.22±2.65	73.98±5.78	71.94±4.86	53.06±7.55	22.73±1.47	5.03±1.22	20.80	y=-3.61+2.58x r=0.893
4m	92.35±2.65	89.80±0.88	73.98±1.33	55.61±2.02	36.73±2.34	/	/	25.05	y=-3.70+2.66x r=0.981
4n	92.35±1.33	84.95±1.77	80.87±4.59	74.74±3.34	41.58±3.09	/	/	16.33	y=-2.48+2.09x r=0.913
4o	72.45±31.33	68.37±3.62	48.98±4.98	42.34±6.42	41.58±3.78	/	/	42.63	y=-1.99+1.23x r=0.896
4p	63.68±3.40	63.22±3.47	59.31±3.58	48.28±0.00	38.62±2.49	/	/	38.88	y=-1.34+0.840x r=0.877
4q	42.07±2.39	34.25±1.74	30.81±0.8	28.51±1.74	20.92±2.42	/	/	730.69	y=-2.17+0.760x r=0.956
4r	71.95±0.80	66.67±2.61	62.07±7.83	56.09±1.59	39.77±4.84	/	/	26.50	y=-1.5+1.05x r=0.929

Compd.	Testing the concentration(mg/L)							EC_{50}(mg/L)	toxicity regression equation
	250	125	62.5	31.3	15.6	7.81	3.91		
4s	58.62±0.00	52.87±1.05	51.03±0.00	49.89±0.80	40.46±0.40	/	/	56.45	y=-0.93+0.530x r=0.892
5a	25.06±8.17	23.91±2.22	22.53±3.80	21.61±2.61	20.00±3.65	/	/	>1000	y=-1.66+0.240x r=0.992
5b	47.13±1.99	31.26±6.18	26.21±4.83	23.22±4.89	20.23±1.44	/	/	486.83	y=-2.64+0.970x r=0.892
5c	29.66±2.49	26.21±1.19	25.75±1.44	25.06±0.80	19.54±5.74	/	/	>1000	y=-1.79+0.390x r=0.841
5d	23.22±3.40	22.76±3.01	20.00±2.07	17.01±1.05	16.55±4.20	/	/	>1000	y=-2.12+0.40x r=0.939
5e	7.59±4.52	6.67±2.11	6.44±1.05	5.52±2.49	5.06±2.79	/	/	>1000	y=-3.35+0.350x r=0.976
5f	7.36±2.79	6.21±0.69	3.91±0.8	3.45±0.00	2.53±0.40	/	/	>1000	y=-4.79+0.950x r=0.973
5g	8.74±2.42	6.67±2.87	5.29±2.11	4.83±6.90	3.45±2.76	/	/	>1000	y=-4.22+0.770x r=0.977
5h	30.12±3.80	23.68±2.79	21.38±1.19	20.46±0.40	19.77±2.22	/	/	>1000	y=-1.99+0.430x r=0.836
5j	18.33±2.38	17.88±4.24	17.65±1.59	16.29±5.00	14.94±2.18	/	/	>1000	y=-1.95+0.200x r=0.898
5k	11.77±2.35	9.28±2.38	8.83±2.07	8.60±2.07	4.53±0.39	/	/	>1000	y=-3.69+0.710x r=0.786

Compd.	Testing the concentration(mg/L)							EC_{50}(mg/L)	toxicity regression equation
	250	125	62.5	31.3	15.6	7.81	3.91		
5l	6.34±1.36	5.89±1.57	5.43±0.78	5.21±1.04	4.76±0.39	/	/	>1000	y=-3.28+0.250x r=0.991
5m	16.07±0.39	15.39±2.83	13.35±0.78	12.00±4.42	5.21±2.74	/	/		y=-3.69+0.930x r=0.750
5n	2.95±0.00	2.27±0.00	1.13±0.78	0.91±0.00	0.45±1.18	/	/	>1000	y=-7.20+1.58x r=0.972
5o	/	/	/	/	/	/	/	/	/
tricyclazole	95.69±1.24	71.53±5.29	39.71±0.00	21.05±	9.81±3.39	/	/	66.78	y=-7.67+4.28x r=0.959

Note: “/” means no antifungal activity.

Table S6. EC_{50} values and toxicity regression equation of camphor derivatives against *Trametes versicolor* (mg/L)

Compd.	Testing the concentration(mg/L)											EC_{50} (mg/L)	toxicity regression equation
	250	125	62.5	31.3	15.6	7.81	3.91	1.95	0.977	0.488	0.244		
1	25.36±1.17	15.80±0.68	13.40±0.68	9.57±1.17	7.18±2.44	/	/	/	/	/	/	>1000	y=-3.99+1.17x r=0.975
2	61.20±1.17	56.90±2.03	14.80±4.44	10.00±2.44	7.66±0.68	/	/	/	/	/	/	151.00	y=-6.31+2.78x r=0.890
3a	100.00±0.00	100.00±0.00	98.60±0.00	97.60±0.68	92.80±1.17	91.20±0.90	89.71±2.64	85.20±0.68	77.71±2.69	46.61±1.88	33.21±3.84	0.43	y=0.780+1.91x r=0.954
4a	92.30±0.68	89.00±0.68	87.10±1.17	79.40±0.68	59.80±2.03	52.30±3.38	44.90±1.79	38.70±1.22	12.44±2.34	7.02±1.79	5.02±1.69	6.80	y=-1.64+1.86x r=0.971
4b	53.60±0.68	50.20±1.79	54.50±0.68	42.60±1.17	32.10±0.68	/	/	/	/	/	/	99.70	y=-1.39+0.700x r=0.727
4c	85.60±0.00	78.00±0.68	67.90±0.68	53.60±2.71	22.50±4.69	/	/	/	/	/	/	35.80	y=-3.73+2.38x r=0.945
4d	93.80±0.6	88.50±1	85.20±	78.50±	71.80±0	58.61±	45.22±	19.62±	7.89±0.	2.63±0.	1.20±0.	7.67	y=-2.4+2.36x

Compd.	Testing the concentration(mg/L)											EC_{50} (mg/L)	toxicity regression equation
	250	125	62.5	31.3	15.6	7.81	3.91	1.95	0.977	0.488	0.244		
	8	.17	1.79	1.17	.68	2.37	0.34	4.06	90	90	34		r=0.953
4e	44.00±5.11	30.60±1.35	28.20±1.17	25.40±2.03	18.20±1.17	/	/	/	/	/	/	593.00	y=-2.58+0.930x r=0.927
4f	48.30±3.52	37.30±3.38	29.70±3.10	26.30±3.38	22.00±1.79	/	/	/	/	/	/	361.00	y=-2.48+0.970x r=0.964
4g	8.13±2.03	7.65±1.35	7.18±0.68	6.70±2.34	6.22±0.68	/	/	/	/	/	/	>1000	y=-2.99+0.240x r=0.999
4h	60.80±1.35	54.50±0.68	45.50±1.17	32.50±1.17	23.00±0.68	/	/	/	/	/	/	102.00	y=-2.81+1.4x r=0.982
4i	15.30±5.37	12.90±1.79	8.13±2.03	7.18±4.12	3.83±2.34	/	/	/	/	/	/	>1000	y=-4.56+1.22x r=0.955
4j	79.60±0.70	72.10±1.86	51.70±0.70	24.90±2.54	10.90±0.70	/	/	/	/	/	/	68.50	y=-5.52+2.98x r=0.976
4k	88.60±0.70	83.60±1.22	71.60±4.22	60.70±0.70	46.80±0.70	41.63±0.59	36.84±1.55	9.33±0.9	4.78±1.9	1.67±0.59	/	17.20	y=-2.79+2.16x r=0.949

Compd.	Testing the concentration(mg/L)											EC_{50} (mg/L)	toxicity regression equation
	250	125	62.5	31.3	15.6	7.81	3.91	1.95	0.977	0.488	0.244		
4l	49.80±0.70	42.40±0.70	33.80±1.86	17.40±3.07	8.46±1.86	/	/	/	/	/	/	204.00	y=-4.56+1.99x r=0.949
4m	56.20±0.70	54.20±0.70	48.30±0.70	36.30±1.86	20.40±0.70	/	/	/	/	/	/	108.00	y=-2.97+1.31x r=0.883
4n	43.80±0.70	42.80±2.81	41.30±1.41	36.30±4.93	21.90±3.52	/	/	/	/	/	/	343.00	y=-1.93+0.770x r=0.746
4o	87.07±1.86	84.08±0.70	78.61±0.70	74.12±0.70	63.60±0.70	62.68±4.23	58.13±2.64	32.54±3.1	17.22±1.35	6.22±0.68	1.20±0.34	6.89	y=-1.86+1.87x r=0.872
4p	57.20±1.86	56.70±6.45	56.20±0.70	55.70±0.70	49.80±1.41	32.54±2.03	16.27±1.79	2.39±0.00	1.67±0.59	0.72±0.34	/	18.90	y=-3.99+3.14x r=0.955
4q	68.70±2.44	61.20±1.22	49.80±0.70	41.80±1.22	39.30±1.86	/	/	/	/	/	/	51.50	y=-1.83+1.07x r=0.960
4r	41.30±0.70	36.80±0.70	36.30±0.70	27.40±0.70	25.90±1.86	/	/	/	/	/	/	>1000	y=-1.79+0.610x r=0.921

Compd.	Testing the concentration(mg/L)											EC_{50} (mg/L)	toxicity regression equation
	250	125	62.5	31.3	15.6	7.81	3.91	1.95	0.977	0.488	0.244		
4s	32.30±0.70	31.30±2.11	27.90±2.54	23.90±0.00	20.40±0.701	/	/	/	/	/	/	>1000	y=-1.97+0.540x r=0.961
5a	71.60±4.22	70.10±1.41	69.70±0.00	68.70±1.22	67.70±0.70	61.96±3.84	53.83±0.90	36.60±0.90	22.49±4.69	12.92±4.74	9.09±0.68	4.31±3.85	y=-1.19+1.82x r=0.980
5b	62.20±1.86	58.70±0.70	58.20±2.11	57.70±0.70	57.20±1.86	42.34±0.34	18.42±1.88	4.78±0.90	3.59±2.77	1.56±0.61	/	11.40	y=-3.35+3.10x r=0.972
5c	64.70±1.41	63.70±0.70	63.20±1.86	62.20±0.70	61.70±1.41	52.63±2.34	43.06±2.95	23.92±2.11	0.57±0.75	0.10±0.10	0.10±0.20	7.40	y=-4.25+4.87x r=0.892
5d	71.40±0.70	64.00±1.84	49.80±1.21	47.30±1.39	40.90±1.21	/	/	/	/	/	/	40.90	y=-1.74+1.08x r=0.948
5e	51.20±0.00	50.20±0.70	48.30±2.41	42.90±1.39	39.40±1.21	/	/	/	/	/	/	140.00	y=-0.90+0.42x r=0.933
5f	51.70±0.70	51.20±1.21	48.30±1.21	47.30±3.48	46.30±3.04	/	/	/	/	/	/	102.00	y=-0.39+0.20x r=0.946

Compd.	Testing the concentration(mg/L)											EC_{50} (mg/L)	toxicity regression equation
	250	125	62.5	31.3	15.6	7.81	3.91	1.95	0.977	0.488	0.244		
5g	58.10±0.70	57.60±0.70	56.70±1.84	55.70±2.09	54.70±1.84	52.15±2.22	48.33±2.03	26.56±3.99	21.29±0.34	14.11±2.64	11.00±2.11	7.85	y=-1.27+1.41x r=0.955
5h	64.00±0.70	63.10±0.00	62.60±0.70	61.60±1.21	57.10±2.09	38.52±2.77	15.31±1.17	9.57±0.59	5.50±1.69	1.67±0.59	0.57±0.29	12.30	y=-3.17+2.93x r=0.989
5j	63.10±0.00	57.60±2.51	56.20±0.70	54.20±0.00	51.20±0.00	42.82±1.79	31.58±0.68	18.90±2.11	17.46±2.55	15.55±2.89	11.96±1.35	16.20	y=-1.44+1.15x r=0.942
5k	79.80±2.09	79.40±0.70	78.90±1.21	78.90±0.70	78.60±1.39	75.12±2.22	72.49±2.89	11.72±3.04	3.83±2.34	1.67±1.01	0.57±0.54	4.86	y=-2.81+3.54x r=0.903
5l	68.00±0.70	65.50±1.39	64.50±1.21	64.00±2.51	63.10±2.09	60.53±2.69	58.37±2.03	32.78±3.43	17.46±1.17	6.22±0.68	3.11±1.01	5.09	y=-1.71+2.38x r=0.934
5m	53.20±0.70	34.50±0.70	24.60±8.70	21.70±0.00	20.20±0.00	/	/	/	/	/	/	295.00	y=-3.03+1.21x r=0.866
5n	27.60±1.21	26.10±0.00	25.60±0.70	25.10±3.48	24.60±4.18	/	/	/	/	/	/	>1000	y=-1.27+0.120x r=0.930

Compd.	Testing the concentration(mg/L)											EC_{50} (mg/L)	toxicity regression equation
	250	125	62.5	31.3	15.6	7.81	3.91	1.95	0.977	0.488	0.244		
5o	28.10±0.70	20.20±1.21	19.70±0.70	15.30±3.04	9.85±2.41	/	/	/	/	/	/	>1000	y=-3.25+0.960x r=0.937
tricyclazole	80.30±0.70	48.30±0.00	24.60±1.21	10.80±1.84	7.39±0.70	/	/	/	/	/	/	118.20	y=-6.79+3.29x r=0.966

Note: "/" means no antifungal activity.

Table S7. EC_{50} values and toxicity regression equation of carbendazim against six fungi (mg/L)

Fungi	Testing the concentration (mg/L)											EC_{50}(mg/L)	toxicity regression equation
	15.6	7.81	3.91	1.95	0.977	0.488	0.244	0.122	0.061	0.031	0.015		
PN	100.00±0.00	94.61±1.2	91.91±0.90	71.32±0.90	13.97±3.16	11.52±0.3	5.15±0.90	/	/	/	/	1.49	y=-0.690+4.26x r=0.937
FV	100.00±0.00	100.00±0.00	100.00±0.00	100.00±0.00	80.70±0.31	34.34±3.77	7.02±0.81	5.26±0.53	4.51±1.41	1.00±2.40	-0.75±	0.55	y=0.670+3.58x r=0.898
CG	98.54±0.00	98.54±0.00	98.54±0.00	97.49±0.61	95.24±0.31	94.99±1.34	88.47±0.81	64.41±0.81	28.82±0.61	6.77±1.68	2.26±1.11	0.20	y=1.85+2.90x r=0.922
SS	82.35±0.00	65.44±3.16	50.74±1.38	19.61±0.60	7.35±2.08	5.88±1.04	5.64±1.08	/	/	/	/	4.67	y=-1.82+2.66x r=0.941
FO	100.00±0.00	99.51±0.60	99.02±0.60	98.03±1.20	88.24±0.00	37.50±2.90	30.63±1.97	/	/	/	/	0.45	y=1.79+4.58x r=0.946
TV	77.69±1.96	75.34±0.64	73.49±0.32	72.70±0.64	62.73±1.29	35.17±3.79	13.39±5.57	6.40±0.91	6.19±0.57	5.77±0.32	5.77±0.64	1.20	y=-0.250+1.7

	1x<br>r=0.904

Note: PN, *Phytophthora nicotianae*; FV, *Fusarium verticillioides*; CG, *Colletotrichum gloeosporioides*; SS, *Sphaeropsis sapinea*; FO, *Fusarium oxysporum*; TV, *Trametes versicolor*. "/" means no antifungal activity.
